# A Causal Association Between Drug Use and Cognitive Impairment: A Two‐Sample Mendelian Randomization Study

**DOI:** 10.1002/brb3.71057

**Published:** 2025-11-11

**Authors:** Bing Yan, Zhugui Chen, Dan Yang

**Affiliations:** ^1^ Department of Anesthesiology Central People's Hospital of Zhanjiang Zhanjiang China

**Keywords:** cognitive impairment, dementia, drug use, Mendelian randomization

## Abstract

**Introduction:**

Observational studies have suggested a link between certain drugs and cognitive impairment. In this study, a two‐sample Mendelian randomization (MR) approach was used to investigate the causal relationship between drug use and different types of dementia.

**Methods:**

We utilized summary data from genome‐wide association studies (GWAS) with populations of European ancestry. The primary analysis was conducted using inverse‐variance weighted (IVW) methods; MR Egger, weighted median, and weighted model methods were used to validate the results. Horizontal pleiotropy and outlier detection were assessed via MR Egger and MR‐PRESSO, respectively; Cochran's *Q* test evaluated heterogeneity, whereas leave‐one‐out analyses were used to evaluate the presence of predominant instrumental variables (IVs). Statistical power was calculated using the online tool mRnd to assess the robustness of MR estimates.

**Results:**

The IVW analysis indicated that antithrombotic agents (odds ratio [OR] = 2.12, 95% confidence interval [CI] = 1.03–4.34, *p* = 0.04), HMG CoA reductase inhibitors (OR = 1.36, 95% CI = 1.07–1.72, *p* = 0.01), and salicylic acid and derivatives (OR = 2.77, 95% CI = 1.44–5.32, *p* = 0.002) are a strong risk factor for dementia with Lewy bodies (DLB); diuretics (OR = 1.13, 95% CI = 1–1.27, *p* = 0.047) and calcium channel blockers (OR = 1.16, 95% CI = 1.04–1.29, *p* = 0.007) might be a risk factor for vascular dementia; thyroid preparations (OR = 1.03, 95% CI = 1.01–1.06, *p* = 0.01), diuretics (OR = 1.06, 95% CI = 1.02–1.11, *p* = 0.004), and immunosuppressants (OR = 1.07, 95% CI = 1.01–1.12, *p* = 0.001), are a risk factor for frontotemporal dementia; HMG CoA reductase inhibitors is a risk factor for dementia in Alzheimer disease (OR = 1.32, 95% CI = 1.05–1.65, *p* = 0.01). Moreover, antihistamines for systemic use showed some causal effect on cognitive performance (*p* < 0.05). The MR‐Egger regression indicated that results were unaffected by horizontal pleiotropy. Moreover, no heterogeneity was detected. However, power analyses revealed that several associations had relatively low statistical power, suggesting that these findings should be interpreted with caution.

**Conclusions:**

Our findings suggest that certain drugs may causally affect dementia. Larger, well‐designed MR studies may help establish the causal status of these dementia risk factors.

## Graphical abstract

The schematic diagram illustrates the overall design of this two‐sample Mendelian randomization (MR) study investigating the causal association between drug use and cognitive impairment. Genetic variants significantly associated with drug use were selected as instrumental variables from large‐scale GWAS datasets. These variants were then used to estimate their causal effects on cognitive impairment through multiple MR analytical methods. The findings provide genetic evidence supporting a potential causal relationship between specific drug use and cognitive impairment risk.

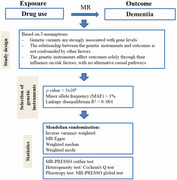

## 1 Introduction

Dementia encompasses a range of cognitive impairments characterized by deterioration in memory, perception, reasoning, language, and other psychological abilities, leading to deficits in instrumental activities of daily living (IADLs) and markedly impairing quality of life in affected individuals (Franjić 2022; Giannouli 2024). Several different types of dementia have been recognized, including Alzheimer's disease, vascular dementia, Lewy body dementia (LBD), and frontotemporal dementia (FTD), with the former one being the most common, accounting for 60%–80% of cases (Foran et al. 2021).

The causes of dementia tend to vary, ranging from neurodegenerative diseases and vascular issues to environmental factors and genetic predispositions (James and Bennett 2019). Symptoms can substantially differ among affected individuals; however, commonly observed manifestations are memory loss, confusion, disorientation, communication issues, problem‐solving difficulties, changes in decision‐making (Raimo et al. 2024), and changes in mood, personality, and behavior (Chen et al. 2024; Giannouli and Kampakis 2025). The incidence tends to increase with age, doubling approximately every 5 years after age 65 (Shaw et al. 2010). Furthermore, comorbid conditions are common in older adults, and they can include hypertension, heart disease, stroke, diabetes, chronic obstructive pulmonary disease, obesity, and others (Fox et al. 2014). The effects of drugs used to manage these conditions on dementia tend to vary depending on several factors, including the type of medication, the underlying condition, and patient's characteristics. For example, previous studies have shown that long‐term use of corticosteroids may be associated with cognitive impairment and an increased risk of dementia (Shorey et al. 2023). Similarly, medications used for anxiety and sleep disorders, such as benzodiazepines, may also contribute to cognitive impairment and increase the risk of developing dementia (Pariente et al. 2016). On the contrary, it has been reported that medications like metformin used for the management of diabetes may actually exert neuroprotective effects, thus potentially ameliorating the risk of cognitive decline (Ralph and Espinet 2018).

As drugs can have a significant effect on cognitive health in general, dementia in particular (Richardson et al. 2018), understanding the relationship between these drugs and dementia is of crucial importance for early detection, management, and support of these individuals.

Mendelian randomization (MR) uses genetic variation as a natural experiment to analyze the causal relations between health outcomes in observational data and potentially modifiable risk factors (Davies et al. 2018). Findings from MR studies depend on specific assumptions and are based on Mendel's first and second laws of genetic inheritance (Sanderson et al. 2022). Thus far, MR has been increasingly applied to examine causal inference of risk factors in dementia. For example, Anderson et al. (2024) used MR to examine factors that may prevent or delay dementia in patients with Alzheimer's disease. Kuzma et al. (2018) systematically review MR studies investigating causal relationships between risk factors and global cognitive function or dementia. However, the link between drug use and dementia using the MR method has not yet been investigated. Recent MR‐based studies have further explored the influence of metabolic, vascular, and lifestyle‐related exposures on dementia risk (Pinheiro et al. 2025), providing methodological support for extending this framework to the study of drug use. However, the link between drug use and dementia using the MR method has not yet been investigated.

In this study, we performed a two‐sample MR analysis to investigate the causal relationship between drug use and cognitive impairment in patients with dementia. Given the rising global burden of dementia, understanding the impact of drugs on cognitive function is crucial for improving patient outcomes. Our findings provide new insights into how certain drugs may influence the progression of dementia, offering valuable evidence for clinicians to refine treatment strategies and guide future research into potential drug repurposing for dementia care.

## 2 Materials and Methods

### 2.1 Study Design

The MR analysis followed the Strengthening the Reporting of Observational Studies in Epidemiology using MR (STROBE‐MR) guidelines (Skrivankova et al. 2021): (1) Genetic variants are strongly associated with gene levels; (2) the relationship between the genetic instruments and outcomes is not confounded by other factors; (3) the genetic instruments affect outcomes solely through their influence on risk factors, with no alternative causal pathways.

### 2.2 Data Sources

Genome‐wide association studies (GWAS) data on cognitive ability were obtained from a large‐scale meta‐analysis published in *Nature Genetics* (2018) (Lee et al. 2018), which included ∼1.1 million individuals of European ancestry and identified 1271 independent genome‐wide significant SNPs. Educational attainment was measured as years of education, with inclusion restricted to participants aged ≥30 years. Data on vascular dementia, Parkinson's disease dementia, Alzheimer's disease dementia, and FTD were derived from the Finnish cohort R10 within the FinnGen consortium, with diagnoses based on standardized ICD codes (Table 1). LBD data were obtained from a GWAS in *Nature Genetics* (Chia et al. 2021), including 2981 cases and 2173 controls of European ancestry from 44 international cohorts, supplemented with 2218 additional control genomes (total controls = 4391).

**TABLE 1:** Data sources on cognitive ability and cognitive impairment.

Trait	GWAS ID	Sample size (case/control)	SNPs	Grch
Cognitive performance	ebi‐a‐GCST006572	257,841	10,066,414	37
Vascular dementia (F5_VASCDEM)	F5_VASCDEM	2717/393,024	16,380,457	
Dementia with Lewy bodies	ebi‐a‐GCST90001390	6618	7,593,175	38
Dementia due to Parkinson's disease	PD_DEMENTIA_EXMORE	589/184,000	16,380,459	
Dementia in Alzheimer's disease	F5_ALZHDEMENT	6145/388,560	16,380,451	
Frontotemporal dementia	https://storage.googleapis.com/finngen‐public‐data‐r10/summary_stats/finngen_R10_F5_STRESSOTH.gz	129/392,463	/	

Drug use data were obtained from a GWAS published in *Biomedicines* (2023) (Wu et al. 2019), based on ∼318,000 UK Biobank participants of European ancestry. Medication data were self‐reported and mapped to the Anatomical Therapeutic Chemical (ATC) system. Quality control excluded individuals with missing genotype data, non‐European ancestry, or insufficient medication information. Details are available in the GWAS Catalog (Table 2).

**TABLE 2:** Data sources on different drug uses.

ID	Trait(s)	Sample size
CST007922	Peptic ulcer and gastro‐esophageal reflux disease (GORD) drug use measurement	132,367
GCST007923	Drugs used in diabetes use measurement	132,367
GCST007924	Antithrombotic agent use measurement	132,367
GCST007925	Vasodilators used in cardiac diseases use measurement	132,367
GCST007926	Antihypertensive use measurement	132,367
GCST007927	Diuretic use measurement	132,367
GCST007928	Beta‐blocking agents use measurement	132,367
GCST007929	Calcium channel blockers use measurement	132,367
GCST007930	Agents acting on the renin–angiotensin system use measurement	132,367
GCST007931	HMG CoA reductase inhibitor use measurement	132,367
GCST007932	Thyroid preparation use measurement	132,367
GCST007933	Immunosuppressant use measurement	132,367
GCST007934	Nonsteroidal anti‐inflammatory and antirheumatic product use measurement	132,367
GCST007935	Drugs affecting bone structure and mineralization use measurement	132,367
GCST007936	Opioid use measurement	132,367
GCST007937	Aspirin use measurement	132,367
GCST007938	Anilide use measurement	132,367
GCST007939	Antimigraine preparation uses measurement	132,367
GCST007940	Antidepressant use measurement	132,367
GCST007941	Inhalant adrenergic use measurement	132,367
GCST007942	Glucocorticoid use measurement	132,367
GCST007943	Antihistamine use measurement	132,367
GCST007944	Antiglaucoma preparations and miotics use measurement	132,367

All datasets comprised individuals of European ancestry to ensure homogeneity and reduce confounding due to population stratification.

### 2.3 Instrumental Variables (IVs) Selection

The IVs included in this study were screened using the following criteria (Yin et al. 2022): (1) SNPs related to drug use were screened separately on the basis of *p* < 5 × 10^−8^. Then, SNPs with a minimum minor allele frequency (MAF) > 0.01 were screened. Consequently, linkage disequilibrium (LD) between SNPs was removed on the basis of the following criteria: window size = 10,000 kb, *R* ^2^ < 0.001. When the selected IVs were not present in the summary data of the outcome, SNPs with high LD (*R* ^2^ > 0.8) with the IVs as proxy SNPs were screened and replaced. *F* value was calculated for each SNP to evaluate the strength of the IV, excluding the possibility of weak instrument bias between the IV and the exposure factor. The following formula was applied: *F* = *R* ^2^(*N* − 2)/(1 − *R* ^2^), where *R* ^2^ was the proportion of exposure variance explained by the SNP in the IV, and the requirement for the *F* value was >10.

### 2.4 MR Analysis

IVW approach was used as the primary analytical method. IVW estimates the odds ratio (OR) and its 95% confidence interval (CI), providing a balanced assessment of the overall effect by assigning weights inversely proportional to the variance of each SNP (Burgess et al. 2017). MR Egger, weighted median, and weighted model methods were used to validate the robustness of the results. The weighted median method, assuming the validity of at least half of the IVs, was used to examine the exposure‐outcome relationship (Bowden et al. 2016); the MR‐Egger regression adjusts for potential pleiotropy by considering the intercept, offering a reliable causal estimate even in pleiotropic effects (Bowden et al. 2015). The analyses were conducted using R version 4.3.2 with the “two‐sample MR” package, and the results were visualized through forest plots, scatter plots, and funnel plots.

### 2.5 Sensitivity Analysis

The MR‐PRESSO method was used to identify outliers, defined by a *p* value <0.05, and to recalibrate the causal association after their exclusion, thereby adjusting for pleiotropy (Verbanck et al. 2018). To assess the impact of individual SNPs on the observed causal relationship, a leave‐one‐out (LOO) sensitivity analysis was applied (Bowden et al. 2015). Heterogeneity among the IVs was assessed using Cochran's *Q* test, with a *p* value >0.05 indicating low heterogeneity and suggesting that the variability among the IVs was random and minimally impactful on the IVW estimates (Bowden et al. 2018). MR‐Egger regression was employed to detect horizontal pleiotropy to address genetic pleiotropy, with a non‐significant intercept indicating minimal pleiotropic bias (Bowden et al. 2015).

### 2.6 Statistical Power Analysis

To further evaluate whether our analyses were sufficiently powered to detect the observed associations, we conducted statistical power calculations for binary outcomes using the mRnd online tool (https://shiny.cnsgenomics.com/mRnd/). The calculation incorporated the proportion of variance explained by the IVs (*R* ^2^), the sample size (*N*), and the number of cases within each outcome (*K*). A power greater than 80% was considered excellent, whereas results with power below this threshold were deemed underpowered.

## 3 Results

### 3.1 IV Selection

In this study, we assessed a causal association between different drug use as an exposure factor and cognitive ability/impairment as an outcome. A total of 1–184 IVs were selected. Data regarding *F*‐statistics and SNPs are shown in Tables S1–S23.

### 3.2 MR and Sensitivity Analysis

**FIGURE 1:**
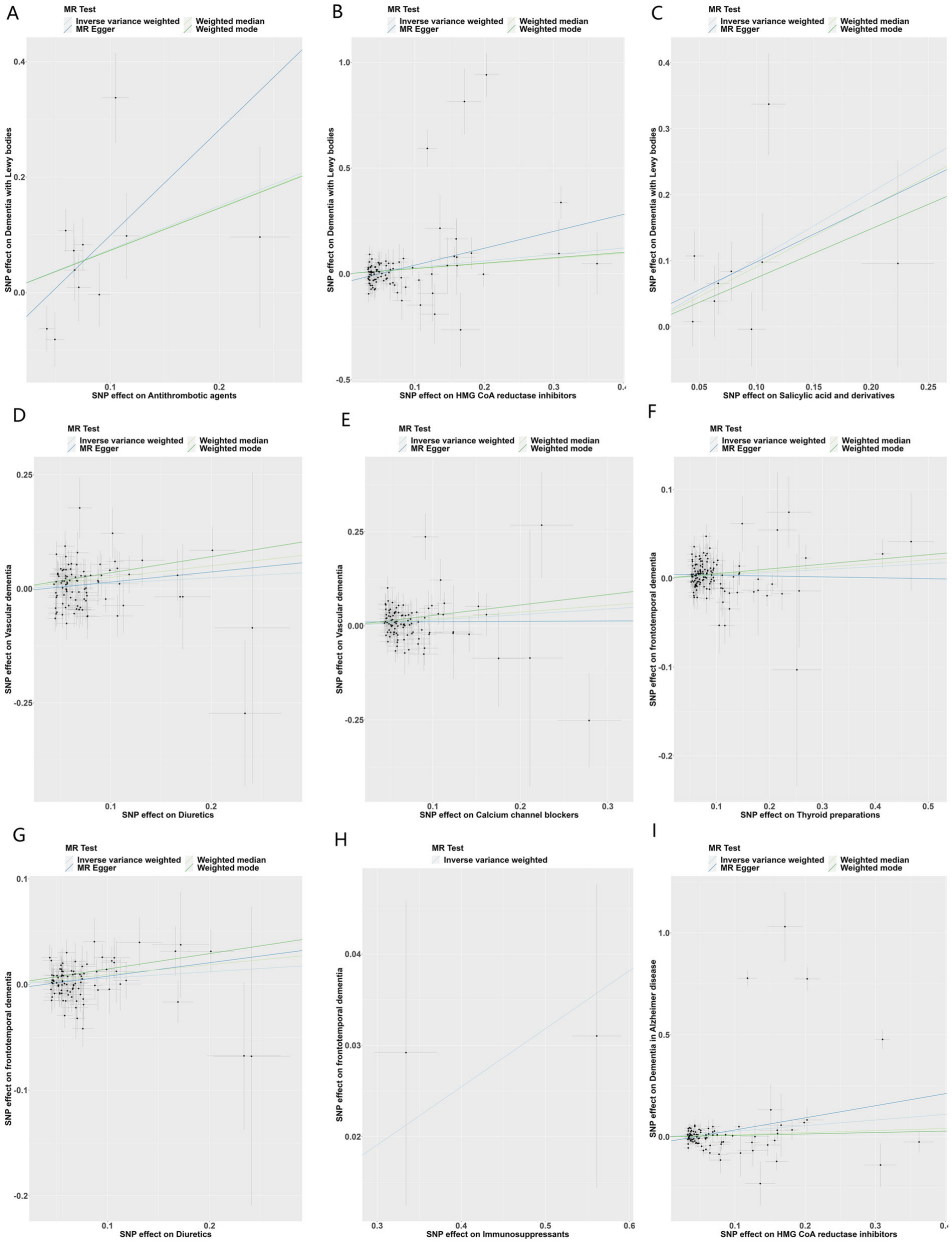
Scatter plots. The IVW analysis suggests a causal link between antithrombotic agents (A), HMG CoA reductase inhibitors (B), and salicylic acid and derivatives (C) with dementia with Lewy bodies; between diuretics (D) and calcium channel blockers (E) and vascular dementia; between thyroid preparations (F), diuretics (G), and immunosuppressants (H) and frontotemporal dementia; between HMG CoA reductase inhibitors and dementia in Alzheimer disease (I). MR, Mendelian randomization.

The IVW analysis indicated that antithrombotic agents (OR = 2.12, 95% CI = 1.03–4.34, *p* = 0.04, Table 3, Figure 1A, Figure S1), HMG CoA reductase inhibitors (OR = 1.36, 95% CI = 1.07–1.72, *p* = 0.01, Table 3, Figure 1B, Figure S2), and salicylic acid and derivatives (OR = 2.77, 95% CI = 1.44–5.32, *p* = 0.002, Table 3, Figure 1C, Figure S3) are a strong risk factor for dementia with Lewy bodies (DLB); diuretics (OR = 1.13, 95% CI = 1–1.27, *p* = 0.047, Table 3, Figure 1D, Figure S4) and calcium channel blockers (OR = 1.16, 95% CI = 1.04–1.29, *p* = 0.007, Table 3, Figure 1E, Figure S5) might be a risk factor for vascular dementia; thyroid preparations (OR = 1.03, 95% CI = 1.01–1.06, *p* = 0.01, Table 3, Figure 1F, Figure S6), diuretics (OR = 1.06, 95% CI = 1.02–1.11, *p* = 0.004, Table 3, Figure 1G, Figure S7), and immunosuppressants (OR = 1.07, 95% CI = 1.01–1.12, *p* = 0.001, Table 3, Figure 1H, Figure S8), are a risk factor for FTD; HMG CoA reductase inhibitors is a risk factor for dementia in Alzheimer disease (OR = 1.32, 95% CI = 1.05–1.65, *p* = 0.01, Table 3, Figure 1I, Figure S9). MR‐Egger regression results suggested that horizontal pleiotropy did not affect all analyses between exposure and outcome mentioned above (Table 4). Yet, some heterogeneity was found for antithrombotic agents versus DLB, salicylic acid and derivatives versus DLB, diuretics versus vascular dementia, and calcium channel blockers versus vascular dementia (Table 4). After correction, that is, elimination of some SNPs, no heterogeneity was found for antithrombotic agents versus DLB and calcium channel blockers versus vascular dementia (Table 4). MR‐PRESSO data further suggested that these data are robust (Table 5). Moreover, antihistamines for systemic use showed some protective effect on cognitive performance (*p* < 0.05, Table 3). The MR‐Egger regression indicated that results were unaffected by horizontal pleiotropy. Moreover, no heterogeneity was detected (Table 4).

**TABLE 3:** A causal association between drug use and cognitive impairment.

outcome	exposure	No. SNP	Methods	OR_CI	*p* value
Vascular dementia	Drugs for peptic ulcer and gastro‐esophageal reflux disease (GORD)	5	Inverse‐variance weighted	1.55 (0.61–3.91)	0.355
Vascular dementia	Drugs for peptic ulcer and gastro‐esophageal reflux disease (GORD)	5	MR Egger	571.97 (7.81–41,877.12)	0.0626
Vascular dementia	Drugs for peptic ulcer and gastro‐esophageal reflux disease (GORD)	5	Weighted median	1.79 (0.8–4)	0.156
Vascular dementia	Drugs for peptic ulcer and gastro‐esophageal reflux disease (GORD)	5	Weighted mode	1.77 (0.65–4.81)	0.324
Dementia due to Parkinson's’ disease	Drugs for peptic ulcer and gastro‐esophageal reflux disease (GORD)	5	Inverse‐variance weighted	0.72 (0.14–3.63)	0.694
Dementia due to Parkinson's’ disease	Drugs for peptic ulcer and gastro‐esophageal reflux disease (GORD)	5	MR Egger	542.6 (0–62,925,849.63)	0.368
Dementia due to Parkinson's disease	Drugs for peptic ulcer and gastro‐esophageal reflux disease (GORD)	5	Weighted median	1.05 (0.2–5.63)	0.952
Dementia due to Parkinson's disease	Drugs for peptic ulcer and gastro‐esophageal reflux disease (GORD)	5	Weighted mode	1.59 (0.14–17.42)	0.724
Dementia with Lewy bodies	Drugs for peptic ulcer and gastro‐esophageal reflux disease (GORD)	5	Inverse‐variance weighted	0.53 (0.23–1.22)	0.135
Dementia with Lewy bodies	Drugs for peptic ulcer and gastro‐esophageal reflux disease (GORD)	5	MR Egger	0.57 (0–468.47)	0.879
Dementia with Lewy bodies	Drugs for peptic ulcer and gastro‐esophageal reflux disease (GORD)	5	Weighted median	0.55 (0.2–1.45)	0.226
Dementia with Lewy bodies	Drugs for peptic ulcer and gastro‐esophageal reflux disease (GORD)	5	Weighted mode	1.04 (0.2–5.45)	0.965
Frontotemporal dementia	Drugs for peptic ulcer and gastro‐esophageal reflux disease (GORD)	5	Inverse‐variance weighted	0.95 (0.75–1.2)	0.666
Frontotemporal dementia	Drugs for peptic ulcer and gastro‐esophageal reflux disease (GORD)	5	MR Egger	0.77 (0.14–4.38)	0.788
Frontotemporal dementia	Drugs for peptic ulcer and gastro‐esophageal reflux disease (GORD)	5	Weighted median	0.92 (0.69–1.24)	0.601
Frontotemporal dementia	Drugs for peptic ulcer and gastro‐esophageal reflux disease (GORD)	5	Weighted mode	0.87 (0.59–1.28)	0.517
Dementia in Alzheimer's disease	Drugs for peptic ulcer and gastro‐esophageal reflux disease (GORD)	5	Inverse‐variance weighted	1.14 (0.71–1.86)	0.584
Dementia in Alzheimer's disease	Drugs for peptic ulcer and gastro‐esophageal reflux disease (GORD)	5	MR Egger	12.16 (0.5–292.85)	0.221
Dementia in Alzheimer's disease	Drugs for peptic ulcer and gastro‐esophageal reflux disease (GORD)	5	Weighted median	1 (0.58–1.7)	0.994
Dementia in Alzheimer's disease	Drugs for peptic ulcer and gastro‐esophageal reflux disease (GORD)	5	Weighted mode	0.87 (0.42–1.82)	0.731
Cognitive performance	Drugs for peptic ulcer and gastro‐esophageal reflux disease (GORD)	5	Inverse‐variance weighted	0.89 (0.73–1.07)	0.207
Cognitive performance	Drugs for peptic ulcer and gastro‐esophageal reflux disease (GORD)	5	MR Egger	0.33 (0.15–0.72)	0.0699
Cognitive performance	Drugs for peptic ulcer and gastro‐esophageal reflux disease (GORD)	5	Weighted median	0.9 (0.81–1)	0.0429
Cognitive performance	Drugs for peptic ulcer and gastro‐esophageal reflux disease (GORD)	5	Weighted mode	0.86 (0.69–1.06)	0.234
Vascular dementia	Drugs used in diabetes	56	Inverse‐variance weighted	1.06 (0.97–1.16)	0.182
Vascular dementia	Drugs used in diabetes	56	MR Egger	1.04 (0.84–1.29)	0.711
Vascular dementia	Drugs used in diabetes	56	Weighted median	1.12 (0.99–1.27)	0.0809
Vascular dementia	Drugs used in diabetes	56	Weighted mode	1.21 (0.99–1.49)	0.0661
Dementia due to Parkinson's disease	Drugs used in diabetes	56	Inverse‐variance weighted	0.87 (0.73–1.04)	0.128
Dementia due to Parkinson's disease	Drugs used in diabetes	56	MR Egger	0.9 (0.58–1.38)	0.628
Dementia due to Parkinson's disease	Drugs used in diabetes	56	Weighted median	0.97 (0.73–1.28)	0.823
Dementia due to Parkinson's disease	Drugs used in diabetes	56	Weighted mode	1.03 (0.66–1.62)	0.888
Dementia with Lewy bodies	Drugs used in diabetes	53	Inverse‐variance weighted	0.97 (0.87–1.1)	0.66
Dementia with Lewy bodies	Drugs used in diabetes	53	MR Egger	0.8 (0.61–1.05)	0.117
Dementia with Lewy bodies	Drugs used in diabetes	53	Weighted median	0.86 (0.72–1.03)	0.107
Dementia with Lewy bodies	Drugs used in diabetes	53	Weighted mode	0.87 (0.71–1.07)	0.198
Frontotemporal dementia	Drugs used in diabetes	56	Inverse‐variance weighted	1 (0.96–1.04)	0.946
Frontotemporal dementia	Drugs used in diabetes	56	MR Egger	0.98 (0.89–1.07)	0.587
Frontotemporal dementia	Drugs used in diabetes	56	Weighted median	1.03 (0.97–1.08)	0.333
Frontotemporal dementia	Drugs used in diabetes	56	Weighted mode	1.05 (0.93–1.18)	0.446
Dementia in Alzheimer's disease	Drugs used in diabetes	56	Inverse‐variance weighted	1.03 (0.96–1.11)	0.346
Dementia in Alzheimer's disease	Drugs used in diabetes	56	MR Egger	0.98 (0.83–1.15)	0.768
Dementia in Alzheimer's disease	Drugs used in diabetes	56	Weighted median	1.09 (0.98–1.2)	0.111
Dementia in Alzheimer's disease	Drugs used in diabetes	56	Weighted mode	1.09 (0.95–1.25)	0.231
Dementia in Alzheimer disease	Drugs used in diabetes (after correction)	55	Inverse‐variance weighted	1.07 (1–1.14)	0.0374
Dementia in Alzheimer disease	Drugs used in diabetes (after correction)	55	MR Egger	1.1 (0.93–1.29)	0.2619
Dementia in Alzheimer disease	Drugs used in diabetes (after correction)	55	Weighted median	1.09 (0.99–1.2)	0.0944
Dementia in Alzheimer disease	Drugs used in diabetes (after correction)	55	Weighted mode	1.09 (0.97–1.24)	0.1659
Cognitive performance	Drugs used in diabetes	55	Inverse‐variance weighted	1.01 (1–1.03)	0.101
Cognitive performance	Drugs used in diabetes	55	MR Egger	1.01 (0.98–1.05)	0.57
Cognitive performance	Drugs used in diabetes	55	Weighted median	1 (0.99–1.02)	0.917
Cognitive performance	Drugs used in diabetes	55	Weighted mode	1 (0.98–1.02)	0.956
Vascular dementia	Antithrombotic agents	13	Inverse‐variance weighted	0.95 (0.56–1.6)	0.852
Vascular dementia	Antithrombotic agents	13	MR Egger	1.62 (0.33–8.06)	0.567
Vascular dementia	Antithrombotic agents	13	Weighted median	0.87 (0.56–1.36)	0.549
Vascular dementia	Antithrombotic agents	13	Weighted mode	0.96 (0.52–1.77)	0.906
Dementia due to Parkinson's disease	Antithrombotic agents	13	Inverse‐variance weighted	1.05 (0.53–2.07)	0.889
Dementia due to Parkinson's disease	Antithrombotic agents	13	MR Egger	1.3 (0.17–9.87)	0.806
Dementia due to Parkinson's disease	Antithrombotic agents	13	Weighted median	0.89 (0.34–2.35)	0.811
Dementia due to Parkinson's disease	Antithrombotic agents	13	Weighted mode	0.57 ((0.11–2.83)	0.504
Dementia with Lewy bodies	Antithrombotic agents	11	Inverse‐variance weighted	2.12 (1.03–4.34)	0.0412
Dementia with Lewy bodies	Antithrombotic agents	11	MR Egger	6.19 (0.81–47.14)	0.112
Dementia with Lewy bodies	Antithrombotic agents	11	Weighted median	2.09 (1.12–3.89)	0.021
Dementia with Lewy bodies	Antithrombotic agents	11	Weighted mode	2.07 (0.93–4.63)	0.106
Frontotemporal dementia	Antithrombotic agents	13	Inverse‐variance weighted	1.07 (0.93–1.22)	0.356
Frontotemporal dementia	Antithrombotic agents	13	MR Egger	1.08 (0.72–1.63)	0.704
Frontotemporal dementia	Antithrombotic agents	13	Weighted median	1.14 (0.95–1.38)	0.158
Frontotemporal dementia	Antithrombotic agents	13	Weighted mode	1.17 (0.86–1.58)	0.331
Dementia in Alzheimer's disease	Antithrombotic agents	13	Inverse‐variance weighted	1.22 (0.59–2.54)	0.589
Dementia in Alzheimer's disease	Antithrombotic agents	13	MR Egger	3.31 (0.36–30.1)	0.31
Dementia in Alzheimer's disease	Antithrombotic agents	13	Weighted median	0.87 (0.64–1.2)	0.413
Dementia in Alzheimer's disease	Antithrombotic agents	13	Weighted mode	0.8 (0.52–1.23)	0.335
Cognitive performance	Antithrombotic agents	13	Inverse‐variance weighted	0.97 (0.91–1.04)	0.399
Cognitive performance	Antithrombotic agents	13	MR Egger	1.1 (0.93–1.31)	0.288
Cognitive performance	Antithrombotic agents	13	Weighted median	0.99 (0.95–1.03)	0.559
Cognitive performance	Antithrombotic agents	13	Weighted mode	0.99 (0.93–1.05)	0.779
Vascular dementia	Vasodilators used in cardiac diseases	2	Inverse‐variance weighted	0.9 (0.63–1.28)	0.545
Dementia due to Parkinson's disease	Vasodilators used in cardiac diseases	2	Inverse‐variance weighted	1.71 (0.81–3.64)	0.162
Dementia with Lewy bodies	Vasodilators used in cardiac diseases	2	Inverse‐variance weighted	1.32 (0.9–1.93)	0.155
Frontotemporal dementia	Vasodilators used in cardiac diseases	2	Inverse‐variance weighted	1.12 (0.96–1.3)	0.154
Dementia in Alzheimer's disease	Vasodilators used in cardiac diseases	2	Inverse‐variance weighted	0.98 (0.68–1.41)	0.898
Cognitive performance	Vasodilators used in cardiac diseases	2	Inverse‐variance weighted	1.01 (0.98–1.03)	0.656
Vascular dementia	Antihypertensives	3	Inverse‐variance weighted	1.42 (0.93–2.18)	0.106
Vascular dementia	Antihypertensives	3	MR Egger	2,156,589.1 (1.84–2,533,058,915,517.78)	0.29
Vascular dementia	Antihypertensives	3	Weighted median	1.47 (0.96–2.23)	0.0746
Vascular dementia	Antihypertensives	3	Weighted mode	1.6 (0.93–2.75)	0.231
Dementia due to Parkinson's disease	Antihypertensives	3	Inverse‐variance weighted	1.31 (0.54–3.16)	0.554
Dementia due to Parkinson's disease	Antihypertensives	3	MR Egger	51,807,664.02 (0–8.75970990571968e + 28)	0.606
Dementia due to Parkinson's disease	Antihypertensives	3	Weighted median	1.32 (0.53–3.26)	0.554
Dementia due to Parkinson's disease	Antihypertensives	3	Weighted mode	1.43 (0.45–4.53)	0.609
Dementia with Lewy bodies	Antihypertensives	4	Inverse‐variance weighted	1.09 (0.66–1.81)	0.742
Dementia with Lewy bodies	Antihypertensives	4	MR Egger	0 (0–6.27)	0.239
Dementia with Lewy bodies	Antihypertensives	4	Weighted median	1.19 (0.77–1.85)	0.432
Dementia with Lewy bodies	Antihypertensives	4	Weighted mode	1.36 (0.66–2.83)	0.466
Frontotemporal dementia	Antihypertensives	3	Inverse‐variance weighted	1.13 (1–1.28)	0.0581
Frontotemporal dementia	Antihypertensives	3	MR Egger	0.05 (0–18.29)	0.5
Frontotemporal dementia	Antihypertensives	3	Weighted median	1.08 (0.92–1.26)	0.371
Frontotemporal dementia	Antihypertensives	3	Weighted mode	1.07 (0.89–1.29)	0.55
Dementia in Alzheimer's disease	Antihypertensives	3	Inverse‐variance weighted	0.83 (0.67–1.01)	0.0665
Dementia in Alzheimer's disease	Antihypertensives	3	MR Egger	0.76 (0–13,310.92)	0.965
Dementia in Alzheimer's disease	Antihypertensives	3	Weighted median	0.86 (0.67–1.11)	0.251
Dementia in Alzheimer's disease	Antihypertensives	3	Weighted mode	0.87 (0.65–1.18)	0.471
Cognitive performance	Antihypertensives	4	Inverse‐variance weighted	0.99 (0.96–1.02)	0.551
Cognitive performance	Antihypertensives	4	MR Egger	2.17 (0.97–4.85)	0.199
Cognitive performance	Antihypertensives	4	Weighted median	0.99 (0.96–1.02)	0.531
Cognitive performance	Antihypertensives	4	Weighted mode	0.98 (0.93–1.04)	0.572
Vascular dementia	Diuretics	93	Inverse‐variance weighted	1.13 (1–1.27)	0.0472
Vascular dementia	Diuretics	93	MR Egger	1.25 (0.86–1.8)	0.245
Vascular dementia	Diuretics	93	Weighted median	1.29 (1.1–1.5)	0.0013
Vascular dementia	Diuretics	93	Weighted mode	1.42 (1.07–1.88)	0.0156
Dementia due to Parkinson's disease	Diuretics	93	Inverse‐variance weighted	0.93 (0.75–1.16)	0.531
Dementia due to Parkinson's disease	Diuretics	93	MR Egger	1.23 (0.63–2.39)	0.542
Dementia due to Parkinson's disease	Diuretics	93	Weighted median	0.82 (0.59–1.13)	0.223
Dementia due to Parkinson's disease	Diuretics	93	Weighted mode	0.64 (0.32–1.27)	0.208
Dementia with Lewy bodies	Diuretics	89	Inverse‐variance weighted	1.04 (0.91–1.2)	0.575
Dementia with Lewy bodies	Diuretics	89	MR Egger	0.79 (0.49–1.26)	0.319
Dementia with Lewy bodies	Diuretics	89	Weighted median	1.14 (0.94–1.4)	0.189
Dementia with Lewy bodies	Diuretics	89	Weighted mode	1.34 (0.84–2.14)	0.228
Frontotemporal dementia	Diuretics	93	Inverse‐variance weighted	1.06 (1.02–1.11)	0.0044
Frontotemporal dementia	Diuretics	93	MR Egger	1.14 (1–1.29)	0.0548
Frontotemporal dementia	Diuretics	93	Weighted median	1.1 (1.03–1.17)	0.0024
Frontotemporal dementia	Diuretics	93	Weighted mode	1.16 (1.03–1.3)	0.0139
Dementia in Alzheimer's disease	Diuretics	93	Inverse‐variance weighted	0.92 (0.85–1)	0.0502
Dementia in Alzheimer's disease	Diuretics	93	MR Egger	0.98 (0.76–1.27)	0.898
Dementia in Alzheimer's disease	Diuretics	93	Weighted median	0.92 (0.82–1.02)	0.111
Dementia in Alzheimer's disease	Diuretics	93	Weighted mode	0.91 (0.75–1.11)	0.366
Cognitive performance	Diuretics	96	Inverse‐variance weighted	0.99 (0.97–1.01)	0.246
Cognitive performance	Diuretics	96	MR Egger	0.98 (0.93–1.03)	0.393
Cognitive performance	Diuretics	96	Weighted median	0.99 (0.98–1.01)	0.299
Cognitive performance	Diuretics	96	Weighted mode	0.99 (0.96–1.02)	0.607
Vascular dementia	Beta blocking agents	57	Inverse‐variance weighted	1.13 (0.99–1.3)	0.0715
Vascular dementia	Beta blocking agents	57	MR Egger	0.98 (0.59–1.65)	0.946
Vascular dementia	Beta blocking agents	57	Weighted median	1.17 (0.96–1.42)	0.12
Vascular dementia	Beta blocking agents	57	Weighted mode	1.12 (0.76–1.64)	0.577
Dementia due to Parkinson's disease	Beta blocking agents	57	Inverse‐variance weighted	1.01 (0.74–1.37)	0.969
Dementia due to Parkinson's disease	Beta blocking agents	57	MR Egger	0.51 (0.16–1.67)	0.275
Dementia due to Parkinson's disease	Beta blocking agents	57	Weighted median	0.79 (0.51–1.2)	0.264
Dementia due to Parkinson's disease	Beta blocking agents	57	Weighted mode	0.72 (0.3–1.72)	0.462
Dementia with Lewy bodies	Beta blocking agents	57	Inverse‐variance weighted	0.96 (0.77–1.2)	0.742
Dementia with Lewy bodies	Beta blocking agents	57	MR Egger	0.73 (0.32–1.63)	0.441
Dementia with Lewy bodies	Beta blocking agents	57	Weighted median	1.05 (0.8–1.4)	0.712
Dementia with Lewy bodies	Beta blocking agents	57	Weighted mode	1.48 (0.73–3)	0.277
Frontotemporal dementia	Beta blocking agents	57	Inverse‐variance weighted	1.06 (0.99–1.13)	0.0916
Frontotemporal dementia	Beta blocking agents	57	MR Egger	1.21 (0.93–1.56)	0.159
Frontotemporal dementia	Beta blocking agents	57	Weighted median	1.16 (1.06–1.26)	0.0012
Frontotemporal dementia	Beta blocking agents	57	Weighted mode	1.21 (1.03–1.42)	0.0214
Dementia in Alzheimer's disease	Beta blocking agents	57	Inverse‐variance weighted	0.97 (0.87–1.08)	0.561
Dementia in Alzheimer's disease	Beta blocking agents	57	MR Egger	0.94 (0.62–1.43)	0.782
Dementia in Alzheimer's disease	Beta blocking agents	57	Weighted median	0.96 (0.84–1.11)	0.595
Dementia in Alzheimer's disease	Beta blocking agents	57	Weighted mode	0.92 (0.68–1.26)	0.624
Cognitive performance	Beta blocking agents	57	Inverse‐variance weighted	1 (0.98–1.03)	0.868
Cognitive performance	Beta blocking agents	57	MR Egger	0.99 (0.91–1.08)	0.816
Cognitive performance	Beta blocking agents	57	Weighted median	1 (0.98–1.02)	0.85
Cognitive performance	Beta blocking agents	57	Weighted mode	1.01 (0.96–1.05)	0.784
Vascular dementia	Calcium channel blockers	99	Inverse‐variance weighted	1.16 (1.04–1.29)	0.0071
Vascular dementia	Calcium channel blockers	99	MR Egger	1.01 (0.71–1.43)	0.959
Vascular dementia	Calcium channel blockers	99	Weighted median	1.2 (1.04–1.39)	0.015
Vascular dementia	Calcium channel blockers	99	Weighted mode	1.32 (0.99–1.75)	0.0576
Dementia due to Parkinson's disease	Calcium channel blockers	99	Inverse‐variance weighted	1.14 (0.91–1.42)	0.269
Dementia due to Parkinson's disease	Calcium channel blockers	99	MR Egger	0.85 (0.42–1.75)	0.663
Dementia due to Parkinson's disease	Calcium channel blockers	99	Weighted median	1.14 (0.84–1.55)	0.403
Dementia due to Parkinson's disease	Calcium channel blockers	99	Weighted mode	0.94 (0.5–1.77)	0.856
Dementia with Lewy bodies	Calcium channel blockers	97	Inverse‐variance weighted	1 (0.86–1.17)	0.982
Dementia with Lewy bodies	Calcium channel blockers	97	MR Egger	0.94 (0.56–1.59)	0.821
Dementia with Lewy bodies	Calcium channel blockers	97	Weighted median	1.08 (0.88–1.32)	0.458
Dementia with Lewy bodies	Calcium channel blockers	97	Weighted mode	1.27 (0.8–1.99)	0.313
Frontotemporal dementia	Calcium channel blockers	99	Inverse‐variance weighted	1.04 (0.99–1.08)	0.108
Frontotemporal dementia	Calcium channel blockers	99	MR Egger	1.04 (0.9–1.2)	0.571
Frontotemporal dementia	Calcium channel blockers	99	Weighted median	1.03 (0.97–1.1)	0.292
Frontotemporal dementia	Calcium channel blockers	99	Weighted mode	1.16 (1.02–1.31)	0.0258
Dementia in Alzheimer's disease	Calcium channel blockers	99	Inverse‐variance weighted	0.99 (0.9–1.1)	0.916
Dementia in Alzheimer's disease	Calcium channel blockers	99	MR Egger	1.07 (0.78–1.47)	0.66
Dementia in Alzheimer's disease	Calcium channel blockers	99	Weighted median	0.97 (0.88–1.07)	0.517
Dementia in Alzheimer's disease	Calcium channel blockers	99	Weighted mode	0.92 (0.74–1.13)	0.416
Cognitive performance	Calcium channel blockers	98	Inverse‐variance weighted	0.99 (0.97–1.01)	0.197
Cognitive performance	Calcium channel blockers	98	MR Egger	0.99 (0.94–1.04)	0.759
Cognitive performance	Calcium channel blockers	98	Weighted median	0.99 (0.98–1.01)	0.466
Cognitive performance	Calcium channel blockers	98	Weighted mode	1 (0.97–1.02)	0.833
Vascular dementia	Agents acting on the renin–angiotensin system	176	Inverse‐variance weighted	1.11 (1–1.23)	0.0571
Vascular dementia	Agents acting on the renin–angiotensin system	176	MR Egger	1.06 (0.76–1.48)	0.721
Vascular dementia	Agents acting on the renin–angiotensin system	176	Weighted median	1.22 (1.05–1.41)	0.0103
Vascular dementia	Agents acting on the renin–angiotensin system	176	Weighted mode	1.39 (1–1.94)	0.0531
Dementia due to Parkinson's disease	Agents acting on the renin–angiotensin system	176	Inverse‐variance weighted	1.11 (0.88–1.4)	0.385
Dementia due to Parkinson's disease	Agents acting on the renin–angiotensin system	176	MR Egger	1.22 (0.6–2.51)	0.585
Dementia due to Parkinson's disease	Agents acting on the renin–angiotensin system	176	Weighted median	1.06 (0.76–1.49)	0.732
Dementia due to Parkinson's disease	Agents acting on the renin–angiotensin system	176	Weighted mode	0.74 (0.36–1.53)	0.416
Dementia with Lewy bodies	Agents acting on the renin–angiotensin system	174	Inverse‐variance weighted	1.04 (0.91–1.2)	0.55
Dementia with Lewy bodies	Agents acting on the renin–angiotensin system	174	MR Egger	0.9 (0.58–1.4)	0.634
Dementia with Lewy bodies	Agents acting on the renin–angiotensin system	174	Weighted median	1.09 (0.9–1.31)	0.4
Dementia with Lewy bodies	Agents acting on the renin–angiotensin system	174	Weighted mode	1.09 (0.63–1.87)	0.768
Frontotemporal dementia	Agents acting on the renin–angiotensin system	176	Inverse‐variance weighted	1.03 (0.99–1.08)	0.147
Frontotemporal dementia	Agents acting on the renin–angiotensin system	176	MR Egger	1.08 (0.94–1.24)	0.287
Frontotemporal dementia	Agents acting on the renin–angiotensin system	176	Weighted median	1.08 (1.01–1.15)	0.0169
Frontotemporal dementia	Agents acting on the renin–angiotensin system	176	Weighted mode	1.18 (1.04–1.34)	0.0093
Dementia in Alzheimer's disease	Agents acting on the renin–angiotensin system	176	Inverse‐variance weighted	0.96 (0.87–1.05)	0.36
Dementia in Alzheimer's disease	Agents acting on the renin–angiotensin system	176	MR Egger	1 (0.74–1.33)	0.974
Dementia in Alzheimer's disease	Agents acting on the renin–angiotensin system	176	Weighted median	0.92 (0.83–1.03)	0.154
Dementia in Alzheimer's disease	Agents acting on the renin–angiotensin system	176	Weighted mode	0.9 (0.71–1.15)	0.403
Cognitive performance	Agents acting on the renin–angiotensin system	179	Inverse‐variance weighted	1 (0.98–1.02)	0.949
Cognitive performance	Agents acting on the renin–angiotensin system	179	MR Egger	1.02 (0.97–1.08)	0.417
Cognitive performance	Agents acting on the renin–angiotensin system	179	Weighted median	0.99 (0.98–1.01)	0.274
Cognitive performance	Agents acting on the renin–angiotensin system	179	Weighted mode	1 (0.96–1.03)	0.806
Vascular dementia	HMG CoA reductase inhibitors	94	Inverse‐variance weighted	1.14 (0.96–1.35)	0.125
Vascular dementia	HMG CoA reductase inhibitors	94	MR Egger	1.19 (0.88–1.6)	0.26
Vascular dementia	HMG CoA reductase inhibitors	94	Weighted median	0.91 (0.76–1.1)	0.331
Vascular dementia	HMG CoA reductase inhibitors	94	Weighted mode	0.85 (0.68–1.05)	0.141
Dementia due to Parkinson's disease	HMG CoA reductase inhibitors	94	Inverse‐variance weighted	1.03 (0.82–1.3)	0.784
Dementia due to Parkinson's disease	HMG CoA reductase inhibitors	94	MR Egger	1.59 (1.06–2.39)	0.0265
Dementia due to Parkinson's disease	HMG CoA reductase inhibitors	94	Weighted median	1.25 (0.88–1.78)	0.216
Dementia due to Parkinson's disease	HMG CoA reductase inhibitors	94	Weighted mode	1.23 (0.81–1.89)	0.337
Dementia with Lewy bodies	HMG CoA reductase inhibitors	92	Inverse‐variance weighted	1.36 (1.07–1.72)	0.0114
Dementia with Lewy bodies	HMG CoA reductase inhibitors	92	MR Egger	2.22 (1.47–3.36)	0.0003
Dementia with Lewy bodies	HMG CoA reductase inhibitors	92	Weighted median	1.27 (1–1.62)	0.0492
Dementia with Lewy bodies	HMG CoA reductase inhibitors	92	Weighted mode	1.29 (0.89–1.88)	0.187
Frontotemporal dementia	HMG CoA reductase inhibitors	94	Inverse‐variance weighted	1.01 (0.96–1.06)	0.682
Frontotemporal dementia	HMG CoA reductase inhibitors	94	MR Egger	1 (0.92–1.09)	0.965
Frontotemporal dementia	HMG CoA reductase inhibitors	94	Weighted median	1 (0.93–1.08)	0.912
Frontotemporal dementia	HMG CoA reductase inhibitors	94	Weighted mode	1 (0.91–1.1)	0.948
Dementia in Alzheimer's disease	HMG CoA reductase inhibitors	94	Inverse‐variance weighted	1.32 (1.05–1.65)	0.0176
Dementia in Alzheimer's disease	HMG CoA reductase inhibitors	94	MR Egger	1.81 (1.22–2.69)	0.0043
Dementia in Alzheimer's disease	HMG CoA reductase inhibitors	94	Weighted median	1.11 (0.98–1.26)	0.113
Dementia in Alzheimer's disease	HMG CoA reductase inhibitors	94	Weighted mode	1.07 (0.9–1.27)	0.423
Cognitive performance	HMG CoA reductase inhibitors	92	Inverse‐variance weighted	1 (0.98–1.02)	0.893
Cognitive performance	HMG CoA reductase inhibitors	92	MR Egger	1 (0.97–1.03)	0.922
Cognitive performance	HMG CoA reductase inhibitors	92	Weighted median	1 (0.98–1.02)	0.997
Cognitive performance	HMG CoA reductase inhibitors	92	Weighted mode	1 (0.98–1.02)	0.78
Vascular dementia	Thyroid preparations	128	Inverse‐variance weighted	0.96 (0.91–1.02)	0.232
Vascular dementia	Thyroid preparations	128	MR Egger	0.93 (0.82–1.06)	0.309
Vascular dementia	Thyroid preparations	128	Weighted median	0.97 (0.88–1.07)	0.54
Vascular dementia	Thyroid preparations	128	Weighted mode	0.99 (0.87–1.11)	0.821
Dementia due to Parkinson's disease	Thyroid preparations	128	Inverse‐variance weighted	0.98 (0.86–1.13)	0.796
Dementia due to Parkinson's disease	Thyroid preparations	128	MR Egger	1.15 (0.86–1.54)	0.351
Dementia due to Parkinson's disease	Thyroid preparations	128	Weighted median	0.99 (0.8–1.22)	0.908
Dementia due to Parkinson's disease	Thyroid preparations	128	Weighted mode	1.05 (0.81–1.36)	0.708
Dementia with Lewy bodies	Thyroid preparations	126	Inverse‐variance weighted	0.99 (0.9–1.08)	0.794
Dementia with Lewy bodies	Thyroid preparations	126	MR Egger	0.92 (0.74–1.14)	0.447
Dementia with Lewy bodies	Thyroid preparations	126	Weighted median	0.91 (0.79–1.04)	0.158
Dementia with Lewy bodies	Thyroid preparations	126	Weighted mode	0.86 (0.73–1.01)	0.0625
Frontotemporal dementia	Thyroid preparations	128	Inverse‐variance weighted	1.03 (1.01–1.06)	0.0158
Frontotemporal dementia	Thyroid preparations	128	MR Egger	0.99 (0.94–1.05)	0.732
Frontotemporal dementia	Thyroid preparations	128	Weighted median	1.04 (1–1.09)	0.0492
Frontotemporal dementia	Thyroid preparations	128	Weighted mode	1.05 (0.99–1.12)	0.0822
Dementia in Alzheimer's disease	Thyroid preparations	128	Inverse‐variance weighted	1.02 (0.97–1.07)	0.484
Dementia in Alzheimer's disease	Thyroid preparations	128	MR Egger	1.02 (0.92–1.13)	0.77
Dementia in Alzheimer's disease	Thyroid preparations	128	Weighted median	1.04 (0.97–1.12)	0.24
Dementia in Alzheimer's disease	Thyroid preparations	128	Weighted mode	1.04 (0.96–1.13)	0.285
Cognitive performance	Thyroid preparations	129	Inverse‐variance weighted	1 (0.99–1.01)	0.541
Cognitive performance	Thyroid preparations	129	MR Egger	1 (0.98–1.02)	0.974
Cognitive performance	Thyroid preparations	129	Weighted median	1.01 (0.99–1.02)	0.273
Cognitive performance	Thyroid preparations	129	Weighted mode	1.01 (0.99–1.02)	0.245
Vascular dementia	Immunosuppressants	2	Inverse‐variance weighted	0.89 (0.79–1.01)	0.0644
Dementia due to Parkinson's disease	Immunosuppressants	2	Inverse‐variance weighted	0.87 (0.38–1.97)	0.741
Dementia with Lewy bodies	Immunosuppressants	1	Wald ratio	0.85 (0.72–1.02)	0.0749
Frontotemporal dementia	Immunosuppressants	2	Inverse‐variance weighted	1.07 (1.01–1.12)	0.0125
Dementia in Alzheimer disease	Immunosuppressants	2	Inverse‐variance weighted	0.96 (0.85–1.08)	0.507
Cognitive performance	Immunosuppressants	2	Inverse‐variance weighted	0.98 (0.97–0.99)	0.0016
Vascular dementia	Antiinflammatroy and antirheumatic products nonsteroids	6	Inverse‐variance weighted	1.03 (0.46–2.29)	0.943
Vascular dementia	Antiinflammatroy and antirheumatic products nonsteroids	6	MR Egger	0.22 (0–95.7)	0.651
Vascular dementia	Antiinflammatroy and antirheumatic products nonsteroids	6	Weighted median	0.84 (0.39–1.81)	0.649
Vascular dementia	Antiinflammatroy and antirheumatic products nonsteroids	6	Weighted mode	0.67 (0.25–1.83)	0.474
Dementia due to Parkinson's disease	Antiinflammatroy and antirheumatic products nonsteroids	6	Inverse‐variance weighted	0.74 (0.18–3.04)	0.674
Dementia due to Parkinson's disease	Antiinflammatroy and antirheumatic products nonsteroids	6	MR Egger	0 (0–2.63)	0.158
Dementia due to Parkinson's disease	Antiinflammatroy and antirheumatic products nonsteroids	6	Weighted median	0.85 (0.18–4.05)	0.838
Dementia due to Parkinson's disease	Antiinflammatroy and antirheumatic products nonsteroids	6	Weighted mode	0.19 (0.02–2.19)	0.243
Dementia with Lewy bodies	Antiinflammatroy and antirheumatic products nonsteroids	6	Inverse‐variance weighted	0.92 (0.36–2.33)	0.858
Dementia with Lewy bodies	Antiinflammatroy and antirheumatic products nonsteroids	6	MR Egger	0.01 (0–1.45)	0.142
Dementia with Lewy bodies	Antiinflammatroy and antirheumatic products nonsteroids	6	Weighted median	0.69 (0.25–1.9)	0.468
Dementia with Lewy bodies	Antiinflammatroy and antirheumatic products nonsteroids	6	Weighted mode	0.44 (0.11–1.74)	0.294
Frontotemporal dementia	Antiinflammatroy and antirheumatic products nonsteroids	6	Inverse‐variance weighted	1.19 (0.83–1.72)	0.347
Frontotemporal dementia	Antiinflammatroy and antirheumatic products nonsteroids	6	MR Egger	1.83 (0.11–30.96)	0.696
Frontotemporal dementia	Antiinflammatroy and antirheumatic products nonsteroids	6	Weighted median	1.14 (0.81–1.61)	0.439
Frontotemporal dementia	Antiinflammatroy and antirheumatic products nonsteroids	6	Weighted mode	1.13 (0.73–1.77)	0.606
Dementia in Alzheimer's disease	Antiinflammatroy and antirheumatic products nonsteroids	6	Inverse‐variance weighted	1.38 (0.69–2.76)	0.357
Dementia in Alzheimer's disease	Antiinflammatroy and antirheumatic products nonsteroids	6	MR Egger	3.67 (0.02–744.71)	0.657
Dementia in Alzheimer's disease	Antiinflammatroy and antirheumatic products nonsteroids	6	Weighted median	1.16 (0.69–1.96)	0.572
Dementia in Alzheimer's disease	Antiinflammatroy and antirheumatic products nonsteroids	6	Weighted mode	1.07 (0.55–2.1)	0.851
Cognitive performance	Antiinflammatroy and antirheumatic products nonsteroids	5	Inverse‐variance weighted	0.97 (0.85–1.11)	0.654
Cognitive performance	Antiinflammatroy and antirheumatic products nonsteroids	5	MR Egger	1.27 (0.47–3.39)	0.67
Cognitive performance	Antiinflammatroy and antirheumatic products nonsteroids	5	Weighted median	1.01 (0.93–1.1)	0.8
Cognitive performance	Antiinflammatroy and antirheumatic products nonsteroids	5	Weighted mode	1.05 (0.96–1.16)	0.332
Vascular dementia	Drugs affecting bone structure and mineralization	11	Inverse‐variance weighted	0.88 (0.74–1.06)	0.19
Vascular dementia	Drugs affecting bone structure and mineralization	11	MR Egger	1.62 (0.66–3.95)	0.317
Vascular dementia	Drugs affecting bone structure and mineralization	11	Weighted median	0.83 (0.65–1.07)	0.152
Vascular dementia	Drugs affecting bone structure and mineralization	11	Weighted mode	0.82 (0.58–1.15)	0.27
Dementia due to Parkinson's disease	Drugs affecting bone structure and mineralization	11	Inverse‐variance weighted	0.88 (0.53–1.44)	0.606
Dementia due to Parkinson's disease	Drugs affecting bone structure and mineralization	11	MR Egger	0.53 (0.04–6.69)	0.636
Dementia due to Parkinson's disease	Drugs affecting bone structure and mineralization	11	Weighted median	0.87 (0.49–1.55)	0.636
Dementia due to Parkinson's disease	Drugs affecting bone structure and mineralization	11	Weighted mode	0.77 (0.3–1.96)	0.595
Dementia due to Parkinson's disease	Drugs affecting bone structure and mineralization	10	Inverse‐variance weighted	0.96 (0.73–1.25)	0.74
Dementia with Lewy bodies	Drugs affecting bone structure and mineralization	10	MR Egger	0.57 (0.17–1.96)	0.401
Dementia with Lewy bodies	Drugs affecting bone structure and mineralization	10	Weighted median	1.08 (0.78–1.51)	0.634
Dementia with Lewy bodies	Drugs affecting bone structure and mineralization	10	Weighted mode	1.12 (0.71–1.77)	0.633
Frontotemporal dementia	Drugs affecting bone structure and mineralization	11	Inverse‐variance weighted	1.03 (0.96–1.12)	0.406
Frontotemporal dementia	Drugs affecting bone structure and mineralization	11	MR Egger	0.65 (0.45–0.96)	0.0575
Frontotemporal dementia	Drugs affecting bone structure and mineralization	11	Weighted median	1.06 (0.95–1.17)	0.281
Frontotemporal dementia	Drugs affecting bone structure and mineralization	11	Weighted mode	1.08 (0.93–1.25)	0.352
Dementia in Alzheimer's disease	Drugs affecting bone structure and mineralization	11	Inverse‐variance weighted	0.93 (0.81–1.05)	0.241
Dementia in Alzheimer's disease	Drugs affecting bone structure and mineralization	11	MR Egger	0.71 (0.38–1.33)	0.311
Dementia in Alzheimer's disease	Drugs affecting bone structure and mineralization	11	Weighted median	0.89 (0.75–1.05)	0.174
Dementia in Alzheimer's disease	Drugs affecting bone structure and mineralization	11	Weighted mode	0.86 (0.67–1.1)	0.264
Cognitive performance	Drugs affecting bone structure and mineralization	10	Inverse‐variance weighted	1 (0.97–1.02)	0.95
Cognitive performance	Drugs affecting bone structure and mineralization	10	MR Egger	1.07 (0.93–1.23)	0.366
Cognitive performance	Drugs affecting bone structure and mineralization	10	Weighted median	1 (0.98–1.03)	0.835
Cognitive performance	Drugs affecting bone structure and mineralization	10	Weighted mode	1 (0.96–1.05)	0.972
Vascular dementia	Opioids	2	Inverse‐variance weighted	1.22 (0.45–3.33)	0.699
Dementia due to Parkinson's disease	Opioids	2	Inverse‐variance weighted	2.64 (0.68–10.32)	0.163
Dementia with Lewy bodies	Opioids	3	Inverse‐variance weighted	1.17 (0.35–3.86)	0.801
Dementia with Lewy bodies	Opioids	3	MR Egger	21.03 (0–6,698,830,718,703.03)	0.859
Dementia with Lewy bodies	Opioids	3	Weighted median	0.96 (0.35–2.62)	0.941
Dementia with Lewy bodies	Opioids	3	Weighted mode	0.77 (0.22–2.67)	0.718
Frontotemporal dementia	Opioids	2	Inverse‐variance weighted	0.94 (0.72–1.23)	0.65
Dementia in Alzheimer disease	Opioids	2	Inverse‐variance weighted	1.36 (0.86–2.16)	0.194
Cognitive performance	Opioids	3	Inverse‐variance weighted	0.83 (0.63–1.1)	0.197
Cognitive performance	Opioids	3	MR Egger	4.83 (0.03–903.29)	0.661
Cognitive performance	Opioids	3	Weighted median	0.93 (0.86–1.01)	0.0754
Cognitive performance	Opioids	3	Weighted mode	0.97 (0.91–1.04)	0.451
Vascular dementia	Salicylic acid and derivatives	10	Inverse‐variance weighted	1.04 (0.62–1.74)	0.88
Vascular dementia	Salicylic acid and derivatives	10	MR Egger	1.27 (0.24–6.62)	0.783
Vascular dementia	Salicylic acid and derivatives	10	Weighted median	0.94 (0.58–1.51)	0.784
Vascular dementia	Salicylic acid and derivatives	10	Weighted mode	1.07 (0.55–2.11)	0.842
Dementia due to Parkinson's disease	Salicylic acid and derivatives	10	Inverse‐variance weighted	1.11 (0.54–2.28)	0.783
Dementia due to Parkinson's disease	Salicylic acid and derivatives	10	MR Egger	5.57 (0.63–49.24)	0.161
Dementia due to Parkinson's disease	Salicylic acid and derivatives	10	Weighted median	1.02 (0.39–2.64)	0.972
Dementia due to Parkinson's disease	Salicylic acid and derivatives	10	Weighted mode	0.59 (0.13–2.63)	0.506
Dementia with Lewy bodies	Salicylic acid and derivatives	9	Inverse‐variance weighted	2.77 (1.44–5.32)	0.0022
Dementia with Lewy bodies	Salicylic acid and derivatives	9	MR Egger	2.32 (0.36–14.8)	0.402
Dementia with Lewy bodies	Salicylic acid and derivatives	9	Weighted median	2.49 (1.27–4.91)	0.0082
Dementia with Lewy bodies	Salicylic acid and derivatives	9	Weighted mode	2.1 (0.9–4.92)	0.126
Frontotemporal dementia	Salicylic acid and derivatives	10	Inverse‐variance weighted	1.13 (0.98–1.31)	0.0968
Frontotemporal dementia	Salicylic acid and derivatives	10	MR Egger	0.98 (0.64–1.52)	0.947
Frontotemporal dementia	Salicylic acid and derivatives	10	Weighted median	1.16 (0.97–1.4)	0.113
Frontotemporal dementia	Salicylic acid and derivatives	10	Weighted mode	1.17 (0.88–1.54)	0.301
Dementia in Alzheimer's disease	Salicylic acid and derivatives	10	Inverse‐variance weighted	1.4 (0.61–3.22)	0.426
Dementia in Alzheimer's disease	Salicylic acid and derivatives	10	MR Egger	4.43 (0.34–57.14)	0.286
Dementia in Alzheimer's disease	Salicylic acid and derivatives	10	Weighted median	0.92 (0.64–1.33)	0.663
Dementia in Alzheimer's disease	Salicylic acid and derivatives	10	Weighted mode	0.79 (0.5–1.23)	0.32
Cognitive performance	Salicylic acid and derivatives	10	Inverse‐variance weighted	0.97 (0.92–1.03)	0.356
Cognitive performance	Salicylic acid and derivatives	10	MR Egger	1.1 (0.95–1.27)	0.259
Cognitive performance	Salicylic acid and derivatives	10	Weighted median	1 (0.95–1.04)	0.853
Cognitive performance	Salicylic acid and derivatives	10	Weighted mode	1 (0.94–1.05)	0.863
Vascular dementia	Anilides	7	Inverse‐variance weighted	0.83 (0.3–2.33)	0.729
Vascular dementia	Anilides	7	MR Egger	0.65 (0–826.7)	0.91
Vascular dementia	Anilides	7	Weighted median	1.22 (0.57–2.62)	0.605
Vascular dementia	Anilides	7	Weighted mode	1.66 (0.49–5.66)	0.446
Dementia due to Parkinson's disease	Anilides	7	Inverse‐variance weighted	0.44 (0.15–1.27)	0.128
Dementia due to Parkinson's disease	Anilides	7	MR Egger	16.22 (0.02–14,456.25)	0.458
Dementia due to Parkinson's disease	Anilides	7	Weighted median	0.28 (0.07–1.15)	0.0765
Dementia due to Parkinson's disease	Anilides	7	Weighted mode	0.2 (0.02–2.26)	0.241
Dementia with Lewy bodies	Anilides	7	Inverse‐variance weighted	0.67 (0.31–1.45)	0.311
Dementia with Lewy bodies	Anilides	7	MR Egger	0.01 (0–0.38)	0.059
Dementia with Lewy bodies	Anilides	7	Weighted median	0.66 (0.28–1.56)	0.346
Dementia with Lewy bodies	Anilides	7	Weighted mode	0.56 (0.16–1.92)	0.388
Frontotemporal dementia	Anilides	7	Inverse‐variance weighted	1.12 (0.91–1.39)	0.28
Frontotemporal dementia	Anilides	7	MR Egger	0.75 (0.19–2.89)	0.694
Frontotemporal dementia	Anilides	7	Weighted median	1.2 (0.93–1.54)	0.166
Frontotemporal dementia	Anilides	7	Weighted mode	1.24 (0.92–1.67)	0.21
Dementia in Alzheimer's disease	Anilides	7	Inverse‐variance weighted	0.76 (0.41–1.42)	0.391
Dementia in Alzheimer's disease	Anilides	7	MR Egger	3.66 (0.06–222.98)	0.563
Dementia in Alzheimer's disease	Anilides	7	Weighted median	1.11 (0.69–1.77)	0.679
Dementia in Alzheimer's disease	Anilides	7	Weighted mode	1.11 (0.7–1.75)	0.678
Cognitive performance	Anilides	6	Inverse‐variance weighted	0.92 (0.77–1.1)	0.364
Cognitive performance	Anilides	6	MR Egger	0.84 (0.24–2.86)	0.789
Cognitive performance	Anilides	6	Weighted median	1.09 (0.99–1.19)	0.0871
Cognitive performance	Anilides	6	Weighted mode	1.09 (1–1.19)	0.105
Vascular dementia	Antimigraine preparations	13	Inverse‐variance weighted	0.93 (0.82–1.04)	0.197
Vascular dementia	Antimigraine preparations	13	MR Egger	2.69 (1.23–5.87)	0.0308
Vascular dementia	Antimigraine preparations	13	Weighted median	0.91 (0.77–1.07)	0.234
Vascular dementia	Antimigraine preparations	13	Weighted mode	0.88 (0.66–1.19)	0.431
Dementia due to Parkinson's disease	Antimigraine preparations	13	Inverse‐variance weighted	0.97 (0.75–1.25)	0.802
Dementia due to Parkinson's disease	Antimigraine preparations	13	MR Egger	0.56 (0.1–3.26)	0.536
Dementia due to Parkinson's disease	Antimigraine preparations	13	Weighted median	0.88 (0.62–1.25)	0.481
Dementia due to Parkinson's disease	Antimigraine preparations	13	Weighted mode	0.76 (0.45–1.28)	0.326
Dementia with Lewy bodies	Antimigraine preparations	13	Inverse‐variance weighted	0.99 (0.84–1.15)	0.855
Dementia with Lewy bodies	Antimigraine preparations	13	MR Egger	0.94 (0.34–2.63)	0.91
Dementia with Lewy bodies	Antimigraine preparations	13	Weighted median	0.99 (0.8–1.22)	0.93
Dementia with Lewy bodies	Antimigraine preparations	13	Weighted mode	1.01 (0.73–1.4)	0.963
Frontotemporal dementia	Antimigraine preparations	13	Inverse‐variance weighted	0.96 (0.92–1.01)	0.127
Frontotemporal dementia	Antimigraine preparations	13	MR Egger	1.06 (0.76–1.47)	0.742
Frontotemporal dementia	Antimigraine preparations	13	Weighted median	0.95 (0.89–1.02)	0.145
Frontotemporal dementia	Antimigraine preparations	13	Weighted mode	0.94 (0.84–1.06)	0.329
Dementia in Alzheimer's disease	Antimigraine preparations	13	Inverse‐variance weighted	1.06 (0.97–1.15)	0.184
Dementia in Alzheimer's disease	Antimigraine preparations	13	MR Egger	1.75 (1.01–3.01)	0.0695
Dementia in Alzheimer's disease	Antimigraine preparations	13	Weighted median	1.08 (0.97–1.2)	0.166
Dementia in Alzheimer's disease	Antimigraine preparations	13	Weighted mode	1.14 (0.97–1.33)	0.138
Cognitive performance	Antimigraine preparations	13	Inverse‐variance weighted	0.98 (0.96–1.01)	0.163
Cognitive performance	Antimigraine preparations	13	MR Egger	0.97 (0.84–1.12)	0.693
Cognitive performance	Antimigraine preparations	13	Weighted median	0.97 (0.95–0.99)	0.0016
Cognitive performance	Antimigraine preparations	13	Weighted mode	0.97 (0.94–1)	0.0573
Vascular dementia	Antidepressants	1	Wald ratio	1.46 (0.45–4.77)	0.531
Dementia due to Parkinson's disease	Antidepressants	1	Wald ratio	0.53 (0.04–6.44)	0.622
Dementia with Lewy bodies	Antidepressants	1	Wald ratio	0.82 (0.18–3.76)	0.802
Frontotemporal dementia	Antidepressants	1	Wald ratio	1.79 (1.09–2.94)	0.0215
Dementia in Alzheimer's disease	Antidepressants	1	Wald ratio	0.86 (0.38–1.96)	0.723
Cognitive performance	Antidepressants	1	Wald ratio	1.27 (1.13–1.43)	######
Vascular dementia	Adrenergics inhalants	56	Inverse‐variance weighted	0.92 (0.83–1.02)	0.131
Vascular dementia	Adrenergics inhalants	56	MR Egger	0.99 (0.73–1.34)	0.963
Vascular dementia	Adrenergics inhalants	56	Weighted median	1.01 (0.87–1.17)	0.915
Vascular dementia	Adrenergics inhalants	56	Weighted mode	1.02 (0.82–1.27)	0.866
Dementia due to Parkinson's disease	Adrenergics inhalants	56	Inverse‐variance weighted	1.12 (0.88–1.43)	0.345
Dementia due to Parkinson's disease	Adrenergics inhalants	56	MR Egger	0.83 (0.42–1.64)	0.591
Dementia due to Parkinson's disease	Adrenergics inhalants	56	Weighted median	0.89 (0.63–1.24)	0.476
Dementia due to Parkinson's disease	Adrenergics inhalants	56	Weighted mode	0.79 (0.49–1.27)	0.337
Dementia with Lewy bodies	Adrenergics inhalants	52	Inverse‐variance weighted	1.03 (0.89–1.2)	0.672
Dementia with Lewy bodies	Adrenergics inhalants	52	MR Egger	0.76 (0.5–1.15)	0.2
Dementia with Lewy bodies	Adrenergics inhalants	52	Weighted median	1.03 (0.84–1.27)	0.781
Dementia with Lewy bodies	Adrenergics inhalants	52	Weighted mode	0.93 (0.64–1.33)	0.68
Frontotemporal dementia	Adrenergics inhalants	56	Inverse‐variance weighted	1.02 (0.97–1.07)	0.479
Frontotemporal dementia	Adrenergics inhalants	56	MR Egger	1.09 (0.94–1.26)	0.282
Frontotemporal dementia	Adrenergics inhalants	56	Weighted median	1.03 (0.97–1.11)	0.331
Frontotemporal dementia	Adrenergics inhalants	56	Weighted mode	1.05 (0.95–1.16)	0.349
Dementia in Alzheimer's disease	Adrenergics inhalants	56	Inverse‐variance weighted	0.96 (0.89–1.03)	0.228
Dementia in Alzheimer's disease	Adrenergics inhalants	56	MR Egger	0.89 (0.73–1.09)	0.264
Dementia in Alzheimer's disease	Adrenergics inhalants	56	Weighted median	0.95 (0.85–1.06)	0.334
Dementia in Alzheimer's disease	Adrenergics inhalants	56	Weighted mode	1 (0.74–1.36)	0.984
Cognitive performance	Adrenergics inhalants	56	Inverse‐variance weighted	0.98 (0.96–1)	0.0943
Cognitive performance	Adrenergics inhalants	56	MR Egger	1 (0.94–1.06)	0.945
Cognitive performance	Adrenergics inhalants	56	Weighted median	0.99 (0.97–1.01)	0.363
Cognitive performance	Adrenergics inhalants	56	Weighted mode	1 (0.97–1.02)	0.78
Cognitive performance	Adrenergics inhalants (after correction)	53	Inverse‐variance weighted	0.99 (0.97–1)	0.0312
Cognitive performance	Adrenergics inhalants (after correction)	53	MR Egger	0.98 (0.94–1.01)	0.2065
Cognitive performance	Adrenergics inhalants (after correction)	53	Weighted median	0.99 (0.98–1.01)	0.3538
Cognitive performance	Adrenergics inhalants (after correction)	53	Weighted mode	1 (0.97–1.02)	0.7805
Vascular dementia	Glucocorticoids	19	Inverse‐variance weighted	1 (0.87–1.16)	0.967
Vascular dementia	Glucocorticoids	19	MR Egger	0.82 (0.5–1.32)	0.417
Vascular dementia	Glucocorticoids	19	Weighted median	1.02 (0.84–1.24)	0.836
Vascular dementia	Glucocorticoids	19	Weighted mode	1.01 (0.72–1.4)	0.974
Dementia due to Parkinson's disease	Glucocorticoids	19	Inverse‐variance weighted	0.94 (0.63–1.42)	0.774
Dementia due to Parkinson's disease	Glucocorticoids	19	MR Egger	0.24 (0.07–0.84)	0.0399
Dementia due to Parkinson's disease	Glucocorticoids	19	Weighted median	0.72 (0.45–1.15)	0.172
Dementia due to Parkinson's disease	Glucocorticoids	19	Weighted mode	0.63 (0.31–1.26)	0.207
Dementia with Lewy bodies	Glucocorticoids	19	Inverse‐variance weighted	1.02 (0.8–1.29)	0.886
Dementia with Lewy bodies	Glucocorticoids	19	MR Egger	0.64 (0.28–1.46)	0.307
Dementia with Lewy bodies	Glucocorticoids	19	Weighted median	0.89 (0.66–1.19)	0.415
Dementia with Lewy bodies	Glucocorticoids	19	Weighted mode	0.81 (0.48–1.36)	0.43
Frontotemporal dementia	Glucocorticoids	19	Inverse‐variance weighted	1.03 (0.97–1.09)	0.407
Frontotemporal dementia	Glucocorticoids	19	MR Egger	1.03 (0.84–1.26)	0.778
Frontotemporal dementia	Glucocorticoids	19	Weighted median	1.08 (0.99–1.17)	0.078
Frontotemporal dementia	Glucocorticoids	19	Weighted mode	1.1 (0.97–1.25)	0.154
Dementia in Alzheimer's disease	Glucocorticoids	19	Inverse‐variance weighted	1.01 (0.92–1.12)	0.808
Dementia in Alzheimer's disease	Glucocorticoids	19	MR Egger	0.9 (0.64–1.26)	0.556
Dementia in Alzheimer's disease	Glucocorticoids	19	Weighted median	0.98 (0.85–1.13)	0.805
Dementia in Alzheimer's disease	Glucocorticoids	19	Weighted mode	0.85 (0.66–1.1)	0.239
Cognitive performance	Glucocorticoids	19	Inverse‐variance weighted	0.99 (0.97–1.02)	0.569
Cognitive performance	Glucocorticoids	19	MR Egger	0.92 (0.85–0.99)	0.0514
Cognitive performance	Glucocorticoids	19	Weighted median	0.98 (0.96–1)	0.122
Cognitive performance	Glucocorticoids	19	Weighted mode	0.97 (0.94–0.99)	0.0322
Vascular dementia	Antihistamines for systemic use	8	Inverse‐variance weighted	0.9 (0.69–1.16)	0.416
Vascular dementia	Antihistamines for systemic use	8	MR Egger	0.43 (0.1–1.79)	0.289
Vascular dementia	Antihistamines for systemic use	8	Weighted median	0.91 (0.65–1.27)	0.578
Vascular dementia	Antihistamines for systemic use	8	Weighted mode	0.94 (0.6–1.48)	0.798
Dementia due to Parkinson's disease	Antihistamines for systemic use	8	Inverse‐variance weighted	0.78 (0.39–1.56)	0.474
Dementia due to Parkinson's disease	Antihistamines for systemic use	8	MR Egger	0.4 (0.01–24.8)	0.678
Dementia due to Parkinson's disease	Antihistamines for systemic use	8	Weighted median	0.7 (0.34–1.43)	0.326
Dementia due to Parkinson's disease	Antihistamines for systemic use	8	Weighted mode	0.76 (0.26–2.28)	0.643
Dementia with Lewy bodies	Antihistamines for systemic use	8	Inverse‐variance weighted	1.26 (0.74–2.12)	0.394
Dementia with Lewy bodies	Antihistamines for systemic use	8	MR Egger	1.61 (0.08–31.93)	0.765
Dementia with Lewy bodies	Antihistamines for systemic use	8	Weighted median	1.25 (0.78–2)	0.344
Dementia with Lewy bodies	Antihistamines for systemic use	8	Weighted mode	1.19 (0.6–2.36)	0.634
Frontotemporal dementia	Antihistamines for systemic use	8	Inverse‐variance weighted	1.02 (0.86–1.2)	0.816
Frontotemporal dementia	Antihistamines for systemic use	8	MR Egger	1.64 (0.66–4.07)	0.33
Frontotemporal dementia	Antihistamines for systemic use	8	Weighted median	1.04 (0.88–1.23)	0.679
Frontotemporal dementia	Antihistamines for systemic use	8	Weighted mode	1.13 (0.87–1.48)	0.382
Dementia in Alzheimer's disease	Antihistamines for systemic use	8	Inverse‐variance weighted	1.02 (0.81–1.29)	0.837
Dementia in Alzheimer's disease	Antihistamines for systemic use	8	MR Egger	0.86 (0.21–3.49)	0.84
Dementia in Alzheimer's disease	Antihistamines for systemic use	8	Weighted median	1.07 (0.82–1.39)	0.63
Dementia in Alzheimer's disease	Antihistamines for systemic use	8	Weighted mode	1.33 (0.81–2.17)	0.297
Cognitive performance	Antihistamines for systemic use	8	Inverse‐variance weighted	0.97 (0.95–1)	0.044
Cognitive performance	Antihistamines for systemic use	8	MR Egger	1.04 (0.92–1.19)	0.538
Cognitive performance	Antihistamines for systemic use	8	Weighted median	0.98 (0.94–1.01)	0.157
Cognitive performance	Antihistamines for systemic use	8	Weighted mode	0.99 (0.94–1.04)	0.621
Vascular dementia	Antiglaucoma preparations and miotics	14	Inverse‐variance weighted	1.04 (0.93–1.16)	0.489
Vascular dementia	Antiglaucoma preparations and miotics	14	MR Egger	0.93 (0.67–1.31)	0.696
Vascular dementia	Antiglaucoma preparations and miotics	14	Weighted median	0.98 (0.86–1.13)	0.802
Vascular dementia	Antiglaucoma preparations and miotics	14	Weighted mode	0.96 (0.81–1.15)	0.685
Dementia due to Parkinson's disease	Antiglaucoma preparations and miotics	14	Inverse‐variance weighted	1.03 (0.82–1.28)	0.819
Dementia due to Parkinson's disease	Antiglaucoma preparations and miotics	14	MR Egger	1.16 (0.59–2.3)	0.673
Dementia due to Parkinson's disease	Antiglaucoma preparations and miotics	14	Weighted median	1.07 (0.8–1.44)	0.657
Dementia due to Parkinson's disease	Antiglaucoma preparations and miotics	14	Weighted mode	0.99 (0.69–1.44)	0.975
Dementia with Lewy bodies	Antiglaucoma preparations and miotics	14	Inverse‐variance weighted	1.05 (0.92–1.2)	0.467
Dementia with Lewy bodies	Antiglaucoma preparations and miotics	14	MR Egger	0.88 (0.57–1.36)	0.584
Dementia with Lewy bodies	Antiglaucoma preparations and miotics	14	Weighted median	1.05 (0.87–1.27)	0.635
Dementia with Lewy bodies	Antiglaucoma preparations and miotics	14	Weighted mode	1.03 (0.81–1.31)	0.842
Frontotemporal dementia	Antiglaucoma preparations and miotics	14	Inverse‐variance weighted	1.01 (0.97–1.06)	0.53
Frontotemporal dementia	Antiglaucoma preparations and miotics	14	MR Egger	1.03 (0.9–1.18)	0.645
Frontotemporal dementia	Antiglaucoma preparations and miotics	14	Weighted median	1.03 (0.97–1.09)	0.341
Frontotemporal dementia	Antiglaucoma preparations and miotics	14	Weighted mode	1.03 (0.97–1.1)	0.359
Dementia in Alzheimer's disease	Antiglaucoma preparations and miotics	14	Inverse‐variance weighted	1.01 (0.91–1.11)	0.855
Dementia in Alzheimer's disease	Antiglaucoma preparations and miotics	14	MR Egger	1.09 (0.81–1.48)	0.57
Dementia in Alzheimer's disease	Antiglaucoma preparations and miotics	14	Weighted median	1.05 (0.95–1.15)	0.372
Dementia in Alzheimer's disease	Antiglaucoma preparations and miotics	14	Weighted mode	1.06 (0.94–1.19)	0.36
Cognitive performance	Antiglaucoma preparations and miotics	14	Inverse‐variance weighted	1.01 (0.99–1.02)	0.563
Cognitive performance	Antiglaucoma preparations and miotics	14	MR Egger	1.02 (0.96–1.08)	0.545
Cognitive performance	Antiglaucoma preparations and miotics	14	Weighted median	1 (0.99–1.02)	0.562
Cognitive performance	Antiglaucoma preparations and miotics	14	Weighted mode	1 (0.98–1.02)	0.738

Abbreviation: MR, Mendelian randomization.

**TABLE 4:** Results of tests for heterogeneity and pliotropy of instrumental variables (IVs).

		Heterogeneity		Pleiotropy	
Outcome	Exposure	*Q* statistic (IVW)	*p* value	MR‐Egger Intercept	*p* value
Cognitive performance	Adrenergics inhalants	218.976	0	−0.001	0.5922
Dementia due to Parkinson's disease	Adrenergics inhalants	66.343	0.141	0.027	0.35523
Dementia in Alzheimer disease	Adrenergics inhalants	55.281	0.464	0.007	0.45445
Dementia with Lewy bodies	Adrenergics inhalants	57.408	0.25	0.027	0.12802
Frontotemporal dementia	Adrenergics inhalants	82.136	0.01	−0.006	0.37512
Vascular dementia	Adrenergics inhalants	58.085	0.362	−0.007	0.60895
Cognitive performance	Agents acting on the renin–angiotensin system	547.291	0	−0.001	0.40241
Cognitive performance	Agents acting on the renin–angiotensin system (after correction)	80.301	0.007	0.001	0.574
Dementia due to Parkinson's disease	Agents acting on the renin–angiotensin system	223.202	0.008	−0.005	0.77667
Dementia in Alzheimer disease	Agents acting on the renin–angiotensin system	341.17	0	−0.002	0.78354
Dementia with Lewy bodies	Agents acting on the renin–angiotensin system	211.475	0.025	0.008	0.48272
Frontotemporal dementia	Agents acting on the renin–angiotensin system	216.716	0.017	−0.002	0.52574
Vascular dementia	Agents acting on the renin–angiotensin system	211.739	0.03	0.002	0.79121
Vascular dementia	Agents acting on the renin–angiotensin system	198.65	0.097	0.006	0.512
Cognitive performance	Anilides	60.03	0	0.005	0.88548
Dementia due to Parkinson's disease	Anilides	5.016	0.542	−0.182	0.33928
Dementia in Alzheimer disease	Anilides	19.046	0.004	−0.079	0.48269
Dementia with Lewy bodies	Anilides	9.019	0.172	0.232	0.07259
Frontotemporal dementia	Anilides	4.317	0.634	0.02	0.57814
Vascular dementia	Anilides	25.055	0	0.013	0.94651
Cognitive performance	Antiglaucoma preparations and miotics	40.194	0	−0.002	0.64516
Dementia due to Parkinson's disease	Antiglaucoma preparations and miotics	15.335	0.287	−0.024	0.70987
Dementia in Alzheimer disease	Antiglaucoma preparations and miotics	28.341	0.008	−0.015	0.58798
Dementia with Lewy bodies	Antiglaucoma preparations and miotics	9.409	0.741	0.032	0.42511
Frontotemporal dementia	Antiglaucoma preparations and miotics	15.397	0.283	−0.004	0.78039
Vascular dementia	Antiglaucoma preparations and miotics	17.268	0.187	0.02	0.51753
Cognitive performance	Antihistamines for systemic use	6.628	0.469	−0.007	0.32773
Dementia due to Parkinson's disease	Antihistamines for systemic use	11.311	0.126	0.063	0.75915
Dementia in Alzheimer disease	Antihistamines for systemic use	11.708	0.111	0.017	0.81174
Dementia with Lewy bodies	Antihistamines for systemic use	17.686	0.013	−0.024	0.87305
Frontotemporal dementia	Antihistamines for systemic use	16.197	0.023	−0.045	0.34043
Vascular dementia	Antihistamines for systemic use	2.789	0.904	0.07	0.34192
Cognitive performance	Antihypertensives	3.839	0.279	−0.085	0.19527
Dementia due to Parkinson's disease	Antihypertensives	3.999	0.135	−1.936	0.61046
Dementia in Alzheimer disease	Antihypertensives	0.572	0.751	0.01	0.98891
Dementia with Lewy bodies	Antihypertensives	6.3	0.098	1.107	0.23627
Frontotemporal dementia	Antihypertensives	1.503	0.472	0.348	0.48784
Vascular dementia	Antihypertensives	4.241	0.12	−1.575	0.29565
Cognitive performance	Antiinflammatroy and antirheumatic products nonsteroids	20.209	0	−0.012	0.62939
Dementia due to Parkinson's disease	Antiinflammatroy and antirheumatic products nonsteroids	7.748	0.171	0.328	0.16775
Dementia in Alzheimer disease	Antiinflammatroy and antirheumatic products nonsteroids	17.217	0.004	−0.044	0.73481
Dementia with Lewy bodies	Antiinflammatroy and antirheumatic products nonsteroids	8.934	0.112	0.229	0.14399
Frontotemporal dementia	Antiinflammatroy and antirheumatic products nonsteroids	13.046	0.023	−0.02	0.77803
Vascular dementia	Antiinflammatroy and antirheumatic products nonsteroids	11.18	0.048	0.07	0.64162
Cognitive performance	Antimigraine preparations	38.388	0	0.002	0.83999
Dementia due to Parkinson's disease	Antimigraine preparations	12.697	0.391	0.08	0.55447
Dementia in Alzheimer disease	Antimigraine preparations	9.073	0.697	−0.075	0.09409
Dementia with Lewy bodies	Antimigraine preparations	7.688	0.809	0.007	0.93038
Frontotemporal dementia	Antimigraine preparations	11.382	0.497	−0.014	0.57843
Vascular dementia	Antimigraine preparations	11.282	0.505	−0.158	0.02067
Vascular dementia	Antimigraine preparations (after correction)	7.004	0.725	−0.145	0.058
Cognitive performance	Antithrombotic agents	59.989	0	−0.01	0.14872
Dementia due to Parkinson's disease	Antithrombotic agents	9.946	0.621	−0.016	0.83211
Dementia in Alzheimer disease	Antithrombotic agents	125.89	0	−0.075	0.36813
Dementia with Lewy bodies	Antithrombotic agents	28.953	0.001	−0.083	0.29717
Dementia with Lewy bodies	Antithrombotic agents (after correction)	16.717	0.053	−0.052	0.433
Frontotemporal dementia	Antithrombotic agents	10.426	0.579	−0.001	0.93353
Vascular dementia	Antithrombotic agents	31.235	0.002	0.04	0.50472
Cognitive performance	Beta blocking agents	177.778	0	0.001	0.7725
Dementia due to Parkinson's disease	Beta blocking agents	65.615	0.178	0.044	0.2533
Dementia in Alzheimer disease	Beta blocking agents	74.308	0.051	0.002	0.89606
Dementia with Lewy bodies	Beta blocking agents	86.818	0.005	0.019	0.47856
Frontotemporal dementia	Beta blocking agents	78.547	0.025	−0.008	0.31193
Vascular dementia	Beta blocking agents	55.145	0.507	0.009	0.57776
Cognitive performance	Calcium channel blockers	244.004	0	0	0.92924
Dementia due to Parkinson's disease	Calcium channel blockers	118.446	0.078	0.021	0.41177
Dementia in Alzheimer disease	Calcium channel blockers	207.645	0	−0.005	0.61876
Dementia with Lewy bodies	Calcium channel blockers	138.976	0.003	0.004	0.80725
Frontotemporal dementia	Calcium channel blockers	118.991	0.073	0	0.94422
Vascular dementia	Calcium channel blockers	124.209	0.038	0.01	0.40802
Vascular dementia	Calcium channel blockers (after correction)	111.487	0.149	0.012	0.304
Cognitive performance	Diuretics	256.797	0	0.001	0.59882
Dementia due to Parkinson's disease	Diuretics	97.062	0.339	−0.02	0.39167
Dementia in Alzheimer disease	Diuretics	135.05	0.002	−0.005	0.59627
Dementia with Lewy bodies	Diuretics	94.887	0.289	0.02	0.22592
Frontotemporal dementia	Diuretics	84.997	0.685	−0.005	0.28269
Vascular dementia	Diuretics	134.289	0.003	−0.007	0.57647
Cognitive performance	Drugs affecting bone structure and mineralization	16.763	0.053	−0.008	0.35293
Dementia due to Parkinson's disease	Drugs affecting bone structure and mineralization	16.183	0.095	0.061	0.70052
Dementia in Alzheimer disease	Drugs affecting bone structure and mineralization	9.271	0.507	0.032	0.4156
Dementia with Lewy bodies	Drugs affecting bone structure and mineralization	11.827	0.223	0.062	0.42791
Frontotemporal dementia	Drugs affecting bone structure and mineralization	8.667	0.564	0.055	0.04012
Frontotemporal dementia	Drugs affecting bone structure and mineralization	2.085	0.955	0.034	0.343
Vascular dementia	Drugs affecting bone structure and mineralization (after correction)	8.183	0.611	−0.073	0.2072
Cognitive performance	Drugs for peptic ulcer and gastro‐esophageal reflux disease (GORD)	48.913	0	0.05	0.08822
Dementia due to Parkinson's disease	Drugs for peptic ulcer and gastro‐esophageal reflux disease (GORD)	7.489	0.112	−0.326	0.34324
Dementia in Alzheimer disease	Drugs for peptic ulcer and gastro‐esophageal reflux disease (GORD)	6.294	0.178	−0.116	0.23822
Dementia with Lewy bodies	Drugs for peptic ulcer and gastro‐esophageal reflux disease (GORD)	5.431	0.246	−0.004	0.98442
Frontotemporal dementia	Drugs for peptic ulcer and gastro‐esophageal reflux disease (GORD)	3.103	0.541	0.01	0.82701
Vascular dementia	Drugs for peptic ulcer and gastro‐esophageal reflux disease (GORD)	11.032	0.026	−0.291	0.07235
Cognitive performance	Drugs used in diabetes	165.16	0	0	0.90935
Dementia due to Parkinson's disease	Drugs used in diabetes	60.25	0.292	−0.004	0.87521
Dementia in Alzheimer's disease	Drugs used in diabetes	85.297	0.005	0.007	0.45013
Dementia in Alzheimer's disease	Drugs used in diabetes (after correction)	68.11	0.094	−0.003	−0.003
Dementia with Lewy bodies	Drugs used in diabetes	62.557	0.15	0.024	0.12774
Frontotemporal dementia	Drugs used in diabetes	68.372	0.106	0.003	0.52872
Vascular dementia	Drugs used in diabetes	70.159	0.082	0.002	0.83918
Cognitive performance	Glucocorticoids	59.42	0	0.008	0.06129
Dementia due to Parkinson's disease	Glucocorticoids	33.372	0.015	0.148	0.03951
Dementia due to Parkinson's disease	Glucocorticoids (after correction)	26.627	0.064	0.099	0.292
Dementia in Alzheimer disease	Glucocorticoids	17.848	0.466	0.013	0.49196
Dementia with Lewy bodies	Glucocorticoids	30.455	0.033	0.049	0.26868
Frontotemporal dementia	Glucocorticoids	15.756	0.61	0	0.96565
Vascular dementia	Glucocorticoids	15.323	0.64	0.022	0.38888
Cognitive performance	HMG CoA reductase inhibitors	260.178	0	0	0.83247
Dementia due to Parkinson's disease	HMG CoA reductase inhibitors	86.133	0.68	−0.035	0.01253
Dementia in Alzheimer disease	HMG CoA reductase inhibitors	849.377	0	−0.026	0.06004
Dementia with Lewy bodies	HMG CoA reductase inhibitors	247.036	0	−0.039	0.00581
Frontotemporal dementia	HMG CoA reductase inhibitors	91.321	0.53	0.001	0.82171
Vascular dementia	HMG CoA reductase inhibitors	223.489	0	−0.003	0.74181
Dementia due to Parkinson's disease	HMG CoA reductase inhibitors (after correction)	61.41	0.97	−0.031	0.071
Dementia with Lewy bodies	HMG CoA reductase inhibitors (after correction)	87.245	0.472	−0.012	0.193
Dementia in Alzheimer disease	HMG CoA reductase inhibitors (after correction)	113.622	0.025	0.002	0.75
Cognitive performance	Immunosuppressants	0.001	0.979	NA	NA
Dementia due to Parkinson's disease	Immunosuppressants	10.342	0.001	NA	NA
Dementia in Alzheimer disease	Immunosuppressants	2.041	0.153	NA	NA
Frontotemporal dementia	Immunosuppressants	0.305	0.581	NA	NA
Vascular dementia	Immunosuppressants	0.04	0.841	NA	NA
Cognitive performance	Opioids	59.969	0	−0.116	0.62888
Dementia due to Parkinson's disease	Opioids	1.003	0.316	NA	NA
Dementia in Alzheimer disease	Opioids	1.077	0.299	NA	NA
Dementia with Lewy bodies	Opioids	6.646	0.036	−0.191	0.86552
Frontotemporal dementia	Opioids	0.313	0.576	NA	NA
Vascular dementia	Opioids	2.473	0.116	NA	NA
Cognitive performance	Salicylic acid and derivatives	29.389	0.001	−0.009	0.12875
Dementia due to Parkinson's disease	Salicylic acid and derivatives	6.4	0.699	−0.118	0.16205
Dementia in Alzheimer disease	Salicylic acid and derivatives	109.342	0	−0.083	0.37741
Dementia with Lewy bodies	Salicylic acid and derivatives	16.27	0.039	0.014	0.84633
Frontotemporal dementia	Salicylic acid and derivatives	4.675	0.862	0.01	0.53178
Vascular dementia	Salicylic acid and derivatives	20.234	0.017	−0.015	0.80702
Cognitive performance	Thyroid preparations	399.876	0	0	0.72493
Dementia due to Parkinson's disease	Thyroid preparations	142.257	0.168	−0.017	0.23299
Dementia in Alzheimer disease	Thyroid preparations	162.978	0.017	0	0.96867
Dementia with Lewy bodies	Thyroid preparations	147.571	0.082	0.007	0.47328
Frontotemporal dementia	Thyroid preparations	138.542	0.228	0.005	0.09406
Vascular dementia	Thyroid preparations	118.019	0.704	0.003	0.60353
Cognitive performance	Vasodilators used in cardiac diseases	0.901	0.343	NA	NA
Dementia due to Parkinson's disease	Vasodilators used in cardiac diseases	0.231	0.631	NA	NA
Dementia in Alzheimer disease	Vasodilators used in cardiac diseases	2.188	0.139	NA	NA
Dementia with Lewy bodies	Vasodilators used in cardiac diseases	0.112	0.738	NA	NA
Frontotemporal dementia	Vasodilators used in cardiac diseases	0.166	0.684	NA	NA
Vascular dementia	Vasodilators used in cardiac diseases	0.091	0.762	NA	NA

Abbreviation: MR, Mendelian randomization.

**TABLE 5:** MR‐PRESSO data.

		Raw		Outlier corrected		Global *p*	Outliers	Distortion *p*
Outcome	Exposure	OR_CI	*p*	OR_CI	*p*			
Cognitive performance	Adrenergics inhalants	0.9824 (0.9621–1.0031)	0.1	0.9855 (0.9725–0.9987)	0.0359	<0.001	3	0.515
Cognitive performance	Adrenergics inhalants (after correction)	0.9855 (0.9725–0.9987)	0.0359	NA (NA–NA)	NA	0.007	NA	NA
Cognitive performance	Agents acting on the renin–angiotensin system	1.0011 (0.9846–1.0178)	0.9011	0.9985 (0.9857–1.0116)	0.8257	<0.001	7	0.106
Cognitive performance	Anilides	0.9188 (0.7651–1.1033)	0.4059	0.9751 (0.808–1.1768)	0.8364	<0.001	4	<0.001
Cognitive performance	Antiglaucoma preparations and miotics	1.0053 (0.9874–1.0237)	0.5727	1.0001 (0.987–1.0134)	0.9894	<0.001	1	0.006
Cognitive performance	Antihistamines for systemic use	0.974 (0.9501–0.9986)	0.0773	NA (NA–NA)	NA	0.513	NA	NA
Cognitive performance	Antihypertensives	0.9907 (0.9608–1.0216)	0.5927	NA (NA–NA)	NA	0.334	NA	NA
Cognitive performance	Antiinflammatroy and antirheumatic products nonsteroids	0.9705 (0.8515–1.1062)	0.6775	0.9827 (0.8577–1.1259)	0.8249	0.007	2	0.329
Cognitive performance	Antimigraine preparations	0.9849 (0.9641–1.0062)	0.1881	0.99 (0.9718–1.0086)	0.3137	0.001	1	0.287
Cognitive performance	Antithrombotic agents	0.9718 (0.9093–1.0386)	0.4158	0.9925 (0.9567–1.0296)	0.6948	<0.001	1	0.099
Cognitive performance	Beta blocking agents	1.0046 (0.9809–1.0288)	0.7082	0.991 (0.9713–1.011)	0.3799	<0.001	4	0.27
Cognitive performance	Calcium channel blockers	0.9881 (0.973–1.0036)	0.1342	0.9932 (0.9798–1.0068)	0.3274	<0.001	5	0.191
Cognitive performance	Diuretics	0.994 (0.9779–1.0104)	0.4714	0.999 (0.984–1.0143)	0.9002	<0.001	3	0.087
Cognitive performance	Drugs affecting bone structure and mineralization	0.9992 (0.9749–1.0241)	0.9509	NA (NA–NA)	NA	0.056	NA	NA
Cognitive performance	Drugs for peptic ulcer and gastro‐esophageal reflux disease (GORD)	0.886 (0.7341–1.0693)	0.2756	0.8363 (0.7661–0.913)	0.1562	<0.001	3	<0.001
Cognitive performance	Drugs used in diabetes	1.0124 (0.9976–1.0273)	0.1071	1.0011 (0.9907–1.0116)	0.8322	<0.001	5	0.008
Cognitive performance	Glucocorticoids	0.9926 (0.9677–1.0183)	0.5765	0.9849 (0.967–1.0032)	0.1237	<0.001	1	0.623
Cognitive performance	HMG CoA reductase inhibitors	1.0016 (0.9835–1.02)	0.8636	1.0025 (0.9861–1.0192)	0.7652	<0.001	4	0.931
Cognitive performance	Salicylic acid and derivatives	0.9729 (0.9179–1.0313)	0.3799	0.9899 (0.9482–1.0335)	0.6574	<0.001	1	0.124
Cognitive performance	Thyroid preparations	1.004 (0.9933–1.0149)	0.4632	1.0063 (0.9971–1.0156)	0.1806	<0.001	8	0.739
Dementia due to Parkinson's disease	Adrenergics inhalants	1.1224 (0.8832–1.4264)	0.3492	NA (NA–NA)	NA	0.155	NA	NA
Dementia due to Parkinson's disease	Agents acting on the renin–angiotensin system	1.125 (0.9001–1.4061)	0.3021	1.0994 (0.8847–1.3663)	0.3937	0.009	1	0.777
Dementia due to Parkinson's disease	Anilides	0.4369 (0.1648–1.1582)	0.147	NA (NA–NA)	NA	0.537	NA	NA
Dementia due to Parkinson's disease	Antiglaucoma preparations and miotics	1.0262 (0.8228–1.2798)	0.8222	NA (NA–NA)	NA	0.305	NA	NA
Dementia due to Parkinson's disease	Antihistamines for systemic use	0.775 (0.3858–1.5569)	0.4971	NA (NA–NA)	NA	0.138	NA	NA
Dementia due to Parkinson's disease	Antihypertensives	1.1668 (0.5942–2.2913)	0.6845	NA (NA–NA)	NA	0.276	NA	NA
Dementia due to Parkinson's disease	Antiinflammatroy and antirheumatic products nonsteroids	0.7377 (0.179–3.0395)	0.6911	NA (NA–NA)	NA	0.204	NA	NA
Dementia due to Parkinson's disease	Antimigraine preparations	0.9678 (0.749–1.2505)	0.8064	NA (NA–NA)	NA	0.412	NA	NA
Dementia due to Parkinson's disease	Antithrombotic agents	1.0497 (0.5645–1.9518)	0.8807	NA (NA–NA)	NA	0.643	NA	NA
Dementia due to Parkinson's disease	Beta blocking agents	0.9927 (0.7373–1.3366)	0.9616	NA (NA–NA)	NA	0.214	NA	NA
Dementia due to Parkinson's disease	Calcium channel blockers	1.1179 (0.894–1.3977)	0.3307	NA (NA–NA)	NA	0.069	NA	NA
Dementia due to Parkinson's disease	Diuretics	0.947 (0.7659–1.1711)	0.6165	NA (NA–NA)	NA	0.234	NA	NA
Dementia due to Parkinson's disease	Drugs affecting bone structure and mineralization	0.8777 (0.5348–1.4404)	0.6169	NA (NA–NA)	NA	0.098	NA	NA
Dementia due to Parkinson's disease	Drugs for peptic ulcer and gastro‐esophageal reflux disease (GORD)	0.7234 (0.144–3.6346)	0.7142	NA (NA–NA)	NA	0.171	NA	NA
Dementia due to Parkinson's disease	Drugs used in diabetes	0.8765 (0.7354–1.0447)	0.1467	NA (NA–NA)	NA	0.294	NA	NA
Dementia due to Parkinson's disease	Glucocorticoids	0.9418 (0.6253–1.4187)	0.7776	NA (NA–NA)	NA	0.013	NA	NA
Dementia due to Parkinson's disease	Glucocorticoids (after correction)	1.1221 (0.7437–1.6929)	0.5902	NA (NA–NA)	NA	0.069	NA	NA
Dementia due to Parkinson's disease	HMG CoA reductase inhibitors	1.0121 (0.8119–1.2615)	0.9153	NA (NA–NA)	NA	0.67	NA	NA
Dementia due to Parkinson's disease	HMG CoA reductase inhibitors (after correction)	0.835 (0.6619–1.0534)	0.1319	NA (NA–NA)	NA	0.967	NA	NA
Dementia due to Parkinson's disease	Salicylic acid and derivatives	1.1066 (0.6018–2.0345)	0.752	NA (NA–NA)	NA	0.746	NA	NA
Dementia due to Parkinson's disease	Thyroid preparations	0.9863 (0.8606–1.1304)	0.8436	NA (NA–NA)	NA	0.121	NA	NA
Dementia in Alzheimer disease	Adrenergics inhalants	0.9571 (0.8911–1.0279)	0.2336	NA (NA–NA)	NA	0.453	NA	NA
Dementia in Alzheimer disease	Agents acting on the renin–angiotensin system	0.9681 (0.8843–1.0599)	0.4844	0.9511 (0.8829–1.0245)	0.1876	<0.001	2	0.736
Dementia in Alzheimer disease	Anilides	0.7612 (0.4081–1.4196)	0.4238	0.8994 (0.5605–1.443)	0.6785	0.012	1	0.17
Dementia in Alzheimer disease	Antiglaucoma preparations and miotics	1.0092 (0.9149–1.1132)	0.8581	1.018 (0.9534–1.087)	0.6049	0.01	2	0.847
Dementia in Alzheimer disease	Antihistamines for systemic use	1.0247 (0.812–1.2931)	0.8431	NA (NA–NA)	NA	0.111	NA	NA
Dementia in Alzheimer disease	Antihypertensives	0.8758 (0.7581–1.0118)	0.1695	NA (NA–NA)	NA	0.616	NA	NA
Dementia in Alzheimer disease	Antiinflammatroy and antirheumatic products nonsteroids	1.3842 (0.693–2.7647)	0.3993	1.3986 (0.8283–2.3614)	0.2982	0.018	2	0.743
Dementia in Alzheimer disease	Antimigraine preparations	1.0569 (0.9845–1.1345)	0.1521	NA (NA–NA)	NA	0.701	NA	NA
Dementia in Alzheimer disease	Antithrombotic agents	1.2227 (0.5893–2.5367)	0.5992	0.9435 (0.7204–1.2356)	0.6815	<0.001	2	0.038
Dementia in Alzheimer disease	Beta blocking agents	0.9868 (0.8881–1.0964)	0.805	NA (NA–NA)	NA	0.05	NA	NA
Dementia in Alzheimer disease	Calcium channel blockers	0.9965 (0.9054–1.0968)	0.9428	0.9738 (0.9088–1.0433)	0.4521	<0.001	1	0.751
Dementia in Alzheimer disease	Diuretics	0.9263 (0.8535–1.0054)	0.0699	NA (NA–NA)	NA	0.001	NA	NA
Dementia in Alzheimer disease	Drugs affecting bone structure and mineralization	0.926 (0.8182–1.0479)	0.2508	NA (NA–NA)	NA	0.505	NA	NA
Dementia in Alzheimer disease	Drugs for peptic ulcer and gastro‐esophageal reflux disease (GORD)	1.1447 (0.7056–1.8571)	0.6132	NA (NA–NA)	NA	0.221	NA	NA
Dementia in Alzheimer disease	Drugs used in diabetes	1.0366 (0.9682–1.1098)	0.3061	1.0743 (1.0074–1.1456)	0.0331	0.006	1	0.453
Dementia in Alzheimer disease	Drugs used in diabetes (after correction)	1.0743 (1.0074–1.1456)	0.0331	NA (NA–NA)	NA	0.116	NA	NA
Dementia in Alzheimer disease	Glucocorticoids	1.0123 (0.9179–1.1164)	0.8099	NA (NA–NA)	NA	0.414	NA	NA
Dementia in Alzheimer disease	HMG CoA reductase inhibitors	1.3068 (1.0466–1.6317)	0.0202	1.0316 (0.9417–1.13)	0.5052	<0.001	7	<0.001
Dementia in Alzheimer disease	Salicylic acid and derivatives	1.4023 (0.6099–3.2244)	0.4466	0.9715 (0.7234–1.3048)	0.8525	<0.001	1	<0.001
Dementia in Alzheimer disease	Thyroid preparations	1.0188 (0.9721–1.0678)	0.438	NA (NA–NA)	NA	0.021	NA	NA
Dementia with Lewy bodies	Adrenergics inhalants	1.0323 (0.8912–1.1958)	0.6735	NA (NA–NA)	NA	0.255	NA	NA
Dementia with Lewy bodies	Agents acting on the renin–angiotensin system	1.0528 (0.9172–1.2084)	0.4659	1.0419 (0.9156–1.1855)	0.5343	0.029	2	0.85
Dementia with Lewy bodies	Anilides	0.671 (0.3104–1.4508)	0.3497	NA (NA–NA)	NA	0.209	NA	NA
Dementia with Lewy bodies	Antiglaucoma preparations and miotics	1.0509 (0.9377–1.1777)	0.4084	NA (NA–NA)	NA	0.746	NA	NA
Dementia with Lewy bodies	Antihistamines for systemic use	1.2552 (0.7447–2.1156)	0.4218	1.5628 (1.0655–2.2924)	0.0624	0.023	1	0.452
Dementia with Lewy bodies	Antihypertensives	1.0888 (0.6555–1.8086)	0.764	NA (NA–NA)	NA	0.186	NA	NA
Dementia with Lewy bodies	Antiinflammatroy and antirheumatic products nonsteroids	0.9185 (0.3617–2.3326)	0.8651	NA (NA–NA)	NA	0.129	NA	NA
Dementia with Lewy bodies	Antimigraine preparations	0.9857 (0.871–1.1155)	0.8235	NA (NA–NA)	NA	0.834	NA	NA
Dementia with Lewy bodies	Antithrombotic agents	2.1506 (1.1133–4.1543)	0.0436	1.7452 (1.0057–3.0283)	0.0758	0.004	1	0.181
Dementia with Lewy bodies	Antithrombotic agents (after correction)	1.7452 (1.0057–3.0283)	0.0758	NA (NA–NA)	NA	0.094	NA	NA
Dementia with Lewy bodies	Beta blocking agents	0.9759 (0.7896–1.2061)	0.8221	NA (NA–NA)	NA	0.01	NA	NA
Dementia with Lewy bodies	Calcium channel blockers	1.0094 (0.8677–1.1742)	0.9038	0.9875 (0.857–1.1379)	0.8626	0.003	1	0.079
Dementia with Lewy bodies	Diuretics	1.0294 (0.9002–1.177)	0.6732	NA (NA–NA)	NA	0.32	NA	NA
Dementia with Lewy bodies	Drugs affecting bone structure and mineralization	0.9553 (0.7294–1.2512)	0.7474	NA (NA–NA)	NA	0.255	NA	NA
Dementia with Lewy bodies	Drugs for peptic ulcer and gastro‐esophageal reflux disease (GORD)	0.5277 (0.2283–1.2197)	0.2092	NA (NA–NA)	NA	0.288	NA	NA
Dementia with Lewy bodies	Drugs used in diabetes	0.974 (0.866–1.0954)	0.6616	NA (NA–NA)	NA	0.146	NA	NA
Dementia with Lewy bodies	Glucocorticoids	1.0176 (0.8014–1.2922)	0.8876	NA (NA–NA)	NA	0.032	NA	NA
Dementia with Lewy bodies	HMG CoA reductase inhibitors	1.3459 (1.0657–1.6997)	0.0144	1.0327 (0.8875–1.2017)	0.6779	<0.001	4	<0.001
Dementia with Lewy bodies	HMG CoA reductase inhibitors (after correction)	1.0327 (0.8875–1.2017)	0.6779	NA (NA–NA)	NA	0.508	NA	NA
Dementia with Lewy bodies	Salicylic acid and derivatives	2.6978 (1.5151–4.8038)	0.0082	NA (NA–NA)	NA	0.085	NA	NA
Dementia with Lewy bodies	Thyroid preparations	0.976 (0.8929–1.0668)	0.593	NA (NA–NA)	NA	0.071	NA	NA
Frontotemporal dementia	Adrenergics inhalants	1.0193 (0.9667–1.0747)	0.4822	NA (NA–NA)	NA	0.017	NA	NA
Frontotemporal dementia	Agents acting on the renin–angiotensin system	1.0356 (0.9904–1.0828)	0.1266	1.0297 (0.9865–1.0748)	0.1821	0.009	1	0.744
Frontotemporal dementia	Anilides	1.1238 (0.9389–1.3451)	0.2503	NA (NA–NA)	NA	0.673	NA	NA
Frontotemporal dementia	Antiglaucoma preparations and miotics	1.0142 (0.9706–1.0597)	0.5408	NA (NA–NA)	NA	0.318	NA	NA
Frontotemporal dementia	Antihistamines for systemic use	1.02 (0.8638–1.2044)	0.8222	1.0889 (0.9575–1.2382)	0.2419	0.031	1	0.628
Frontotemporal dementia	Antihypertensives	1.1394 (1.0518–1.2344)	0.0494	NA (NA–NA)	NA	0.69	NA	NA
Frontotemporal dementia	Antiinflammatroy and antirheumatic products nonsteroids	1.1917 (0.8267–1.7178)	0.3905	1.0548 (0.7748–1.4361)	0.7515	0.048	1	0.12
Frontotemporal dementia	Antimigraine preparations	0.9621 (0.9168–1.0097)	0.1426	NA (NA–NA)	NA	0.51	NA	NA
Frontotemporal dementia	Antithrombotic agents	1.0662 (0.9392–1.2103)	0.3412	NA (NA–NA)	NA	0.605	NA	NA
Frontotemporal dementia	Beta blocking agents	1.0501 (0.9837–1.121)	0.148	NA (NA–NA)	NA	0.023	NA	NA
Frontotemporal dementia	Calcium channel blockers	1.0376 (0.9938–1.0833)	0.0967	NA (NA–NA)	NA	0.146	NA	NA
Frontotemporal dementia	Diuretics	1.0561 (1.0138–1.1002)	0.0102	NA (NA–NA)	NA	0.346	NA	NA
Frontotemporal dementia	Drugs affecting bone structure and mineralization	1.0336 (0.9612–1.1114)	0.3931	NA (NA–NA)	NA	0.589	NA	NA
Frontotemporal dementia	Drugs affecting bone structure and mineralization (after correction)	1.053 (1.0015–1.1071)	0.0831	NA (NA–NA)	NA	0.969	NA	NA
Frontotemporal dementia	Drugs for peptic ulcer and gastro‐esophageal reflux disease (GORD)	0.9497 (0.7726–1.1673)	0.6496	NA (NA–NA)	NA	0.57	NA	NA
Frontotemporal dementia	Drugs used in diabetes	0.999 (0.9625–1.0369)	0.9576	NA (NA–NA)	NA	0.084	NA	NA
Frontotemporal dementia	Glucocorticoids	1.0255 (0.9699–1.0844)	0.3877	NA (NA–NA)	NA	0.606	NA	NA
Frontotemporal dementia	HMG CoA reductase inhibitors	1.0082 (0.9639–1.0546)	0.7212	NA (NA–NA)	NA	0.52	NA	NA
Frontotemporal dementia	Salicylic acid and derivatives	1.1301 (1.0184–1.254)	0.0467	NA (NA–NA)	NA	0.86	NA	NA
Frontotemporal dementia	Thyroid preparations	1.0239 (0.996–1.0527)	0.0965	NA (NA–NA)	NA	0.063	NA	NA
Vascular dementia	Adrenergics inhalants	0.9218 (0.8294–1.0246)	0.1369	NA (NA–NA)	NA	0.371	NA	NA
Vascular dementia	Agents acting on the renin–angiotensin system	1.1132 (1.0048–1.2334)	0.0417	1.098 (0.9936–1.2135)	0.0683	0.045	1	0.742
Vascular dementia	Agents acting on the renin–angiotensin system (after correction)	1.098 (0.9936–1.2135)	0.0683	NA (NA–NA)	NA	0.152	NA	NA
Vascular dementia	Anilides	0.8338 (0.2981–2.3318)	0.7408	0.8123 (0.3453–1.9106)	0.6586	<0.001	2	0.952
Vascular dementia	Antiglaucoma preparations and miotics	1.0398 (0.931–1.1612)	0.5011	NA (NA–NA)	NA	0.232	NA	NA
Vascular dementia	Antihistamines for systemic use	0.8982 (0.7629–1.0576)	0.2388	NA (NA–NA)	NA	0.896	NA	NA
Vascular dementia	Antihypertensives	1.4284 (1.0517–1.9399)	0.1066	NA (NA–NA)	NA	0.334	NA	NA
Vascular dementia	Antiinflammatroy and antirheumatic products nonsteroids	1.0296 (0.462–2.2946)	0.9459	NA (NA–NA)	NA	0.067	NA	NA
Vascular dementia	Antimigraine preparations	0.9257 (0.826–1.0373)	0.2084	NA (NA–NA)	NA	0.497	NA	NA
Vascular dementia	Antimigraine preparations (after correction)	0.9151 (0.8178–1.024)	0.1529	NA (NA–NA)	NA	0.745	NA	NA
Vascular dementia	Antithrombotic agents	0.9516 (0.5647–1.6035)	0.8552	0.8523 (0.6515–1.1148)	0.2704	0.002	2	0.671
Vascular dementia	Beta blocking agents	1.1329 (0.9964–1.2881)	0.0617	NA (NA–NA)	NA	0.579	NA	NA
Vascular dementia	Calcium channel blockers	1.1511 (1.0313–1.2849)	0.0136	1.137 (1.0232–1.2635)	0.0188	0.012	1	0.791
Vascular dementia	Calcium channel blockers (after correction)	1.137 (1.0232–1.2635)	0.0188	NA (NA–NA)	NA	0.057	NA	NA
Vascular dementia	Diuretics	1.1287 (1.0099–1.2615)	0.0352	NA (NA–NA)	NA	0.011	NA	NA
Vascular dementia	Drugs affecting bone structure and mineralization	0.8842 (0.7486–1.0443)	0.1779	NA (NA–NA)	NA	0.616	NA	NA
Vascular dementia	Drugs for peptic ulcer and gastro‐esophageal reflux disease (GORD)	1.5489 (0.6131–3.9127)	0.4072	NA (NA–NA)	NA	0.061	NA	NA
Vascular dementia	Drugs used in diabetes	1.0636 (0.9732–1.1623)	0.1793	NA (NA–NA)	NA	0.065	NA	NA
Vascular dementia	Glucocorticoids	1.003 (0.8801–1.1431)	0.9645	NA (NA–NA)	NA	0.63	NA	NA
Vascular dementia	HMG CoA reductase inhibitors	1.1246 (0.9516–1.329)	0.1715	0.961 (0.8462–1.0914)	0.5417	<0.001	4	0.031
Vascular dementia	Salicylic acid and derivatives	1.0402 (0.6222–1.739)	0.8837	0.8335 (0.626–1.1099)	0.2478	0.023	1	0.298
Vascular dementia	Thyroid preparations	0.9675 (0.9124–1.026)	0.2721	NA (NA–NA)	NA	0.64	NA	NA

Abbreviations: CI, confidence interval; MR, Mendelian randomization; OR, odds ratio.

No causal effect was observed for other exposure factors and outcomes (all *p* > 0.05, Table 3). Yet, MR‐Egger regression indicated that some results were affected by horizontal pleiotropy, including HMG CoA reductase inhibitors versus dementia due to Parkinson's disease, glucocorticoids versus dementia due to Parkinson's disease, drugs affecting bone structure and mineralization versus FTD, and antimigraine preparations versus vascular dementia (all *p* < 0.05, Table 4). After correction, that is, elimination of some SNPs, no heterogeneity rate was significantly reduced (Table 4).

After correction, that is, elimination of some SNPs, drugs used in diabetes resulted in being a risk factor for dementia in Alzheimer's disease (OR = 1.07, 95% CI = 1–1.14, *p* = 0.034, Table 3), whereas adrenergic inhalants resulted in a protective factor against cognitive performance (OR = 0.99, 95% CI = 0.97–1, *p* = 0.031, Table 3). The MR‐Egger regression indicated that results were unaffected by horizontal pleiotropy. Moreover, no heterogeneity was detected (Table 4).

### 3.3 Statistical Power

To evaluate the robustness of our findings, we calculated the statistical power of significant associations (Table 5; full results in Table S24). Some outcomes, such as antihistamines with cognitive performance and salicylic acid with DLB, could not be assessed and are reported as NA. For other associations, the power was generally low—for instance, calcium channel blockers with vascular dementia (13.8%) and diuretics with FTD (5.1%). These results suggest that, despite nominal significance, several associations may be underpowered and should be interpreted with caution.

## 4 Discussion

Given that dementia usually affects individuals older than 65, this condition is commonly associated with various comorbid conditions whose management usually relies on the usage of drugs that can further complicate the onset and progression of dementia (Tisher and Salardini 2019). Herein, we used two‐sample MR to investigate the causal relationship between drug use and different types of dementia, finding that antithrombotic agents, HMG CoA reductase inhibitors, and salicylic acid were strong risk factors for DLB; diuretics were a potential risk factor for vascular dementia; thyroid preparations and diuretics, immunosuppressants resulted as a risk factor for FTD; HMG CoA reductase inhibitors were a risk factor for dementia in Alzheimer's disease; and antihistamines for systemic use were a protective factor for cognitive performance. In interpreting these findings, we note that statistical power was low for several associations (e.g., calcium channel blockers with vascular dementia: 13.8%; diuretics with FTD: 5.1%). Thus, although nominally significant, these signals may be underpowered and should be regarded as exploratory pending replication.

DLB is a type of progressive dementia characterized by cognitive decline, visual hallucinations, and Parkinsonism (Gomperts 2016). Research into the risk factors associated with DLB has identified various potential influences, including certain medications and health conditions. A previous study (Rahman et al. 2023) has reported conflicting results on the role of oral anticoagulants on the onset of dementia, failing to detect any significant reduction in the risk of dementia and detecting an association between the use of oral anticoagulants and reduced occurrence of dementia, which is contrary with our results. This inconsistency with our findings could be due to the nature of the previous studies, which were cross‐sectional and thus could not confirm the temporal relationship (Mongkhon et al. 2019). Moreover, we only found this effect for DLB, suggesting there might be a different underlying mechanism driving the relationship between other subtypes of dementia and antithrombotic agents (Raz et al. 2016). Mechanistically, antithrombotic exposure could contribute to cerebral microbleeds or intracranial hemorrhage in susceptible older adults, potentially aggravating α‐synuclein–related network vulnerability characteristic of DLB (Simon et al. 2021). HMG‐CoA reductase inhibitors, also known as statins, are used to lower cholesterol levels in the blood. They effectively reduce the production of cholesterol, which can help lower the risk of cardiovascular diseases, including heart attacks and strokes (Tobert 2003). It has been suggested that statins may exert neuroprotective effects due to their anti‐inflammatory properties and ability to influence cholesterol metabolism in the brain (van der Most et al. 2009). In fact, a previous study found that statin use was associated with a 28% lower risk of Alzheimer's disease, an 18% lower risk of vascular dementia, and a 20% lower risk of unspecified dementia (Ren et al. 2024), which is again inconsistent with our results and could be explained by the specific molecular mechanism driving different subtypes of dementia (Raz et al. 2016). Heterogeneity in blood–brain barrier penetration (lipophilic vs. hydrophilic statins), dose, and effects on membrane lipid rafts and APP processing may also yield subtype‐specific cognitive effects (Li et al. 2018). Salicylic acid is a common anti‐inflammatory and analgesic compound in medications like aspirin. According to previous studies, nonsteroidal anti‐inflammatory drugs (NSAIDs), including aspirin, may have a protective effect against the development of certain types of dementia, including Alzheimer's disease (Gasparini et al. 2004). Conversely, salicylic acid drugs may also affect the balance of neurochemicals in the brain (Li et al. 2023), especially when used over a long period of time, thus exacerbating degenerative neuronal changes and leading to an increased risk of DLB.

Vascular dementia is a type of dementia caused by problems in the supply of blood to the brain, which usually occurs due to strokes or other conditions affecting the blood vessels. Calcium channel blockers are a risk factor for vascular dementia. They are commonly used to treat high blood pressure and heart disease, but they may increase the risk of vascular dementia by affecting blood flow and vascular health (Kalaria 2018). Vascular dementia is strongly associated with vascular damage and inadequate blood supply to the brain, and calcium channel blockers may negatively affect vascular function during long‐term use, thereby promoting the development of dementia (Kalaria 2018). Furthermore, our results showed that diuretics were a potential risk factor for this type of dementia, which could be associated with fluid balance and dehydration that can exacerbate cognitive deficits (Wittbrodt and Millard‐Stafford 2018). Diuretics are widely used to treat diseases such as high blood pressure and heart failure, but their long‐term use may cause fluid and electrolyte imbalances, affecting vascular function, which, in turn, increases the risk of vascular dementia (Qavi et al. 2015). In addition, diuretics may be associated with the development of FTD by interfering with cerebral blood flow, neuronal health, and metabolic processes. Nevertheless, given the generally low power for these associations (Table 6), these findings should be interpreted cautiously and validated in larger MR datasets.

**TABLE 6:** Statistical power of significant associations between drug exposure and dementia/cognitive outcomes.

**Exposure**	**Outcome**	**IVW_OR**	**Power (%)**
Antihistamines for systemic use	Cognitive performance	0.97	NA
Calcium channel blockers	Vascular dementia	1.146	13.80
Diuretics	Vascular dementia	1.128	11.98
Diuretics	Frontotemporal dementia	1.062	5.08
Immunosuppressants	Frontotemporal dementia	1.066	5.01
Immunosuppressants	Cognitive performance	0.98	NA
Salicylic acid and derivatives	Dementia with Lewy bodies	2.770	NA
Thyroid preparations	Frontotemporal dementia	1.034	5.04
Drugs used in diabetes	Dementia in Alzheimer's disease	1.072	7.98
Adrenergics inhalants	Cognitive performance	0.98	NA

Abbreviations: IVW, inverse‐variance weighted; OR, odds ratio.

FTD refers to a group of neurodegenerative disorders characterized by progressive degeneration of the frontal and temporal lobes of the brain. Herein, we found that diuretics, thyroid preparations, and immunosuppressants were risk factors for FTD. Diuretics may be associated with the development of frontotemporal lobe dementia in some cases by interfering with cerebral blood flow, neuronal health, and metabolic processes. Furthermore, thyroid medications are used for the treatment of hypothyroidism, but their prolonged use may adversely affect the metabolic and neurological functions of the brain. The regulation of thyroid hormones is closely linked to metabolic and cognitive functions in the brain, and excessive or prolonged use of thyroid medications may negatively affect the brain, thus increasing the risk of frontotemporal lobe dementia (Smith et al. 2002). Immunosuppressants are commonly used in the treatment of organ transplants or autoimmune disorders to reduce inflammation by suppressing the immune system. However, long‐term use of immunosuppressants may increase the risk of FTD by affecting the immune response and neurodegenerative changes in the brain (Alberici et al. 2018). These mechanistic hypotheses align with prior observational signals, but replication with greater statistical power and functional follow‐up is warranted.

Alzheimer's disease is a progressive neurodegenerative disorder and the most common cause of dementia, accounting for 60%–80% of dementia cases. The exact cause of Alzheimer's remains unclear; however, the condition has been associated with the accumulation of amyloid plaques and tau tangles in the brain, death of neurons, and brain tissue loss. In the present study, we identified statins as the risk factor for Alzheimer's. Existing research on this relationship has generated mixed findings. Some studies have suggested that statins may exert a protective effect against the development of Alzheimer's disease and other forms of dementia, potentially due to their anti‐inflammatory and neuroprotective properties (Haag et al. 2009), whereas others reported that statins may influence the metabolism of amyloid‐beta, a protein that accumulates in the brains of Alzheimer's patients (Haag et al. 2009). However, other studies have not found a significant association between statin use and a reduced risk of Alzheimer's disease. These inconsistencies could be due to different factors, such as the specific type of statin used, the dosage, and the duration of use. Our MR findings add to this heterogeneous literature and underscore the need to consider class, dose, and CNS penetration when interpreting statin–cognition relationships.

Finally, antihistamines for systemic use were found to have a protective effect on cognitive performance (Bachurin et al. 2001). Antihistamines are primarily used to treat allergic reactions, but some studies have suggested that certain antihistamines may have effects on cognitive performance, which is consistent with our results. Unlike first‐generation antihistamines, such as diphenhydramine, which are known to cross the blood–brain barrier and exert sedative effects, thus impairing cognitive function, especially in older adults, second‐generation antihistamines, like loratadine and cetirizine, are less likely to cause sedation and are generally considered safer in terms of cognitive effects (Kay 2000). However, because power was not estimable for this continuous outcome (NA in table S24), this protective association should be viewed as hypothesis‐generating.

### 4.1 Clinical Implications and Future Directions

From a geriatric prescribing perspective, our results support careful medication review and deprescribing where appropriate in older adults with neurocognitive disorders. Practical steps include the following: preferential use of the lowest effective dose; periodic review of antithrombotic indications with individualized bleed‐risk assessment; consideration of statin class and lipophilicity when cognitive concerns arise; vigilant monitoring of electrolytes and hydration in patients on chronic diuretics; and caution with long‐term salicylate exposure in patients at high cerebrovascular risk. These proposals align with contemporary deprescribing frameworks and guidance for older adults with neurocognitive disorders (Zhou et al. 2021).

### 4.2 Strengths and Limitations

Strengths include the use of large‐scale GWAS, a two‐sample MR framework that reduces confounding and reverse causation, and multiple sensitivity analyses (MR Egger, weighted median, MR‐PRESSO). Key limitations are (1) limited statistical power for several associations and NA values for some continuous outcomes, which raise the possibility of false positives or missed small effects; (2) restriction to European ancestry, limiting generalizability; (3) residual horizontal pleiotropy cannot be fully excluded despite diagnostics; and (4) exposure definitions based on medication classes may mask heterogeneity within classes (e.g., lipophilic vs. hydrophilic statins). Future work should expand non‐European samples, refine drug‐class instruments, and integrate functional studies or pragmatic trials to test mechanistic hypotheses and replicate low‐power signals identified here.

## 5 Conclusion

As the global population ages, the prevalence of dementia is expected to rise, highlighting the importance of research in this field. Our findings add to the existing knowledge of the intricate association relationship between drug use and cognitive impairment, that is, different types of dementia. Given the generally low or non‐estimable power for several associations, these results should be interpreted cautiously and validated in larger cohorts. Nevertheless, future research is needed to further understand the mechanisms at play and to determine which drugs can be definitively classified as risk factors for the onset and progression of dementia.

## Author Contributions

Bing Yan and Zhugui Chen carried out the studies, participated in collecting data, and drafted the manuscript. Bing Yan and Zhugui Chen performed the statistical analysis and participated in its design. Zhugui Chen and Dan Yang participated in acquisition, analysis, or interpretation of data and draft the manuscript. All authors read and approved the final manuscript.

## Funding

The authors have nothing to report.

## Ethics Statement

The authors have nothing to report.

## Consent

The authors have nothing to report.

## Conflicts of Interest

The authors declare no conflicts of interest.

## Supporting information




**Supporting Fig.1**: The IVW analysis indicates that antithrombotic agents are a risk factor for dementia with Lewy bodies: (A) forest plot; (B) funnel plot; (C) loo plot.

**Supporting Fig.2**: The IVW analysis indicates that HMG CoA reductase inhibitors are a risk factor for dementia with Lewy bodies: (A) forest plot; (B) funnel plot; (C) loo plot.

**Supporting Fig.3**: The IVW analysis indicates that salicylic acid and derivatives are a risk factor for dementia with Lewy bodies: (A) forest plot; (B) funnel plot; (C) loo plot.

**Supporting Fig.4**: The IVW analysis indicates that diuretics are a risk factor for vascular dementia: (A) forest plot; (B) funnel plot; (C) loo plot.

**Supporting Fig.5**: The IVW analysis indicates that calcium channel blockers are a risk factor for vascular dementia: (A) forest plot; (B) funnel plot; (C) loo plot.

**Supporting Fig.6**: The IVW analysis indicates that thyroid preparations are a risk factor for thyroid preparations: (A) forest plot; (B) funnel plot; (C) loo plot.

**Supporting Fig.7**: The IVW analysis indicates that diuretics are a risk factor for thyroid preparations: (A) forest plot; (B) funnel plot; (C) loo plot.

**Supporting Fig.8**: The IVW analysis indicates that immunosuppressants are a risk factor for thyroid preparations: (A) forest plot; (B) funnel plot; (C) loo plot.

**Supporting Fig.9**: The IVW analysis indicates that HMG CoA reductase inhibitors are a risk factor for dementia in Alzheimer's disease: (A) forest plot; (B) funnel plot; (C) loo plot.

Supplementary Tables: brb371057‐sup‐0010‐tablesS1‐S24.docx

## Data Availability

All data generated or analyzed during this study are included in this published article.
