# Supplementary figures and images for: A Causal Association Between Drug Use and Cognitive Impairment: A Two‐Sample Mendelian Randomization Study
Source: Brain Behav. 2025 Nov 11;15(11):e71057. doi: 10.1002/brb3.71057 (PMC12606049; doi:10.1002/brb3.71057)

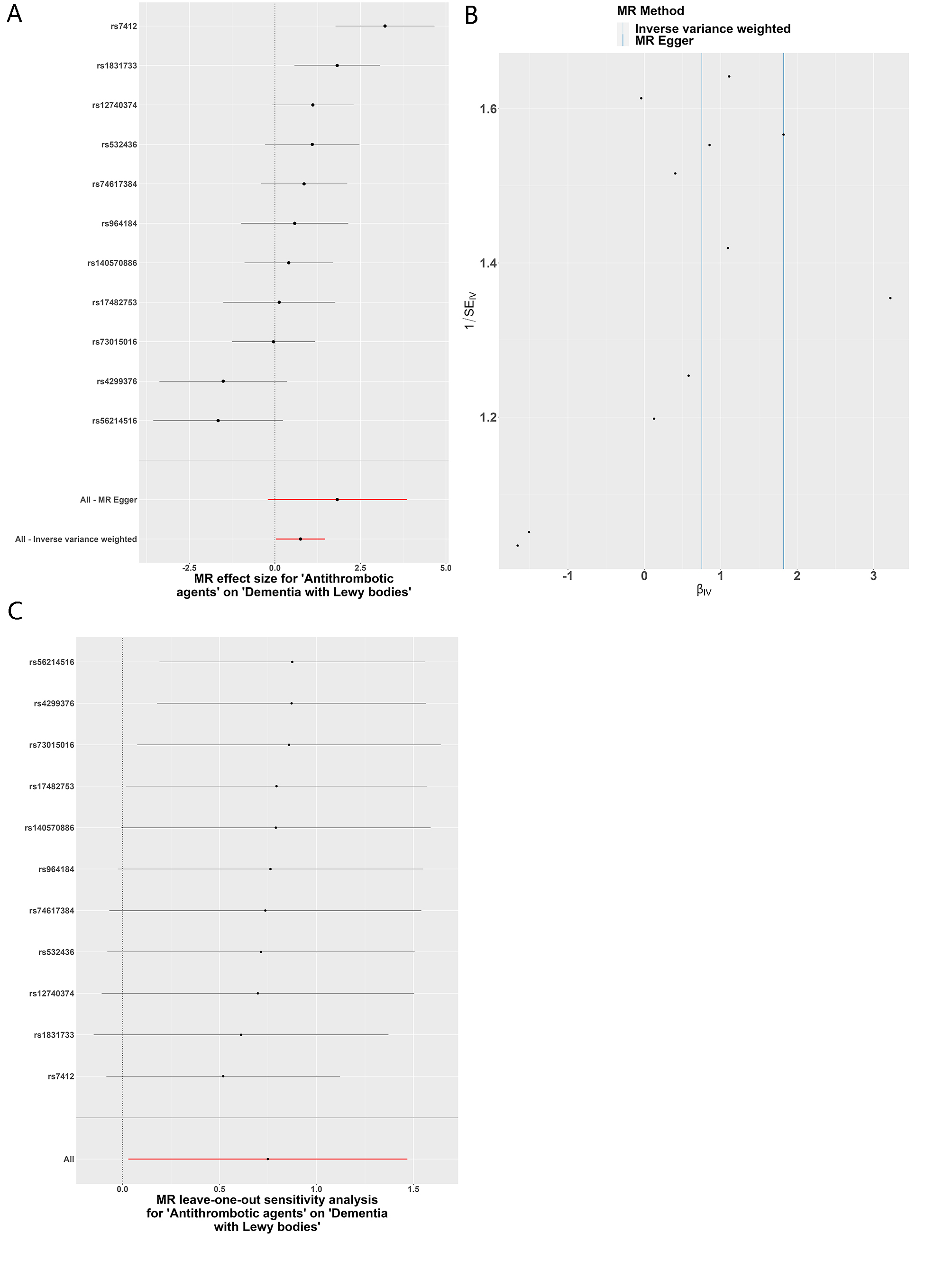

Supplement: Supplementary file 1 — Supporting Fig.1: The IVW analysis indicates that antithrombotic agents are a risk factor for dementia with Lewy bodies: (A) forest plot; (B) funnel plot; (C) loo plot. [file BRB3-15-e71057-s007.jpg]

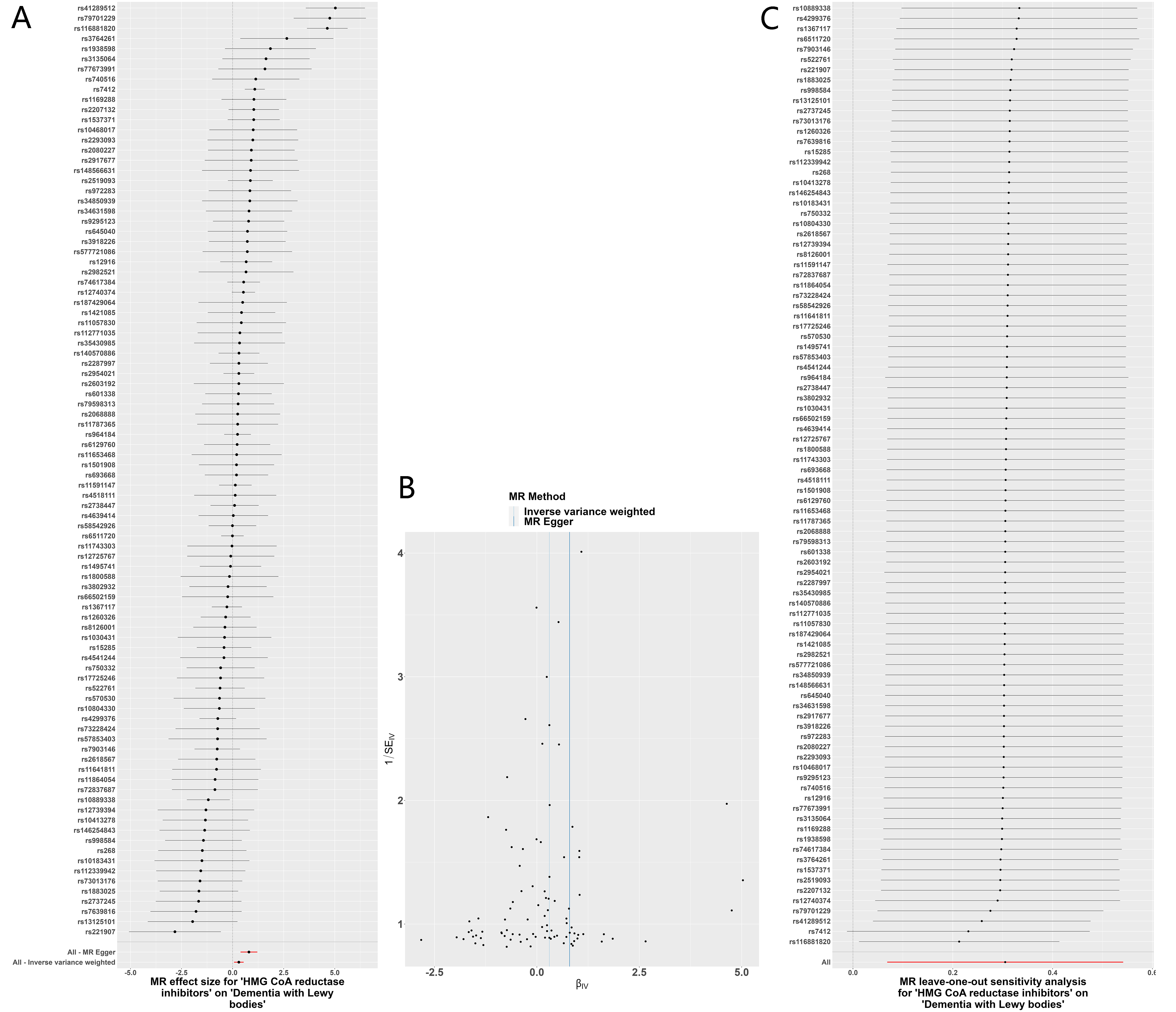

Supplement: Supplementary file 2 — Supporting Fig.2: The IVW analysis indicates that HMG CoA reductase inhibitors are a risk factor for dementia with Lewy bodies: (A) forest plot; (B) funnel plot; (C) loo plot. [file BRB3-15-e71057-s009.jpg]

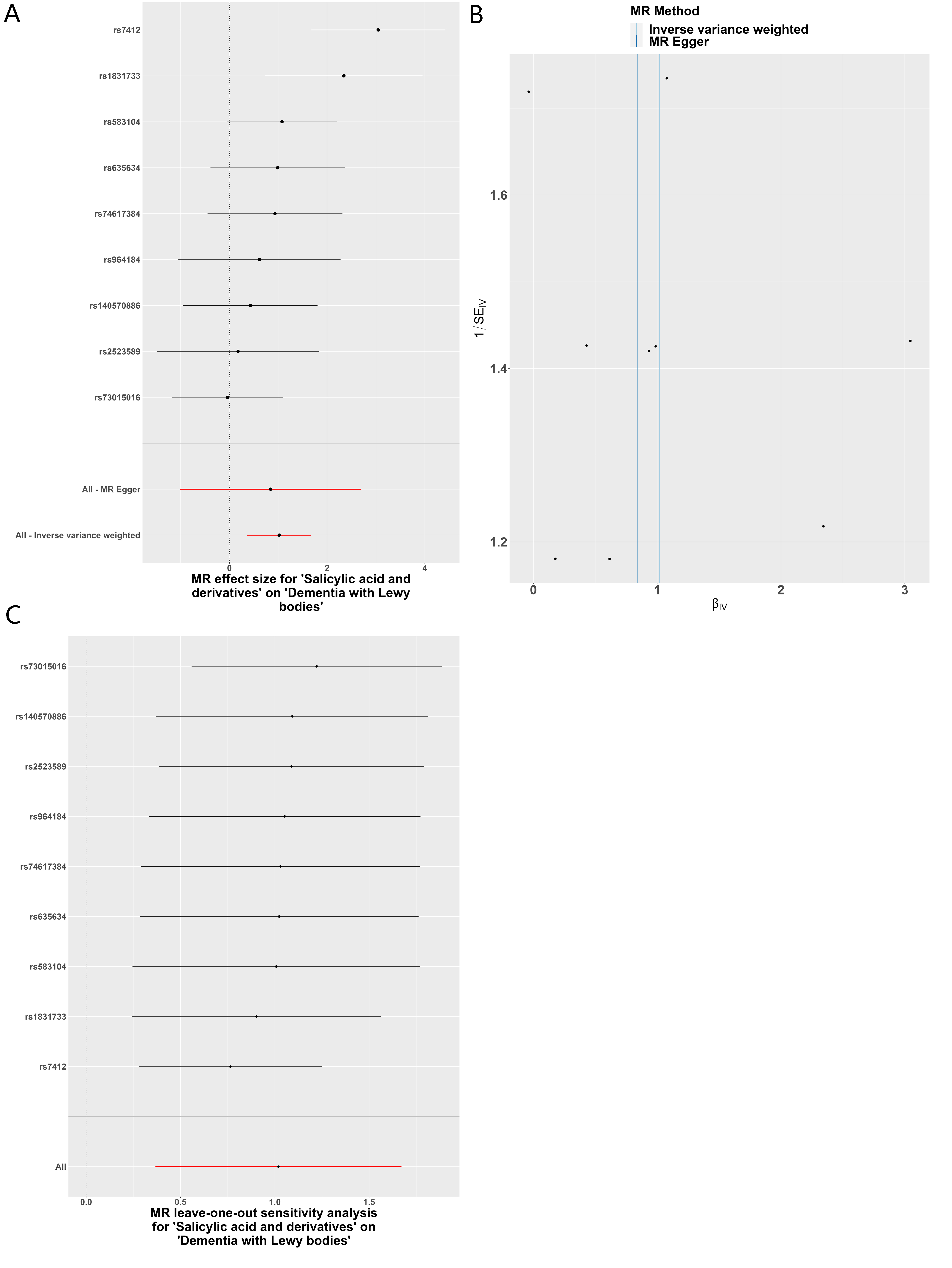

Supplement: Supplementary file 3 — Supporting Fig.3: The IVW analysis indicates that salicylic acid and derivatives are a risk factor for dementia with Lewy bodies: (A) forest plot; (B) funnel plot; (C) loo plot. [file BRB3-15-e71057-s002.jpg]

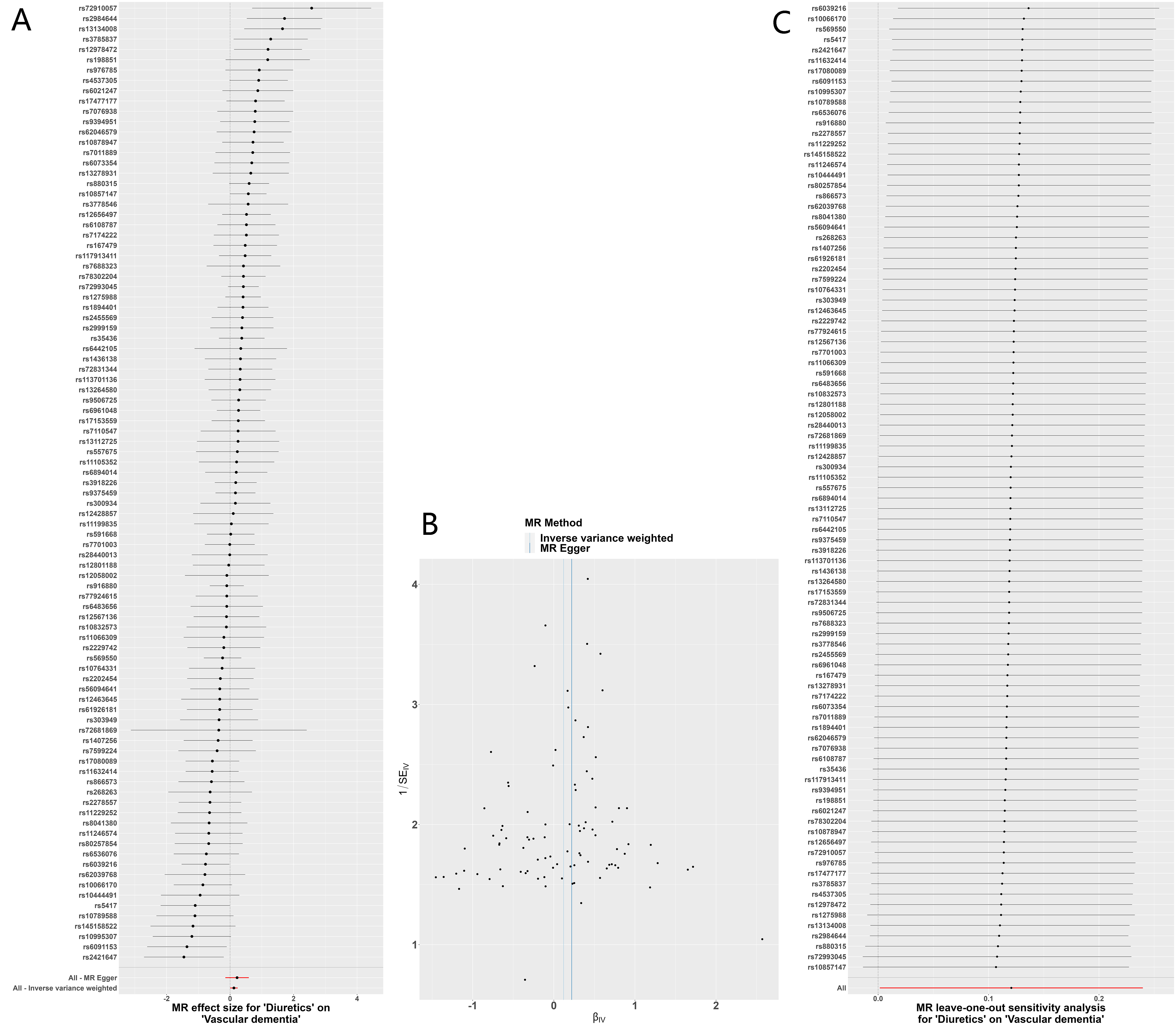

Supplement: Supplementary file 4 — Supporting Fig.4: The IVW analysis indicates that diuretics are a risk factor for vascular dementia: (A) forest plot; (B) funnel plot; (C) loo plot. [file BRB3-15-e71057-s004.jpg]

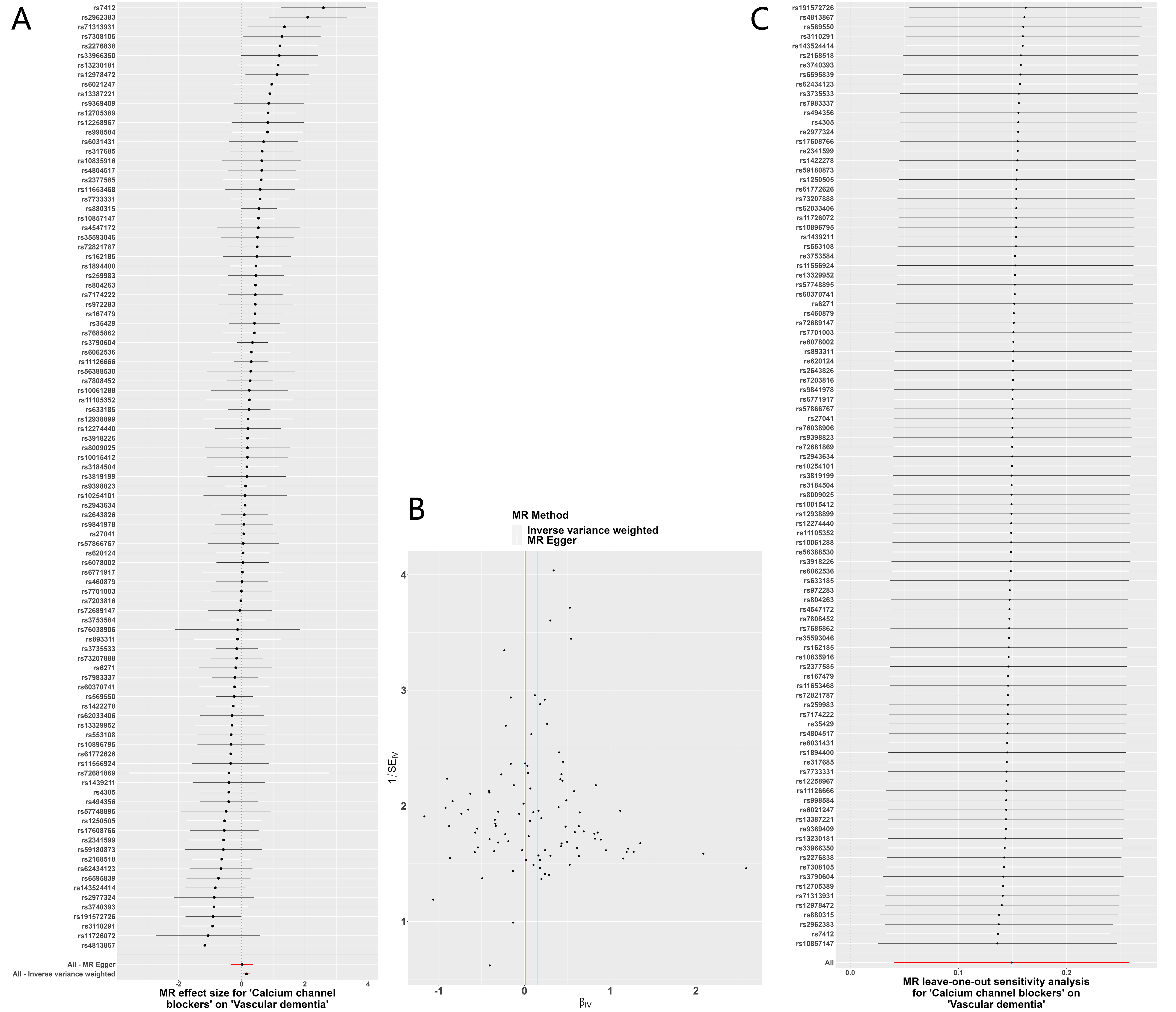

Supplement: Supplementary file 5 — Supporting Fig.5: The IVW analysis indicates that calcium channel blockers are a risk factor for vascular dementia: (A) forest plot; (B) funnel plot; (C) loo plot. [file BRB3-15-e71057-s003.jpg]

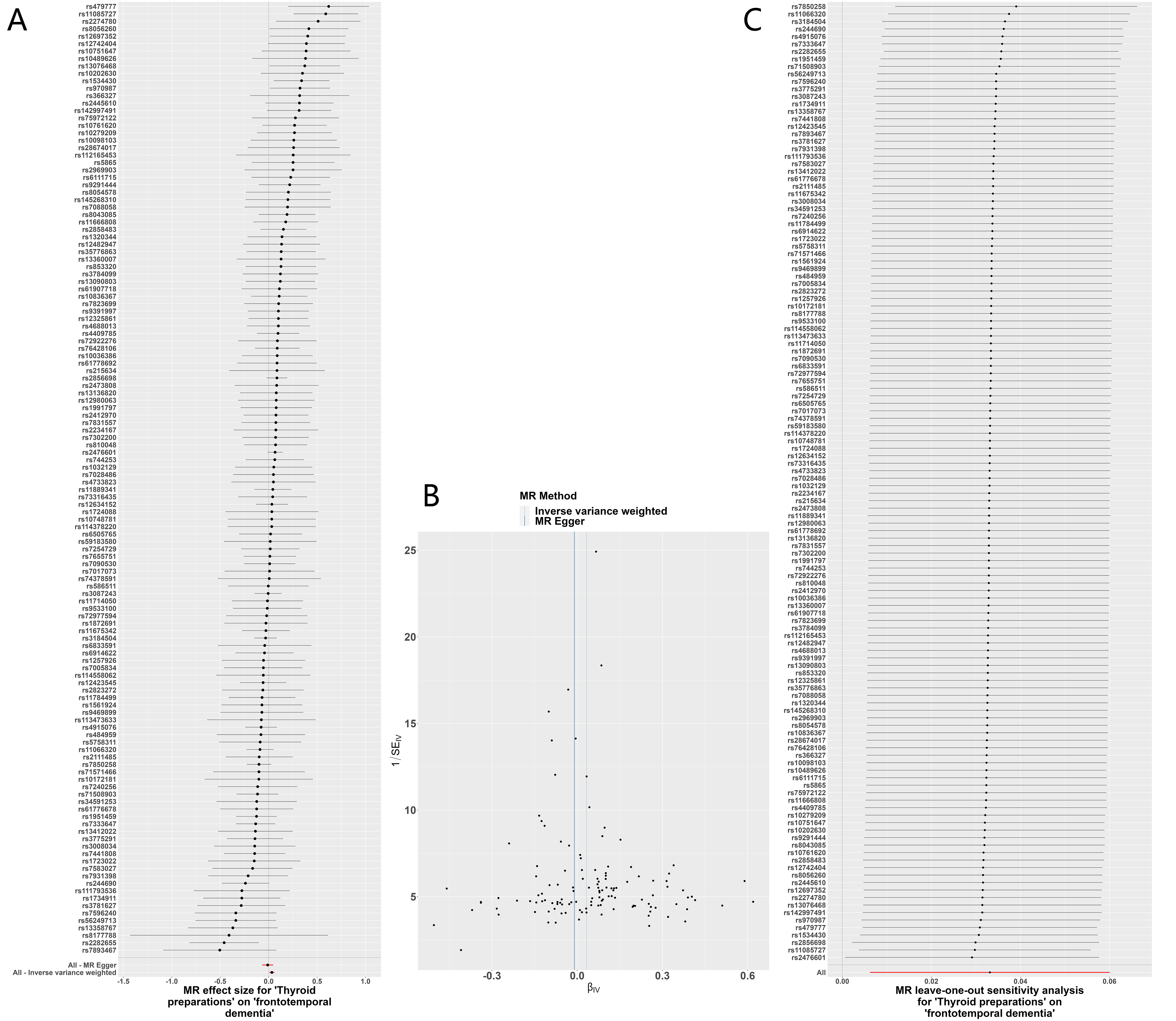

Supplement: Supplementary file 6 — Supporting Fig.6: The IVW analysis indicates that thyroid preparations are a risk factor for thyroid preparations: (A) forest plot; (B) funnel plot; (C) loo plot. [file BRB3-15-e71057-s008.jpg]

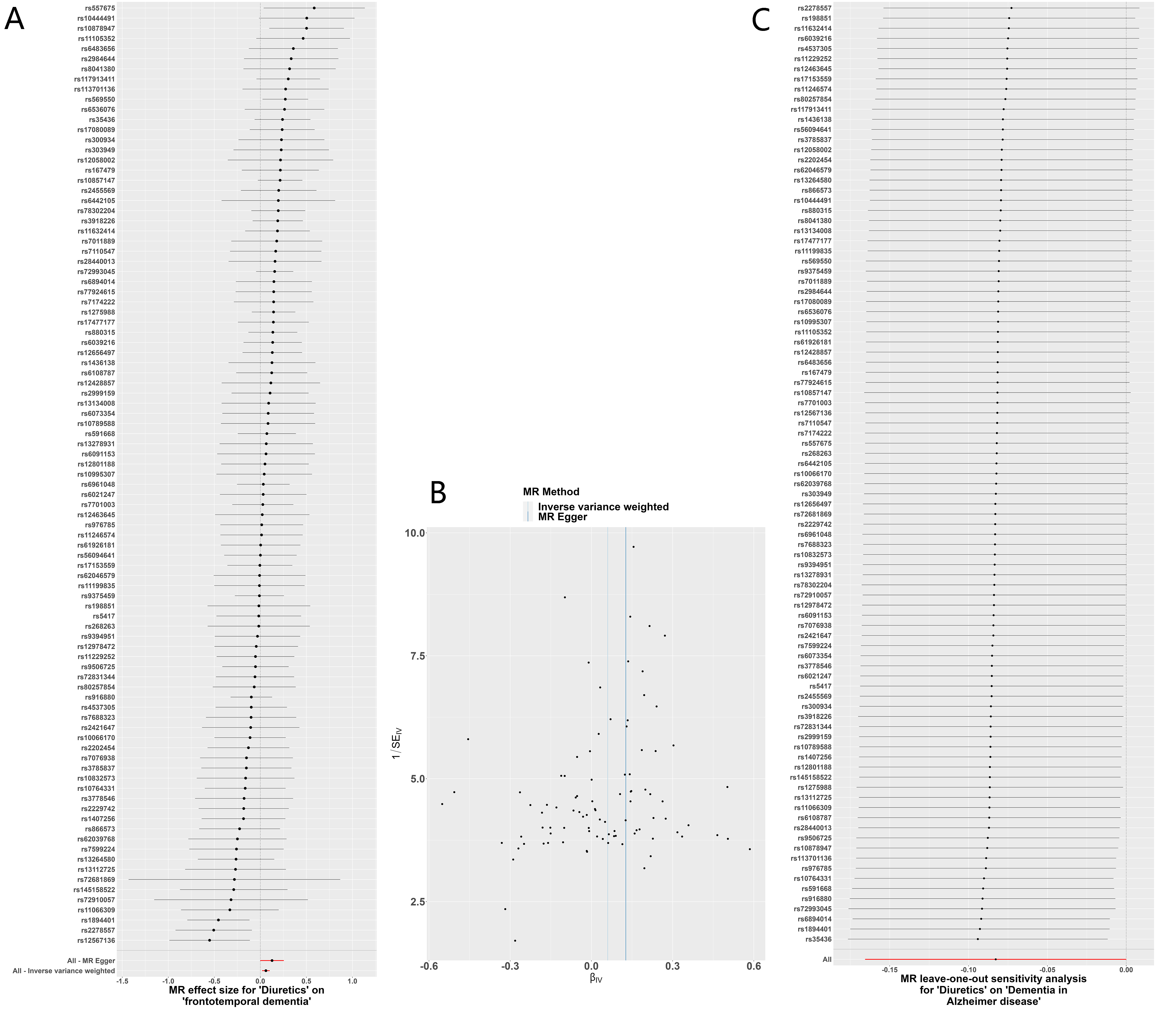

Supplement: Supplementary file 7 — Supporting Fig.7: The IVW analysis indicates that diuretics are a risk factor for thyroid preparations: (A) forest plot; (B) funnel plot; (C) loo plot. [file BRB3-15-e71057-s005.jpg]

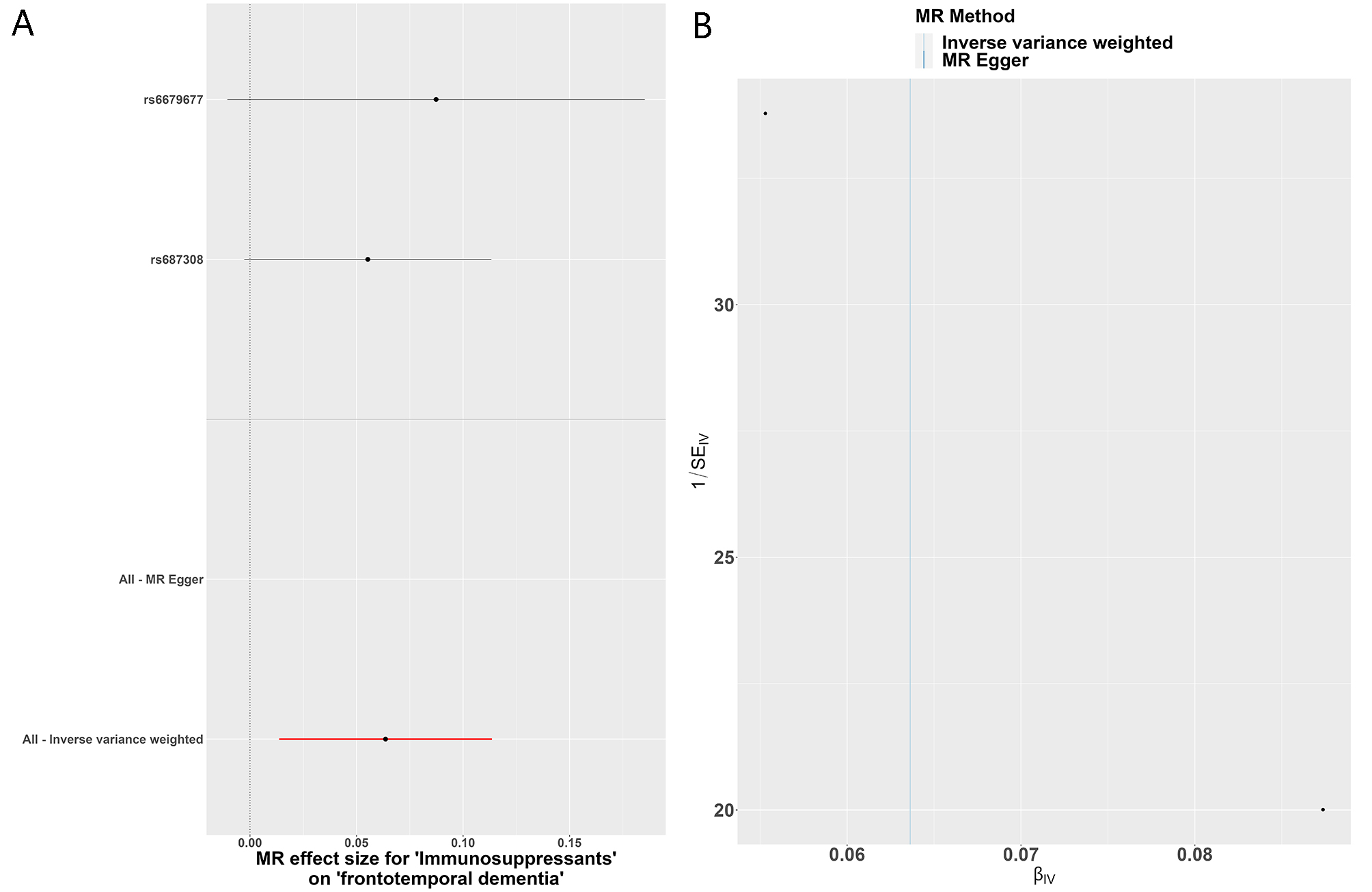

Supplement: Supplementary file 8 — Supporting Fig.8: The IVW analysis indicates that immunosuppressants are a risk factor for thyroid preparations: (A) forest plot; (B) funnel plot; (C) loo plot. [file BRB3-15-e71057-s001.jpg]

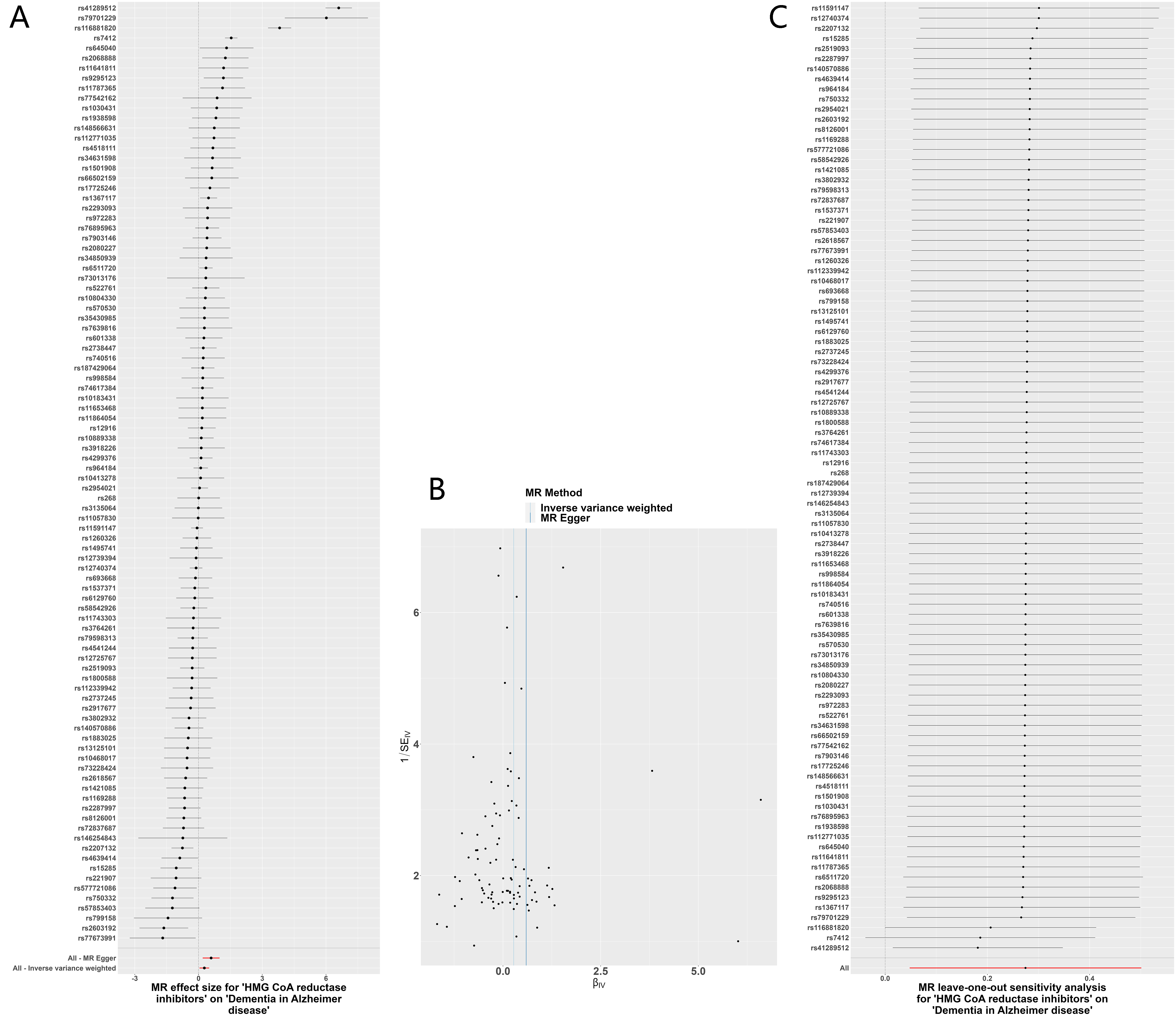

Supplement: Supplementary file 9 — Supporting Fig.9: The IVW analysis indicates that HMG CoA reductase inhibitors are a risk factor for dementia in Alzheimer's disease: (A) forest plot; (B) funnel plot; (C) loo plot. [file BRB3-15-e71057-s010.jpg]
