# Supplementary material for: A Causal Association Between Drug Use and Cognitive Impairment: A Two‐Sample Mendelian Randomization Study
Source: Brain Behav. 2025 Nov 11;15(11):e71057. doi: 10.1002/brb3.71057 (PMC12606049; doi:10.1002/brb3.71057)
Supplement: Supplementary file 10 — Supplementary Tables: brb371057‐sup‐0010‐tablesS1‐S24.docx [file BRB3-15-e71057-s006.docx]

**Table S1.** Selected SNPs and F values

| **Exposure factor** | **Outcome** | **Selected SNPs** | **F value (mean)** | **F value (min)** | **F value (max** | **SNPs that did not match** |
| --- | --- | --- | --- | --- | --- | --- |
| Drugs for peptic ulcer and gastro-oesophageal reflux disease (GORD) | Cognitive ability  & Cognitive impairment | 5 | 36.62 | 29.77 | 43.46 |  |
| Drugs used in diabetes |  | 57 | 63.13 | 29.74 | 657.9 |  |
| Antithrombotic agents |  | 13 | 59.89 | 32.28 | 87.06 |  |
| Vasodilators used in cardiac diseases |  | 3 | 62.92 | 40.96 | 75.0 |  |
| Antihypertensives |  | 4 | 33.3 | 31.64 | 34.43 |  |
| Diuretics |  | 102 | 51.65 | 29.89 | 149.58 |  |
| Beta blocking agents |  | 59 | 44.21 | 29.72 | 113.17 |  |
| Calcium channel blockers |  | 104 | 49.15 | 29.74 | 160.85 |  |
| Agents acting on the renin-angiotensin system |  | 184 | 51.83 | 29.7 | 313.46 |  |
| HMG CoA reductase inhibitors |  | 97 | 88.56 | 29.71 | 897.13 |  |
| Thyroid preparations |  | 132 | 77.78 | 29.82 | 773.67 |  |
| Immunosuppressants |  | 2 | 229.71 | 80.51 | 378.92 |  |
| Anti-inflammatory and antirheumatic products non-steroids. |  | 6 | 38.3 | 29.74 | 64.37 |  |
| Drugs affecting bone structure and mineralization |  | 11 | 36.87 | 29.71 | 62.19 |  |
| Opioids |  | 3 | 35.09 | 35.09 | 37.71 |  |
| Salicylic acid and derivatives |  | 10 | 48 | 29.77 | 29.77 |  |
| Anilides |  | 7 | 45.61 | 31.46 | 64.66 |  |
| Antimigraine preparations |  | 13 | 45.04 | 30.47 | 84.09 |  |
| Antidepressants |  | 1 | 34.32 | 34.32 | 34.32 |  |
| Adrenergics inhalants |  | 56 | 62.51 | 29.94 | 332.25 |  |
| Glucocorticoids |  | 19 | 67.17 | 33.87 | 214.12 |  |
| Antihistamines for systemic use |  | 8 | 38.13 | 33.87 | 55.35 |  |
| Antiglaucoma preparations and miotics |  | 14 | 52.88 | 29.79 | 173.55 |  |

**Table S2.** SNPs selection for adrenergic inhalants

|  | chr.exposure | SNP | pos.exposure | effect_allele.exposure | other_allele.exposure | eaf.exposure | beta.exposure | se.exposure | pval.exposure | exposure | id.exposure | samplesize.exposure | mr_keep.exposure | pval_origin.exposure | data_source.exposure | r2 | F |
| --- | --- | --- | --- | --- | --- | --- | --- | --- | --- | --- | --- | --- | --- | --- | --- | --- | --- |
| 1 | 6 | rs17843580 | 32615551 | A | G | 0,394094 | -0,17041 | 0,009349 | 3,1E-74 | Adrenergics inhalants | GCST007941 | 132367 | NA | NA | NA | 0,002504 | 332,2498 |
| 2 | 9 | rs992969 | 6209697 | A | G | 0,253772 | 0,135792 | 0,01038 | 4,2E-39 | Adrenergics inhalants | GCST007941 | 132367 | NA | NA | NA | 0,001291 | 171,134 |
| 3 | 2 | rs11679146 | 1,03E+08 | G | A | 0,135806 | -0,15992 | 0,01316 | 5,6E-34 | Adrenergics inhalants | GCST007941 | 132367 | NA | NA | NA | 0,001114 | 147,6664 |
| 4 | 10 | rs1775553 | 9054325 | T | C | 0,421285 | -0,10921 | 0,009118 | 4,7E-33 | Adrenergics inhalants | GCST007941 | 132367 | NA | NA | NA | 0,001083 | 143,4576 |
| 5 | 11 | rs7936312 | 76293726 | T | G | 0,479902 | 0,100179 | 0,009014 | 1,1E-28 | Adrenergics inhalants | GCST007941 | 132367 | NA | NA | NA | 0,000932 | 123,5028 |
| 6 | 2 | rs34290285 | 2,43E+08 | A | G | 0,253471 | -0,11348 | 0,010345 | 5,4E-28 | Adrenergics inhalants | GCST007941 | 132367 | NA | NA | NA | 0,000908 | 120,3294 |
| 7 | 15 | rs72743461 | 67441750 | A | C | 0,23821 | 0,110148 | 0,010571 | 2E-25 | Adrenergics inhalants | GCST007941 | 132367 | NA | NA | NA | 0,00082 | 108,5652 |
| 8 | 5 | rs1837253 | 1,1E+08 | T | C | 0,257988 | -0,10192 | 0,01029 | 4E-23 | Adrenergics inhalants | GCST007941 | 132367 | NA | NA | NA | 0,000741 | 98,11053 |
| 9 | 8 | rs2102418 | 81295744 | G | T | 0,365913 | 0,085786 | 0,009342 | 4,2E-20 | Adrenergics inhalants | GCST007941 | 132367 | NA | NA | NA | 0,000637 | 84,32953 |
| 10 | 17 | rs1011082 | 38068514 | C | T | 0,485127 | 0,082203 | 0,009001 | 6,7E-20 | Adrenergics inhalants | GCST007941 | 132367 | NA | NA | NA | 0,00063 | 83,40914 |
| 11 | 6 | rs1504215 | 91006227 | A | G | 0,349167 | -0,08325 | 0,009434 | 1,1E-18 | Adrenergics inhalants | GCST007941 | 132367 | NA | NA | NA | 0,000588 | 77,87279 |
| 12 | 19 | rs117710327 | 33726578 | A | C | 0,061602 | -0,1633 | 0,018929 | 6,3E-18 | Adrenergics inhalants | GCST007941 | 132367 | NA | NA | NA | 0,000562 | 74,42054 |
| 13 | 5 | rs115008099 | 1,32E+08 | T | C | 0,164643 | 0,105575 | 0,012315 | 1E-17 | Adrenergics inhalants | GCST007941 | 132367 | NA | NA | NA | 0,000555 | 73,49821 |
| 14 | 12 | rs34415530 | 56444632 | T | C | 0,342583 | 0,07985 | 0,009515 | 4,8E-17 | Adrenergics inhalants | GCST007941 | 132367 | NA | NA | NA | 0,000532 | 70,42964 |
| 15 | 12 | rs1059513 | 57489709 | C | T | 0,106698 | -0,12182 | 0,014568 | 6,2E-17 | Adrenergics inhalants | GCST007941 | 132367 | NA | NA | NA | 0,000528 | 69,92549 |
| 16 | 5 | rs7723819 | 1,1E+08 | A | G | 0,439866 | -0,073 | 0,009076 | 8,8E-16 | Adrenergics inhalants | GCST007941 | 132367 | NA | NA | NA | 0,000488 | 64,69118 |
| 17 | 16 | rs35441874 | 11213021 | A | T | 0,244249 | -0,08479 | 0,010549 | 9,1E-16 | Adrenergics inhalants | GCST007941 | 132367 | NA | NA | NA | 0,000488 | 64,60433 |
| 18 | 5 | rs2548992 | 1,32E+08 | A | G | 0,268078 | 0,080788 | 0,010184 | 2,1E-15 | Adrenergics inhalants | GCST007941 | 132367 | NA | NA | NA | 0,000475 | 62,92453 |
| 19 | 1 | rs12123821 | 1,52E+08 | T | C | 0,047278 | 0,164837 | 0,021156 | 6,6E-15 | Adrenergics inhalants | GCST007941 | 132367 | NA | NA | NA | 0,000458 | 60,7067 |
| 20 | 9 | rs2150968 | 6081298 | A | G | 0,309265 | -0,07604 | 0,009766 | 6,9E-15 | Adrenergics inhalants | GCST007941 | 132367 | NA | NA | NA | 0,000458 | 60,62344 |
| 21 | 4 | rs56293330 | 1,23E+08 | A | C | 0,074345 | 0,131546 | 0,017193 | 2E-14 | Adrenergics inhalants | GCST007941 | 132367 | NA | NA | NA | 0,000442 | 58,53769 |
| 22 | 1 | rs2949661 | 1,67E+08 | T | C | 0,401258 | -0,06964 | 0,009204 | 3,8E-14 | Adrenergics inhalants | GCST007941 | 132367 | NA | NA | NA | 0,000432 | 57,24548 |
| 23 | 16 | rs2239347 | 27359021 | C | A | 0,459344 | 0,067054 | 0,009039 | 1,2E-13 | Adrenergics inhalants | GCST007941 | 132367 | NA | NA | NA | 0,000416 | 55,03608 |
| 24 | 15 | rs11071559 | 61069988 | T | C | 0,128561 | -0,09462 | 0,013448 | 2E-12 | Adrenergics inhalants | GCST007941 | 132367 | NA | NA | NA | 0,000374 | 49,50691 |
| 25 | 5 | rs249681 | 1,42E+08 | G | C | 0,381436 | -0,06455 | 0,009305 | 4E-12 | Adrenergics inhalants | GCST007941 | 132367 | NA | NA | NA | 0,000363 | 48,12275 |
| 26 | 22 | rs139496 | 41639728 | T | G | 0,286408 | -0,06953 | 0,010056 | 4,7E-12 | Adrenergics inhalants | GCST007941 | 132367 | NA | NA | NA | 0,000361 | 47,80699 |
| 27 | 11 | rs174572 | 61598288 | T | C | 0,268763 | -0,06981 | 0,01017 | 6,7E-12 | Adrenergics inhalants | GCST007941 | 132367 | NA | NA | NA | 0,000356 | 47,11805 |
| 28 | 13 | rs7992229 | 1E+08 | G | A | 0,296065 | -0,06778 | 0,009874 | 6,7E-12 | Adrenergics inhalants | GCST007941 | 132367 | NA | NA | NA | 0,000356 | 47,1188 |
| 29 | 10 | rs2275806 | 8095340 | G | A | 0,427595 | 0,061035 | 0,009234 | 3,8E-11 | Adrenergics inhalants | GCST007941 | 132367 | NA | NA | NA | 0,00033 | 43,69343 |
| 30 | 17 | rs12952581 | 47448346 | A | G | 0,36465 | 0,061254 | 0,009373 | 6,4E-11 | Adrenergics inhalants | GCST007941 | 132367 | NA | NA | NA | 0,000323 | 42,70581 |
| 31 | 15 | rs2289790 | 67476970 | C | T | 0,237487 | -0,06938 | 0,010707 | 9,2E-11 | Adrenergics inhalants | GCST007941 | 132367 | NA | NA | NA | 0,000317 | 41,98616 |
| 32 | 5 | rs74455974 | 14572453 | T | A | 0,054195 | 0,130564 | 0,020287 | 1,2E-10 | Adrenergics inhalants | GCST007941 | 132367 | NA | NA | NA | 0,000313 | 41,41885 |
| 33 | 5 | rs13355228 | 1,57E+08 | T | C | 0,133402 | 0,084995 | 0,013264 | 1,5E-10 | Adrenergics inhalants | GCST007941 | 132367 | NA | NA | NA | 0,00031 | 41,05923 |
| 34 | 11 | rs12788104 | 1123739 | A | G | 0,310163 | -0,0616 | 0,009764 | 2,8E-10 | Adrenergics inhalants | GCST007941 | 132367 | NA | NA | NA | 0,000301 | 39,79637 |
| 35 | 7 | rs2390314 | 20455978 | A | T | 0,069144 | 0,112173 | 0,017791 | 2,9E-10 | Adrenergics inhalants | GCST007941 | 132367 | NA | NA | NA | 0,0003 | 39,75219 |
| 36 | 1 | rs159960 | 8476428 | A | G | 0,440566 | -0,05709 | 0,009148 | 4,4E-10 | Adrenergics inhalants | GCST007941 | 132367 | NA | NA | NA | 0,000294 | 38,94675 |
| 37 | 5 | rs4594881 | 35846815 | T | G | 0,339271 | -0,05842 | 0,009528 | 8,7E-10 | Adrenergics inhalants | GCST007941 | 132367 | NA | NA | NA | 0,000284 | 37,60023 |
| 38 | 3 | rs75366683 | 1,96E+08 | T | G | 0,065044 | -0,11008 | 0,018288 | 1,8E-09 | Adrenergics inhalants | GCST007941 | 132367 | NA | NA | NA | 0,000274 | 36,23145 |
| 39 | 3 | rs7428430 | 50174184 | T | C | 0,485019 | -0,05388 | 0,009023 | 2,4E-09 | Adrenergics inhalants | GCST007941 | 132367 | NA | NA | NA | 0,000269 | 35,65701 |
| 40 | 3 | rs35570272 | 33047662 | T | G | 0,395575 | 0,055107 | 0,009297 | 3,1E-09 | Adrenergics inhalants | GCST007941 | 132367 | NA | NA | NA | 0,000265 | 35,13412 |
| 41 | 21 | rs8128234 | 36470865 | T | C | 0,210674 | 0,065895 | 0,011131 | 3,2E-09 | Adrenergics inhalants | GCST007941 | 132367 | NA | NA | NA | 0,000265 | 35,04351 |
| 42 | 1 | rs2296618 | 1,99E+08 | G | A | 0,131595 | -0,07942 | 0,013428 | 3,3E-09 | Adrenergics inhalants | GCST007941 | 132367 | NA | NA | NA | 0,000264 | 34,98127 |
| 43 | 3 | rs60946162 | 1,88E+08 | T | C | 0,455369 | 0,05294 | 0,009086 | 5,7E-09 | Adrenergics inhalants | GCST007941 | 132367 | NA | NA | NA | 0,000256 | 33,94524 |
| 44 | 8 | rs6990534 | 1,29E+08 | A | G | 0,293571 | 0,057633 | 0,009901 | 5,9E-09 | Adrenergics inhalants | GCST007941 | 132367 | NA | NA | NA | 0,000256 | 33,88266 |
| 45 | 10 | rs2025758 | 8841669 | C | T | 0,454005 | -0,05292 | 0,0091 | 6E-09 | Adrenergics inhalants | GCST007941 | 132367 | NA | NA | NA | 0,000255 | 33,82166 |
| 46 | 10 | rs2497318 | 94432000 | T | C | 0,447177 | -0,05281 | 0,009095 | 6,4E-09 | Adrenergics inhalants | GCST007941 | 132367 | NA | NA | NA | 0,000255 | 33,7123 |
| 47 | 2 | rs10460475 | 1,2E+08 | G | T | 0,0937 | 0,089924 | 0,015528 | 7E-09 | Adrenergics inhalants | GCST007941 | 132367 | NA | NA | NA | 0,000253 | 33,53522 |
| 48 | 5 | rs7733410 | 1,48E+08 | A | G | 0,442033 | -0,05246 | 0,009065 | 7,2E-09 | Adrenergics inhalants | GCST007941 | 132367 | NA | NA | NA | 0,000253 | 33,49059 |
| 49 | 3 | rs7626218 | 1,77E+08 | T | A | 0,394521 | -0,05266 | 0,009248 | 1,2E-08 | Adrenergics inhalants | GCST007941 | 132367 | NA | NA | NA | 0,000245 | 32,42662 |
| 50 | 2 | rs7595769 | 8471319 | A | G | 0,280882 | -0,05696 | 0,01001 | 1,3E-08 | Adrenergics inhalants | GCST007941 | 132367 | NA | NA | NA | 0,000245 | 32,37496 |
| 51 | 5 | rs7735519 | 1,1E+08 | A | C | 0,147692 | 0,07197 | 0,012749 | 1,6E-08 | Adrenergics inhalants | GCST007941 | 132367 | NA | NA | NA | 0,000241 | 31,86956 |
| 52 | 12 | rs17226898 | 71585743 | C | T | 0,381266 | -0,05279 | 0,009379 | 1,8E-08 | Adrenergics inhalants | GCST007941 | 132367 | NA | NA | NA | 0,000239 | 31,67563 |
| 53 | 3 | rs6802894 | 1,69E+08 | G | T | 0,414885 | 0,051398 | 0,009175 | 2,1E-08 | Adrenergics inhalants | GCST007941 | 132367 | NA | NA | NA | 0,000237 | 31,37975 |
| 54 | 16 | rs11646066 | 27275248 | A | G | 0,015308 | -0,20486 | 0,036776 | 2,5E-08 | Adrenergics inhalants | GCST007941 | 132367 | NA | NA | NA | 0,000234 | 31,02973 |
| 55 | 9 | rs10814853 | 4144996 | T | C | 0,311041 | -0,05384 | 0,009759 | 3,4E-08 | Adrenergics inhalants | GCST007941 | 132367 | NA | NA | NA | 0,00023 | 30,43816 |
| 56 | 5 | rs17057868 | 1,6E+08 | C | T | 0,233336 | -0,05822 | 0,010639 | 4,4E-08 | Adrenergics inhalants | GCST007941 | 132367 | NA | NA | NA | 0,000226 | 29,94426 |

**Table S3.** SNPs selection for agents acting on the renin-angiotensin system

|  | chr.exposure | SNP | pos.exposure | effect_allele.exposure | other_allele.exposure | eaf.exposure | beta.exposure | se.exposure | pval.exposure | exposure | id.exposure | samplesize.exposure | mr_keep.exposure | pval_origin.exposure | data_source.exposure | r2 | F |
| --- | --- | --- | --- | --- | --- | --- | --- | --- | --- | --- | --- | --- | --- | --- | --- | --- | --- |
| 1 | 4 | rs13125101 | 81174592 | A | G | 0,2921 | 0,117786 | 0,006653 | 3,8E-70 | Agents acting on the renin-angiotensin system | GCST007930 | 132367 | NA | NA | NA | 0,002363 | 313,4585 |
| 2 | 7 | rs3918226 | 1,51E+08 | T | C | 0,0773 | 0,145321 | 0,011366 | 2E-37 | Agents acting on the renin-angiotensin system | GCST007930 | 132367 | NA | NA | NA | 0,001234 | 163,476 |
| 3 | 11 | rs604723 | 1,01E+08 | T | C | 0,271336 | -0,08634 | 0,006842 | 1,7E-36 | Agents acting on the renin-angiotensin system | GCST007930 | 132367 | NA | NA | NA | 0,001202 | 159,2338 |
| 4 | 15 | rs1894400 | 91428955 | T | C | 0,325947 | 0,076664 | 0,006427 | 8,4E-33 | Agents acting on the renin-angiotensin system | GCST007930 | 132367 | NA | NA | NA | 0,001074 | 142,2974 |
| 5 | 10 | rs57541197 | 63511306 | A | G | 0,144319 | -0,0963 | 0,008556 | 2,2E-29 | Agents acting on the renin-angiotensin system | GCST007930 | 132367 | NA | NA | NA | 0,000956 | 126,668 |
| 6 | 20 | rs6026739 | 57739469 | T | A | 0,11701 | 0,103486 | 0,009419 | 4,4E-28 | Agents acting on the renin-angiotensin system | GCST007930 | 132367 | NA | NA | NA | 0,000911 | 120,7077 |
| 7 | 11 | rs7938342 | 1887806 | T | A | 0,415375 | -0,06741 | 0,006136 | 4,5E-28 | Agents acting on the renin-angiotensin system | GCST007930 | 132367 | NA | NA | NA | 0,000911 | 120,6559 |
| 8 | 12 | rs7310615 | 1,12E+08 | C | G | 0,484183 | 0,065732 | 0,006074 | 2,7E-27 | Agents acting on the renin-angiotensin system | GCST007930 | 132367 | NA | NA | NA | 0,000884 | 117,1148 |
| 9 | 12 | rs35441 | 1,16E+08 | T | C | 0,380577 | -0,0662 | 0,006191 | 1,1E-26 | Agents acting on the renin-angiotensin system | GCST007930 | 132367 | NA | NA | NA | 0,000863 | 114,3089 |
| 10 | 2 | rs1731243 | 26930411 | C | T | 0,394283 | 0,064797 | 0,006168 | 8,1E-26 | Agents acting on the renin-angiotensin system | GCST007930 | 132367 | NA | NA | NA | 0,000833 | 110,366 |
| 11 | 6 | rs11752759 | 1,27E+08 | C | A | 0,438169 | 0,063558 | 0,00606 | 9,7E-26 | Agents acting on the renin-angiotensin system | GCST007930 | 132367 | NA | NA | NA | 0,00083 | 110,0118 |
| 12 | 19 | rs167479 | 11526765 | T | G | 0,471256 | -0,06187 | 0,006039 | 1,2E-24 | Agents acting on the renin-angiotensin system | GCST007930 | 132367 | NA | NA | NA | 0,000792 | 104,9812 |
| 13 | 1 | rs880315 | 10796866 | C | T | 0,339584 | 0,064731 | 0,006428 | 7,4E-24 | Agents acting on the renin-angiotensin system | GCST007930 | 132367 | NA | NA | NA | 0,000766 | 101,4185 |
| 14 | 1 | rs56153133 | 11885647 | G | A | 0,162373 | -0,08191 | 0,008149 | 9,1E-24 | Agents acting on the renin-angiotensin system | GCST007930 | 132367 | NA | NA | NA | 0,000763 | 101,0136 |
| 15 | 19 | rs12978472 | 7257990 | G | C | 0,127092 | -0,08938 | 0,00911 | 1E-22 | Agents acting on the renin-angiotensin system | GCST007930 | 132367 | NA | NA | NA | 0,000727 | 96,25778 |
| 16 | 16 | rs55872725 | 53809123 | T | C | 0,402662 | 0,058851 | 0,006134 | 8,4E-22 | Agents acting on the renin-angiotensin system | GCST007930 | 132367 | NA | NA | NA | 0,000695 | 92,05622 |
| 17 | 5 | rs13154066 | 32831670 | T | C | 0,403118 | -0,05872 | 0,00614 | 1,1E-21 | Agents acting on the renin-angiotensin system | GCST007930 | 132367 | NA | NA | NA | 0,00069 | 91,44664 |
| 18 | 15 | rs11072508 | 75062397 | C | T | 0,327501 | 0,060411 | 0,006463 | 9E-21 | Agents acting on the renin-angiotensin system | GCST007930 | 132367 | NA | NA | NA | 0,00066 | 87,3694 |
| 19 | 19 | rs7412 | 45412079 | T | C | 0,082017 | -0,103 | 0,01102 | 9E-21 | Agents acting on the renin-angiotensin system | GCST007930 | 132367 | NA | NA | NA | 0,00066 | 87,36513 |
| 20 | 20 | rs6039216 | 8622480 | C | T | 0,360894 | -0,05769 | 0,006284 | 4,3E-20 | Agents acting on the renin-angiotensin system | GCST007930 | 132367 | NA | NA | NA | 0,000636 | 84,28949 |
| 21 | 17 | rs4277405 | 61548918 | C | T | 0,375555 | 0,056869 | 0,006228 | 6,8E-20 | Agents acting on the renin-angiotensin system | GCST007930 | 132367 | NA | NA | NA | 0,00063 | 83,37939 |
| 22 | 1 | rs3790604 | 1,13E+08 | A | C | 0,072707 | 0,1031 | 0,011591 | 5,9E-19 | Agents acting on the renin-angiotensin system | GCST007930 | 132367 | NA | NA | NA | 0,000597 | 79,11437 |
| 23 | 3 | rs2643826 | 27562988 | T | C | 0,453083 | 0,053949 | 0,006073 | 6,5E-19 | Agents acting on the renin-angiotensin system | GCST007930 | 132367 | NA | NA | NA | 0,000596 | 78,92283 |
| 24 | 5 | rs7701003 | 1,58E+08 | G | A | 0,369354 | -0,05506 | 0,006235 | 1E-18 | Agents acting on the renin-angiotensin system | GCST007930 | 132367 | NA | NA | NA | 0,000589 | 77,96547 |
| 25 | 7 | rs3735533 | 27245893 | T | C | 0,073275 | -0,09908 | 0,011582 | 1,2E-17 | Agents acting on the renin-angiotensin system | GCST007930 | 132367 | NA | NA | NA | 0,000553 | 73,18194 |
| 26 | 9 | rs10217586 | 22121349 | A | T | 0,467959 | -0,05177 | 0,006058 | 1,3E-17 | Agents acting on the renin-angiotensin system | GCST007930 | 132367 | NA | NA | NA | 0,000551 | 73,02271 |
| 27 | 20 | rs8118848 | 62461572 | A | G | 0,236442 | -0,05972 | 0,007108 | 4,4E-17 | Agents acting on the renin-angiotensin system | GCST007930 | 132367 | NA | NA | NA | 0,000533 | 70,60787 |
| 28 | 1 | rs146754848 | 1,16E+08 | G | T | 0,017704 | 0,190722 | 0,022753 | 5,2E-17 | Agents acting on the renin-angiotensin system | GCST007930 | 132367 | NA | NA | NA | 0,000531 | 70,26357 |
| 29 | 16 | rs77924615 | 20392332 | A | G | 0,19261 | -0,06461 | 0,007734 | 6,6E-17 | Agents acting on the renin-angiotensin system | GCST007930 | 132367 | NA | NA | NA | 0,000527 | 69,78395 |
| 30 | 11 | rs415895 | 9769562 | C | G | 0,353354 | -0,05208 | 0,006301 | 1,4E-16 | Agents acting on the renin-angiotensin system | GCST007930 | 132367 | NA | NA | NA | 0,000516 | 68,31427 |
| 31 | 10 | rs79668541 | 1,05E+08 | T | C | 0,075918 | -0,09327 | 0,011365 | 2,3E-16 | Agents acting on the renin-angiotensin system | GCST007930 | 132367 | NA | NA | NA | 0,000509 | 67,35075 |
| 32 | 20 | rs1887320 | 10965998 | A | G | 0,476625 | 0,049325 | 0,006057 | 3,8E-16 | Agents acting on the renin-angiotensin system | GCST007930 | 132367 | NA | NA | NA | 0,000501 | 66,31557 |
| 33 | 10 | rs74157123 | 1,06E+08 | T | C | 0,010108 | 0,244794 | 0,030104 | 4,2E-16 | Agents acting on the renin-angiotensin system | GCST007930 | 132367 | NA | NA | NA | 0,000499 | 66,12396 |
| 34 | 7 | rs34748838 | 1,3E+08 | T | C | 0,489913 | -0,04805 | 0,006041 | 1,8E-15 | Agents acting on the renin-angiotensin system | GCST007930 | 132367 | NA | NA | NA | 0,000478 | 63,27867 |
| 35 | 16 | rs164749 | 89708224 | G | T | 0,430761 | -0,04841 | 0,006097 | 2E-15 | Agents acting on the renin-angiotensin system | GCST007930 | 132367 | NA | NA | NA | 0,000476 | 63,04056 |
| 36 | 7 | rs28451436 | 27319039 | A | G | 0,101625 | 0,078462 | 0,009976 | 3,7E-15 | Agents acting on the renin-angiotensin system | GCST007930 | 132367 | NA | NA | NA | 0,000467 | 61,86082 |
| 37 | 11 | rs7121869 | 16337827 | A | G | 0,206893 | 0,058297 | 0,007416 | 3,8E-15 | Agents acting on the renin-angiotensin system | GCST007930 | 132367 | NA | NA | NA | 0,000467 | 61,79334 |
| 38 | 6 | rs1799945 | 26091179 | G | C | 0,152431 | 0,065564 | 0,008364 | 4,6E-15 | Agents acting on the renin-angiotensin system | GCST007930 | 132367 | NA | NA | NA | 0,000464 | 61,4443 |
| 39 | 10 | rs7070847 | 18726054 | A | G | 0,283561 | -0,05249 | 0,006707 | 5E-15 | Agents acting on the renin-angiotensin system | GCST007930 | 132367 | NA | NA | NA | 0,000463 | 61,25532 |
| 40 | 17 | rs17637472 | 47461433 | A | G | 0,39989 | 0,048654 | 0,006254 | 7,3E-15 | Agents acting on the renin-angiotensin system | GCST007930 | 132367 | NA | NA | NA | 0,000457 | 60,52061 |
| 41 | 6 | rs55730499 | 1,61E+08 | T | C | 0,079514 | 0,08663 | 0,011135 | 7,3E-15 | Agents acting on the renin-angiotensin system | GCST007930 | 132367 | NA | NA | NA | 0,000457 | 60,52454 |
| 42 | 12 | rs11066309 | 1,13E+08 | A | G | 0,409764 | 0,047623 | 0,006143 | 9E-15 | Agents acting on the renin-angiotensin system | GCST007930 | 132367 | NA | NA | NA | 0,000454 | 60,10431 |
| 43 | 6 | rs57139556 | 1,51E+08 | G | A | 0,070976 | -0,09007 | 0,011714 | 1,5E-14 | Agents acting on the renin-angiotensin system | GCST007930 | 132367 | NA | NA | NA | 0,000446 | 59,11973 |
| 44 | 14 | rs72681869 | 50655357 | C | G | 0,010984 | -0,22207 | 0,028947 | 1,7E-14 | Agents acting on the renin-angiotensin system | GCST007930 | 132367 | NA | NA | NA | 0,000444 | 58,85606 |
| 45 | 12 | rs7299436 | 90113070 | G | T | 0,157439 | -0,06237 | 0,008289 | 5,3E-14 | Agents acting on the renin-angiotensin system | GCST007930 | 132367 | NA | NA | NA | 0,000428 | 56,62166 |
| 46 | 11 | rs2306363 | 65405600 | T | G | 0,205555 | -0,05549 | 0,007456 | 9,8E-14 | Agents acting on the renin-angiotensin system | GCST007930 | 132367 | NA | NA | NA | 0,000418 | 55,39906 |
| 47 | 4 | rs56329057 | 1,57E+08 | T | C | 0,183334 | -0,05748 | 0,007786 | 1,6E-13 | Agents acting on the renin-angiotensin system | GCST007930 | 132367 | NA | NA | NA | 0,000412 | 54,49717 |
| 48 | 10 | rs1801253 | 1,16E+08 | G | C | 0,259296 | -0,05095 | 0,006921 | 1,8E-13 | Agents acting on the renin-angiotensin system | GCST007930 | 132367 | NA | NA | NA | 0,000409 | 54,1803 |
| 49 | 17 | rs11874 | 45017193 | A | G | 0,136094 | 0,064689 | 0,008794 | 1,9E-13 | Agents acting on the renin-angiotensin system | GCST007930 | 132367 | NA | NA | NA | 0,000409 | 54,11025 |
| 50 | 3 | rs6800730 | 48174210 | A | G | 0,332275 | -0,04666 | 0,00638 | 2,6E-13 | Agents acting on the renin-angiotensin system | GCST007930 | 132367 | NA | NA | NA | 0,000404 | 53,47624 |
| 51 | 5 | rs11743404 | 1,28E+08 | C | T | 0,360119 | -0,04597 | 0,00629 | 2,7E-13 | Agents acting on the renin-angiotensin system | GCST007930 | 132367 | NA | NA | NA | 0,000403 | 53,41307 |
| 52 | 1 | rs4970834 | 1,1E+08 | T | C | 0,185684 | -0,05628 | 0,007777 | 4,6E-13 | Agents acting on the renin-angiotensin system | GCST007930 | 132367 | NA | NA | NA | 0,000395 | 52,36694 |
| 53 | 11 | rs2856653 | 47361084 | T | C | 0,358513 | 0,04566 | 0,006322 | 5,1E-13 | Agents acting on the renin-angiotensin system | GCST007930 | 132367 | NA | NA | NA | 0,000394 | 52,16776 |
| 54 | 19 | rs7246865 | 17219105 | A | G | 0,257107 | 0,050054 | 0,006965 | 6,7E-13 | Agents acting on the renin-angiotensin system | GCST007930 | 132367 | NA | NA | NA | 0,00039 | 51,64305 |
| 55 | 16 | rs55683214 | 81536191 | T | G | 0,253547 | -0,04968 | 0,006929 | 7,6E-13 | Agents acting on the renin-angiotensin system | GCST007930 | 132367 | NA | NA | NA | 0,000388 | 51,39104 |
| 56 | 8 | rs7460226 | 10197718 | G | A | 0,409371 | 0,044416 | 0,006197 | 7,6E-13 | Agents acting on the renin-angiotensin system | GCST007930 | 132367 | NA | NA | NA | 0,000388 | 51,37323 |
| 57 | 10 | rs2901761 | 95895127 | A | G | 0,418024 | 0,043702 | 0,006107 | 8,3E-13 | Agents acting on the renin-angiotensin system | GCST007930 | 132367 | NA | NA | NA | 0,000387 | 51,21745 |
| 58 | 8 | rs332040 | 8730488 | A | G | 0,456548 | 0,042917 | 0,006087 | 1,8E-12 | Agents acting on the renin-angiotensin system | GCST007930 | 132367 | NA | NA | NA | 0,000375 | 49,71318 |
| 59 | 11 | rs11229555 | 58408687 | T | G | 0,238421 | -0,04979 | 0,007075 | 1,9E-12 | Agents acting on the renin-angiotensin system | GCST007930 | 132367 | NA | NA | NA | 0,000374 | 49,53833 |
| 60 | 11 | rs4459340 | 1,12E+08 | G | A | 0,275503 | 0,047696 | 0,006776 | 1,9E-12 | Agents acting on the renin-angiotensin system | GCST007930 | 132367 | NA | NA | NA | 0,000374 | 49,5457 |
| 61 | 11 | rs964184 | 1,17E+08 | G | C | 0,131318 | 0,062594 | 0,008903 | 2,1E-12 | Agents acting on the renin-angiotensin system | GCST007930 | 132367 | NA | NA | NA | 0,000373 | 49,42958 |
| 62 | 4 | rs13118687 | 1,11E+08 | A | G | 0,470423 | -0,04266 | 0,006081 | 2,3E-12 | Agents acting on the renin-angiotensin system | GCST007930 | 132367 | NA | NA | NA | 0,000372 | 49,20827 |
| 63 | 18 | rs72915163 | 48792829 | T | C | 0,255546 | 0,048986 | 0,007012 | 2,8E-12 | Agents acting on the renin-angiotensin system | GCST007930 | 132367 | NA | NA | NA | 0,000369 | 48,80799 |
| 64 | 1 | rs61772578 | 56939235 | G | A | 0,110553 | 0,067211 | 0,00963 | 3E-12 | Agents acting on the renin-angiotensin system | GCST007930 | 132367 | NA | NA | NA | 0,000368 | 48,70744 |
| 65 | 4 | rs10213458 | 38366390 | A | G | 0,319084 | 0,044956 | 0,006448 | 3,1E-12 | Agents acting on the renin-angiotensin system | GCST007930 | 132367 | NA | NA | NA | 0,000367 | 48,60291 |
| 66 | 10 | rs2236295 | 64564892 | T | G | 0,402916 | -0,04305 | 0,006193 | 3,6E-12 | Agents acting on the renin-angiotensin system | GCST007930 | 132367 | NA | NA | NA | 0,000365 | 48,31748 |
| 67 | 4 | rs13112725 | 1,07E+08 | G | C | 0,2401 | -0,0488 | 0,007052 | 4,5E-12 | Agents acting on the renin-angiotensin system | GCST007930 | 132367 | NA | NA | NA | 0,000362 | 47,87747 |
| 68 | 13 | rs9506725 | 22314146 | C | T | 0,370015 | -0,04321 | 0,006247 | 4,6E-12 | Agents acting on the renin-angiotensin system | GCST007930 | 132367 | NA | NA | NA | 0,000361 | 47,8347 |
| 69 | 17 | rs11653468 | 47192620 | A | G | 0,234355 | -0,04902 | 0,007111 | 5,4E-12 | Agents acting on the renin-angiotensin system | GCST007930 | 132367 | NA | NA | NA | 0,000359 | 47,5284 |
| 70 | 5 | rs458356 | 55815892 | A | G | 0,236286 | -0,05071 | 0,007393 | 7E-12 | Agents acting on the renin-angiotensin system | GCST007930 | 132367 | NA | NA | NA | 0,000355 | 47,03771 |
| 71 | 12 | rs478863 | 69639207 | G | A | 0,274025 | 0,046254 | 0,006766 | 8,1E-12 | Agents acting on the renin-angiotensin system | GCST007930 | 132367 | NA | NA | NA | 0,000353 | 46,73068 |
| 72 | 1 | rs2493298 | 3325912 | A | C | 0,137877 | 0,058988 | 0,008758 | 1,6E-11 | Agents acting on the renin-angiotensin system | GCST007930 | 132367 | NA | NA | NA | 0,000343 | 45,3664 |
| 73 | 8 | rs67529638 | 76829893 | G | C | 0,348627 | -0,04279 | 0,006348 | 1,6E-11 | Agents acting on the renin-angiotensin system | GCST007930 | 132367 | NA | NA | NA | 0,000343 | 45,44453 |
| 74 | 4 | rs300934 | 1,44E+08 | T | G | 0,312643 | 0,043723 | 0,006509 | 1,9E-11 | Agents acting on the renin-angiotensin system | GCST007930 | 132367 | NA | NA | NA | 0,000341 | 45,1198 |
| 75 | 10 | rs12413195 | 18470329 | C | T | 0,235191 | 0,047622 | 0,007115 | 2,2E-11 | Agents acting on the renin-angiotensin system | GCST007930 | 132367 | NA | NA | NA | 0,000338 | 44,80216 |
| 76 | 7 | rs35301188 | 1,06E+08 | A | G | 0,169551 | 0,053643 | 0,008088 | 3,3E-11 | Agents acting on the renin-angiotensin system | GCST007930 | 132367 | NA | NA | NA | 0,000332 | 43,98394 |
| 77 | 12 | rs73075659 | 20373541 | G | A | 0,343772 | -0,04251 | 0,00641 | 3,3E-11 | Agents acting on the renin-angiotensin system | GCST007930 | 132367 | NA | NA | NA | 0,000332 | 43,9853 |
| 78 | 7 | rs10254101 | 1,51E+08 | T | C | 0,288171 | 0,04403 | 0,006642 | 3,4E-11 | Agents acting on the renin-angiotensin system | GCST007930 | 132367 | NA | NA | NA | 0,000332 | 43,94699 |
| 79 | 8 | rs11250157 | 11525435 | G | A | 0,497878 | -0,03987 | 0,006017 | 3,5E-11 | Agents acting on the renin-angiotensin system | GCST007930 | 132367 | NA | NA | NA | 0,000332 | 43,89516 |
| 80 | 1 | rs193084249 | 26987646 | G | A | 0,022233 | 0,135676 | 0,020481 | 3,5E-11 | Agents acting on the renin-angiotensin system | GCST007930 | 132367 | NA | NA | NA | 0,000331 | 43,88183 |
| 81 | 20 | rs6122248 | 62751120 | C | T | 0,35001 | 0,041885 | 0,006322 | 3,5E-11 | Agents acting on the renin-angiotensin system | GCST007930 | 132367 | NA | NA | NA | 0,000332 | 43,89855 |
| 82 | 7 | rs7803355 | 7221775 | T | C | 0,114798 | 0,062578 | 0,009447 | 3,5E-11 | Agents acting on the renin-angiotensin system | GCST007930 | 132367 | NA | NA | NA | 0,000331 | 43,874 |
| 83 | 3 | rs35593046 | 53553923 | T | G | 0,262947 | -0,04615 | 0,006982 | 3,9E-11 | Agents acting on the renin-angiotensin system | GCST007930 | 132367 | NA | NA | NA | 0,00033 | 43,68378 |
| 84 | 12 | rs7952935 | 20166268 | G | C | 0,270584 | -0,04472 | 0,006773 | 4E-11 | Agents acting on the renin-angiotensin system | GCST007930 | 132367 | NA | NA | NA | 0,000329 | 43,59231 |
| 85 | 1 | rs12731208 | 6676485 | G | T | 0,353303 | 0,041496 | 0,006299 | 4,5E-11 | Agents acting on the renin-angiotensin system | GCST007930 | 132367 | NA | NA | NA | 0,000328 | 43,39085 |
| 86 | 19 | rs2656918 | 4927531 | T | C | 0,297864 | 0,043434 | 0,006593 | 4,5E-11 | Agents acting on the renin-angiotensin system | GCST007930 | 132367 | NA | NA | NA | 0,000328 | 43,40081 |
| 87 | 9 | rs111820018 | 71709456 | A | G | 0,096091 | 0,067283 | 0,010229 | 4,8E-11 | Agents acting on the renin-angiotensin system | GCST007930 | 132367 | NA | NA | NA | 0,000327 | 43,26398 |
| 88 | 5 | rs2984644 | 1,57E+08 | A | G | 0,269037 | 0,044591 | 0,006798 | 5,4E-11 | Agents acting on the renin-angiotensin system | GCST007930 | 132367 | NA | NA | NA | 0,000325 | 43,01997 |
| 89 | 4 | rs4691666 | 1,56E+08 | C | T | 0,466701 | 0,039504 | 0,006046 | 6,4E-11 | Agents acting on the renin-angiotensin system | GCST007930 | 132367 | NA | NA | NA | 0,000322 | 42,69515 |
| 90 | 17 | rs12453576 | 3881293 | T | C | 0,177701 | 0,051418 | 0,007874 | 6,6E-11 | Agents acting on the renin-angiotensin system | GCST007930 | 132367 | NA | NA | NA | 0,000322 | 42,63893 |
| 91 | 6 | rs2105092 | 1,34E+08 | A | G | 0,28919 | -0,04321 | 0,006663 | 8,9E-11 | Agents acting on the renin-angiotensin system | GCST007930 | 132367 | NA | NA | NA | 0,000318 | 42,04858 |
| 92 | 2 | rs268263 | 1,65E+08 | T | A | 0,244312 | -0,04583 | 0,007088 | 1E-10 | Agents acting on the renin-angiotensin system | GCST007930 | 132367 | NA | NA | NA | 0,000316 | 41,80471 |
| 93 | 13 | rs1006096 | 32189143 | T | C | 0,434974 | -0,03933 | 0,006097 | 1,1E-10 | Agents acting on the renin-angiotensin system | GCST007930 | 132367 | NA | NA | NA | 0,000314 | 41,59992 |
| 94 | 3 | rs6766859 | 1,38E+08 | C | T | 0,371212 | 0,040738 | 0,006307 | 1,1E-10 | Agents acting on the renin-angiotensin system | GCST007930 | 132367 | NA | NA | NA | 0,000315 | 41,72133 |
| 95 | 3 | rs6445597 | 53734531 | A | G | 0,321786 | 0,041901 | 0,006504 | 1,2E-10 | Agents acting on the renin-angiotensin system | GCST007930 | 132367 | NA | NA | NA | 0,000313 | 41,5061 |
| 96 | 15 | rs1757463 | 41783814 | G | A | 0,284522 | 0,042849 | 0,006747 | 2,1E-10 | Agents acting on the renin-angiotensin system | GCST007930 | 132367 | NA | NA | NA | 0,000305 | 40,33475 |
| 97 | 11 | rs11212104 | 1,07E+08 | G | A | 0,435024 | -0,03856 | 0,006078 | 2,2E-10 | Agents acting on the renin-angiotensin system | GCST007930 | 132367 | NA | NA | NA | 0,000304 | 40,2458 |
| 98 | 1 | rs2760061 | 2,28E+08 | A | T | 0,481262 | 0,03879 | 0,006155 | 2,9E-10 | Agents acting on the renin-angiotensin system | GCST007930 | 132367 | NA | NA | NA | 0,0003 | 39,71922 |
| 99 | 5 | rs7716114 | 1,42E+08 | G | A | 0,287064 | -0,04247 | 0,006744 | 3E-10 | Agents acting on the renin-angiotensin system | GCST007930 | 132367 | NA | NA | NA | 0,000299 | 39,64897 |
| 100 | 3 | rs1290786 | 1,69E+08 | T | C | 0,429711 | 0,038311 | 0,00609 | 3,2E-10 | Agents acting on the renin-angiotensin system | GCST007930 | 132367 | NA | NA | NA | 0,000299 | 39,57746 |
| 101 | 4 | rs28667801 | 26785356 | T | A | 0,406024 | 0,038929 | 0,006206 | 3,5E-10 | Agents acting on the renin-angiotensin system | GCST007930 | 132367 | NA | NA | NA | 0,000297 | 39,3477 |
| 102 | 7 | rs1722883 | 1,34E+08 | C | T | 0,47102 | -0,03794 | 0,006061 | 3,9E-10 | Agents acting on the renin-angiotensin system | GCST007930 | 132367 | NA | NA | NA | 0,000296 | 39,18202 |
| 103 | 4 | rs13107325 | 1,03E+08 | T | C | 0,07458 | -0,07159 | 0,011466 | 4,3E-10 | Agents acting on the renin-angiotensin system | GCST007930 | 132367 | NA | NA | NA | 0,000294 | 38,98841 |
| 104 | 9 | rs10817003 | 1,13E+08 | A | G | 0,035526 | 0,101509 | 0,016295 | 4,7E-10 | Agents acting on the renin-angiotensin system | GCST007930 | 132367 | NA | NA | NA | 0,000293 | 38,80446 |
| 105 | 7 | rs13230181 | 2537866 | A | G | 0,236028 | 0,044131 | 0,007091 | 4,9E-10 | Agents acting on the renin-angiotensin system | GCST007930 | 132367 | NA | NA | NA | 0,000293 | 38,73266 |
| 106 | 20 | rs6031431 | 42795152 | G | A | 0,461195 | 0,038108 | 0,006128 | 5E-10 | Agents acting on the renin-angiotensin system | GCST007930 | 132367 | NA | NA | NA | 0,000292 | 38,66807 |
| 107 | 17 | rs62059712 | 7740170 | C | T | 0,080321 | -0,06931 | 0,011158 | 5,3E-10 | Agents acting on the renin-angiotensin system | GCST007930 | 132367 | NA | NA | NA | 0,000291 | 38,57805 |
| 108 | 12 | rs28520411 | 1,22E+08 | A | G | 0,459707 | -0,03757 | 0,006057 | 5,5E-10 | Agents acting on the renin-angiotensin system | GCST007930 | 132367 | NA | NA | NA | 0,000291 | 38,48371 |
| 109 | 11 | rs3802932 | 63988045 | A | G | 0,05766 | 0,080054 | 0,012918 | 5,7E-10 | Agents acting on the renin-angiotensin system | GCST007930 | 132367 | NA | NA | NA | 0,00029 | 38,40366 |
| 110 | 6 | rs2655438 | 22418441 | C | G | 0,295517 | -0,04077 | 0,006584 | 5,9E-10 | Agents acting on the renin-angiotensin system | GCST007930 | 132367 | NA | NA | NA | 0,00029 | 38,34786 |
| 111 | 5 | rs28081 | 96092990 | A | G | 0,150332 | -0,05214 | 0,008431 | 6,2E-10 | Agents acting on the renin-angiotensin system | GCST007930 | 132367 | NA | NA | NA | 0,000289 | 38,24361 |
| 112 | 8 | rs73563812 | 25900405 | T | G | 0,23594 | -0,04388 | 0,007101 | 6,5E-10 | Agents acting on the renin-angiotensin system | GCST007930 | 132367 | NA | NA | NA | 0,000288 | 38,17625 |
| 113 | 2 | rs76217384 | 1,44E+08 | G | A | 0,231528 | 0,043917 | 0,007116 | 6,7E-10 | Agents acting on the renin-angiotensin system | GCST007930 | 132367 | NA | NA | NA | 0,000288 | 38,09309 |
| 114 | 13 | rs3803266 | 30154349 | G | C | 0,231717 | 0,044119 | 0,007159 | 7,2E-10 | Agents acting on the renin-angiotensin system | GCST007930 | 132367 | NA | NA | NA | 0,000287 | 37,97747 |
| 115 | 19 | rs4804517 | 7406066 | G | A | 0,333729 | -0,03927 | 0,006388 | 7,9E-10 | Agents acting on the renin-angiotensin system | GCST007930 | 132367 | NA | NA | NA | 0,000285 | 37,78422 |
| 116 | 11 | rs751984 | 61278246 | C | T | 0,112856 | -0,05871 | 0,009557 | 8,1E-10 | Agents acting on the renin-angiotensin system | GCST007930 | 132367 | NA | NA | NA | 0,000285 | 37,74119 |
| 117 | 2 | rs2943660 | 2,27E+08 | T | G | 0,363073 | -0,03829 | 0,006246 | 8,8E-10 | Agents acting on the renin-angiotensin system | GCST007930 | 132367 | NA | NA | NA | 0,000284 | 37,5789 |
| 118 | 1 | rs2478543 | 2,31E+08 | C | T | 0,403731 | 0,037725 | 0,006156 | 8,9E-10 | Agents acting on the renin-angiotensin system | GCST007930 | 132367 | NA | NA | NA | 0,000284 | 37,55185 |
| 119 | 15 | rs7174222 | 81018543 | T | C | 0,468424 | 0,037073 | 0,006064 | 9,7E-10 | Agents acting on the renin-angiotensin system | GCST007930 | 132367 | NA | NA | NA | 0,000282 | 37,37574 |
| 120 | 6 | rs13205180 | 51832494 | T | C | 0,493653 | 0,036775 | 0,006039 | 1,1E-09 | Agents acting on the renin-angiotensin system | GCST007930 | 132367 | NA | NA | NA | 0,00028 | 37,08306 |
| 121 | 5 | rs1422278 | 1,22E+08 | T | G | 0,131869 | 0,053985 | 0,008887 | 1,2E-09 | Agents acting on the renin-angiotensin system | GCST007930 | 132367 | NA | NA | NA | 0,000279 | 36,89953 |
| 122 | 2 | rs7368883 | 43378720 | A | G | 0,36012 | 0,038085 | 0,006284 | 1,4E-09 | Agents acting on the renin-angiotensin system | GCST007930 | 132367 | NA | NA | NA | 0,000277 | 36,73046 |
| 123 | 20 | rs6076983 | 6331376 | C | T | 0,398614 | -0,03713 | 0,006157 | 1,6E-09 | Agents acting on the renin-angiotensin system | GCST007930 | 132367 | NA | NA | NA | 0,000275 | 36,37704 |
| 124 | 17 | rs11079428 | 59466701 | T | A | 0,223194 | -0,04425 | 0,007351 | 1,8E-09 | Agents acting on the renin-angiotensin system | GCST007930 | 132367 | NA | NA | NA | 0,000274 | 36,23279 |
| 125 | 12 | rs10876531 | 54443718 | C | A | 0,286877 | -0,0403 | 0,006721 | 2E-09 | Agents acting on the renin-angiotensin system | GCST007930 | 132367 | NA | NA | NA | 0,000271 | 35,94565 |
| 126 | 3 | rs77463690 | 1,69E+08 | G | A | 0,072232 | -0,06993 | 0,011657 | 2E-09 | Agents acting on the renin-angiotensin system | GCST007930 | 132367 | NA | NA | NA | 0,000272 | 35,98177 |
| 127 | 11 | rs10839204 | 49002247 | T | C | 0,139222 | -0,05212 | 0,008703 | 2,1E-09 | Agents acting on the renin-angiotensin system | GCST007930 | 132367 | NA | NA | NA | 0,000271 | 35,86354 |
| 128 | 6 | rs9394951 | 43350753 | C | T | 0,434424 | 0,036298 | 0,006063 | 2,1E-09 | Agents acting on the renin-angiotensin system | GCST007930 | 132367 | NA | NA | NA | 0,000271 | 35,84394 |
| 129 | 5 | rs3814424 | 87968953 | T | C | 0,157566 | 0,0494 | 0,008257 | 2,2E-09 | Agents acting on the renin-angiotensin system | GCST007930 | 132367 | NA | NA | NA | 0,00027 | 35,792 |
| 130 | 21 | rs762395 | 44769676 | A | G | 0,335664 | -0,03852 | 0,006441 | 2,2E-09 | Agents acting on the renin-angiotensin system | GCST007930 | 132367 | NA | NA | NA | 0,00027 | 35,76457 |
| 131 | 17 | rs9897348 | 1320113 | C | A | 0,395725 | -0,03704 | 0,006195 | 2,2E-09 | Agents acting on the renin-angiotensin system | GCST007930 | 132367 | NA | NA | NA | 0,00027 | 35,74372 |
| 132 | 7 | rs11556924 | 1,3E+08 | T | C | 0,389132 | -0,03651 | 0,006178 | 3,4E-09 | Agents acting on the renin-angiotensin system | GCST007930 | 132367 | NA | NA | NA | 0,000264 | 34,92699 |
| 133 | 4 | rs139051778 | 1,11E+08 | G | A | 0,011974 | 0,16302 | 0,027613 | 3,6E-09 | Agents acting on the renin-angiotensin system | GCST007930 | 132367 | NA | NA | NA | 0,000263 | 34,8524 |
| 134 | 20 | rs6068117 | 50889988 | G | A | 0,286206 | 0,039529 | 0,00671 | 3,8E-09 | Agents acting on the renin-angiotensin system | GCST007930 | 132367 | NA | NA | NA | 0,000262 | 34,70502 |
| 135 | 6 | rs140570886 | 1,61E+08 | C | T | 0,014863 | 0,146842 | 0,024957 | 4E-09 | Agents acting on the renin-angiotensin system | GCST007930 | 132367 | NA | NA | NA | 0,000261 | 34,61795 |
| 136 | 4 | rs9683944 | 1,38E+08 | G | A | 0,204222 | 0,043979 | 0,007482 | 4,1E-09 | Agents acting on the renin-angiotensin system | GCST007930 | 132367 | NA | NA | NA | 0,000261 | 34,55378 |
| 137 | 2 | rs1861410 | 58933591 | C | T | 0,444736 | 0,035802 | 0,006104 | 4,5E-09 | Agents acting on the renin-angiotensin system | GCST007930 | 132367 | NA | NA | NA | 0,00026 | 34,4018 |
| 138 | 3 | rs1000010 | 11604119 | G | A | 0,351806 | -0,03724 | 0,006387 | 5,5E-09 | Agents acting on the renin-angiotensin system | GCST007930 | 132367 | NA | NA | NA | 0,000257 | 33,99961 |
| 139 | 2 | rs115262049 | 43196694 | T | A | 0,086458 | -0,06246 | 0,010763 | 6,5E-09 | Agents acting on the renin-angiotensin system | GCST007930 | 132367 | NA | NA | NA | 0,000254 | 33,68236 |
| 140 | 4 | rs13130741 | 1,04E+08 | T | C | 0,485206 | -0,03498 | 0,006037 | 6,9E-09 | Agents acting on the renin-angiotensin system | GCST007930 | 132367 | NA | NA | NA | 0,000254 | 33,56968 |
| 141 | 5 | rs36071027 | 1,58E+08 | T | C | 0,357521 | -0,03641 | 0,006291 | 7,1E-09 | Agents acting on the renin-angiotensin system | GCST007930 | 132367 | NA | NA | NA | 0,000253 | 33,50769 |
| 142 | 3 | rs9844972 | 1,5E+08 | C | G | 0,06711 | 0,070061 | 0,012144 | 8E-09 | Agents acting on the renin-angiotensin system | GCST007930 | 132367 | NA | NA | NA | 0,000251 | 33,28412 |
| 143 | 3 | rs6445821 | 56754233 | A | C | 0,321349 | -0,03717 | 0,006458 | 8,6E-09 | Agents acting on the renin-angiotensin system | GCST007930 | 132367 | NA | NA | NA | 0,00025 | 33,1389 |
| 144 | 16 | rs9939774 | 30068354 | T | C | 0,405631 | 0,035333 | 0,006142 | 8,8E-09 | Agents acting on the renin-angiotensin system | GCST007930 | 132367 | NA | NA | NA | 0,00025 | 33,0889 |
| 145 | 11 | rs56175671 | 26163506 | C | A | 0,091793 | 0,060369 | 0,010504 | 9,1E-09 | Agents acting on the renin-angiotensin system | GCST007930 | 132367 | NA | NA | NA | 0,000249 | 33,02879 |
| 146 | 16 | rs62039768 | 51560761 | A | C | 0,095796 | 0,059182 | 0,010312 | 9,5E-09 | Agents acting on the renin-angiotensin system | GCST007930 | 132367 | NA | NA | NA | 0,000249 | 32,93949 |
| 147 | 12 | rs7962222 | 1,16E+08 | C | T | 0,35159 | -0,03626 | 0,006321 | 9,7E-09 | Agents acting on the renin-angiotensin system | GCST007930 | 132367 | NA | NA | NA | 0,000249 | 32,90784 |
| 148 | 6 | rs3857599 | 50938247 | A | C | 0,163118 | 0,046682 | 0,008142 | 9,8E-09 | Agents acting on the renin-angiotensin system | GCST007930 | 132367 | NA | NA | NA | 0,000248 | 32,87273 |
| 149 | 21 | rs2229742 | 16339172 | C | G | 0,103481 | 0,056737 | 0,009906 | 1E-08 | Agents acting on the renin-angiotensin system | GCST007930 | 132367 | NA | NA | NA | 0,000248 | 32,804 |
| 150 | 8 | rs12549801 | 1,42E+08 | A | G | 0,233986 | -0,04076 | 0,007147 | 1,2E-08 | Agents acting on the renin-angiotensin system | GCST007930 | 132367 | NA | NA | NA | 0,000246 | 32,52966 |
| 151 | 4 | rs62328145 | 1,57E+08 | A | G | 0,051741 | 0,078751 | 0,013826 | 1,2E-08 | Agents acting on the renin-angiotensin system | GCST007930 | 132367 | NA | NA | NA | 0,000245 | 32,442 |
| 152 | 17 | rs74439044 | 7781019 | C | T | 0,097249 | 0,057803 | 0,010142 | 1,2E-08 | Agents acting on the renin-angiotensin system | GCST007930 | 132367 | NA | NA | NA | 0,000245 | 32,48154 |
| 153 | 21 | rs76346476 | 44720384 | A | G | 0,037629 | 0,090309 | 0,015857 | 1,2E-08 | Agents acting on the renin-angiotensin system | GCST007930 | 132367 | NA | NA | NA | 0,000245 | 32,43535 |
| 154 | 12 | rs1557848 | 48181374 | G | A | 0,135722 | 0,05075 | 0,008922 | 1,3E-08 | Agents acting on the renin-angiotensin system | GCST007930 | 132367 | NA | NA | NA | 0,000244 | 32,35151 |
| 155 | 19 | rs516246 | 49206172 | C | T | 0,490532 | -0,0344 | 0,00605 | 1,3E-08 | Agents acting on the renin-angiotensin system | GCST007930 | 132367 | NA | NA | NA | 0,000244 | 32,31887 |
| 156 | 2 | rs1486195 | 1,09E+08 | G | A | 0,38746 | 0,035349 | 0,006231 | 1,4E-08 | Agents acting on the renin-angiotensin system | GCST007930 | 132367 | NA | NA | NA | 0,000243 | 32,18834 |
| 157 | 6 | rs9472136 | 43810021 | T | C | 0,398083 | -0,0352 | 0,006208 | 1,4E-08 | Agents acting on the renin-angiotensin system | GCST007930 | 132367 | NA | NA | NA | 0,000243 | 32,16158 |
| 158 | 16 | rs4888411 | 75443183 | T | A | 0,404043 | -0,03478 | 0,006146 | 1,5E-08 | Agents acting on the renin-angiotensin system | GCST007930 | 132367 | NA | NA | NA | 0,000242 | 32,02105 |
| 159 | 1 | rs10218741 | 2,21E+08 | G | A | 0,410449 | -0,03449 | 0,006108 | 1,6E-08 | Agents acting on the renin-angiotensin system | GCST007930 | 132367 | NA | NA | NA | 0,000241 | 31,88996 |
| 160 | 5 | rs1694068 | 53283630 | T | A | 0,382275 | -0,0351 | 0,006213 | 1,6E-08 | Agents acting on the renin-angiotensin system | GCST007930 | 132367 | NA | NA | NA | 0,000241 | 31,90319 |
| 161 | 7 | rs78745308 | 6404258 | G | C | 0,044988 | 0,081933 | 0,014507 | 1,6E-08 | Agents acting on the renin-angiotensin system | GCST007930 | 132367 | NA | NA | NA | 0,000241 | 31,89821 |
| 162 | 7 | rs2107595 | 19049388 | A | G | 0,151612 | 0,04741 | 0,008408 | 1,7E-08 | Agents acting on the renin-angiotensin system | GCST007930 | 132367 | NA | NA | NA | 0,00024 | 31,79658 |
| 163 | 11 | rs7483826 | 32527238 | G | A | 0,487817 | 0,034208 | 0,006062 | 1,7E-08 | Agents acting on the renin-angiotensin system | GCST007930 | 132367 | NA | NA | NA | 0,00024 | 31,84095 |
| 164 | 2 | rs10931284 | 1,88E+08 | C | T | 0,361893 | -0,03531 | 0,006275 | 1,8E-08 | Agents acting on the renin-angiotensin system | GCST007930 | 132367 | NA | NA | NA | 0,000239 | 31,66194 |
| 165 | 10 | rs12262629 | 60265656 | A | G | 0,336391 | -0,03609 | 0,00642 | 1,9E-08 | Agents acting on the renin-angiotensin system | GCST007930 | 132367 | NA | NA | NA | 0,000239 | 31,59291 |
| 166 | 11 | rs986721 | 13270717 | A | T | 0,27422 | -0,03797 | 0,006758 | 1,9E-08 | Agents acting on the renin-angiotensin system | GCST007930 | 132367 | NA | NA | NA | 0,000238 | 31,5758 |
| 167 | 8 | rs13280592 | 1,17E+08 | C | G | 0,270034 | -0,03842 | 0,006861 | 2,1E-08 | Agents acting on the renin-angiotensin system | GCST007930 | 132367 | NA | NA | NA | 0,000237 | 31,36075 |
| 168 | 14 | rs989501 | 71361414 | G | A | 0,351584 | -0,03543 | 0,006334 | 2,2E-08 | Agents acting on the renin-angiotensin system | GCST007930 | 132367 | NA | NA | NA | 0,000236 | 31,28758 |
| 169 | 19 | rs142158911 | 11190534 | A | G | 0,114529 | -0,05321 | 0,009519 | 2,3E-08 | Agents acting on the renin-angiotensin system | GCST007930 | 132367 | NA | NA | NA | 0,000236 | 31,2411 |
| 170 | 11 | rs7113478 | 46260223 | A | G | 0,373423 | 0,035193 | 0,006318 | 2,5E-08 | Agents acting on the renin-angiotensin system | GCST007930 | 132367 | NA | NA | NA | 0,000234 | 31,0248 |
| 171 | 12 | rs3819536 | 2436998 | G | A | 0,295117 | -0,03667 | 0,006601 | 2,8E-08 | Agents acting on the renin-angiotensin system | GCST007930 | 132367 | NA | NA | NA | 0,000233 | 30,8648 |
| 172 | 7 | rs73033340 | 1195692 | G | A | 0,031752 | -0,09538 | 0,01717 | 2,8E-08 | Agents acting on the renin-angiotensin system | GCST007930 | 132367 | NA | NA | NA | 0,000233 | 30,85644 |
| 173 | 4 | rs10155132 | 3369500 | A | G | 0,39295 | -0,03431 | 0,006187 | 2,9E-08 | Agents acting on the renin-angiotensin system | GCST007930 | 132367 | NA | NA | NA | 0,000232 | 30,75874 |
| 174 | 14 | rs28637873 | 24825677 | T | C | 0,077109 | -0,06273 | 0,011331 | 3,1E-08 | Agents acting on the renin-angiotensin system | GCST007930 | 132367 | NA | NA | NA | 0,000231 | 30,648 |
| 175 | 8 | rs2247355 | 1,04E+08 | T | C | 0,181421 | -0,04316 | 0,007826 | 3,5E-08 | Agents acting on the renin-angiotensin system | GCST007930 | 132367 | NA | NA | NA | 0,00023 | 30,41911 |
| 176 | 20 | rs6091153 | 49015311 | C | T | 0,228887 | 0,040122 | 0,007294 | 3,8E-08 | Agents acting on the renin-angiotensin system | GCST007930 | 132367 | NA | NA | NA | 0,000229 | 30,25827 |
| 177 | 17 | rs2680707 | 56455641 | T | C | 0,212003 | 0,040724 | 0,007416 | 4E-08 | Agents acting on the renin-angiotensin system | GCST007930 | 132367 | NA | NA | NA | 0,000228 | 30,15055 |
| 178 | 9 | rs563132 | 37245476 | T | A | 0,390895 | -0,03387 | 0,00617 | 4E-08 | Agents acting on the renin-angiotensin system | GCST007930 | 132367 | NA | NA | NA | 0,000228 | 30,14146 |
| 179 | 7 | rs75511781 | 1,31E+08 | G | A | 0,043338 | 0,081116 | 0,01477 | 4E-08 | Agents acting on the renin-angiotensin system | GCST007930 | 132367 | NA | NA | NA | 0,000228 | 30,15954 |
| 180 | 14 | rs7149242 | 1,01E+08 | T | G | 0,281233 | -0,03673 | 0,006697 | 4,1E-08 | Agents acting on the renin-angiotensin system | GCST007930 | 132367 | NA | NA | NA | 0,000227 | 30,07759 |
| 181 | 20 | rs6021247 | 50108980 | G | A | 0,471547 | -0,03307 | 0,006044 | 4,4E-08 | Agents acting on the renin-angiotensin system | GCST007930 | 132367 | NA | NA | NA | 0,000226 | 29,94326 |
| 182 | 2 | rs2252867 | 65296280 | C | T | 0,361669 | -0,03409 | 0,00625 | 4,9E-08 | Agents acting on the renin-angiotensin system | GCST007930 | 132367 | NA | NA | NA | 0,000225 | 29,75803 |
| 183 | 11 | rs7104561 | 55492764 | C | T | 0,099031 | 0,054953 | 0,010072 | 4,9E-08 | Agents acting on the renin-angiotensin system | GCST007930 | 132367 | NA | NA | NA | 0,000225 | 29,76686 |
| 184 | 3 | rs645040 | 1,36E+08 | G | T | 0,22559 | -0,03916 | 0,007186 | 5E-08 | Agents acting on the renin-angiotensin system | GCST007930 | 132367 | NA | NA | NA | 0,000224 | 29,70334 |

**Table S4.** SNPs selection for anilides

|  | chr.exposure | SNP | pos.exposure | effect_allele.exposure | other_allele.exposure | eaf.exposure | beta.exposure | se.exposure | pval.exposure | exposure | id.exposure | samplesize.exposure | mr_keep.exposure | pval_origin.exposure | data_source.exposure | r2 | F |
| --- | --- | --- | --- | --- | --- | --- | --- | --- | --- | --- | --- | --- | --- | --- | --- | --- | --- |
| 1 | 12 | rs11172113 | 57527283 | C | T | 0,409705 | -0,05341 | 0,006642 | 8,9E-16 | Anilides | GCST007938 | 132367 | NA | NA | NA | 0,000488 | 64,65982 |
| 2 | 6 | rs3130486 | 31722780 | T | C | 0,265164 | -0,05493 | 0,007387 | 1E-13 | Anilides | GCST007938 | 132367 | NA | NA | NA | 0,000418 | 55,29614 |
| 3 | 17 | rs17652520 | 44098967 | A | G | 0,231386 | 0,05571 | 0,007781 | 8,1E-13 | Anilides | GCST007938 | 132367 | NA | NA | NA | 0,000387 | 51,25759 |
| 4 | 2 | rs4663983 | 2,35E+08 | G | A | 0,191953 | -0,05578 | 0,008286 | 1,7E-11 | Anilides | GCST007938 | 132367 | NA | NA | NA | 0,000342 | 45,30805 |
| 5 | 1 | rs12568655 | 1,74E+08 | A | G | 0,388338 | -0,04073 | 0,006694 | 1,2E-09 | Anilides | GCST007938 | 132367 | NA | NA | NA | 0,00028 | 37,02714 |
| 6 | 6 | rs9368402 | 22125157 | T | C | 0,453647 | -0,03843 | 0,006565 | 4,8E-09 | Anilides | GCST007938 | 132367 | NA | NA | NA | 0,000259 | 34,26368 |
| 7 | 2 | rs7567892 | 2,42E+08 | T | C | 0,098818 | 0,061421 | 0,01095 | 2E-08 | Anilides | GCST007938 | 132367 | NA | NA | NA | 0,000238 | 31,46055 |

**Table S5.** SNPs selection for antidepressants

|  | chr.exposure | SNP | pos.exposure | effect_allele.exposure | other_allele.exposure | eaf.exposure | beta.exposure | se.exposure | pval.exposure | exposure | id.exposure | samplesize.exposure | mr_keep.exposure | pval_origin.exposure | data_source.exposure | r2 | F |
| --- | --- | --- | --- | --- | --- | --- | --- | --- | --- | --- | --- | --- | --- | --- | --- | --- | --- |
| 1 | 12 | rs10846305 | 16310816 | G | A | 0,391489 | 0,048607 | 0,008297 | 4,7E-09 | Antidepressants | GCST007940 | 132367 | NA | NA | NA | 0,000259 | 34,32179 |

**Table S6.** SNPs selection for antiglaucoma preparations and miotics

|  | chr.exposure | SNP | pos.exposure | effect_allele.exposure | other_allele.exposure | eaf.exposure | beta.exposure | se.exposure | pval.exposure | exposure | id.exposure | samplesize.exposure | mr_keep.exposure | pval_origin.exposure | data_source.exposure | r2 | F |
| --- | --- | --- | --- | --- | --- | --- | --- | --- | --- | --- | --- | --- | --- | --- | --- | --- | --- |
| 1 | 1 | rs2814471 | 1,66E+08 | C | T | 0,124329 | 0,398595 | 0,030256 | 1,2E-39 | Antiglaucoma preparations and miotics | GCST007944 | 132367 | NA | NA | NA | 0,001309 | 173,552 |
| 2 | 9 | rs2472493 | 1,08E+08 | G | A | 0,446503 | 0,170451 | 0,020464 | 8,1E-17 | Antiglaucoma preparations and miotics | GCST007944 | 132367 | NA | NA | NA | 0,000524 | 69,37381 |
| 3 | 17 | rs9913911 | 10031183 | G | A | 0,373894 | -0,17172 | 0,02073 | 1,2E-16 | Antiglaucoma preparations and miotics | GCST007944 | 132367 | NA | NA | NA | 0,000518 | 68,61697 |
| 4 | 9 | rs1360589 | 22045317 | C | T | 0,427984 | -0,14783 | 0,020099 | 1,9E-13 | Antiglaucoma preparations and miotics | GCST007944 | 132367 | NA | NA | NA | 0,000409 | 54,09498 |
| 5 | 14 | rs33912345 | 60976537 | C | A | 0,390214 | 0,145683 | 0,020479 | 1,1E-12 | Antiglaucoma preparations and miotics | GCST007944 | 132367 | NA | NA | NA | 0,000382 | 50,60569 |
| 6 | 9 | rs34186918 | 1,29E+08 | T | C | 0,238886 | 0,163547 | 0,023405 | 2,8E-12 | Antiglaucoma preparations and miotics | GCST007944 | 132367 | NA | NA | NA | 0,000369 | 48,82678 |
| 7 | 3 | rs6791035 | 85122909 | C | T | 0,406745 | 0,131386 | 0,020355 | 1,1E-10 | Antiglaucoma preparations and miotics | GCST007944 | 132367 | NA | NA | NA | 0,000315 | 41,66242 |
| 8 | 6 | rs2073006 | 637465 | T | C | 0,136887 | 0,177909 | 0,028929 | 7,8E-10 | Antiglaucoma preparations and miotics | GCST007944 | 132367 | NA | NA | NA | 0,000286 | 37,81906 |
| 9 | 8 | rs2507804 | 1,08E+08 | T | C | 0,135336 | -0,17396 | 0,029157 | 2,4E-09 | Antiglaucoma preparations and miotics | GCST007944 | 132367 | NA | NA | NA | 0,000269 | 35,5989 |
| 10 | 4 | rs28485535 | 7854170 | T | C | 0,226559 | 0,139597 | 0,023788 | 4,4E-09 | Antiglaucoma preparations and miotics | GCST007944 | 132367 | NA | NA | NA | 0,00026 | 34,4379 |
| 11 | 7 | rs1725452 | 11682722 | C | T | 0,42392 | 0,117862 | 0,020382 | 7,4E-09 | Antiglaucoma preparations and miotics | GCST007944 | 132367 | NA | NA | NA | 0,000253 | 33,43934 |
| 12 | 11 | rs111867185 | 47737549 | T | C | 0,133232 | 0,168181 | 0,029534 | 1,2E-08 | Antiglaucoma preparations and miotics | GCST007944 | 132367 | NA | NA | NA | 0,000245 | 32,42735 |
| 13 | 3 | rs11709963 | 1,69E+08 | C | T | 0,160763 | -0,14855 | 0,027089 | 4,2E-08 | Antiglaucoma preparations and miotics | GCST007944 | 132367 | NA | NA | NA | 0,000227 | 30,074 |
| 14 | 2 | rs706550 | 55902416 | A | G | 0,094039 | 0,186467 | 0,034164 | 4,8E-08 | Antiglaucoma preparations and miotics | GCST007944 | 132367 | NA | NA | NA | 0,000225 | 29,78979 |

**Table S7.** SNPs selection for antihistamines for systemic use

|  | chr.exposure | SNP | pos.exposure | effect_allele.exposure | other_allele.exposure | eaf.exposure | beta.exposure | se.exposure | pval.exposure | exposure | id.exposure | samplesize.exposure | mr_keep.exposure | pval_origin.exposure | data_source.exposure | r2 | F |
| --- | --- | --- | --- | --- | --- | --- | --- | --- | --- | --- | --- | --- | --- | --- | --- | --- | --- |
| 1 | 11 | rs11236797 | 76299649 | A | C | 0,453871 | 0,093666 | 0,012589 | 1E-13 | Antihistamines for systemic use | GCST007943 | 132367 | NA | NA | NA | 0,000418 | 55,35495 |
| 2 | 6 | rs28407950 | 32626348 | T | C | 0,243266 | -0,09466 | 0,014631 | 9,8E-11 | Antihistamines for systemic use | GCST007943 | 132367 | NA | NA | NA | 0,000316 | 41,86154 |
| 3 | 3 | rs1968514 | 29575263 | A | G | 0,057198 | -0,1654 | 0,026949 | 8,4E-10 | Antihistamines for systemic use | GCST007943 | 132367 | NA | NA | NA | 0,000285 | 37,6706 |
| 4 | 9 | rs2095044 | 6192796 | T | C | 0,257651 | 0,085321 | 0,014331 | 2,6E-09 | Antihistamines for systemic use | GCST007943 | 132367 | NA | NA | NA | 0,000268 | 35,44332 |
| 5 | 5 | rs6594499 | 1,1E+08 | A | C | 0,48439 | -0,07499 | 0,012596 | 2,6E-09 | Antihistamines for systemic use | GCST007943 | 132367 | NA | NA | NA | 0,000268 | 35,4412 |
| 6 | 2 | rs34290285 | 2,43E+08 | A | G | 0,25507 | -0,08337 | 0,014356 | 6,3E-09 | Antihistamines for systemic use | GCST007943 | 132367 | NA | NA | NA | 0,000255 | 33,72642 |
| 7 | 12 | rs1059513 | 57489709 | C | T | 0,107253 | -0,11708 | 0,020202 | 6,8E-09 | Antihistamines for systemic use | GCST007943 | 132367 | NA | NA | NA | 0,000254 | 33,58936 |
| 8 | 5 | rs10055042 | 1,1E+08 | A | G | 0,155523 | 0,098188 | 0,017361 | 1,6E-08 | Antihistamines for systemic use | GCST007943 | 132367 | NA | NA | NA | 0,000242 | 31,98513 |

**Table S8.** SNPs selection for antihypertensives

|  | chr.exposure | SNP | pos.exposure | effect_allele.exposure | other_allele.exposure | eaf.exposure | beta.exposure | se.exposure | pval.exposure | exposure | id.exposure | samplesize.exposure | mr_keep.exposure | pval_origin.exposure | data_source.exposure | r2 | F |
| --- | --- | --- | --- | --- | --- | --- | --- | --- | --- | --- | --- | --- | --- | --- | --- | --- | --- |
| 1 | 22 | rs758374 | 19971552 | C | T | 0,304289 | 0,113639 | 0,019366 | 4,4E-09 | Antihypertensives | GCST007926 | 132367 | NA | NA | NA | 0,00026 | 34,43207 |
| 2 | 11 | rs4980379 | 1888614 | T | C | 0,365232 | 0,107984 | 0,018611 | 6,6E-09 | Antihypertensives | GCST007926 | 132367 | NA | NA | NA | 0,000254 | 33,66304 |
| 3 | 1 | rs34071855 | 10798489 | G | C | 0,339889 | 0,110219 | 0,019059 | 7,3E-09 | Antihypertensives | GCST007926 | 132367 | NA | NA | NA | 0,000253 | 33,44221 |
| 4 | 2 | rs35021474 | 26916844 | C | G | 0,385032 | 0,103529 | 0,018404 | 1,9E-08 | Antihypertensives | GCST007926 | 132367 | NA | NA | NA | 0,000239 | 31,64461 |

**Table S9.** SNPs selection for antiinflammatroy and antirheumatic products non-steroids

|  | chr.exposure | SNP | pos.exposure | effect_allele.exposure | other_allele.exposure | eaf.exposure | beta.exposure | se.exposure | pval.exposure | exposure | id.exposure | samplesize.exposure | mr_keep.exposure | pval_origin.exposure | data_source.exposure | r2 | F |
| --- | --- | --- | --- | --- | --- | --- | --- | --- | --- | --- | --- | --- | --- | --- | --- | --- | --- |
| 1 | 12 | rs3001426 | 57509055 | C | T | 0,452847 | -0,05619 | 0,007004 | 1E-15 | Antiinflammatroy and antirheumatic products non-steroids | GCST007934 | 132367 | NA | NA | NA | 0,000486 | 64,37349 |
| 2 | 6 | rs2517611 | 30169327 | G | A | 0,229963 | -0,05048 | 0,008191 | 7,1E-10 | Antiinflammatroy and antirheumatic products non-steroids | GCST007934 | 132367 | NA | NA | NA | 0,000287 | 37,99111 |
| 3 | 5 | rs12522598 | 1,2E+08 | G | A | 0,288625 | 0,04522 | 0,007622 | 3E-09 | Antiinflammatroy and antirheumatic products non-steroids | GCST007934 | 132367 | NA | NA | NA | 0,000266 | 35,20084 |
| 4 | 4 | rs56166763 | 1,41E+08 | C | G | 0,368625 | -0,04085 | 0,007163 | 1,2E-08 | Antiinflammatroy and antirheumatic products non-steroids | GCST007934 | 132367 | NA | NA | NA | 0,000246 | 32,53133 |
| 5 | 15 | rs34862454 | 75101530 | C | T | 0,329747 | -0,0402 | 0,007343 | 4,4E-08 | Antiinflammatroy and antirheumatic products non-steroids | GCST007934 | 132367 | NA | NA | NA | 0,000226 | 29,96999 |
| 6 | 5 | rs6891880 | 1,5E+08 | G | A | 0,42715 | -0,03849 | 0,007058 | 4,9E-08 | Antiinflammatroy and antirheumatic products non-steroids | GCST007934 | 132367 | NA | NA | NA | 0,000225 | 29,74414 |

**Table S10.** SNPs selection for antimigraine preparations

|  | chr.exposure | SNP | pos.exposure | effect_allele.exposure | other_allele.exposure | eaf.exposure | beta.exposure | se.exposure | pval.exposure | exposure | id.exposure | samplesize.exposure | mr_keep.exposure | pval_origin.exposure | data_source.exposure | r2 | F |
| --- | --- | --- | --- | --- | --- | --- | --- | --- | --- | --- | --- | --- | --- | --- | --- | --- | --- |
| 1 | 6 | rs9349379 | 12903957 | G | A | 0,402462 | -0,18023 | 0,019655 | 4,7E-20 | Antimigraine preparations | GCST007939 | 132367 | NA | NA | NA | 0,000635 | 84,08733 |
| 2 | 2 | rs4663983 | 2,35E+08 | G | A | 0,193803 | -0,18898 | 0,02439 | 9,3E-15 | Antimigraine preparations | GCST007939 | 132367 | NA | NA | NA | 0,000453 | 60,0352 |
| 3 | 6 | rs7770889 | 96858453 | T | C | 0,347302 | 0,150991 | 0,020326 | 1,1E-13 | Antimigraine preparations | GCST007939 | 132367 | NA | NA | NA | 0,000417 | 55,18073 |
| 4 | 12 | rs11172113 | 57527283 | C | T | 0,411039 | -0,1435 | 0,019624 | 2,6E-13 | Antimigraine preparations | GCST007939 | 132367 | NA | NA | NA | 0,000404 | 53,47597 |
| 5 | 9 | rs7852872 | 1,19E+08 | G | C | 0,365622 | -0,14274 | 0,020058 | 1,1E-12 | Antimigraine preparations | GCST007939 | 132367 | NA | NA | NA | 0,000382 | 50,63828 |
| 6 | 1 | rs61759167 | 3091587 | T | C | 0,22306 | 0,151167 | 0,02328 | 8,4E-11 | Antimigraine preparations | GCST007939 | 132367 | NA | NA | NA | 0,000318 | 42,1649 |
| 7 | 12 | rs10849061 | 4523456 | C | T | 0,486742 | 0,119227 | 0,019418 | 8,3E-10 | Antimigraine preparations | GCST007939 | 132367 | NA | NA | NA | 0,000285 | 37,69845 |
| 8 | 10 | rs827405 | 8689003 | T | C | 0,20682 | 0,147334 | 0,024346 | 1,4E-09 | Antimigraine preparations | GCST007939 | 132367 | NA | NA | NA | 0,000277 | 36,62088 |
| 9 | 1 | rs10908504 | 1,56E+08 | C | A | 0,347013 | 0,122428 | 0,020323 | 1,7E-09 | Antimigraine preparations | GCST007939 | 132367 | NA | NA | NA | 0,000274 | 36,28972 |
| 10 | 6 | rs74809038 | 1,22E+08 | G | A | 0,154414 | 0,15841 | 0,026817 | 3,5E-09 | Antimigraine preparations | GCST007939 | 132367 | NA | NA | NA | 0,000264 | 34,89194 |
| 11 | 2 | rs11886495 | 2,3E+08 | G | C | 0,290581 | 0,120742 | 0,021328 | 1,5E-08 | Antimigraine preparations | GCST007939 | 132367 | NA | NA | NA | 0,000242 | 32,04937 |
| 12 | 7 | rs12532479 | 40427634 | C | T | 0,105246 | 0,177533 | 0,031446 | 1,6E-08 | Antimigraine preparations | GCST007939 | 132367 | NA | NA | NA | 0,000241 | 31,87313 |
| 13 | 8 | rs16914980 | 93448756 | C | T | 0,259439 | -0,12227 | 0,022149 | 3,4E-08 | Antimigraine preparations | GCST007939 | 132367 | NA | NA | NA | 0,00023 | 30,47327 |

**Table S11.** SNPs selection for antithrombotic agents

|  | chr.exposure | SNP | pos.exposure | effect_allele.exposure | other_allele.exposure | eaf.exposure | beta.exposure | se.exposure | pval.exposure | exposure | id.exposure | samplesize.exposure | mr_keep.exposure | pval_origin.exposure | data_source.exposure | r2 | F |
| --- | --- | --- | --- | --- | --- | --- | --- | --- | --- | --- | --- | --- | --- | --- | --- | --- | --- |
| 1 | 6 | rs74617384 | 160997118 | T | A | 0,0815418 | 0,114872005 | 0,012311457 | 1,1E-20 | Antithrombotic agents | GCST007924 | 132367 | NA | NA | NA | 0,000657271 | 87,05685199 |
| 2 | 1 | rs12740374 | 109817590 | T | G | 0,221168 | -0,074709755 | 0,008105044 | 3E-20 | Antithrombotic agents | GCST007924 | 132367 | NA | NA | NA | 0,000641484 | 84,96446808 |
| 3 | 6 | rs140570886 | 161013013 | C | T | 0,0155987 | 0,237483407 | 0,027271668 | 3,1E-18 | Antithrombotic agents | GCST007924 | 132367 | NA | NA | NA | 0,000572552 | 75,82922029 |
| 4 | 9 | rs1831733 | 22076071 | C | T | 0,480371 | 0,058895787 | 0,006789594 | 4,2E-18 | Antithrombotic agents | GCST007924 | 132367 | NA | NA | NA | 0,000568139 | 75,24442773 |
| 5 | 19 | rs73015016 | 11191300 | A | G | 0,119006 | -0,089790481 | 0,010400082 | 5,9E-18 | Antithrombotic agents | GCST007924 | 132367 | NA | NA | NA | 0,000562811 | 74,53848323 |
| 6 | 19 | rs7412 | 45412079 | T | C | 0,0800838 | -0,104794922 | 0,01239736 | 2,8E-17 | Antithrombotic agents | GCST007924 | 132367 | NA | NA | NA | 0,000539521 | 71,45218399 |
| 7 | 9 | rs532436 | 136149830 | A | G | 0,185935 | 0,066340996 | 0,008661608 | 1,9E-14 | Antithrombotic agents | GCST007924 | 132367 | NA | NA | NA | 0,000442991 | 58,66246871 |
| 8 | 8 | rs28601761 | 126500031 | G | C | 0,415801 | -0,051481012 | 0,006971504 | 1,5E-13 | Antithrombotic agents | GCST007924 | 132367 | NA | NA | NA | 0,000411796 | 54,52989537 |
| 9 | 11 | rs964184 | 116648917 | G | C | 0,132603 | 0,067194752 | 0,009910024 | 1,2E-11 | Antithrombotic agents | GCST007924 | 132367 | NA | NA | NA | 0,000347209 | 45,97426062 |
| 10 | 1 | rs6025 | 169519049 | T | C | 0,0233795 | 0,147872685 | 0,022256377 | 3,1E-11 | Antithrombotic agents | GCST007924 | 132367 | NA | NA | NA | 0,000333382 | 44,14285328 |
| 11 | 8 | rs17482753 | 19832646 | T | G | 0,10142 | -0,070946572 | 0,011154625 | 2E-10 | Antithrombotic agents | GCST007924 | 132367 | NA | NA | NA | 0,000305521 | 40,45258883 |
| 12 | 2 | rs4299376 | 44072576 | G | T | 0,323265 | 0,041627513 | 0,007198171 | 7,3E-09 | Antithrombotic agents | GCST007924 | 132367 | NA | NA | NA | 0,000252596 | 33,44337026 |
| 13 | 17 | rs56214516 | 43836953 | C | A | 0,192373 | 0,049012261 | 0,008626306 | 0,000000013 | Antithrombotic agents | GCST007924 | 132367 | NA | NA | NA | 0,000243823 | 32,28146457 |

**Table S12.** SNPs selection for beta blocking agents

|  | chr.exposure | SNP | pos.exposure | effect_allele.exposure | other_allele.exposure | eaf.exposure | beta.exposure | se.exposure | pval.exposure | exposure | id.exposure | samplesize.exposure | mr_keep.exposure | pval_origin.exposure | data_source.exposure | r2 | F |
| --- | --- | --- | --- | --- | --- | --- | --- | --- | --- | --- | --- | --- | --- | --- | --- | --- | --- |
| 1 | 4 | rs13125101 | 81174592 | A | G | 0,288018 | 0,097112074 | 0,009128556 | 2E-26 | Beta blocking agents | GCST007928 | 132367 | NA | NA | NA | 0,000854263 | 113,1711409 |
| 2 | 12 | rs7310615 | 111865049 | C | G | 0,482401 | 0,07970556 | 0,008299443 | 7,7E-22 | Beta blocking agents | GCST007928 | 132367 | NA | NA | NA | 0,000696301 | 92,23011016 |
| 3 | 15 | rs7183988 | 91428589 | T | G | 0,472301 | 0,078119033 | 0,008276394 | 3,8E-21 | Beta blocking agents | GCST007928 | 132367 | NA | NA | NA | 0,000672604 | 89,08915043 |
| 4 | 7 | rs3918226 | 150690176 | T | C | 0,0761934 | 0,146365761 | 0,01563296 | 7,8E-21 | Beta blocking agents | GCST007928 | 132367 | NA | NA | NA | 0,000661804 | 87,6576799 |
| 5 | 9 | rs2891168 | 22098619 | G | A | 0,488953 | 0,071484648 | 0,008223027 | 3,5E-18 | Beta blocking agents | GCST007928 | 132367 | NA | NA | NA | 0,000570604 | 75,57106831 |
| 6 | 20 | rs6039216 | 8622480 | C | T | 0,362735 | -0,067307226 | 0,008573564 | 4,1E-15 | Beta blocking agents | GCST007928 | 132367 | NA | NA | NA | 0,000465392 | 61,6302594 |
| 7 | 11 | rs1973765 | 1898664 | C | T | 0,384178 | 0,066691154 | 0,00851874 | 4,9E-15 | Beta blocking agents | GCST007928 | 132367 | NA | NA | NA | 0,000462812 | 61,2885225 |
| 8 | 12 | rs11066301 | 112871372 | G | A | 0,426667 | 0,064854955 | 0,008326362 | 6,8E-15 | Beta blocking agents | GCST007928 | 132367 | NA | NA | NA | 0,000458139 | 60,66933356 |
| 9 | 1 | rs2050265 | 11879699 | G | A | 0,164122 | -0,083174712 | 0,011092184 | 6,5E-14 | Beta blocking agents | GCST007928 | 132367 | NA | NA | NA | 0,000424604 | 56,22661044 |
| 10 | 1 | rs12046278 | 10799577 | C | T | 0,343988 | 0,063121655 | 0,008638761 | 2,7E-13 | Beta blocking agents | GCST007928 | 132367 | NA | NA | NA | 0,00040318 | 53,3884482 |
| 11 | 6 | rs62436821 | 127175945 | A | G | 0,435368 | 0,060421275 | 0,008293105 | 3,2E-13 | Beta blocking agents | GCST007928 | 132367 | NA | NA | NA | 0,000400859 | 53,08097525 |
| 12 | 6 | rs74617384 | 160997118 | T | A | 0,0800491 | 0,110204024 | 0,015174364 | 3,8E-13 | Beta blocking agents | GCST007928 | 132367 | NA | NA | NA | 0,00039831 | 52,74330926 |
| 13 | 20 | rs78302204 | 57735448 | A | G | 0,105587 | 0,095600705 | 0,013445344 | 1,2E-12 | Beta blocking agents | GCST007928 | 132367 | NA | NA | NA | 0,000381797 | 50,55589562 |
| 14 | 19 | rs167479 | 11526765 | T | G | 0,473349 | -0,056873344 | 0,008244125 | 5,2E-12 | Beta blocking agents | GCST007928 | 132367 | NA | NA | NA | 0,000359412 | 47,59066668 |
| 15 | 19 | rs36047283 | 7255701 | G | A | 0,125448 | -0,085640154 | 0,012486908 | 7E-12 | Beta blocking agents | GCST007928 | 132367 | NA | NA | NA | 0,000355231 | 47,03687691 |
| 16 | 5 | rs7442660 | 157803630 | A | G | 0,36088 | -0,058263787 | 0,008569942 | 1,1E-11 | Beta blocking agents | GCST007928 | 132367 | NA | NA | NA | 0,000349068 | 46,22054436 |
| 17 | 16 | rs258317 | 89732238 | T | C | 0,427246 | -0,056158983 | 0,008348011 | 1,7E-11 | Beta blocking agents | GCST007928 | 132367 | NA | NA | NA | 0,000341778 | 45,25492356 |
| 18 | 5 | rs17677603 | 127857493 | G | A | 0,390229 | 0,057320334 | 0,008528454 | 1,8E-11 | Beta blocking agents | GCST007928 | 132367 | NA | NA | NA | 0,000341153 | 45,17209743 |
| 19 | 2 | rs1275988 | 26914364 | C | T | 0,382355 | 0,056638242 | 0,008493221 | 2,6E-11 | Beta blocking agents | GCST007928 | 132367 | NA | NA | NA | 0,000335853 | 44,47009631 |
| 20 | 7 | rs3735533 | 27245893 | T | C | 0,0746658 | -0,104314952 | 0,015703754 | 3,1E-11 | Beta blocking agents | GCST007928 | 132367 | NA | NA | NA | 0,000333243 | 44,12447668 |
| 21 | 10 | rs17210692 | 63525112 | A | G | 0,188106 | -0,069548268 | 0,010543942 | 4,2E-11 | Beta blocking agents | GCST007928 | 132367 | NA | NA | NA | 0,000328582 | 43,50708883 |
| 22 | 10 | rs7076100 | 18759537 | A | T | 0,405545 | -0,055563161 | 0,008452227 | 4,9E-11 | Beta blocking agents | GCST007928 | 132367 | NA | NA | NA | 0,00032637 | 43,21405742 |
| 23 | 12 | rs35429 | 115555867 | G | A | 0,383864 | -0,055391609 | 0,008464163 | 6E-11 | Beta blocking agents | GCST007928 | 132367 | NA | NA | NA | 0,000323444 | 42,82657707 |
| 24 | 1 | rs10776752 | 113044328 | T | G | 0,0719762 | 0,10393464 | 0,015904859 | 6,4E-11 | Beta blocking agents | GCST007928 | 132367 | NA | NA | NA | 0,000322509 | 42,70261117 |
| 25 | 16 | rs77924615 | 20392332 | A | G | 0,194395 | -0,068638894 | 0,010518341 | 6,8E-11 | Beta blocking agents | GCST007928 | 132367 | NA | NA | NA | 0,000321608 | 42,58331594 |
| 26 | 11 | rs604723 | 100610546 | T | C | 0,273879 | -0,060166911 | 0,009330484 | 1,1E-10 | Beta blocking agents | GCST007928 | 132367 | NA | NA | NA | 0,000314044 | 41,58153621 |
| 27 | 2 | rs7368883 | 43378720 | A | G | 0,359437 | 0,055433427 | 0,008596778 | 1,1E-10 | Beta blocking agents | GCST007928 | 132367 | NA | NA | NA | 0,000314019 | 41,57817703 |
| 28 | 6 | rs2105092 | 134184972 | A | G | 0,290392 | -0,057990489 | 0,009096041 | 1,8E-10 | Beta blocking agents | GCST007928 | 132367 | NA | NA | NA | 0,00030697 | 40,64453993 |
| 29 | 3 | rs55988870 | 27483164 | C | T | 0,367162 | -0,053607436 | 0,008535039 | 3,4E-10 | Beta blocking agents | GCST007928 | 132367 | NA | NA | NA | 0,000297941 | 39,44867808 |
| 30 | 5 | rs13154066 | 32831670 | T | C | 0,40523 | -0,052533343 | 0,008379457 | 3,6E-10 | Beta blocking agents | GCST007928 | 132367 | NA | NA | NA | 0,000296845 | 39,3035465 |
| 31 | 1 | rs57748895 | 115826169 | T | A | 0,0175751 | 0,195610394 | 0,031256701 | 3,9E-10 | Beta blocking agents | GCST007928 | 132367 | NA | NA | NA | 0,000295794 | 39,1643577 |
| 32 | 16 | rs11646715 | 53824007 | G | A | 0,477716 | -0,05062956 | 0,008238775 | 8E-10 | Beta blocking agents | GCST007928 | 132367 | NA | NA | NA | 0,00028522 | 37,76387068 |
| 33 | 1 | rs2493136 | 230851536 | T | C | 0,40279 | 0,051549224 | 0,008391064 | 8,1E-10 | Beta blocking agents | GCST007928 | 132367 | NA | NA | NA | 0,000285041 | 37,74015018 |
| 34 | 15 | rs12906962 | 95312071 | C | T | 0,316545 | 0,054708365 | 0,008963919 | 0,000000001 | Beta blocking agents | GCST007928 | 132367 | NA | NA | NA | 0,000281326 | 37,24817832 |
| 35 | 4 | rs9286351 | 138441530 | G | A | 0,416748 | 0,051066837 | 0,008374271 | 1,1E-09 | Beta blocking agents | GCST007928 | 132367 | NA | NA | NA | 0,000280855 | 37,1858166 |
| 36 | 7 | rs185963 | 130450151 | T | C | 0,434857 | 0,050627008 | 0,008369661 | 1,5E-09 | Beta blocking agents | GCST007928 | 132367 | NA | NA | NA | 0,000276343 | 36,58829824 |
| 37 | 20 | rs6108787 | 10967214 | G | T | 0,475612 | 0,049310899 | 0,008258778 | 2,4E-09 | Beta blocking agents | GCST007928 | 132367 | NA | NA | NA | 0,000269251 | 35,64902706 |
| 38 | 6 | rs140570886 | 161013013 | C | T | 0,0153689 | 0,199122928 | 0,033531905 | 2,9E-09 | Beta blocking agents | GCST007928 | 132367 | NA | NA | NA | 0,000266336 | 35,2630217 |
| 39 | 4 | rs17042098 | 111664158 | A | G | 0,101276 | 0,079749929 | 0,013626774 | 4,8E-09 | Beta blocking agents | GCST007928 | 132367 | NA | NA | NA | 0,000258692 | 34,25058167 |
| 40 | 11 | rs664485 | 107184296 | A | G | 0,186539 | -0,062066166 | 0,010612431 | 0,000000005 | Beta blocking agents | GCST007928 | 132367 | NA | NA | NA | 0,000258338 | 34,20373033 |
| 41 | 3 | rs12494396 | 48178293 | G | C | 0,334855 | -0,050804817 | 0,008717046 | 5,6E-09 | Beta blocking agents | GCST007928 | 132367 | NA | NA | NA | 0,000256555 | 33,96757693 |
| 42 | 18 | rs113537788 | 43052474 | G | C | 0,0180989 | 0,180156723 | 0,031079222 | 6,8E-09 | Beta blocking agents | GCST007928 | 132367 | NA | NA | NA | 0,000253788 | 33,60114668 |
| 43 | 1 | rs417743 | 111313972 | T | C | 0,331842 | 0,050602807 | 0,008778702 | 8,2E-09 | Beta blocking agents | GCST007928 | 132367 | NA | NA | NA | 0,000250957 | 33,22630166 |
| 44 | 1 | rs4970834 | 109814880 | T | C | 0,18364 | -0,061627654 | 0,010692198 | 8,2E-09 | Beta blocking agents | GCST007928 | 132367 | NA | NA | NA | 0,000250916 | 33,22084815 |
| 45 | 11 | rs11023910 | 16334778 | T | A | 0,205274 | 0,057553295 | 0,010154822 | 0,000000014 | Beta blocking agents | GCST007928 | 132367 | NA | NA | NA | 0,000242611 | 32,12100815 |
| 46 | 16 | rs62043959 | 81525204 | C | A | 0,27972 | -0,052480659 | 0,009259525 | 0,000000014 | Beta blocking agents | GCST007928 | 132367 | NA | NA | NA | 0,000242625 | 32,12288545 |
| 47 | 8 | rs7820612 | 77316120 | A | C | 0,304079 | -0,050677634 | 0,008944821 | 0,000000015 | Beta blocking agents | GCST007928 | 132367 | NA | NA | NA | 0,00024244 | 32,09835466 |
| 48 | 11 | rs4923536 | 28422496 | G | A | 0,456313 | 0,046465392 | 0,008249064 | 0,000000018 | Beta blocking agents | GCST007928 | 132367 | NA | NA | NA | 0,000239644 | 31,72804017 |
| 49 | 6 | rs7770615 | 166177226 | C | T | 0,0676465 | -0,092071706 | 0,016358434 | 0,000000018 | Beta blocking agents | GCST007928 | 132367 | NA | NA | NA | 0,000239268 | 31,67833561 |
| 50 | 3 | rs7652333 | 72417372 | T | C | 0,483816 | 0,047401272 | 0,008436339 | 0,000000019 | Beta blocking agents | GCST007928 | 132367 | NA | NA | NA | 0,000238445 | 31,56932905 |
| 51 | 7 | rs4515482 | 107072681 | C | A | 0,224624 | -0,055357858 | 0,009883747 | 0,000000021 | Beta blocking agents | GCST007928 | 132367 | NA | NA | NA | 0,000236937 | 31,36958254 |
| 52 | 15 | rs1543927 | 75063573 | T | C | 0,26383 | 0,052594505 | 0,00940383 | 0,000000022 | Beta blocking agents | GCST007928 | 132367 | NA | NA | NA | 0,000236259 | 31,27985046 |
| 53 | 1 | rs778124 | 56606206 | A | G | 0,374347 | 0,047101221 | 0,00852121 | 0,000000032 | Beta blocking agents | GCST007928 | 132367 | NA | NA | NA | 0,000230771 | 30,55309764 |
| 54 | 11 | rs11026578 | 22495330 | T | C | 0,0656016 | 0,091600021 | 0,016652229 | 0,000000038 | Beta blocking agents | GCST007928 | 132367 | NA | NA | NA | 0,000228543 | 30,25797189 |
| 55 | 7 | rs38853 | 116356076 | G | C | 0,290021 | 0,050386062 | 0,009157615 | 0,000000038 | Beta blocking agents | GCST007928 | 132367 | NA | NA | NA | 0,000228653 | 30,27258601 |
| 56 | 4 | rs11731886 | 156659819 | C | A | 0,236995 | -0,053222102 | 0,009705774 | 0,000000042 | Beta blocking agents | GCST007928 | 132367 | NA | NA | NA | 0,000227115 | 30,06886973 |
| 57 | 17 | rs11649807 | 79357995 | G | A | 0,370876 | -0,047116861 | 0,008623614 | 0,000000047 | Beta blocking agents | GCST007928 | 132367 | NA | NA | NA | 0,000225474 | 29,8515929 |
| 58 | 8 | rs513159 | 102899476 | T | C | 0,233686 | -0,053433496 | 0,009780355 | 0,000000047 | Beta blocking agents | GCST007928 | 132367 | NA | NA | NA | 0,000225445 | 29,84773516 |
| 59 | 19 | rs12459368 | 18459377 | G | A | 0,26695 | -0,050655985 | 0,009291876 | 0,00000005 | Beta blocking agents | GCST007928 | 132367 | NA | NA | NA | 0,00022448 | 29,71995201 |

**Table S13.** SNPs selection for calcium channel blockers

|  | chr.exposure | SNP | pos.exposure | effect_allele.exposure | other_allele.exposure | eaf.exposure | beta.exposure | se.exposure | pval.exposure | exposure | id.exposure | samplesize.exposure | mr_keep.exposure | pval_origin.exposure | data_source.exposure | r2 | F |
| --- | --- | --- | --- | --- | --- | --- | --- | --- | --- | --- | --- | --- | --- | --- | --- | --- | --- |
| 1 | 4 | rs10857147 | 81181072 | T | A | 0,286785 | 0,113139437 | 0,008920802 | 7,4E-37 | Calcium channel blockers | GCST007929 | 132367 | NA | NA | NA | 0,001213705 | 160,8472535 |
| 2 | 2 | rs11126666 | 26928811 | A | G | 0,255998 | 0,106201255 | 0,009194838 | 7,4E-31 | Calcium channel blockers | GCST007929 | 132367 | NA | NA | NA | 0,001006825 | 133,4026965 |
| 3 | 11 | rs569550 | 1887068 | G | T | 0,386833 | 0,095690744 | 0,008293405 | 8,5E-31 | Calcium channel blockers | GCST007929 | 132367 | NA | NA | NA | 0,001004749 | 133,1274215 |
| 4 | 1 | rs880315 | 10796866 | C | T | 0,339218 | 0,09883418 | 0,008580194 | 1,1E-30 | Calcium channel blockers | GCST007929 | 132367 | NA | NA | NA | 0,001001395 | 132,6824846 |
| 5 | 7 | rs3918226 | 150690176 | T | C | 0,0761387 | 0,161264917 | 0,015257245 | 4,1E-26 | Calcium channel blockers | GCST007929 | 132367 | NA | NA | NA | 0,000843299 | 111,7174409 |
| 6 | 6 | rs9398823 | 127197773 | G | A | 0,437024 | 0,08327413 | 0,008121924 | 1,1E-24 | Calcium channel blockers | GCST007929 | 132367 | NA | NA | NA | 0,000793556 | 105,1225199 |
| 7 | 11 | rs633185 | 100593538 | G | C | 0,283039 | -0,090648795 | 0,008984415 | 6,1E-24 | Calcium channel blockers | GCST007929 | 132367 | NA | NA | NA | 0,000768477 | 101,7976891 |
| 8 | 1 | rs3790604 | 113046879 | A | C | 0,0725619 | 0,1520593 | 0,01547951 | 8,9E-23 | Calcium channel blockers | GCST007929 | 132367 | NA | NA | NA | 0,000728476 | 96,49504106 |
| 9 | 13 | rs7983337 | 32181534 | C | T | 0,493522 | -0,077298487 | 0,008079718 | 1,1E-21 | Calcium channel blockers | GCST007929 | 132367 | NA | NA | NA | 0,000690987 | 91,52568434 |
| 10 | 3 | rs2643826 | 27562988 | T | C | 0,452509 | 0,075560754 | 0,008097102 | 1E-20 | Calcium channel blockers | GCST007929 | 132367 | NA | NA | NA | 0,000657458 | 87,08168102 |
| 11 | 7 | rs3735533 | 27245893 | T | C | 0,0735793 | -0,141444033 | 0,015402461 | 4,2E-20 | Calcium channel blockers | GCST007929 | 132367 | NA | NA | NA | 0,000636697 | 84,33006791 |
| 12 | 19 | rs12978472 | 7257990 | G | C | 0,127677 | -0,108858108 | 0,012116541 | 2,6E-19 | Calcium channel blockers | GCST007929 | 132367 | NA | NA | NA | 0,000609424 | 80,71563741 |
| 13 | 15 | rs1894400 | 91428955 | T | C | 0,323994 | 0,075941215 | 0,008576929 | 8,4E-19 | Calcium channel blockers | GCST007929 | 132367 | NA | NA | NA | 0,000591908 | 78,39436451 |
| 14 | 12 | rs35429 | 115555867 | G | A | 0,382183 | -0,07223407 | 0,008266229 | 2,4E-18 | Calcium channel blockers | GCST007929 | 132367 | NA | NA | NA | 0,000576553 | 76,35943283 |
| 15 | 10 | rs72821787 | 63444217 | A | C | 0,156182 | -0,095073594 | 0,011068223 | 8,7E-18 | Calcium channel blockers | GCST007929 | 132367 | NA | NA | NA | 0,000557112 | 73,78319942 |
| 16 | 7 | rs7808452 | 27321550 | A | T | 0,101394 | 0,111801142 | 0,013305825 | 4,4E-17 | Calcium channel blockers | GCST007929 | 132367 | NA | NA | NA | 0,000533086 | 70,59961551 |
| 17 | 16 | rs460879 | 89712889 | T | C | 0,430755 | -0,067857549 | 0,008151074 | 8,4E-17 | Calcium channel blockers | GCST007929 | 132367 | NA | NA | NA | 0,000523311 | 69,30429097 |
| 18 | 20 | rs6078002 | 10979010 | A | C | 0,289318 | 0,073390788 | 0,008855823 | 1,2E-16 | Calcium channel blockers | GCST007929 | 132367 | NA | NA | NA | 0,000518585 | 68,67816256 |
| 19 | 15 | rs7174222 | 81018543 | T | C | 0,468408 | 0,06535783 | 0,00806947 | 5,5E-16 | Calcium channel blockers | GCST007929 | 132367 | NA | NA | NA | 0,000495348 | 65,59921021 |
| 20 | 1 | rs3753584 | 11864586 | C | T | 0,163965 | -0,087289015 | 0,010817492 | 7,1E-16 | Calcium channel blockers | GCST007929 | 132367 | NA | NA | NA | 0,000491669 | 65,11176621 |
| 21 | 19 | rs167479 | 11526765 | T | G | 0,473277 | -0,06429934 | 0,008042371 | 1,3E-15 | Calcium channel blockers | GCST007929 | 132367 | NA | NA | NA | 0,000482676 | 63,92022585 |
| 22 | 13 | rs620124 | 22295868 | G | C | 0,329831 | -0,066454275 | 0,008672728 | 1,8E-14 | Calcium channel blockers | GCST007929 | 132367 | NA | NA | NA | 0,000443366 | 58,71213048 |
| 23 | 10 | rs191572726 | 106983028 | C | T | 0,0122709 | 0,278685684 | 0,03658244 | 2,6E-14 | Calcium channel blockers | GCST007929 | 132367 | NA | NA | NA | 0,000438242 | 58,03332143 |
| 24 | 5 | rs7733331 | 32828846 | T | C | 0,400308 | -0,060675316 | 0,008208006 | 1,4E-13 | Calcium channel blockers | GCST007929 | 132367 | NA | NA | NA | 0,000412657 | 54,64395904 |
| 25 | 3 | rs9841978 | 53730735 | A | G | 0,322134 | 0,063494085 | 0,008657698 | 2,2E-13 | Calcium channel blockers | GCST007929 | 132367 | NA | NA | NA | 0,000406168 | 53,78425248 |
| 26 | 17 | rs4305 | 61558229 | A | G | 0,444171 | 0,059748909 | 0,008193735 | 3,1E-13 | Calcium channel blockers | GCST007929 | 132367 | NA | NA | NA | 0,000401552 | 53,17280106 |
| 27 | 5 | rs7701003 | 157824481 | G | A | 0,370667 | -0,059909914 | 0,008302363 | 5,4E-13 | Calcium channel blockers | GCST007929 | 132367 | NA | NA | NA | 0,000393227 | 52,06998325 |
| 28 | 3 | rs9855086 | 169131330 | T | A | 0,4614 | -0,057911126 | 0,008047229 | 6,2E-13 | Calcium channel blockers | GCST007929 | 132367 | NA | NA | NA | 0,000391094 | 51,78747681 |
| 29 | 11 | rs494356 | 117071970 | T | C | 0,132695 | 0,083987685 | 0,011858067 | 1,4E-12 | Calcium channel blockers | GCST007929 | 132367 | NA | NA | NA | 0,000378843 | 50,16454035 |
| 30 | 7 | rs12705389 | 106405642 | T | C | 0,241001 | 0,06615352 | 0,00938393 | 1,8E-12 | Calcium channel blockers | GCST007929 | 132367 | NA | NA | NA | 0,000375313 | 49,69696438 |
| 31 | 12 | rs3184504 | 111884608 | T | C | 0,481977 | 0,056394442 | 0,008023773 | 2,1E-12 | Calcium channel blockers | GCST007929 | 132367 | NA | NA | NA | 0,000373056 | 49,39793833 |
| 32 | 15 | rs2168518 | 75081078 | G | A | 0,321597 | 0,05984615 | 0,008581712 | 3,1E-12 | Calcium channel blockers | GCST007929 | 132367 | NA | NA | NA | 0,00036727 | 48,63153991 |
| 33 | 4 | rs7685862 | 111389101 | C | A | 0,203951 | 0,068732413 | 0,009977091 | 5,6E-12 | Calcium channel blockers | GCST007929 | 132367 | NA | NA | NA | 0,00035841 | 47,45792502 |
| 34 | 12 | rs11105352 | 90026462 | A | G | 0,166874 | -0,07447448 | 0,010823489 | 6E-12 | Calcium channel blockers | GCST007929 | 132367 | NA | NA | NA | 0,000357557 | 47,34497255 |
| 35 | 20 | rs259983 | 57735457 | C | A | 0,132896 | 0,081478097 | 0,011900197 | 7,6E-12 | Calcium channel blockers | GCST007929 | 132367 | NA | NA | NA | 0,00035403 | 46,87776141 |
| 36 | 5 | rs6595839 | 127881566 | T | C | 0,313861 | 0,059347497 | 0,00871797 | 9,9E-12 | Calcium channel blockers | GCST007929 | 132367 | NA | NA | NA | 0,000349979 | 46,34122031 |
| 37 | 2 | rs2943634 | 227068080 | A | C | 0,326679 | -0,058035315 | 0,008538671 | 1,1E-11 | Calcium channel blockers | GCST007929 | 132367 | NA | NA | NA | 0,000348877 | 46,19527849 |
| 38 | 16 | rs62033406 | 53824226 | G | A | 0,407052 | 0,055290178 | 0,00816952 | 1,3E-11 | Calcium channel blockers | GCST007929 | 132367 | NA | NA | NA | 0,000345918 | 45,80325438 |
| 39 | 20 | rs4813867 | 8623250 | C | T | 0,337352 | -0,057213284 | 0,008506333 | 1,7E-11 | Calcium channel blockers | GCST007929 | 132367 | NA | NA | NA | 0,00034165 | 45,23789932 |
| 40 | 10 | rs12258967 | 18727959 | G | C | 0,298591 | -0,05833311 | 0,008768982 | 2,9E-11 | Calcium channel blockers | GCST007929 | 132367 | NA | NA | NA | 0,0003342 | 44,25122986 |
| 41 | 19 | rs4804517 | 7406066 | G | A | 0,334256 | -0,056163068 | 0,008506864 | 4,1E-11 | Calcium channel blockers | GCST007929 | 132367 | NA | NA | NA | 0,000329185 | 43,58690835 |
| 42 | 12 | rs317685 | 69715457 | G | A | 0,281102 | 0,057492406 | 0,008943272 | 1,3E-10 | Calcium channel blockers | GCST007929 | 132367 | NA | NA | NA | 0,000312114 | 41,32582824 |
| 43 | 5 | rs1422278 | 122467417 | T | G | 0,131748 | 0,075955638 | 0,011840077 | 1,4E-10 | Calcium channel blockers | GCST007929 | 132367 | NA | NA | NA | 0,000310811 | 41,15327918 |
| 44 | 10 | rs57866767 | 96023077 | C | T | 0,433102 | -0,051913773 | 0,008102719 | 1,5E-10 | Calcium channel blockers | GCST007929 | 132367 | NA | NA | NA | 0,00031002 | 41,04847811 |
| 45 | 4 | rs72689147 | 156639888 | T | G | 0,184352 | -0,066058708 | 0,010371672 | 1,9E-10 | Calcium channel blockers | GCST007929 | 132367 | NA | NA | NA | 0,000306373 | 40,56542768 |
| 46 | 6 | rs553108 | 31840455 | A | G | 0,418103 | -0,051669798 | 0,008151226 | 2,3E-10 | Calcium channel blockers | GCST007929 | 132367 | NA | NA | NA | 0,00030347 | 40,1810364 |
| 47 | 9 | rs6271 | 136522274 | T | C | 0,0739659 | -0,096342052 | 0,01532435 | 3,2E-10 | Calcium channel blockers | GCST007929 | 132367 | NA | NA | NA | 0,00029851 | 39,52402003 |
| 48 | 6 | rs9369409 | 43346462 | C | G | 0,403623 | 0,051312717 | 0,008163599 | 3,3E-10 | Calcium channel blockers | GCST007929 | 132367 | NA | NA | NA | 0,000298385 | 39,50755312 |
| 49 | 6 | rs162185 | 134226147 | C | T | 0,409998 | -0,051430378 | 0,008213548 | 3,8E-10 | Calcium channel blockers | GCST007929 | 132367 | NA | NA | NA | 0,000296121 | 39,20769242 |
| 50 | 12 | rs2377585 | 8932201 | G | T | 0,274675 | -0,056192673 | 0,0089832 | 4E-10 | Calcium channel blockers | GCST007929 | 132367 | NA | NA | NA | 0,000295522 | 39,12827123 |
| 51 | 19 | rs7412 | 45412079 | T | C | 0,082871 | -0,091432187 | 0,014629545 | 4,1E-10 | Calcium channel blockers | GCST007929 | 132367 | NA | NA | NA | 0,000295005 | 39,05980189 |
| 52 | 8 | rs891556 | 10623633 | G | C | 0,397703 | 0,051189439 | 0,008223948 | 4,8E-10 | Calcium channel blockers | GCST007929 | 132367 | NA | NA | NA | 0,000292613 | 38,74302417 |
| 53 | 4 | rs33966350 | 111431444 | A | G | 0,012401 | 0,224491192 | 0,036154383 | 5,3E-10 | Calcium channel blockers | GCST007929 | 132367 | NA | NA | NA | 0,000291186 | 38,55406272 |
| 54 | 6 | rs998584 | 43757896 | A | C | 0,481178 | 0,049987934 | 0,008051252 | 5,3E-10 | Calcium channel blockers | GCST007929 | 132367 | NA | NA | NA | 0,000291137 | 38,54756664 |
| 55 | 11 | rs10896795 | 58366561 | G | A | 0,241124 | -0,058248167 | 0,009389243 | 5,5E-10 | Calcium channel blockers | GCST007929 | 132367 | NA | NA | NA | 0,000290668 | 38,48546774 |
| 56 | 10 | rs3740393 | 104636655 | C | G | 0,136347 | -0,07248351 | 0,011709435 | 6E-10 | Calcium channel blockers | GCST007929 | 132367 | NA | NA | NA | 0,000289402 | 38,3177474 |
| 57 | 12 | rs60370741 | 121966676 | C | T | 0,461998 | -0,049962808 | 0,008094293 | 6,7E-10 | Calcium channel blockers | GCST007929 | 132367 | NA | NA | NA | 0,00028776 | 38,10037594 |
| 58 | 20 | rs6031431 | 42795152 | G | A | 0,460604 | 0,0502499 | 0,008173999 | 7,9E-10 | Calcium channel blockers | GCST007929 | 132367 | NA | NA | NA | 0,000285429 | 37,79155061 |
| 59 | 2 | rs1439211 | 165094300 | A | C | 0,273839 | -0,055266418 | 0,009025103 | 9,1E-10 | Calcium channel blockers | GCST007929 | 132367 | NA | NA | NA | 0,000283215 | 37,49831714 |
| 60 | 18 | rs72915163 | 48792829 | T | C | 0,254784 | 0,057151266 | 0,009350833 | 9,8E-10 | Calcium channel blockers | GCST007929 | 132367 | NA | NA | NA | 0,00028213 | 37,35464032 |
| 61 | 16 | rs7203816 | 81597957 | A | G | 0,31252 | -0,052671659 | 0,008698537 | 1,4E-09 | Calcium channel blockers | GCST007929 | 132367 | NA | NA | NA | 0,000276925 | 36,66527688 |
| 62 | 4 | rs2341599 | 156433308 | G | A | 0,339251 | 0,050767986 | 0,008482801 | 2,2E-09 | Calcium channel blockers | GCST007929 | 132367 | NA | NA | NA | 0,000270523 | 35,81745797 |
| 63 | 17 | rs11653468 | 47192620 | A | G | 0,235338 | -0,056122836 | 0,009464622 | 0,000000003 | Calcium channel blockers | GCST007929 | 132367 | NA | NA | NA | 0,000265569 | 35,16139527 |
| 64 | 5 | rs27041 | 96115721 | C | T | 0,148646 | -0,067071121 | 0,011306505 | 0,000000003 | Calcium channel blockers | GCST007929 | 132367 | NA | NA | NA | 0,000265778 | 35,18907427 |
| 65 | 17 | rs17608766 | 45013271 | C | T | 0,145965 | 0,067002878 | 0,011356909 | 3,6E-09 | Calcium channel blockers | GCST007929 | 132367 | NA | NA | NA | 0,000262889 | 34,80647702 |
| 66 | 8 | rs7463212 | 143991858 | T | A | 0,459666 | 0,04745489 | 0,008064536 | 0,000000004 | Calcium channel blockers | GCST007929 | 132367 | NA | NA | NA | 0,000261523 | 34,6255419 |
| 67 | 17 | rs12938899 | 7525546 | T | C | 0,199939 | -0,058853246 | 0,010041159 | 4,6E-09 | Calcium channel blockers | GCST007929 | 132367 | NA | NA | NA | 0,000259466 | 34,35315059 |
| 68 | 7 | rs11556924 | 129663496 | T | C | 0,389692 | -0,048157666 | 0,008222885 | 4,7E-09 | Calcium channel blockers | GCST007929 | 132367 | NA | NA | NA | 0,000259054 | 34,29855994 |
| 69 | 3 | rs35593046 | 53553923 | T | G | 0,263655 | -0,054404004 | 0,009303389 | 0,000000005 | Calcium channel blockers | GCST007929 | 132367 | NA | NA | NA | 0,000258278 | 34,19580227 |
| 70 | 1 | rs61772626 | 57015668 | G | A | 0,123313 | 0,070881731 | 0,01219822 | 6,2E-09 | Calcium channel blockers | GCST007929 | 132367 | NA | NA | NA | 0,000255026 | 33,76518411 |
| 71 | 8 | rs804263 | 11630915 | T | C | 0,422661 | 0,04730512 | 0,008143483 | 6,3E-09 | Calcium channel blockers | GCST007929 | 132367 | NA | NA | NA | 0,000254862 | 33,74344027 |
| 72 | 6 | rs62434123 | 150999464 | T | C | 0,0713639 | -0,090459779 | 0,015589352 | 6,5E-09 | Calcium channel blockers | GCST007929 | 132367 | NA | NA | NA | 0,000254311 | 33,67040668 |
| 73 | 7 | rs972283 | 130466854 | A | G | 0,491048 | -0,046500169 | 0,008029086 | 0,000000007 | Calcium channel blockers | GCST007929 | 132367 | NA | NA | NA | 0,00025333 | 33,54055518 |
| 74 | 1 | rs57748895 | 115826169 | T | A | 0,0175421 | 0,175209601 | 0,030480331 | 0,000000009 | Calcium channel blockers | GCST007929 | 132367 | NA | NA | NA | 0,000249568 | 33,04227051 |
| 75 | 20 | rs6021247 | 50108980 | G | A | 0,471729 | -0,046153791 | 0,00804609 | 9,7E-09 | Calcium channel blockers | GCST007929 | 132367 | NA | NA | NA | 0,000248518 | 32,90322064 |
| 76 | 2 | rs13387221 | 44135314 | A | G | 0,174669 | -0,060807096 | 0,010603273 | 9,8E-09 | Calcium channel blockers | GCST007929 | 132367 | NA | NA | NA | 0,000248394 | 32,88683195 |
| 77 | 14 | rs8009025 | 98631171 | C | T | 0,206232 | 0,057095396 | 0,009965932 | 0,00000001 | Calcium channel blockers | GCST007929 | 132367 | NA | NA | NA | 0,000247901 | 32,82160016 |
| 78 | 8 | rs3110291 | 105981953 | C | A | 0,195127 | -0,059019261 | 0,010337057 | 0,000000011 | Calcium channel blockers | GCST007929 | 132367 | NA | NA | NA | 0,000246211 | 32,59771637 |
| 79 | 11 | rs893311 | 16318518 | T | G | 0,20571 | 0,056468227 | 0,009893514 | 0,000000011 | Calcium channel blockers | GCST007929 | 132367 | NA | NA | NA | 0,000246048 | 32,57621227 |
| 80 | 8 | rs907183 | 8729761 | C | G | 0,458738 | 0,046217404 | 0,00808526 | 0,000000011 | Calcium channel blockers | GCST007929 | 132367 | NA | NA | NA | 0,000246795 | 32,67507362 |
| 81 | 11 | rs10835916 | 32483319 | C | A | 0,406175 | 0,046684858 | 0,008185157 | 0,000000012 | Calcium channel blockers | GCST007929 | 132367 | NA | NA | NA | 0,000245704 | 32,53055326 |
| 82 | 5 | rs10061288 | 114389826 | A | G | 0,485343 | 0,045694383 | 0,008036829 | 0,000000013 | Calcium channel blockers | GCST007929 | 132367 | NA | NA | NA | 0,000244158 | 32,32582434 |
| 83 | 9 | rs1250505 | 128193456 | T | G | 0,422789 | -0,046380003 | 0,00814743 | 0,000000013 | Calcium channel blockers | GCST007929 | 132367 | NA | NA | NA | 0,000244756 | 32,40512472 |
| 84 | 7 | rs13230181 | 2537866 | A | G | 0,235101 | 0,053742903 | 0,009460599 | 0,000000013 | Calcium channel blockers | GCST007929 | 132367 | NA | NA | NA | 0,000243736 | 32,26995968 |
| 85 | 3 | rs2276838 | 53858164 | C | T | 0,460738 | -0,046080083 | 0,008108185 | 0,000000013 | Calcium channel blockers | GCST007929 | 132367 | NA | NA | NA | 0,000243946 | 32,2977803 |
| 86 | 20 | rs6062536 | 62443779 | A | G | 0,233334 | -0,053996746 | 0,009493088 | 0,000000013 | Calcium channel blockers | GCST007929 | 132367 | NA | NA | NA | 0,000244362 | 32,35292647 |
| 87 | 3 | rs6771917 | 48108442 | T | C | 0,240852 | -0,053709047 | 0,009445265 | 0,000000013 | Calcium channel blockers | GCST007929 | 132367 | NA | NA | NA | 0,00024422 | 32,33404467 |
| 88 | 7 | rs10254101 | 151415536 | T | C | 0,287323 | 0,049741682 | 0,008863186 | 0,00000002 | Calcium channel blockers | GCST007929 | 132367 | NA | NA | NA | 0,000237891 | 31,49594189 |
| 89 | 9 | rs76038906 | 113250200 | T | G | 0,034493 | 0,123353899 | 0,022045885 | 0,000000022 | Calcium channel blockers | GCST007929 | 132367 | NA | NA | NA | 0,000236466 | 31,30719337 |
| 90 | 4 | rs56388530 | 106910958 | C | T | 0,240756 | -0,052418575 | 0,009384992 | 0,000000023 | Calcium channel blockers | GCST007929 | 132367 | NA | NA | NA | 0,000235625 | 31,19579188 |
| 91 | 11 | rs12274440 | 9959637 | A | G | 0,112471 | 0,071488101 | 0,012859245 | 0,000000027 | Calcium channel blockers | GCST007929 | 132367 | NA | NA | NA | 0,000233429 | 30,90508834 |
| 92 | 14 | rs72681869 | 50655357 | C | G | 0,0112732 | -0,211039164 | 0,038048871 | 0,000000029 | Calcium channel blockers | GCST007929 | 132367 | NA | NA | NA | 0,00023236 | 30,7635189 |
| 93 | 8 | rs73207888 | 18282931 | T | C | 0,0339351 | 0,123120096 | 0,022212128 | 0,00000003 | Calcium channel blockers | GCST007929 | 132367 | NA | NA | NA | 0,000232058 | 30,72352199 |
| 94 | 12 | rs4547172 | 48338422 | A | G | 0,284436 | 0,049543405 | 0,008968398 | 0,000000033 | Calcium channel blockers | GCST007929 | 132367 | NA | NA | NA | 0,000230495 | 30,51654814 |
| 95 | 4 | rs11726072 | 187543527 | A | G | 0,122139 | -0,06785178 | 0,012292728 | 0,000000034 | Calcium channel blockers | GCST007929 | 132367 | NA | NA | NA | 0,000230116 | 30,46627473 |
| 96 | 4 | rs10015412 | 144162582 | A | C | 0,313756 | 0,047643527 | 0,00863872 | 0,000000035 | Calcium channel blockers | GCST007929 | 132367 | NA | NA | NA | 0,000229736 | 30,41602252 |
| 97 | 1 | rs59180873 | 27848634 | G | A | 0,0777984 | -0,083082077 | 0,01507787 | 0,000000036 | Calcium channel blockers | GCST007929 | 132367 | NA | NA | NA | 0,000229327 | 30,36184497 |
| 98 | 5 | rs2962383 | 157494859 | T | C | 0,301942 | 0,048286941 | 0,008783025 | 0,000000038 | Calcium channel blockers | GCST007929 | 132367 | NA | NA | NA | 0,000228293 | 30,22487742 |
| 99 | 16 | rs13329952 | 20366507 | C | T | 0,192068 | -0,055942094 | 0,010179543 | 0,000000039 | Calcium channel blockers | GCST007929 | 132367 | NA | NA | NA | 0,000228109 | 30,20051062 |
| 100 | 8 | rs2977324 | 76716737 | T | G | 0,298571 | -0,04855126 | 0,008838592 | 0,000000039 | Calcium channel blockers | GCST007929 | 132367 | NA | NA | NA | 0,000227906 | 30,1736824 |
| 101 | 4 | rs3819199 | 109002559 | C | T | 0,429505 | 0,044605074 | 0,008156236 | 0,000000045 | Calcium channel blockers | GCST007929 | 132367 | NA | NA | NA | 0,000225897 | 29,90765902 |
| 102 | 7 | rs143524414 | 98965615 | A | G | 0,0650858 | -0,089636155 | 0,016399388 | 0,000000046 | Calcium channel blockers | GCST007929 | 132367 | NA | NA | NA | 0,000225649 | 29,87476949 |
| 103 | 12 | rs7308105 | 54424123 | C | T | 0,36704 | -0,045739778 | 0,008371669 | 0,000000047 | Calcium channel blockers | GCST007929 | 132367 | NA | NA | NA | 0,000225469 | 29,8509037 |
| 104 | 22 | rs71313931 | 19960184 | G | C | 0,283681 | 0,048869627 | 0,008960731 | 0,000000049 | Calcium channel blockers | GCST007929 | 132367 | NA | NA | NA | 0,000224654 | 29,74298909 |

**Table S13.** SNPs selection for diuretics

|  | chr.exposure | SNP | pos.exposure | effect_allele.exposure | other_allele.exposure | eaf.exposure | beta.exposure | se.exposure | pval.exposure | exposure | id.exposure | samplesize.exposure | mr_keep.exposure | pval_origin.exposure | data_source.exposure | r2 | F |
| --- | --- | --- | --- | --- | --- | --- | --- | --- | --- | --- | --- | --- | --- | --- | --- | --- | --- |
| 1 | 2 | rs1275988 | 26914364 | C | T | 0,384127 | 0,099095875 | 0,008102494 | 2,1E-34 | Diuretics | GCST007927 | 132367 | NA | NA | NA | 0,001128765 | 149,5778058 |
| 2 | 4 | rs10857147 | 81181072 | T | A | 0,286176 | 0,104208016 | 0,008750747 | 1,1E-32 | Diuretics | GCST007927 | 132367 | NA | NA | NA | 0,001070206 | 141,8095475 |
| 3 | 11 | rs569550 | 1887068 | G | T | 0,385649 | 0,09494089 | 0,008128008 | 1,6E-31 | Diuretics | GCST007927 | 132367 | NA | NA | NA | 0,001029701 | 136,4368938 |
| 4 | 7 | rs3918226 | 150690176 | T | C | 0,0763907 | 0,166541395 | 0,014925387 | 6,5E-29 | Diuretics | GCST007927 | 132367 | NA | NA | NA | 0,000939734 | 124,5049496 |
| 5 | 7 | rs916880 | 27229119 | G | A | 0,0730081 | -0,169045029 | 0,015202282 | 1E-28 | Diuretics | GCST007927 | 132367 | NA | NA | NA | 0,000933259 | 123,6461751 |
| 6 | 6 | rs9375459 | 127147704 | T | C | 0,435739 | 0,087651471 | 0,007926246 | 2E-28 | Diuretics | GCST007927 | 132367 | NA | NA | NA | 0,000923002 | 122,2860185 |
| 7 | 1 | rs880315 | 10796866 | C | T | 0,337723 | 0,089395954 | 0,008414763 | 2,3E-26 | Diuretics | GCST007927 | 132367 | NA | NA | NA | 0,000851926 | 112,8613754 |
| 8 | 12 | rs35436 | 115554523 | T | C | 0,382191 | -0,0799003 | 0,008091302 | 5,4E-23 | Diuretics | GCST007927 | 132367 | NA | NA | NA | 0,00073614 | 97,51095188 |
| 9 | 20 | rs6039216 | 8622480 | C | T | 0,36254 | -0,076972714 | 0,008192966 | 5,7E-21 | Diuretics | GCST007927 | 132367 | NA | NA | NA | 0,00066638 | 88,26422916 |
| 10 | 3 | rs591668 | 27535931 | A | G | 0,396391 | -0,075242162 | 0,008042858 | 8,3E-21 | Diuretics | GCST007927 | 132367 | NA | NA | NA | 0,000660747 | 87,51756377 |
| 11 | 5 | rs12656497 | 32831939 | T | C | 0,404841 | -0,073033892 | 0,0080067 | 7,4E-20 | Diuretics | GCST007927 | 132367 | NA | NA | NA | 0,000628187 | 83,20227804 |
| 12 | 5 | rs7701003 | 157824481 | G | A | 0,370273 | -0,073908247 | 0,008135208 | 1E-19 | Diuretics | GCST007927 | 132367 | NA | NA | NA | 0,000623158 | 82,53571658 |
| 13 | 7 | rs6961048 | 27328187 | G | C | 0,10066 | 0,118298494 | 0,013091684 | 1,6E-19 | Diuretics | GCST007927 | 132367 | NA | NA | NA | 0,000616481 | 81,65087037 |
| 14 | 15 | rs1894401 | 91429042 | G | A | 0,472142 | 0,070338357 | 0,007909967 | 6E-19 | Diuretics | GCST007927 | 132367 | NA | NA | NA | 0,00059703 | 79,07305938 |
| 15 | 1 | rs72993045 | 113047178 | C | T | 0,03029 | 0,201280792 | 0,022972393 | 1,9E-18 | Diuretics | GCST007927 | 132367 | NA | NA | NA | 0,000579643 | 76,76895655 |
| 16 | 19 | rs12978472 | 7257990 | G | C | 0,127718 | -0,101797515 | 0,011856302 | 9E-18 | Diuretics | GCST007927 | 132367 | NA | NA | NA | 0,000556614 | 73,71727355 |
| 17 | 20 | rs78302204 | 57735448 | A | G | 0,105716 | 0,106527733 | 0,012842886 | 1,1E-16 | Diuretics | GCST007927 | 132367 | NA | NA | NA | 0,000519511 | 68,80080884 |
| 18 | 12 | rs11105352 | 90026462 | A | G | 0,166833 | -0,087141979 | 0,010606129 | 2,1E-16 | Diuretics | GCST007927 | 132367 | NA | NA | NA | 0,00050973 | 67,50477601 |
| 19 | 10 | rs72831344 | 63518378 | T | C | 0,145464 | -0,090673989 | 0,011139335 | 4E-16 | Diuretics | GCST007927 | 132367 | NA | NA | NA | 0,000500322 | 66,25831519 |
| 20 | 13 | rs9506725 | 22314146 | C | T | 0,370441 | -0,066165998 | 0,00814593 | 4,6E-16 | Diuretics | GCST007927 | 132367 | NA | NA | NA | 0,000498187 | 65,97536881 |
| 21 | 12 | rs7310615 | 111865049 | C | G | 0,48155 | 0,064379096 | 0,007930315 | 4,7E-16 | Diuretics | GCST007927 | 132367 | NA | NA | NA | 0,000497637 | 65,90255907 |
| 22 | 8 | rs17153559 | 11491128 | G | A | 0,375293 | 0,066706247 | 0,008276551 | 7,6E-16 | Diuretics | GCST007927 | 132367 | NA | NA | NA | 0,000490503 | 64,95725611 |
| 23 | 15 | rs11632414 | 75061916 | A | G | 0,3264 | 0,066837416 | 0,008456791 | 2,7E-15 | Diuretics | GCST007927 | 132367 | NA | NA | NA | 0,000471676 | 62,46281842 |
| 24 | 5 | rs10066170 | 127863098 | G | A | 0,392928 | 0,063519455 | 0,008152661 | 6,6E-15 | Diuretics | GCST007927 | 132367 | NA | NA | NA | 0,000458391 | 60,70273228 |
| 25 | 20 | rs6108787 | 10967214 | G | T | 0,475926 | 0,060976767 | 0,007884688 | 1E-14 | Diuretics | GCST007927 | 132367 | NA | NA | NA | 0,000451631 | 59,80716393 |
| 26 | 8 | rs4537305 | 8902670 | G | A | 0,469306 | 0,060717795 | 0,007869348 | 1,2E-14 | Diuretics | GCST007927 | 132367 | NA | NA | NA | 0,000449551 | 59,53164341 |
| 27 | 16 | rs56094641 | 53806453 | G | A | 0,40302 | 0,059995114 | 0,008004352 | 6,6E-14 | Diuretics | GCST007927 | 132367 | NA | NA | NA | 0,000424244 | 56,17884803 |
| 28 | 8 | rs13264580 | 10250699 | T | C | 0,379121 | 0,059026765 | 0,008102181 | 3,2E-13 | Diuretics | GCST007927 | 132367 | NA | NA | NA | 0,000400812 | 53,07469809 |
| 29 | 11 | rs2455569 | 100585807 | T | C | 0,273535 | -0,064772524 | 0,008903907 | 3,5E-13 | Diuretics | GCST007927 | 132367 | NA | NA | NA | 0,000399638 | 52,9192655 |
| 30 | 16 | rs2278557 | 30093779 | G | C | 0,40521 | 0,058077356 | 0,00800263 | 3,9E-13 | Diuretics | GCST007927 | 132367 | NA | NA | NA | 0,000397737 | 52,66736663 |
| 31 | 5 | rs6894014 | 114393181 | T | G | 0,485217 | 0,056612507 | 0,007896427 | 7,5E-13 | Diuretics | GCST007927 | 132367 | NA | NA | NA | 0,000388164 | 51,39926643 |
| 32 | 19 | rs167479 | 11526765 | T | G | 0,473078 | -0,056449016 | 0,007876236 | 7,7E-13 | Diuretics | GCST007927 | 132367 | NA | NA | NA | 0,000387907 | 51,36517755 |
| 33 | 1 | rs12567136 | 11883731 | T | C | 0,163932 | -0,07584197 | 0,01060785 | 8,7E-13 | Diuretics | GCST007927 | 132367 | NA | NA | NA | 0,000386026 | 51,11612031 |
| 34 | 1 | rs2999159 | 113230758 | A | G | 0,168544 | -0,074384791 | 0,010479969 | 1,3E-12 | Diuretics | GCST007927 | 132367 | NA | NA | NA | 0,000380455 | 50,37809856 |
| 35 | 6 | rs17080089 | 150997269 | G | T | 0,0716652 | -0,106669389 | 0,015238674 | 2,6E-12 | Diuretics | GCST007927 | 132367 | NA | NA | NA | 0,000370037 | 48,99803734 |
| 36 | 15 | rs7174222 | 81018543 | T | C | 0,467487 | 0,054932513 | 0,007902219 | 3,6E-12 | Diuretics | GCST007927 | 132367 | NA | NA | NA | 0,000364941 | 48,32304138 |
| 37 | 10 | rs976785 | 18705074 | T | C | 0,330782 | -0,057924587 | 0,008390972 | 5,1E-12 | Diuretics | GCST007927 | 132367 | NA | NA | NA | 0,000359886 | 47,65350326 |
| 38 | 11 | rs6483656 | 9771687 | G | C | 0,35589 | -0,056152007 | 0,008215897 | 8,2E-12 | Diuretics | GCST007927 | 132367 | NA | NA | NA | 0,000352767 | 46,71044736 |
| 39 | 11 | rs12801188 | 47388214 | A | G | 0,363738 | 0,055872217 | 0,008195314 | 9,3E-12 | Diuretics | GCST007927 | 132367 | NA | NA | NA | 0,000351017 | 46,47870914 |
| 40 | 12 | rs10878947 | 69642683 | A | T | 0,27337 | 0,05999934 | 0,008835427 | 1,1E-11 | Diuretics | GCST007927 | 132367 | NA | NA | NA | 0,000348263 | 46,11383776 |
| 41 | 16 | rs77924615 | 20392332 | A | G | 0,194072 | -0,068199584 | 0,010057113 | 1,2E-11 | Diuretics | GCST007927 | 132367 | NA | NA | NA | 0,000347285 | 45,98436651 |
| 42 | 17 | rs35317317 | 47450521 | A | T | 0,374293 | 0,055267322 | 0,008188818 | 1,5E-11 | Diuretics | GCST007927 | 132367 | NA | NA | NA | 0,000344005 | 45,54994235 |
| 43 | 13 | rs1200385 | 32217240 | G | C | 0,438386 | -0,05330472 | 0,007915367 | 1,6E-11 | Diuretics | GCST007927 | 132367 | NA | NA | NA | 0,0003425 | 45,35055959 |
| 44 | 7 | rs17477177 | 106411858 | C | T | 0,200037 | 0,066040307 | 0,009839307 | 1,9E-11 | Diuretics | GCST007927 | 132367 | NA | NA | NA | 0,000340221 | 45,04873089 |
| 45 | 10 | rs10764331 | 18451836 | G | A | 0,413287 | 0,053320685 | 0,008025092 | 3E-11 | Diuretics | GCST007927 | 132367 | NA | NA | NA | 0,000333401 | 44,14533985 |
| 46 | 6 | rs12190287 | 134214525 | G | C | 0,374524 | -0,053729375 | 0,008114938 | 3,6E-11 | Diuretics | GCST007927 | 132367 | NA | NA | NA | 0,000331077 | 43,83758551 |
| 47 | 6 | rs9394951 | 43350753 | C | T | 0,433625 | 0,051841201 | 0,007916384 | 5,8E-11 | Diuretics | GCST007927 | 132367 | NA | NA | NA | 0,000323874 | 42,88345721 |
| 48 | 17 | rs5417 | 7185062 | C | A | 0,426897 | -0,051768025 | 0,007955829 | 7,7E-11 | Diuretics | GCST007927 | 132367 | NA | NA | NA | 0,000319767 | 42,33950224 |
| 49 | 4 | rs300934 | 144171748 | T | G | 0,312059 | 0,05524486 | 0,00850054 | 8,1E-11 | Diuretics | GCST007927 | 132367 | NA | NA | NA | 0,000318987 | 42,23613724 |
| 50 | 11 | rs2202454 | 49011009 | C | T | 0,140301 | -0,073486174 | 0,011314722 | 8,3E-11 | Diuretics | GCST007927 | 132367 | NA | NA | NA | 0,00031857 | 42,18100619 |
| 51 | 10 | rs145158522 | 106629859 | T | C | 0,0120438 | 0,233760735 | 0,036073234 | 9,2E-11 | Diuretics | GCST007927 | 132367 | NA | NA | NA | 0,000317143 | 41,9919857 |
| 52 | 14 | rs72681869 | 50655357 | C | G | 0,0111769 | -0,241093199 | 0,03744182 | 1,2E-10 | Diuretics | GCST007927 | 132367 | NA | NA | NA | 0,000313141 | 41,46192526 |
| 53 | 4 | rs6536076 | 156420605 | A | T | 0,307892 | 0,054945583 | 0,008558846 | 1,4E-10 | Diuretics | GCST007927 | 132367 | NA | NA | NA | 0,000311258 | 41,21246638 |
| 54 | 20 | rs6021247 | 50108980 | G | A | 0,472054 | -0,050186094 | 0,007886958 | 2E-10 | Diuretics | GCST007927 | 132367 | NA | NA | NA | 0,000305798 | 40,48938927 |
| 55 | 12 | rs2024385 | 12888438 | A | T | 0,427823 | -0,050581401 | 0,007969838 | 2,2E-10 | Diuretics | GCST007927 | 132367 | NA | NA | NA | 0,000304208 | 40,27876479 |
| 56 | 13 | rs866573 | 30157252 | T | C | 0,149412 | 0,069751227 | 0,01101294 | 2,4E-10 | Diuretics | GCST007927 | 132367 | NA | NA | NA | 0,00030296 | 40,11350557 |
| 57 | 11 | rs10832573 | 16273208 | C | T | 0,203654 | 0,061532356 | 0,009734904 | 2,6E-10 | Diuretics | GCST007927 | 132367 | NA | NA | NA | 0,00030174 | 39,95187345 |
| 58 | 19 | rs113701136 | 30277729 | T | C | 0,318913 | 0,053559701 | 0,008515879 | 3,2E-10 | Diuretics | GCST007927 | 132367 | NA | NA | NA | 0,00029875 | 39,55585025 |
| 59 | 15 | rs28440013 | 85939155 | A | T | 0,286 | 0,055449518 | 0,008835271 | 3,5E-10 | Diuretics | GCST007927 | 132367 | NA | NA | NA | 0,000297473 | 39,38667005 |
| 60 | 17 | rs1436138 | 75316880 | G | A | 0,359306 | -0,051573776 | 0,00826129 | 4,3E-10 | Diuretics | GCST007927 | 132367 | NA | NA | NA | 0,000294344 | 38,97226077 |
| 61 | 16 | rs154656 | 89708003 | A | T | 0,425003 | -0,04966361 | 0,00797469 | 4,7E-10 | Diuretics | GCST007927 | 132367 | NA | NA | NA | 0,000292915 | 38,78308443 |
| 62 | 11 | rs7110547 | 32447347 | G | C | 0,380033 | 0,050580227 | 0,008138025 | 5,1E-10 | Diuretics | GCST007927 | 132367 | NA | NA | NA | 0,000291754 | 38,62930587 |
| 63 | 1 | rs12058002 | 115840517 | G | C | 0,0204073 | 0,17174667 | 0,027738925 | 6E-10 | Diuretics | GCST007927 | 132367 | NA | NA | NA | 0,000289529 | 38,33459166 |
| 64 | 10 | rs11199835 | 122977157 | G | A | 0,319005 | 0,051952882 | 0,008433155 | 7,2E-10 | Diuretics | GCST007927 | 132367 | NA | NA | NA | 0,000286639 | 37,95181376 |
| 65 | 16 | rs62046579 | 81538463 | C | A | 0,257233 | -0,05542917 | 0,009023024 | 8,1E-10 | Diuretics | GCST007927 | 132367 | NA | NA | NA | 0,000285016 | 37,73687914 |
| 66 | 4 | rs13112725 | 106911742 | G | C | 0,240779 | -0,056514365 | 0,009202481 | 8,2E-10 | Diuretics | GCST007927 | 132367 | NA | NA | NA | 0,000284842 | 37,71388659 |
| 67 | 4 | rs28702684 | 38395515 | G | C | 0,498848 | 0,04827632 | 0,007863087 | 8,3E-10 | Diuretics | GCST007927 | 132367 | NA | NA | NA | 0,000284694 | 37,69428825 |
| 68 | 5 | rs2984644 | 157482258 | A | G | 0,268206 | 0,054463326 | 0,008881524 | 8,7E-10 | Diuretics | GCST007927 | 132367 | NA | NA | NA | 0,000284008 | 37,60336782 |
| 69 | 6 | rs198851 | 26104632 | T | G | 0,150702 | 0,066807363 | 0,010987504 | 1,2E-09 | Diuretics | GCST007927 | 132367 | NA | NA | NA | 0,000279222 | 36,96953474 |
| 70 | 13 | rs12428857 | 73811791 | T | C | 0,0495341 | 0,108705011 | 0,018154207 | 2,1E-09 | Diuretics | GCST007927 | 132367 | NA | NA | NA | 0,000270799 | 35,85403111 |
| 71 | 8 | rs7011889 | 144005260 | C | A | 0,444985 | -0,047267039 | 0,00796209 | 2,9E-09 | Diuretics | GCST007927 | 132367 | NA | NA | NA | 0,000266175 | 35,2416366 |
| 72 | 10 | rs7076938 | 115789375 | C | T | 0,263878 | -0,053165881 | 0,008969648 | 3,1E-09 | Diuretics | GCST007927 | 132367 | NA | NA | NA | 0,000265351 | 35,13246557 |
| 73 | 18 | rs72915163 | 48792829 | T | C | 0,253582 | 0,054368549 | 0,009172663 | 3,1E-09 | Diuretics | GCST007927 | 132367 | NA | NA | NA | 0,000265344 | 35,13161186 |
| 74 | 12 | rs117913411 | 48254353 | A | T | 0,0324951 | 0,131361377 | 0,022249712 | 3,5E-09 | Diuretics | GCST007927 | 132367 | NA | NA | NA | 0,000263265 | 34,85620022 |
| 75 | 8 | rs13278931 | 30879207 | T | G | 0,443525 | 0,047323154 | 0,008009674 | 3,5E-09 | Diuretics | GCST007927 | 132367 | NA | NA | NA | 0,000263647 | 34,90688719 |
| 76 | 21 | rs2229742 | 16339172 | C | G | 0,10298 | 0,076235633 | 0,012948072 | 3,9E-09 | Diuretics | GCST007927 | 132367 | NA | NA | NA | 0,000261826 | 34,66564403 |
| 77 | 20 | rs6073354 | 42794270 | C | T | 0,499418 | -0,047011589 | 0,007988621 | 0,000000004 | Diuretics | GCST007927 | 132367 | NA | NA | NA | 0,000261561 | 34,63056972 |
| 78 | 15 | rs8041380 | 85645452 | C | G | 0,133447 | -0,06845347 | 0,011634661 | 0,000000004 | Diuretics | GCST007927 | 132367 | NA | NA | NA | 0,000261451 | 34,61599957 |
| 79 | 20 | rs6091153 | 49015311 | C | T | 0,227994 | 0,055956741 | 0,009531107 | 4,3E-09 | Diuretics | GCST007927 | 132367 | NA | NA | NA | 0,000260331 | 34,46763879 |
| 80 | 11 | rs10789588 | 107128909 | G | A | 0,432521 | -0,046345492 | 0,007950585 | 5,6E-09 | Diuretics | GCST007927 | 132367 | NA | NA | NA | 0,000256641 | 33,97897091 |
| 81 | 17 | rs3785837 | 59468942 | G | A | 0,228623 | -0,055156658 | 0,009527429 | 7,1E-09 | Diuretics | GCST007927 | 132367 | NA | NA | NA | 0,000253136 | 33,51489604 |
| 82 | 10 | rs10995307 | 64552242 | C | T | 0,500042 | -0,0455296 | 0,007890792 | 7,9E-09 | Diuretics | GCST007927 | 132367 | NA | NA | NA | 0,000251453 | 33,29199866 |
| 83 | 20 | rs1407256 | 31213647 | G | A | 0,145224 | 0,064974991 | 0,011292261 | 8,7E-09 | Diuretics | GCST007927 | 132367 | NA | NA | NA | 0,000250059 | 33,10732633 |
| 84 | 4 | rs13134008 | 111425895 | G | A | 0,425252 | -0,045851183 | 0,007971795 | 8,8E-09 | Diuretics | GCST007927 | 132367 | NA | NA | NA | 0,000249862 | 33,08128084 |
| 85 | 11 | rs11229252 | 55129640 | T | C | 0,109453 | -0,071722908 | 0,012574209 | 0,000000012 | Diuretics | GCST007927 | 132367 | NA | NA | NA | 0,000245736 | 32,53477904 |
| 86 | 16 | rs62039768 | 51560761 | A | C | 0,0954804 | 0,07656379 | 0,013471417 | 0,000000013 | Diuretics | GCST007927 | 132367 | NA | NA | NA | 0,000243969 | 32,30083529 |
| 87 | 2 | rs7599224 | 145214607 | G | T | 0,446085 | 0,045817295 | 0,008064615 | 0,000000013 | Diuretics | GCST007927 | 132367 | NA | NA | NA | 0,000243784 | 32,27639988 |
| 88 | 2 | rs12463645 | 135674370 | C | T | 0,364349 | -0,046416946 | 0,008193592 | 0,000000015 | Diuretics | GCST007927 | 132367 | NA | NA | NA | 0,000242393 | 32,09208154 |
| 89 | 3 | rs2421647 | 169113110 | C | A | 0,463343 | 0,044606174 | 0,007884923 | 0,000000015 | Diuretics | GCST007927 | 132367 | NA | NA | NA | 0,000241719 | 32,00283951 |
| 90 | 12 | rs11066309 | 112883476 | A | G | 0,407665 | 0,04528675 | 0,008029396 | 0,000000017 | Diuretics | GCST007927 | 132367 | NA | NA | NA | 0,000240266 | 31,81046072 |
| 91 | 2 | rs268263 | 164954174 | T | A | 0,244695 | -0,05207466 | 0,009254369 | 0,000000018 | Diuretics | GCST007927 | 132367 | NA | NA | NA | 0,000239153 | 31,66304374 |
| 92 | 11 | rs11246574 | 51457277 | A | G | 0,11677 | -0,068452061 | 0,012227895 | 0,000000022 | Diuretics | GCST007927 | 132367 | NA | NA | NA | 0,000236694 | 31,33740868 |
| 93 | 11 | rs80257854 | 50404319 | G | A | 0,128966 | -0,065886599 | 0,011769196 | 0,000000022 | Diuretics | GCST007927 | 132367 | NA | NA | NA | 0,000236711 | 31,339644 |
| 94 | 4 | rs7688323 | 156616621 | G | A | 0,204893 | -0,054884224 | 0,009849325 | 0,000000025 | Diuretics | GCST007927 | 132367 | NA | NA | NA | 0,000234531 | 31,05099834 |
| 95 | 11 | rs72910057 | 46331362 | T | G | 0,11283 | 0,068998102 | 0,012405787 | 0,000000027 | Diuretics | GCST007927 | 132367 | NA | NA | NA | 0,000233639 | 30,93278939 |
| 96 | 12 | rs10444491 | 121876558 | C | T | 0,394125 | -0,044955619 | 0,008101946 | 0,000000029 | Diuretics | GCST007927 | 132367 | NA | NA | NA | 0,000232546 | 30,78808659 |
| 97 | 6 | rs3778546 | 1901387 | T | C | 0,284027 | 0,048194926 | 0,00874652 | 0,000000036 | Diuretics | GCST007927 | 132367 | NA | NA | NA | 0,000229326 | 30,36165481 |
| 98 | 12 | rs61926181 | 50767037 | A | G | 0,0385937 | -0,112539117 | 0,020435418 | 0,000000036 | Diuretics | GCST007927 | 132367 | NA | NA | NA | 0,000229066 | 30,32727478 |
| 99 | 11 | rs557675 | 65566719 | G | T | 0,47008 | -0,043455708 | 0,00789721 | 0,000000037 | Diuretics | GCST007927 | 132367 | NA | NA | NA | 0,000228701 | 30,27887392 |
| 100 | 13 | rs303949 | 72365326 | C | A | 0,091547 | 0,075260241 | 0,013694779 | 0,000000039 | Diuretics | GCST007927 | 132367 | NA | NA | NA | 0,000228109 | 30,20053049 |
| 101 | 11 | rs490445 | 30565144 | C | G | 0,42422 | -0,043681418 | 0,007979699 | 0,000000044 | Diuretics | GCST007927 | 132367 | NA | NA | NA | 0,00022633 | 29,96497057 |
| 102 | 3 | rs6442105 | 48182326 | A | G | 0,332355 | -0,045588923 | 0,008339005 | 0,000000046 | Diuretics | GCST007927 | 132367 | NA | NA | NA | 0,000225742 | 29,8870918 |

**Table S14.** SNPs selection for drugs affecting bone structure and mineralization

|  | chr.exposure | SNP | pos.exposure | effect_allele.exposure | other_allele.exposure | eaf.exposure | beta.exposure | se.exposure | pval.exposure | exposure | id.exposure | samplesize.exposure | mr_keep.exposure | pval_origin.exposure | data_source.exposure | r2 | F |
| --- | --- | --- | --- | --- | --- | --- | --- | --- | --- | --- | --- | --- | --- | --- | --- | --- | --- |
| 1 | 13 | rs9594738 | 42952145 | T | C | 0,48902 | 0,125975042 | 0,015974856 | 3,1E-15 | Drugs affecting bone structure and mineralization | GCST007935 | 132367 | NA | NA | NA | 0,000469582 | 62,18542111 |
| 2 | 7 | rs3779381 | 120966790 | G | A | 0,258819 | -0,113869923 | 0,018283648 | 4,7E-10 | Drugs affecting bone structure and mineralization | GCST007935 | 132367 | NA | NA | NA | 0,000292945 | 38,78696329 |
| 3 | 18 | rs7230704 | 77198331 | A | G | 0,221148 | -0,119432003 | 0,019365489 | 6,9E-10 | Drugs affecting bone structure and mineralization | GCST007935 | 132367 | NA | NA | NA | 0,000287263 | 38,03451839 |
| 4 | 3 | rs442115 | 41118450 | T | G | 0,438769 | 0,097873963 | 0,016079607 | 1,2E-09 | Drugs affecting bone structure and mineralization | GCST007935 | 132367 | NA | NA | NA | 0,000279822 | 37,04903811 |
| 5 | 11 | rs11228240 | 68218290 | T | C | 0,276044 | 0,108600224 | 0,017921129 | 1,4E-09 | Drugs affecting bone structure and mineralization | GCST007935 | 132367 | NA | NA | NA | 0,000277352 | 36,72181641 |
| 6 | 1 | rs17130567 | 68663847 | G | A | 0,254436 | -0,109555584 | 0,018352051 | 2,4E-09 | Drugs affecting bone structure and mineralization | GCST007935 | 132367 | NA | NA | NA | 0,000269155 | 35,63635488 |
| 7 | 4 | rs899040 | 88775160 | T | A | 0,338096 | -0,096350051 | 0,016881751 | 0,000000011 | Drugs affecting bone structure and mineralization | GCST007935 | 132367 | NA | NA | NA | 0,000246027 | 32,57334373 |
| 8 | 1 | rs6684375 | 22706434 | T | C | 0,175671 | -0,118161792 | 0,020956325 | 0,000000017 | Drugs affecting bone structure and mineralization | GCST007935 | 132367 | NA | NA | NA | 0,000240126 | 31,791961 |
| 9 | 1 | rs9661787 | 240592636 | G | C | 0,183508 | -0,117555128 | 0,020837268 | 0,000000017 | Drugs affecting bone structure and mineralization | GCST007935 | 132367 | NA | NA | NA | 0,000240391 | 31,82695213 |
| 10 | 6 | rs9268500 | 32376517 | T | C | 0,0508279 | 0,203065151 | 0,036352049 | 0,000000023 | Drugs affecting bone structure and mineralization | GCST007935 | 132367 | NA | NA | NA | 0,000235684 | 31,2037259 |
| 11 | 11 | rs10832519 | 15811930 | C | T | 0,0308714 | -0,251403717 | 0,046123518 | 0,00000005 | Drugs affecting bone structure and mineralization | GCST007935 | 132367 | NA | NA | NA | 0,000224399 | 29,70927035 |

**Table S15.** SNPs selection for drugs for peptic ulcer and gastro-oesophageal reflux disease

|  | chr.exposure | SNP | pos.exposure | effect_allele.exposure | other_allele.exposure | eaf.exposure | beta.exposure | se.exposure | pval.exposure | exposure | id.exposure | samplesize.exposure | mr_keep.exposure | pval_origin.exposure | data_source.exposure | r2 | F |
| --- | --- | --- | --- | --- | --- | --- | --- | --- | --- | --- | --- | --- | --- | --- | --- | --- | --- |
| 1 | 6 | rs1619179 | 31175946 | C | A | 0,154861 | -0,070056871 | 0,010626536 | 4,3E-11 | Drugs for peptic ulcer and gastro-oesophageal reflux disease (GORD) | GCST007922 | 132367 | NA | NA | NA | 0,000328243 | 43,46217884 |
| 2 | 7 | rs6965956 | 21610379 | A | G | 0,4279 | 0,050471478 | 0,007786131 | 9E-11 | Drugs for peptic ulcer and gastro-oesophageal reflux disease (GORD) | GCST007922 | 132367 | NA | NA | NA | 0,000317345 | 42,01864242 |
| 3 | 12 | rs11171710 | 56368078 | A | G | 0,447089 | 0,046545742 | 0,007827957 | 2,7E-09 | Drugs for peptic ulcer and gastro-oesophageal reflux disease (GORD) | GCST007922 | 132367 | NA | NA | NA | 0,000267035 | 35,35546481 |
| 4 | 11 | rs3858461 | 32494962 | C | T | 0,448978 | -0,044241407 | 0,007762368 | 0,000000012 | Drugs for peptic ulcer and gastro-oesophageal reflux disease (GORD) | GCST007922 | 132367 | NA | NA | NA | 0,000245348 | 32,48350139 |
| 5 | 19 | rs2051815 | 18835115 | A | G | 0,320032 | -0,04516697 | 0,008278086 | 0,000000049 | Drugs for peptic ulcer and gastro-oesophageal reflux disease (GORD) | GCST007922 | 132367 | NA | NA | NA | 0,000224856 | 29,76976747 |

**Table S16.** SNPs selection for drugs used in diabetes

|  | chr.exposure | SNP | pos.exposure | effect_allele.exposure | other_allele.exposure | eaf.exposure | beta.exposure | se.exposure | pval.exposure | exposure | id.exposure | samplesize.exposure | mr_keep.exposure | pval_origin.exposure | data_source.exposure | r2 | F |
| --- | --- | --- | --- | --- | --- | --- | --- | --- | --- | --- | --- | --- | --- | --- | --- | --- | --- |
| 1 | 10 | rs7903146 | 114758349 | T | C | 0,291287 | 0,327302553 | 0,012760456 | 4,3E-145 | Drugs used in diabetes | GCST007923 | 132367 | NA | NA | NA | 0,004945764 | 657,8998876 |
| 2 | 6 | rs9273364 | 32626302 | G | T | 0,302909 | 0,231936996 | 0,012629949 | 2,5E-75 | Drugs used in diabetes | GCST007923 | 132367 | NA | NA | NA | 0,002541278 | 337,2332055 |
| 3 | 16 | rs56094641 | 53806453 | G | A | 0,404982 | 0,117936656 | 0,01181671 | 1,9E-23 | Drugs used in diabetes | GCST007923 | 132367 | NA | NA | NA | 0,000751966 | 99,60884406 |
| 4 | 9 | rs10965246 | 22132698 | C | T | 0,17563 | -0,143406968 | 0,015294879 | 6,8E-21 | Drugs used in diabetes | GCST007923 | 132367 | NA | NA | NA | 0,000663713 | 87,91071825 |
| 5 | 10 | rs697238 | 80947668 | T | G | 0,420872 | 0,108555698 | 0,01176904 | 2,9E-20 | Drugs used in diabetes | GCST007923 | 132367 | NA | NA | NA | 0,000642339 | 85,07787093 |
| 6 | 4 | rs10755148 | 6295064 | A | G | 0,359115 | -0,111188202 | 0,012095099 | 3,8E-20 | Drugs used in diabetes | GCST007923 | 132367 | NA | NA | NA | 0,000638031 | 84,50687308 |
| 7 | 3 | rs9854769 | 185520948 | G | A | 0,31516 | 0,112895556 | 0,012502245 | 1,7E-19 | Drugs used in diabetes | GCST007923 | 132367 | NA | NA | NA | 0,000615645 | 81,54007941 |
| 8 | 6 | rs7766070 | 20686573 | A | C | 0,265183 | 0,11792 | 0,013141332 | 2,9E-19 | Drugs used in diabetes | GCST007923 | 132367 | NA | NA | NA | 0,000607928 | 80,51736959 |
| 9 | 12 | rs76895963 | 4384844 | G | T | 0,0141213 | -0,442334936 | 0,049928609 | 8E-19 | Drugs used in diabetes | GCST007923 | 132367 | NA | NA | NA | 0,000592606 | 78,48686716 |
| 10 | 10 | rs1112718 | 94479107 | G | A | 0,405782 | -0,101585444 | 0,01183558 | 9,2E-18 | Drugs used in diabetes | GCST007923 | 132367 | NA | NA | NA | 0,00055624 | 73,6677352 |
| 11 | 3 | rs1496653 | 23454790 | G | A | 0,20471 | -0,115987473 | 0,014375717 | 7,1E-16 | Drugs used in diabetes | GCST007923 | 132367 | NA | NA | NA | 0,000491552 | 65,09634383 |
| 12 | 13 | rs1359790 | 80717156 | A | G | 0,288114 | -0,102855312 | 0,012846225 | 1,2E-15 | Drugs used in diabetes | GCST007923 | 132367 | NA | NA | NA | 0,000484075 | 64,10558515 |
| 13 | 5 | rs116782923 | 102331465 | T | A | 0,052571 | 0,207473217 | 0,02604948 | 1,7E-15 | Drugs used in diabetes | GCST007923 | 132367 | NA | NA | NA | 0,000479003 | 63,43360799 |
| 14 | 2 | rs77059113 | 43672508 | G | T | 0,0734371 | -0,175301046 | 0,022359209 | 4,5E-15 | Drugs used in diabetes | GCST007923 | 132367 | NA | NA | NA | 0,000464168 | 61,46807194 |
| 15 | 8 | rs3802177 | 118185025 | A | G | 0,309928 | -0,095131107 | 0,012563303 | 3,7E-14 | Drugs used in diabetes | GCST007923 | 132367 | NA | NA | NA | 0,000432982 | 57,33645789 |
| 16 | 9 | rs7018475 | 22137685 | G | T | 0,257707 | 0,10020067 | 0,013282867 | 4,6E-14 | Drugs used in diabetes | GCST007923 | 132367 | NA | NA | NA | 0,000429726 | 56,90508068 |
| 17 | 9 | rs9410573 | 84311800 | C | T | 0,421202 | -0,087119321 | 0,011811566 | 1,6E-13 | Drugs used in diabetes | GCST007923 | 132367 | NA | NA | NA | 0,000410824 | 54,4010723 |
| 18 | 11 | rs4930011 | 2856658 | G | C | 0,389262 | 0,087292628 | 0,01200349 | 3,5E-13 | Drugs used in diabetes | GCST007923 | 132367 | NA | NA | NA | 0,00039938 | 52,8851205 |
| 19 | 20 | rs1800961 | 43042364 | T | C | 0,0308713 | 0,237393444 | 0,033523211 | 1,4E-12 | Drugs used in diabetes | GCST007923 | 132367 | NA | NA | NA | 0,000378706 | 50,14638738 |
| 20 | 2 | rs2972155 | 227117698 | C | G | 0,346629 | -0,086238024 | 0,012217194 | 1,7E-12 | Drugs used in diabetes | GCST007923 | 132367 | NA | NA | NA | 0,00037628 | 49,82509027 |
| 21 | 3 | rs11720108 | 123069058 | T | C | 0,247134 | -0,093975934 | 0,013475255 | 3,1E-12 | Drugs used in diabetes | GCST007923 | 132367 | NA | NA | NA | 0,000367299 | 48,63542737 |
| 22 | 10 | rs11257655 | 12307894 | T | C | 0,208417 | 0,099439972 | 0,014271732 | 3,2E-12 | Drugs used in diabetes | GCST007923 | 132367 | NA | NA | NA | 0,000366631 | 48,54696088 |
| 23 | 7 | rs849140 | 28183702 | T | C | 0,411436 | 0,081292854 | 0,011819008 | 6,1E-12 | Drugs used in diabetes | GCST007923 | 132367 | NA | NA | NA | 0,000357279 | 47,30816653 |
| 24 | 2 | rs780093 | 27742603 | T | C | 0,383979 | -0,081971326 | 0,01192995 | 6,4E-12 | Drugs used in diabetes | GCST007923 | 132367 | NA | NA | NA | 0,000356543 | 47,21066185 |
| 25 | 19 | rs112972879 | 46165082 | A | G | 0,361546 | -0,083148637 | 0,012265265 | 1,2E-11 | Drugs used in diabetes | GCST007923 | 132367 | NA | NA | NA | 0,000347077 | 45,95680824 |
| 26 | 17 | rs7501939 | 36101156 | T | C | 0,397921 | 0,080408395 | 0,011856895 | 1,2E-11 | Drugs used in diabetes | GCST007923 | 132367 | NA | NA | NA | 0,00034732 | 45,98902941 |
| 27 | 11 | rs77464186 | 72460398 | C | A | 0,156347 | -0,105954187 | 0,015973225 | 3,3E-11 | Drugs used in diabetes | GCST007923 | 132367 | NA | NA | NA | 0,000332297 | 43,99916916 |
| 28 | 15 | rs12910361 | 77782335 | A | G | 0,28639 | -0,083818465 | 0,012831087 | 6,5E-11 | Drugs used in diabetes | GCST007923 | 132367 | NA | NA | NA | 0,00032228 | 42,67229213 |
| 29 | 3 | rs17036160 | 12329783 | T | C | 0,116465 | -0,11809141 | 0,018121775 | 7,2E-11 | Drugs used in diabetes | GCST007923 | 132367 | NA | NA | NA | 0,000320713 | 42,4647516 |
| 30 | 2 | rs10184004 | 165508389 | T | C | 0,405503 | -0,076864867 | 0,011829002 | 8,1E-11 | Drugs used in diabetes | GCST007923 | 132367 | NA | NA | NA | 0,00031889 | 42,22337996 |
| 31 | 6 | rs11759026 | 126792095 | G | A | 0,226203 | 0,090316867 | 0,013935279 | 9,1E-11 | Drugs used in diabetes | GCST007923 | 132367 | NA | NA | NA | 0,000317241 | 42,00488529 |
| 32 | 1 | rs79687284 | 214150821 | C | G | 0,0346315 | 0,199636214 | 0,031752396 | 3,2E-10 | Drugs used in diabetes | GCST007923 | 132367 | NA | NA | NA | 0,000298549 | 39,52929732 |
| 33 | 5 | rs145510090 | 101273694 | A | T | 0,0500239 | 0,16795466 | 0,026805539 | 3,7E-10 | Drugs used in diabetes | GCST007923 | 132367 | NA | NA | NA | 0,000296501 | 39,25802558 |
| 34 | 5 | rs464605 | 55807370 | C | T | 0,253257 | -0,086158117 | 0,013943375 | 6,4E-10 | Drugs used in diabetes | GCST007923 | 132367 | NA | NA | NA | 0,000288371 | 38,18123663 |
| 35 | 6 | rs4715207 | 50809278 | T | C | 0,179898 | 0,09093272 | 0,015124692 | 1,8E-09 | Drugs used in diabetes | GCST007923 | 132367 | NA | NA | NA | 0,000273004 | 36,14603847 |
| 36 | 7 | rs1974619 | 15065300 | C | T | 0,449969 | -0,069310806 | 0,011680278 | 0,000000003 | Drugs used in diabetes | GCST007923 | 132367 | NA | NA | NA | 0,00026595 | 35,21186256 |
| 37 | 12 | rs2258238 | 66221060 | T | A | 0,104567 | 0,112517315 | 0,019022363 | 3,3E-09 | Drugs used in diabetes | GCST007923 | 132367 | NA | NA | NA | 0,00026425 | 34,98672003 |
| 38 | 18 | rs9957264 | 56881633 | A | C | 0,16477 | -0,092831513 | 0,015698315 | 3,3E-09 | Drugs used in diabetes | GCST007923 | 132367 | NA | NA | NA | 0,000264113 | 34,96859751 |
| 39 | 1 | rs2476601 | 114377568 | A | G | 0,102882 | 0,110628642 | 0,019065163 | 6,5E-09 | Drugs used in diabetes | GCST007923 | 132367 | NA | NA | NA | 0,00025431 | 33,67034483 |
| 40 | 14 | rs8017808 | 38848419 | T | G | 0,242765 | -0,079489992 | 0,013695558 | 6,5E-09 | Drugs used in diabetes | GCST007923 | 132367 | NA | NA | NA | 0,000254434 | 33,68673031 |
| 41 | 16 | rs72802358 | 75243657 | C | G | 0,1004 | -0,112404307 | 0,019383779 | 6,7E-09 | Drugs used in diabetes | GCST007923 | 132367 | NA | NA | NA | 0,00025398 | 33,62656046 |
| 42 | 17 | rs668799 | 40716235 | T | C | 0,277947 | 0,075102695 | 0,012985714 | 7,3E-09 | Drugs used in diabetes | GCST007923 | 132367 | NA | NA | NA | 0,000252633 | 33,44820499 |
| 43 | 11 | rs5213 | 17408404 | C | T | 0,347606 | 0,070679557 | 0,012247871 | 7,9E-09 | Drugs used in diabetes | GCST007923 | 132367 | NA | NA | NA | 0,000251523 | 33,30120043 |
| 44 | 14 | rs2268967 | 69430370 | A | G | 0,228056 | 0,08002151 | 0,013956616 | 9,8E-09 | Drugs used in diabetes | GCST007923 | 132367 | NA | NA | NA | 0,000248294 | 32,87355462 |
| 45 | 15 | rs11070332 | 41809205 | A | G | 0,347665 | 0,07004768 | 0,012274731 | 0,000000012 | Drugs used in diabetes | GCST007923 | 132367 | NA | NA | NA | 0,000245967 | 32,56544104 |
| 46 | 6 | rs3383 | 143073041 | T | C | 0,365922 | -0,067971362 | 0,012099948 | 0,000000019 | Drugs used in diabetes | GCST007923 | 132367 | NA | NA | NA | 0,000238343 | 31,55574165 |
| 47 | 10 | rs61123794 | 114818042 | T | C | 0,0193324 | -0,236103125 | 0,042184163 | 0,000000022 | Drugs used in diabetes | GCST007923 | 132367 | NA | NA | NA | 0,000236604 | 31,32550153 |
| 48 | 1 | rs2293476 | 40036847 | C | G | 0,228804 | 0,077206 | 0,013822208 | 0,000000023 | Drugs used in diabetes | GCST007923 | 132367 | NA | NA | NA | 0,000235649 | 31,19900262 |
| 49 | 2 | rs2723063 | 65280185 | G | A | 0,411067 | -0,065654269 | 0,011799927 | 0,000000026 | Drugs used in diabetes | GCST007923 | 132367 | NA | NA | NA | 0,000233822 | 30,95713101 |
| 50 | 8 | rs515071 | 41519462 | A | G | 0,238099 | -0,075833455 | 0,01364169 | 0,000000027 | Drugs used in diabetes | GCST007923 | 132367 | NA | NA | NA | 0,000233402 | 30,90144545 |
| 51 | 6 | rs9379084 | 7231843 | A | G | 0,104171 | -0,10827128 | 0,019512389 | 0,000000029 | Drugs used in diabetes | GCST007923 | 132367 | NA | NA | NA | 0,000232555 | 30,78924768 |
| 52 | 12 | rs3217792 | 4384696 | T | C | 0,0776632 | -0,121524672 | 0,022002052 | 0,000000033 | Drugs used in diabetes | GCST007923 | 132367 | NA | NA | NA | 0,000230421 | 30,50675298 |
| 53 | 15 | rs28678152 | 90449523 | T | C | 0,262906 | 0,073226251 | 0,013281349 | 0,000000035 | Drugs used in diabetes | GCST007923 | 132367 | NA | NA | NA | 0,000229599 | 30,39783546 |
| 54 | 19 | rs7409148 | 13094298 | C | T | 0,185829 | -0,082887832 | 0,015064625 | 0,000000038 | Drugs used in diabetes | GCST007923 | 132367 | NA | NA | NA | 0,000228658 | 30,27320164 |
| 55 | 11 | rs1056387 | 47681370 | G | T | 0,450353 | -0,064100826 | 0,011663284 | 0,000000039 | Drugs used in diabetes | GCST007923 | 132367 | NA | NA | NA | 0,000228143 | 30,20500849 |
| 56 | 11 | rs231361 | 2691500 | A | G | 0,250479 | 0,073669429 | 0,013429525 | 0,000000041 | Drugs used in diabetes | GCST007923 | 132367 | NA | NA | NA | 0,000227287 | 30,09170202 |
| 57 | 6 | rs719728 | 127414618 | T | C | 0,291654 | -0,069789194 | 0,012796573 | 0,000000049 | Drugs used in diabetes | GCST007923 | 132367 | NA | NA | NA | 0,000224653 | 29,74284286 |

**Table S17.** SNPs selection for glucocorticoids

|  | chr.exposure | SNP | pos.exposure | effect_allele.exposure | other_allele.exposure | eaf.exposure | beta.exposure | se.exposure | pval.exposure | exposure | id.exposure | samplesize.exposure | mr_keep.exposure | pval_origin.exposure | data_source.exposure | r2 | F |
| --- | --- | --- | --- | --- | --- | --- | --- | --- | --- | --- | --- | --- | --- | --- | --- | --- | --- |
| 1 | 6 | rs1391371 | 32603798 | T | A | 0,202145 | 0,204121963 | 0,013949579 | 1,7E-48 | Glucocorticoids | GCST007942 | 132367 | NA | NA | NA | 0,001615011 | 214,116777 |
| 2 | 9 | rs992969 | 6209697 | A | G | 0,251777 | 0,139871247 | 0,012898849 | 2,1E-27 | Glucocorticoids | GCST007942 | 132367 | NA | NA | NA | 0,000887543 | 117,5840531 |
| 3 | 10 | rs1775553 | 9054325 | T | C | 0,423019 | -0,117927194 | 0,011302505 | 1,7E-25 | Glucocorticoids | GCST007942 | 132367 | NA | NA | NA | 0,000821754 | 108,8609161 |
| 4 | 2 | rs34290285 | 242698640 | A | G | 0,255506 | -0,12759555 | 0,012807354 | 2,2E-23 | Glucocorticoids | GCST007942 | 132367 | NA | NA | NA | 0,000749285 | 99,25345784 |
| 5 | 11 | rs7936312 | 76293726 | T | G | 0,477022 | 0,098884502 | 0,011205948 | 1,1E-18 | Glucocorticoids | GCST007942 | 132367 | NA | NA | NA | 0,000587928 | 77,86686772 |
| 6 | 2 | rs2287037 | 102979028 | T | C | 0,395554 | 0,096040754 | 0,011432775 | 4,4E-17 | Glucocorticoids | GCST007942 | 132367 | NA | NA | NA | 0,00053284 | 70,56693957 |
| 7 | 5 | rs1898671 | 110408002 | T | C | 0,350684 | 0,097538431 | 0,011702287 | 7,8E-17 | Glucocorticoids | GCST007942 | 132367 | NA | NA | NA | 0,000524569 | 69,47099457 |
| 8 | 15 | rs72743461 | 67441750 | A | C | 0,236679 | 0,09897992 | 0,013141574 | 5E-14 | Glucocorticoids | GCST007942 | 132367 | NA | NA | NA | 0,000428384 | 56,72739763 |
| 9 | 6 | rs1504215 | 91006227 | A | G | 0,350757 | -0,086968465 | 0,011709394 | 1,1E-13 | Glucocorticoids | GCST007942 | 132367 | NA | NA | NA | 0,000416576 | 55,16304005 |
| 10 | 12 | rs1689510 | 56396768 | C | G | 0,338025 | 0,080533166 | 0,011856495 | 1,1E-11 | Glucocorticoids | GCST007942 | 132367 | NA | NA | NA | 0,000348422 | 46,13498027 |
| 11 | 8 | rs4739738 | 81291645 | G | A | 0,358014 | 0,078805425 | 0,011672134 | 1,5E-11 | Glucocorticoids | GCST007942 | 132367 | NA | NA | NA | 0,000344257 | 45,5832381 |
| 12 | 15 | rs1963497 | 61071791 | A | C | 0,182496 | -0,097599281 | 0,014568562 | 2,1E-11 | Glucocorticoids | GCST007942 | 132367 | NA | NA | NA | 0,000338948 | 44,88004582 |
| 13 | 17 | rs1011082 | 38068514 | C | T | 0,483207 | 0,07408 | 0,011180029 | 3,4E-11 | Glucocorticoids | GCST007942 | 132367 | NA | NA | NA | 0,000331583 | 43,90455286 |
| 14 | 1 | rs2949661 | 167424924 | T | C | 0,401994 | -0,074315371 | 0,011428852 | 7,9E-11 | Glucocorticoids | GCST007942 | 132367 | NA | NA | NA | 0,000319326 | 42,28102914 |
| 15 | 9 | rs2150968 | 6081298 | A | G | 0,310387 | -0,078262699 | 0,012102651 | 1E-10 | Glucocorticoids | GCST007942 | 132367 | NA | NA | NA | 0,000315814 | 41,81595796 |
| 16 | 3 | rs10154834 | 33068055 | C | T | 0,494958 | -0,067905188 | 0,011217121 | 1,4E-09 | Glucocorticoids | GCST007942 | 132367 | NA | NA | NA | 0,000276785 | 36,64684173 |
| 17 | 19 | rs117710327 | 33726578 | A | C | 0,0620109 | -0,141427443 | 0,023380492 | 1,5E-09 | Glucocorticoids | GCST007942 | 132367 | NA | NA | NA | 0,000276351 | 36,58925125 |
| 18 | 16 | rs35441874 | 11213021 | A | T | 0,246038 | -0,077133744 | 0,013065188 | 3,6E-09 | Glucocorticoids | GCST007942 | 132367 | NA | NA | NA | 0,000263247 | 34,85386248 |
| 19 | 1 | rs12123821 | 152179152 | T | C | 0,0470148 | 0,153392807 | 0,026356427 | 5,9E-09 | Glucocorticoids | GCST007942 | 132367 | NA | NA | NA | 0,000255827 | 33,87118379 |

**Table S18.** SNPs selection for HMG CoA reductase inhibitors

|  | chr.exposure | SNP | pos.exposure | effect_allele.exposure | other_allele.exposure | eaf.exposure | beta.exposure | se.exposure | pval.exposure | exposure | id.exposure | samplesize.exposure | mr_keep.exposure | pval_origin.exposure | data_source.exposure | r2 | F |
| --- | --- | --- | --- | --- | --- | --- | --- | --- | --- | --- | --- | --- | --- | --- | --- | --- | --- |
| 1 | 19 | rs7412 | 45412079 | T | C | 0,0792273 | -0,310311732 | 0,010360179 | 4,1E-197 | HMG CoA reductase inhibitors | GCST007931 | 132367 | NA | NA | NA | 0,006732069 | 897,1298357 |
| 2 | 1 | rs12740374 | 109817590 | T | G | 0,219778 | -0,156618273 | 0,006742468 | 2,3E-119 | HMG CoA reductase inhibitors | GCST007931 | 132367 | NA | NA | NA | 0,004059762 | 539,5609299 |
| 3 | 19 | rs6511720 | 11202306 | T | G | 0,117712 | -0,198647127 | 0,008684178 | 8,3E-116 | HMG CoA reductase inhibitors | GCST007931 | 132367 | NA | NA | NA | 0,00393744 | 523,2395316 |
| 4 | 11 | rs964184 | 116648917 | G | C | 0,133698 | 0,160718726 | 0,008218578 | 3,7E-85 | HMG CoA reductase inhibitors | GCST007931 | 132367 | NA | NA | NA | 0,002880759 | 382,4133035 |
| 5 | 2 | rs1367117 | 21263900 | A | G | 0,337599 | 0,105726091 | 0,005900949 | 8,7E-72 | HMG CoA reductase inhibitors | GCST007931 | 132367 | NA | NA | NA | 0,002419295 | 321,0066457 |
| 6 | 6 | rs74617384 | 160997118 | T | A | 0,0801433 | 0,181372313 | 0,010313884 | 3,2E-69 | HMG CoA reductase inhibitors | GCST007931 | 132367 | NA | NA | NA | 0,002330797 | 309,2366529 |
| 7 | 8 | rs2954021 | 126482077 | A | G | 0,495627 | 0,096635253 | 0,005595082 | 7,7E-67 | HMG CoA reductase inhibitors | GCST007931 | 132367 | NA | NA | NA | 0,002248542 | 298,2989463 |
| 8 | 1 | rs11591147 | 55505647 | T | G | 0,017203 | -0,362323999 | 0,021494841 | 9,4E-64 | HMG CoA reductase inhibitors | GCST007931 | 132367 | NA | NA | NA | 0,002141977 | 284,13135 |
| 9 | 2 | rs4299376 | 44072576 | G | T | 0,324311 | 0,086690463 | 0,005971001 | 9,2E-48 | HMG CoA reductase inhibitors | GCST007931 | 132367 | NA | NA | NA | 0,001589929 | 210,7860247 |
| 10 | 6 | rs140570886 | 161013013 | C | T | 0,0154611 | 0,307376865 | 0,022743813 | 1,3E-41 | HMG CoA reductase inhibitors | GCST007931 | 132367 | NA | NA | NA | 0,001377962 | 182,645612 |
| 11 | 1 | rs10889338 | 62984897 | T | C | 0,351107 | -0,074726134 | 0,005858093 | 2,9E-37 | HMG CoA reductase inhibitors | GCST007931 | 132367 | NA | NA | NA | 0,001227776 | 162,7143862 |
| 12 | 19 | rs58542926 | 19379549 | T | C | 0,074933 | -0,12420776 | 0,010639856 | 1,7E-31 | HMG CoA reductase inhibitors | GCST007931 | 132367 | NA | NA | NA | 0,001028488 | 136,2759933 |
| 13 | 9 | rs2519093 | 136141870 | T | C | 0,185023 | 0,083506924 | 0,007223517 | 6,5E-31 | HMG CoA reductase inhibitors | GCST007931 | 132367 | NA | NA | NA | 0,001008625 | 133,6413825 |
| 14 | 19 | rs116881820 | 45397952 | C | T | 0,025925 | 0,202944931 | 0,017623061 | 1,1E-30 | HMG CoA reductase inhibitors | GCST007931 | 132367 | NA | NA | NA | 0,001000873 | 132,6133265 |
| 15 | 10 | rs7903146 | 114758349 | T | C | 0,290995 | 0,070760069 | 0,006160627 | 1,6E-30 | HMG CoA reductase inhibitors | GCST007931 | 132367 | NA | NA | NA | 0,000995667 | 131,9228748 |
| 16 | 19 | rs2738447 | 11227480 | A | C | 0,406512 | -0,062571332 | 0,005699048 | 4,8E-28 | HMG CoA reductase inhibitors | GCST007931 | 132367 | NA | NA | NA | 0,000909853 | 120,5423264 |
| 17 | 2 | rs522761 | 21401789 | T | C | 0,245722 | -0,068290274 | 0,006491256 | 7E-26 | HMG CoA reductase inhibitors | GCST007931 | 132367 | NA | NA | NA | 0,000835444 | 110,6760609 |
| 18 | 2 | rs1260326 | 27730940 | T | C | 0,395882 | 0,059920058 | 0,005712328 | 9,7E-26 | HMG CoA reductase inhibitors | GCST007931 | 132367 | NA | NA | NA | 0,000830572 | 110,0301109 |
| 19 | 9 | rs1537371 | 22099568 | A | C | 0,499383 | 0,058189402 | 0,005604606 | 3E-25 | HMG CoA reductase inhibitors | GCST007931 | 132367 | NA | NA | NA | 0,0008137 | 107,7931402 |
| 20 | 5 | rs12916 | 74656539 | C | T | 0,40104 | 0,058663467 | 0,005712752 | 9,7E-25 | HMG CoA reductase inhibitors | GCST007931 | 132367 | NA | NA | NA | 0,000796011 | 105,4479645 |
| 21 | 20 | rs2207132 | 39142516 | A | G | 0,0335157 | 0,159389121 | 0,015550983 | 1,2E-24 | HMG CoA reductase inhibitors | GCST007931 | 132367 | NA | NA | NA | 0,000793008 | 105,0497709 |
| 22 | 8 | rs15285 | 19824667 | T | C | 0,28486 | -0,058676165 | 0,006203907 | 3,1E-21 | HMG CoA reductase inhibitors | GCST007931 | 132367 | NA | NA | NA | 0,000675336 | 89,45124316 |
| 23 | 16 | rs2287997 | 72140553 | A | G | 0,18536 | 0,064653304 | 0,007201719 | 2,8E-19 | HMG CoA reductase inhibitors | GCST007931 | 132367 | NA | NA | NA | 0,000608506 | 80,59397026 |
| 24 | 8 | rs1495741 | 18272881 | G | A | 0,221421 | 0,05763756 | 0,00673432 | 1,1E-17 | HMG CoA reductase inhibitors | GCST007931 | 132367 | NA | NA | NA | 0,0005531 | 73,2515979 |
| 25 | 19 | rs41289512 | 45351516 | G | C | 0,0428705 | 0,117883538 | 0,013792655 | 1,3E-17 | HMG CoA reductase inhibitors | GCST007931 | 132367 | NA | NA | NA | 0,000551558 | 73,04726979 |
| 26 | 12 | rs76895963 | 4384844 | G | T | 0,0141825 | -0,202726945 | 0,024010456 | 3,1E-17 | HMG CoA reductase inhibitors | GCST007931 | 132367 | NA | NA | NA | 0,00053828 | 71,2878582 |
| 27 | 1 | rs693668 | 55521109 | G | A | 0,350319 | -0,048941477 | 0,005866453 | 7,3E-17 | HMG CoA reductase inhibitors | GCST007931 | 132367 | NA | NA | NA | 0,000525527 | 69,59795283 |
| 28 | 20 | rs8126001 | 62711459 | T | C | 0,489093 | -0,046777285 | 0,005628309 | 9,5E-17 | HMG CoA reductase inhibitors | GCST007931 | 132367 | NA | NA | NA | 0,000521564 | 69,07288878 |
| 29 | 17 | rs77542162 | 67081278 | G | A | 0,0230779 | 0,151112025 | 0,018663359 | 5,6E-16 | HMG CoA reductase inhibitors | GCST007931 | 132367 | NA | NA | NA | 0,000495021 | 65,55591178 |
| 30 | 19 | rs601338 | 49206674 | G | A | 0,489712 | -0,044931922 | 0,005608468 | 1,1E-15 | HMG CoA reductase inhibitors | GCST007931 | 132367 | NA | NA | NA | 0,000484653 | 64,18225205 |
| 31 | 20 | rs6129760 | 39746403 | G | A | 0,324412 | 0,047792028 | 0,005979625 | 1,3E-15 | HMG CoA reductase inhibitors | GCST007931 | 132367 | NA | NA | NA | 0,000482363 | 63,8787555 |
| 32 | 12 | rs1169288 | 121416650 | C | A | 0,313809 | 0,048448524 | 0,006082533 | 1,6E-15 | HMG CoA reductase inhibitors | GCST007931 | 132367 | NA | NA | NA | 0,000479076 | 63,44326918 |
| 33 | 1 | rs79598313 | 27284913 | T | C | 0,0236446 | 0,146636976 | 0,018408443 | 1,6E-15 | HMG CoA reductase inhibitors | GCST007931 | 132367 | NA | NA | NA | 0,000479143 | 63,45214405 |
| 34 | 7 | rs4639414 | 21589243 | G | A | 0,230148 | 0,052494586 | 0,006676649 | 3,8E-15 | HMG CoA reductase inhibitors | GCST007931 | 132367 | NA | NA | NA | 0,000466798 | 61,8166342 |
| 35 | 16 | rs1421085 | 53800954 | C | T | 0,403509 | 0,044655313 | 0,005696826 | 4,6E-15 | HMG CoA reductase inhibitors | GCST007931 | 132367 | NA | NA | NA | 0,00046398 | 61,44319635 |
| 36 | 6 | rs750332 | 31607050 | C | T | 0,180658 | -0,055028614 | 0,007267379 | 3,7E-14 | HMG CoA reductase inhibitors | GCST007931 | 132367 | NA | NA | NA | 0,000432966 | 57,33435827 |
| 37 | 6 | rs9295123 | 160570114 | A | G | 0,389246 | -0,042763179 | 0,005750317 | 1E-13 | HMG CoA reductase inhibitors | GCST007931 | 132367 | NA | NA | NA | 0,000417633 | 55,3031422 |
| 38 | 2 | rs10804330 | 227185749 | C | T | 0,429504 | -0,041844942 | 0,005740635 | 3,1E-13 | HMG CoA reductase inhibitors | GCST007931 | 132367 | NA | NA | NA | 0,000401247 | 53,13241313 |
| 39 | 5 | rs1501908 | 156398169 | G | C | 0,364761 | -0,040749671 | 0,005813226 | 2,4E-12 | HMG CoA reductase inhibitors | GCST007931 | 132367 | NA | NA | NA | 0,000371085 | 49,13684126 |
| 40 | 19 | rs79701229 | 45384931 | A | G | 0,012772 | 0,17124922 | 0,024910228 | 6,2E-12 | HMG CoA reductase inhibitors | GCST007931 | 132367 | NA | NA | NA | 0,000356917 | 47,26016524 |
| 41 | 6 | rs998584 | 43757896 | A | C | 0,482799 | 0,038632303 | 0,005618626 | 6,2E-12 | HMG CoA reductase inhibitors | GCST007931 | 132367 | NA | NA | NA | 0,000357031 | 47,27530202 |
| 42 | 20 | rs2618567 | 17844492 | G | T | 0,340873 | 0,040058837 | 0,005911214 | 1,2E-11 | HMG CoA reductase inhibitors | GCST007931 | 132367 | NA | NA | NA | 0,000346827 | 45,92369313 |
| 43 | 11 | rs3802932 | 63988045 | A | G | 0,0581295 | 0,08027365 | 0,011960626 | 1,9E-11 | HMG CoA reductase inhibitors | GCST007931 | 132367 | NA | NA | NA | 0,000340182 | 45,04345131 |
| 44 | 9 | rs1883025 | 107664301 | T | C | 0,254856 | -0,042749952 | 0,006416125 | 2,7E-11 | HMG CoA reductase inhibitors | GCST007931 | 132367 | NA | NA | NA | 0,000335274 | 44,39346736 |
| 45 | 7 | rs3918226 | 150690176 | T | C | 0,0779394 | 0,068522545 | 0,01051933 | 7,3E-11 | HMG CoA reductase inhibitors | GCST007931 | 132367 | NA | NA | NA | 0,000320459 | 42,43109842 |
| 46 | 3 | rs4518111 | 12377344 | A | C | 0,433772 | 0,037062173 | 0,005718625 | 9,1E-11 | HMG CoA reductase inhibitors | GCST007931 | 132367 | NA | NA | NA | 0,00031722 | 42,00219821 |
| 47 | 8 | rs11787365 | 145041333 | C | T | 0,400918 | 0,037016407 | 0,005727778 | 1E-10 | HMG CoA reductase inhibitors | GCST007931 | 132367 | NA | NA | NA | 0,000315427 | 41,76472433 |
| 48 | 7 | rs972283 | 130466854 | A | G | 0,488765 | -0,035923041 | 0,00559905 | 1,4E-10 | HMG CoA reductase inhibitors | GCST007931 | 132367 | NA | NA | NA | 0,000310887 | 41,16336891 |
| 49 | 3 | rs645040 | 135926622 | G | T | 0,225004 | -0,042834024 | 0,006691199 | 1,5E-10 | HMG CoA reductase inhibitors | GCST007931 | 132367 | NA | NA | NA | 0,000309496 | 40,97918047 |
| 50 | 11 | rs112771035 | 126225876 | G | C | 0,0718487 | 0,068585506 | 0,010822763 | 2,3E-10 | HMG CoA reductase inhibitors | GCST007931 | 132367 | NA | NA | NA | 0,000303303 | 40,15890168 |
| 51 | 2 | rs77673991 | 44077323 | T | C | 0,017311 | -0,136064684 | 0,021477857 | 2,4E-10 | HMG CoA reductase inhibitors | GCST007931 | 132367 | NA | NA | NA | 0,000303108 | 40,13306792 |
| 52 | 17 | rs72837687 | 7073747 | A | G | 0,199899 | -0,044388387 | 0,007016908 | 2,5E-10 | HMG CoA reductase inhibitors | GCST007931 | 132367 | NA | NA | NA | 0,000302229 | 40,01664373 |
| 53 | 8 | rs2737245 | 116658583 | T | G | 0,278192 | -0,039369802 | 0,006276604 | 3,6E-10 | HMG CoA reductase inhibitors | GCST007931 | 132367 | NA | NA | NA | 0,000297145 | 39,34327316 |
| 54 | 6 | rs2066905 | 16131297 | C | G | 0,45116 | -0,035224747 | 0,005632965 | 4E-10 | HMG CoA reductase inhibitors | GCST007931 | 132367 | NA | NA | NA | 0,000295334 | 39,1034538 |
| 55 | 10 | rs2068888 | 94839642 | A | G | 0,450113 | -0,035113056 | 0,005626034 | 4,3E-10 | HMG CoA reductase inhibitors | GCST007931 | 132367 | NA | NA | NA | 0,000294188 | 38,9516648 |
| 56 | 8 | rs268 | 19813529 | G | A | 0,0185977 | 0,128529373 | 0,0206911 | 5,2E-10 | HMG CoA reductase inhibitors | GCST007931 | 132367 | NA | NA | NA | 0,000291428 | 38,5861172 |
| 57 | 15 | rs10468017 | 58678512 | T | C | 0,297184 | 0,037635972 | 0,006131474 | 8,3E-10 | HMG CoA reductase inhibitors | GCST007931 | 132367 | NA | NA | NA | 0,00028456 | 37,67644442 |
| 58 | 19 | rs10413278 | 11355752 | G | A | 0,497904 | 0,034844859 | 0,005710159 | 0,000000001 | HMG CoA reductase inhibitors | GCST007931 | 132367 | NA | NA | NA | 0,000281241 | 37,23697961 |
| 59 | 19 | rs73013176 | 11147526 | C | T | 0,0107678 | -0,165802759 | 0,027140769 | 0,000000001 | HMG CoA reductase inhibitors | GCST007931 | 132367 | NA | NA | NA | 0,000281862 | 37,31922827 |
| 60 | 4 | rs3135064 | 3285389 | T | C | 0,450203 | -0,034444179 | 0,005658626 | 1,2E-09 | HMG CoA reductase inhibitors | GCST007931 | 132367 | NA | NA | NA | 0,000279839 | 37,05127666 |
| 61 | 7 | rs17725246 | 44581986 | C | T | 0,183202 | 0,043688347 | 0,007218703 | 1,4E-09 | HMG CoA reductase inhibitors | GCST007931 | 132367 | NA | NA | NA | 0,000276639 | 36,6274217 |
| 62 | 1 | rs112339942 | 55703776 | G | A | 0,0454208 | 0,081106968 | 0,013422398 | 1,5E-09 | HMG CoA reductase inhibitors | GCST007931 | 132367 | NA | NA | NA | 0,000275776 | 36,51317245 |
| 63 | 16 | rs11864054 | 30846134 | G | A | 0,385154 | -0,034885282 | 0,005811639 | 1,9E-09 | HMG CoA reductase inhibitors | GCST007931 | 132367 | NA | NA | NA | 0,000272138 | 36,03136301 |
| 64 | 1 | rs12725767 | 6676040 | G | A | 0,354298 | 0,035128029 | 0,005856082 | 0,000000002 | HMG CoA reductase inhibitors | GCST007931 | 132367 | NA | NA | NA | 0,000271766 | 35,98211142 |
| 65 | 2 | rs4541244 | 58917535 | T | C | 0,421979 | -0,034275082 | 0,005711376 | 0,000000002 | HMG CoA reductase inhibitors | GCST007931 | 132367 | NA | NA | NA | 0,000272005 | 36,01379764 |
| 66 | 19 | rs187429064 | 19380513 | G | A | 0,0111654 | -0,160271645 | 0,026744215 | 2,1E-09 | HMG CoA reductase inhibitors | GCST007931 | 132367 | NA | NA | NA | 0,000271241 | 35,91262123 |
| 67 | 4 | rs13125101 | 81174592 | A | G | 0,292467 | 0,03686969 | 0,006180362 | 2,4E-09 | HMG CoA reductase inhibitors | GCST007931 | 132367 | NA | NA | NA | 0,000268791 | 35,58808039 |
| 68 | 17 | rs740516 | 67082962 | G | C | 0,148541 | -0,047164632 | 0,007900013 | 2,4E-09 | HMG CoA reductase inhibitors | GCST007931 | 132367 | NA | NA | NA | 0,000269203 | 35,64271169 |
| 69 | 16 | rs11641811 | 71635836 | C | A | 0,476408 | -0,033493562 | 0,005619208 | 2,5E-09 | HMG CoA reductase inhibitors | GCST007931 | 132367 | NA | NA | NA | 0,000268334 | 35,52759561 |
| 70 | 5 | rs11743303 | 55859952 | G | A | 0,208058 | 0,041238974 | 0,006954898 | 0,000000003 | HMG CoA reductase inhibitors | GCST007931 | 132367 | NA | NA | NA | 0,000265546 | 35,15827888 |
| 71 | 9 | rs34631598 | 19322511 | A | G | 0,0768843 | 0,062131128 | 0,010502716 | 3,3E-09 | HMG CoA reductase inhibitors | GCST007931 | 132367 | NA | NA | NA | 0,000264314 | 34,99521443 |
| 72 | 6 | rs577721086 | 127440047 | C | T | 0,0483191 | 0,077321853 | 0,013085579 | 3,4E-09 | HMG CoA reductase inhibitors | GCST007931 | 132367 | NA | NA | NA | 0,000263709 | 34,91500334 |
| 73 | 17 | rs7213086 | 45752310 | C | G | 0,44498 | -0,033201609 | 0,005647779 | 4,1E-09 | HMG CoA reductase inhibitors | GCST007931 | 132367 | NA | NA | NA | 0,000261018 | 34,55861008 |
| 74 | 17 | rs11653468 | 47192620 | A | G | 0,233873 | -0,038664313 | 0,006608344 | 4,9E-09 | HMG CoA reductase inhibitors | GCST007931 | 132367 | NA | NA | NA | 0,000258549 | 34,23172565 |
| 75 | 2 | rs2080227 | 63081759 | T | A | 0,298602 | -0,035931189 | 0,006145548 | 0,000000005 | HMG CoA reductase inhibitors | GCST007931 | 132367 | NA | NA | NA | 0,000258184 | 34,18341005 |
| 76 | 11 | rs1938598 | 58413910 | C | T | 0,239796 | -0,038035965 | 0,006555382 | 6,5E-09 | HMG CoA reductase inhibitors | GCST007931 | 132367 | NA | NA | NA | 0,000254274 | 33,66559894 |
| 77 | 16 | rs3764261 | 56993324 | A | C | 0,323979 | -0,034744173 | 0,005988408 | 6,6E-09 | HMG CoA reductase inhibitors | GCST007931 | 132367 | NA | NA | NA | 0,000254244 | 33,66159162 |
| 78 | 3 | rs35430985 | 185488303 | A | C | 0,266787 | 0,036945826 | 0,006372475 | 6,7E-09 | HMG CoA reductase inhibitors | GCST007931 | 132367 | NA | NA | NA | 0,000253878 | 33,61305006 |
| 79 | 1 | rs570530 | 234866776 | A | G | 0,495536 | -0,032541516 | 0,005613812 | 6,8E-09 | HMG CoA reductase inhibitors | GCST007931 | 132367 | NA | NA | NA | 0,000253787 | 33,6010855 |
| 80 | 4 | rs2603192 | 69333810 | C | T | 0,413377 | -0,033421764 | 0,005784338 | 7,6E-09 | HMG CoA reductase inhibitors | GCST007931 | 132367 | NA | NA | NA | 0,000252152 | 33,38450178 |
| 81 | 7 | rs799158 | 73019074 | T | C | 0,0431865 | 0,079353562 | 0,013771121 | 8,3E-09 | HMG CoA reductase inhibitors | GCST007931 | 132367 | NA | NA | NA | 0,000250787 | 33,20379079 |
| 82 | 16 | rs2917677 | 69750849 | T | C | 0,409065 | -0,032871774 | 0,005711217 | 8,6E-09 | HMG CoA reductase inhibitors | GCST007931 | 132367 | NA | NA | NA | 0,000250208 | 33,1270188 |
| 83 | 12 | rs2293093 | 52317057 | G | C | 0,111646 | 0,051159056 | 0,008895074 | 8,9E-09 | HMG CoA reductase inhibitors | GCST007931 | 132367 | NA | NA | NA | 0,000249838 | 33,07800856 |
| 84 | 14 | rs221907 | 71596347 | A | G | 0,360751 | -0,033361871 | 0,005826718 | 0,00000001 | HMG CoA reductase inhibitors | GCST007931 | 132367 | NA | NA | NA | 0,000247608 | 32,78281616 |
| 85 | 2 | rs73228424 | 21128294 | A | G | 0,0164566 | -0,12550562 | 0,021949965 | 0,000000011 | HMG CoA reductase inhibitors | GCST007931 | 132367 | NA | NA | NA | 0,000246929 | 32,69279954 |
| 86 | 3 | rs7639816 | 150073703 | T | C | 0,180878 | 0,041868539 | 0,007355367 | 0,000000013 | HMG CoA reductase inhibitors | GCST007931 | 132367 | NA | NA | NA | 0,000244726 | 32,40114656 |
| 87 | 2 | rs148566631 | 203447875 | A | G | 0,118597 | 0,048858199 | 0,008647989 | 0,000000016 | HMG CoA reductase inhibitors | GCST007931 | 132367 | NA | NA | NA | 0,000241079 | 31,91818089 |
| 88 | 8 | rs1030431 | 59311697 | A | G | 0,33226 | 0,03366996 | 0,005988778 | 0,000000019 | HMG CoA reductase inhibitors | GCST007931 | 132367 | NA | NA | NA | 0,00023874 | 31,6083753 |
| 89 | 3 | rs34850939 | 47063910 | A | G | 0,349518 | 0,032981402 | 0,005867987 | 0,000000019 | HMG CoA reductase inhibitors | GCST007931 | 132367 | NA | NA | NA | 0,000238603 | 31,59027335 |
| 90 | 6 | rs2982521 | 139835329 | A | T | 0,372013 | 0,032467972 | 0,00578365 | 0,00000002 | HMG CoA reductase inhibitors | GCST007931 | 132367 | NA | NA | NA | 0,000238025 | 31,51372792 |
| 91 | 12 | rs11057830 | 125307053 | A | G | 0,138404 | 0,045655189 | 0,008143289 | 0,000000021 | HMG CoA reductase inhibitors | GCST007931 | 132367 | NA | NA | NA | 0,000237409 | 31,43214719 |
| 92 | 16 | rs146254843 | 72110323 | T | G | 0,0214145 | -0,107948801 | 0,019350807 | 0,000000024 | HMG CoA reductase inhibitors | GCST007931 | 132367 | NA | NA | NA | 0,000235048 | 31,11937952 |
| 93 | 15 | rs1800588 | 58723675 | T | C | 0,216347 | 0,037284391 | 0,006792414 | 0,00000004 | HMG CoA reductase inhibitors | GCST007931 | 132367 | NA | NA | NA | 0,000227576 | 30,13000565 |
| 94 | 16 | rs66502159 | 75245937 | T | C | 0,100215 | -0,05124117 | 0,009347076 | 0,000000042 | HMG CoA reductase inhibitors | GCST007931 | 132367 | NA | NA | NA | 0,000226991 | 30,05245541 |
| 95 | 2 | rs10183431 | 44213990 | T | C | 0,239911 | -0,036092561 | 0,00660173 | 0,000000046 | HMG CoA reductase inhibitors | GCST007931 | 132367 | NA | NA | NA | 0,000225757 | 29,88913368 |
| 96 | 1 | rs12739394 | 55489082 | C | G | 0,27884 | 0,034125137 | 0,006241631 | 0,000000046 | HMG CoA reductase inhibitors | GCST007931 | 132367 | NA | NA | NA | 0,000225774 | 29,89138909 |
| 97 | 2 | rs57853403 | 20728467 | T | C | 0,0600086 | 0,064235851 | 0,011784915 | 0,00000005 | HMG CoA reductase inhibitors | GCST007931 | 132367 | NA | NA | NA | 0,000224401 | 29,70951189 |

**Table S19.** SNPs selection for immunosuppressants

|  | chr.exposure | SNP | pos.exposure | effect_allele.exposure | other_allele.exposure | eaf.exposure | beta.exposure | se.exposure | pval.exposure | exposure | id.exposure | samplesize.exposure | mr_keep.exposure | pval_origin.exposure | data_source.exposure | r2 | F |
| --- | --- | --- | --- | --- | --- | --- | --- | --- | --- | --- | --- | --- | --- | --- | --- | --- | --- |
| 1 | 6 | rs687308 | 32567256 | T | C | 0,191895 | 0,56109229 | 0,028824366 | 2,1E-84 | Immunosuppressants | GCST007933 | 132367 | NA | NA | NA | 0,002854486 | 378,9156273 |
| 2 | 1 | rs6679677 | 114303808 | A | C | 0,102915 | 0,334267837 | 0,037253014 | 2,9E-19 | Immunosuppressants | GCST007933 | 132367 | NA | NA | NA | 0,000607887 | 80,51184702 |

**Table S20.** SNPs selection for opioids

|  | chr.exposure | SNP | pos.exposure | effect_allele.exposure | other_allele.exposure | eaf.exposure | beta.exposure | se.exposure | pval.exposure | exposure | id.exposure | samplesize.exposure | mr_keep.exposure | pval_origin.exposure | data_source.exposure | r2 | F |
| --- | --- | --- | --- | --- | --- | --- | --- | --- | --- | --- | --- | --- | --- | --- | --- | --- | --- |
| 1 | 9 | rs12238134 | 128746612 | A | G | 0,305094 | -0,07148008 | 0,011640261 | 8,2E-10 | Opioids | GCST007936 | 132367 | NA | NA | NA | 0,000284801 | 37,70839267 |
| 2 | 3 | rs7428430 | 50174184 | T | C | 0,482569 | -0,065191409 | 0,010717268 | 1,2E-09 | Opioids | GCST007936 | 132367 | NA | NA | NA | 0,000279455 | 37,00036352 |
| 3 | 1 | rs2618039 | 112324111 | T | A | 0,380661 | 0,061221961 | 0,011072679 | 0,000000032 | Opioids | GCST007936 | 132367 | NA | NA | NA | 0,000230903 | 30,57049714 |

**Table S21.** SNPs selection for salicylic acid and derivatives

|  | chr.exposure | SNP | pos.exposure | effect_allele.exposure | other_allele.exposure | eaf.exposure | beta.exposure | se.exposure | pval.exposure | exposure | id.exposure | samplesize.exposure | mr_keep.exposure | pval_origin.exposure | data_source.exposure | r2 | F |
| --- | --- | --- | --- | --- | --- | --- | --- | --- | --- | --- | --- | --- | --- | --- | --- | --- | --- |
| 1 | 1 | rs583104 | 109821307 | G | T | 0,224727 | -0,078039336 | 0,009467476 | 1,7E-16 | Salicylic acid and derivatives | GCST007937 | 132367 | NA | NA | NA | 0,000513046 | 67,94416674 |
| 2 | 19 | rs73015016 | 11191300 | A | G | 0,117682 | -0,095657302 | 0,012271248 | 6,4E-15 | Salicylic acid and derivatives | GCST007937 | 132367 | NA | NA | NA | 0,000458859 | 60,76481735 |
| 3 | 19 | rs7412 | 45412079 | T | C | 0,0784037 | -0,11077729 | 0,014702809 | 4,9E-14 | Salicylic acid and derivatives | GCST007937 | 132367 | NA | NA | NA | 0,000428682 | 56,7667817 |
| 4 | 8 | rs28601761 | 126500031 | G | C | 0,415104 | -0,060665793 | 0,008191943 | 1,3E-13 | Salicylic acid and derivatives | GCST007937 | 132367 | NA | NA | NA | 0,000414147 | 54,84125766 |
| 5 | 6 | rs74617384 | 160997118 | T | A | 0,0826667 | 0,105044421 | 0,014373422 | 2,7E-13 | Salicylic acid and derivatives | GCST007937 | 132367 | NA | NA | NA | 0,000403339 | 53,40957093 |
| 6 | 6 | rs140570886 | 161013013 | C | T | 0,0159652 | 0,223431599 | 0,031677394 | 1,7E-12 | Salicylic acid and derivatives | GCST007937 | 132367 | NA | NA | NA | 0,000375705 | 49,7489295 |
| 7 | 9 | rs635634 | 136155000 | T | C | 0,184501 | 0,066638239 | 0,010233878 | 7,4E-11 | Salicylic acid and derivatives | GCST007937 | 132367 | NA | NA | NA | 0,000320219 | 42,39942585 |
| 8 | 9 | rs1831733 | 22076071 | C | T | 0,483126 | 0,045792655 | 0,007975758 | 9,4E-09 | Salicylic acid and derivatives | GCST007937 | 132367 | NA | NA | NA | 0,000248977 | 32,9640936 |
| 9 | 6 | rs2523589 | 31327334 | T | G | 0,495166 | 0,044395192 | 0,007923358 | 0,000000021 | Salicylic acid and derivatives | GCST007937 | 132367 | NA | NA | NA | 0,000237121 | 31,39400562 |
| 10 | 11 | rs964184 | 116648917 | G | C | 0,13389 | 0,063261947 | 0,011595182 | 0,000000049 | Salicylic acid and derivatives | GCST007937 | 132367 | NA | NA | NA | 0,000224829 | 29,76620025 |

**Table S22.** SNPs selection for thyroid preparations

|  | chr.exposure | SNP | pos.exposure | effect_allele.exposure | other_allele.exposure | eaf.exposure | beta.exposure | se.exposure | pval.exposure | exposure | id.exposure | samplesize.exposure | mr_keep.exposure | pval_origin.exposure | data_source.exposure | r2 | F |
| --- | --- | --- | --- | --- | --- | --- | --- | --- | --- | --- | --- | --- | --- | --- | --- | --- | --- |
| 1 | 6 | rs2856698 | 32636376 | G | C | 0,328285 | 0,268845124 | 0,009665421 | 2,8E-170 | Thyroid preparations | GCST007932 | 132367 | NA | NA | NA | 0,005811015 | 773,6708041 |
| 2 | 1 | rs2476601 | 114377568 | A | G | 0,102931 | 0,413973244 | 0,014925497 | 2,6E-169 | Thyroid preparations | GCST007932 | 132367 | NA | NA | NA | 0,005778173 | 769,2728379 |
| 3 | 12 | rs3184504 | 111884608 | T | C | 0,484908 | 0,205206492 | 0,009094226 | 9,7E-113 | Thyroid preparations | GCST007932 | 132367 | NA | NA | NA | 0,003831807 | 509,1481427 |
| 4 | 9 | rs7850258 | 100549013 | A | G | 0,331608 | -0,195523605 | 0,009675413 | 8,3E-91 | Thyroid preparations | GCST007932 | 132367 | NA | NA | NA | 0,003075685 | 408,3690038 |
| 5 | 2 | rs3087243 | 204738919 | A | G | 0,448827 | -0,178713501 | 0,009145473 | 4,9E-85 | Thyroid preparations | GCST007932 | 132367 | NA | NA | NA | 0,002876548 | 381,8526445 |
| 6 | 12 | rs11066320 | 112906415 | A | G | 0,426876 | 0,17050133 | 0,009237976 | 4,6E-76 | Thyroid preparations | GCST007932 | 132367 | NA | NA | NA | 0,002566882 | 340,6397744 |
| 7 | 3 | rs12634152 | 188121019 | C | T | 0,45193 | 0,142340723 | 0,009196319 | 4,9E-54 | Thyroid preparations | GCST007932 | 132367 | NA | NA | NA | 0,001806614 | 239,5652082 |
| 8 | 1 | rs4915076 | 108359505 | C | T | 0,0891965 | 0,223871735 | 0,015949774 | 9,4E-45 | Thyroid preparations | GCST007932 | 132367 | NA | NA | NA | 0,001486154 | 197,0075518 |
| 9 | 6 | rs7754251 | 90989125 | G | C | 0,418931 | -0,120908846 | 0,009181357 | 1,3E-39 | Thyroid preparations | GCST007932 | 132367 | NA | NA | NA | 0,001308441 | 173,4186637 |
| 10 | 2 | rs11889341 | 191943742 | T | C | 0,222787 | 0,143350669 | 0,010942145 | 3,3E-39 | Thyroid preparations | GCST007932 | 132367 | NA | NA | NA | 0,001294947 | 171,6279238 |
| 11 | 13 | rs7333647 | 24786356 | C | T | 0,287791 | 0,124414188 | 0,010090322 | 6,2E-35 | Thyroid preparations | GCST007932 | 132367 | NA | NA | NA | 0,001147233 | 152,0278815 |
| 12 | 6 | rs1951459 | 167402536 | A | G | 0,334248 | -0,115464516 | 0,009609606 | 2,9E-33 | Thyroid preparations | GCST007932 | 132367 | NA | NA | NA | 0,001089514 | 144,3707908 |
| 13 | 11 | rs4409785 | 95311422 | C | T | 0,172241 | 0,143064027 | 0,012049951 | 1,6E-32 | Thyroid preparations | GCST007932 | 132367 | NA | NA | NA | 0,001063772 | 140,9560644 |
| 14 | 10 | rs71508903 | 63779871 | T | C | 0,190444 | 0,139273382 | 0,011730452 | 1,6E-32 | Thyroid preparations | GCST007932 | 132367 | NA | NA | NA | 0,001063812 | 140,9613973 |
| 15 | 4 | rs7655751 | 149633421 | T | C | 0,211042 | -0,131974448 | 0,011143826 | 2,3E-32 | Thyroid preparations | GCST007932 | 132367 | NA | NA | NA | 0,001058453 | 140,2505239 |
| 16 | 12 | rs12423545 | 9939224 | T | C | 0,325966 | 0,107592163 | 0,009872058 | 1,2E-27 | Thyroid preparations | GCST007932 | 132367 | NA | NA | NA | 0,000896554 | 118,7789037 |
| 17 | 22 | rs2858483 | 37586672 | C | A | 0,425467 | 0,1004399 | 0,009228291 | 1,4E-27 | Thyroid preparations | GCST007932 | 132367 | NA | NA | NA | 0,000894132 | 118,4577223 |
| 18 | 13 | rs76428106 | 28604007 | C | T | 0,01095 | 0,467726731 | 0,04388013 | 1,6E-26 | Thyroid preparations | GCST007932 | 132367 | NA | NA | NA | 0,000857623 | 113,6166586 |
| 19 | 2 | rs11675342 | 1407628 | T | C | 0,423396 | 0,09530659 | 0,009231734 | 5,5E-25 | Thyroid preparations | GCST007932 | 132367 | NA | NA | NA | 0,000804544 | 106,5792724 |
| 20 | 8 | rs2921053 | 8319963 | C | G | 0,451729 | -0,092992823 | 0,009178196 | 4E-24 | Thyroid preparations | GCST007932 | 132367 | NA | NA | NA | 0,00077494 | 102,6544188 |
| 21 | 10 | rs7090530 | 6110875 | C | A | 0,395908 | -0,093977185 | 0,00931912 | 6,5E-24 | Thyroid preparations | GCST007932 | 132367 | NA | NA | NA | 0,000767683 | 101,6924091 |
| 22 | 6 | rs6914622 | 148514301 | T | G | 0,321667 | 0,089143108 | 0,009792307 | 8,8E-20 | Thyroid preparations | GCST007932 | 132367 | NA | NA | NA | 0,000625682 | 82,87030234 |
| 23 | 11 | rs10836367 | 35314455 | C | T | 0,388263 | 0,083144647 | 0,0093819 | 7,8E-19 | Thyroid preparations | GCST007932 | 132367 | NA | NA | NA | 0,000592993 | 78,53811363 |
| 24 | 2 | rs1534430 | 12644736 | T | C | 0,389139 | -0,08207014 | 0,00935578 | 1,8E-18 | Thyroid preparations | GCST007932 | 132367 | NA | NA | NA | 0,000581003 | 76,94913747 |
| 25 | 15 | rs8043085 | 38828140 | T | G | 0,232174 | 0,094800679 | 0,010843896 | 2,3E-18 | Thyroid preparations | GCST007932 | 132367 | NA | NA | NA | 0,000577061 | 76,4267895 |
| 26 | 4 | rs3775291 | 187004074 | T | C | 0,297329 | -0,086711872 | 0,009948768 | 2,9E-18 | Thyroid preparations | GCST007932 | 132367 | NA | NA | NA | 0,000573574 | 75,96471534 |
| 27 | 19 | rs7254729 | 4832362 | T | C | 0,406483 | 0,078747633 | 0,009273444 | 2E-17 | Thyroid preparations | GCST007932 | 132367 | NA | NA | NA | 0,000544473 | 72,10847681 |
| 28 | 3 | rs13090803 | 105934953 | T | G | 0,21057 | 0,094675173 | 0,011272253 | 4,5E-17 | Thyroid preparations | GCST007932 | 132367 | NA | NA | NA | 0,000532647 | 70,5414237 |
| 29 | 9 | rs970987 | 21585265 | C | A | 0,337145 | 0,079637028 | 0,009665984 | 1,7E-16 | Thyroid preparations | GCST007932 | 132367 | NA | NA | NA | 0,000512549 | 67,87837182 |
| 30 | 4 | rs7441808 | 26090375 | G | A | 0,301669 | 0,08148547 | 0,009910206 | 2E-16 | Thyroid preparations | GCST007932 | 132367 | NA | NA | NA | 0,000510497 | 67,60649995 |
| 31 | 5 | rs244690 | 133421744 | G | A | 0,123038 | 0,112015565 | 0,013847775 | 6E-16 | Thyroid preparations | GCST007932 | 132367 | NA | NA | NA | 0,000494086 | 65,43200019 |
| 32 | 17 | rs35776863 | 7226957 | A | G | 0,229451 | 0,088579869 | 0,010950075 | 6E-16 | Thyroid preparations | GCST007932 | 132367 | NA | NA | NA | 0,00049413 | 65,43789942 |
| 33 | 6 | rs9391997 | 409119 | A | G | 0,470259 | -0,073869488 | 0,009155586 | 7,1E-16 | Thyroid preparations | GCST007932 | 132367 | NA | NA | NA | 0,000491547 | 65,09555344 |
| 34 | 4 | rs9291444 | 10713674 | T | C | 0,471241 | 0,07350931 | 0,009131422 | 8,3E-16 | Thyroid preparations | GCST007932 | 132367 | NA | NA | NA | 0,000489345 | 64,8039191 |
| 35 | 19 | rs11085727 | 10466123 | T | C | 0,291692 | -0,080379486 | 0,010054665 | 1,3E-15 | Thyroid preparations | GCST007932 | 132367 | NA | NA | NA | 0,000482576 | 63,90703064 |
| 36 | 10 | rs10761620 | 64057202 | A | G | 0,449981 | 0,072898789 | 0,009220965 | 2,7E-15 | Thyroid preparations | GCST007932 | 132367 | NA | NA | NA | 0,000471958 | 62,5001918 |
| 37 | 18 | rs6505765 | 12782849 | G | C | 0,344388 | 0,075718868 | 0,009598114 | 3E-15 | Thyroid preparations | GCST007932 | 132367 | NA | NA | NA | 0,000469951 | 62,23431013 |
| 38 | 10 | rs744253 | 6393009 | A | G | 0,258633 | -0,081497927 | 0,010382308 | 4,2E-15 | Thyroid preparations | GCST007932 | 132367 | NA | NA | NA | 0,00046529 | 61,61673928 |
| 39 | 19 | rs11666808 | 18383506 | T | C | 0,373327 | -0,07337282 | 0,009466754 | 9,1E-15 | Thyroid preparations | GCST007932 | 132367 | NA | NA | NA | 0,000453619 | 60,07056244 |
| 40 | 12 | rs7302200 | 56449435 | A | G | 0,340319 | 0,074568815 | 0,009635056 | 1E-14 | Thyroid preparations | GCST007932 | 132367 | NA | NA | NA | 0,000452303 | 59,8962268 |
| 41 | 17 | rs12325861 | 40289412 | C | T | 0,166127 | 0,0937906 | 0,012218159 | 1,6E-14 | Thyroid preparations | GCST007932 | 132367 | NA | NA | NA | 0,000444973 | 58,92512381 |
| 42 | 22 | rs2412970 | 30486826 | G | A | 0,444091 | -0,069808676 | 0,009166328 | 2,6E-14 | Thyroid preparations | GCST007932 | 132367 | NA | NA | NA | 0,000437984 | 57,99912168 |
| 43 | 17 | rs73316435 | 45329477 | T | C | 0,179924 | 0,090641418 | 0,011906228 | 2,7E-14 | Thyroid preparations | GCST007932 | 132367 | NA | NA | NA | 0,000437658 | 57,95600332 |
| 44 | 8 | rs11784499 | 11834539 | G | T | 0,487653 | 0,069232981 | 0,009128636 | 3,3E-14 | Thyroid preparations | GCST007932 | 132367 | NA | NA | NA | 0,000434356 | 57,51851795 |
| 45 | 8 | rs2445610 | 128197088 | G | A | 0,36907 | -0,069989502 | 0,009426584 | 1,1E-13 | Thyroid preparations | GCST007932 | 132367 | NA | NA | NA | 0,000416291 | 55,12525286 |
| 46 | 8 | rs853320 | 133918528 | G | A | 0,379935 | 0,069425326 | 0,009383118 | 1,4E-13 | Thyroid preparations | GCST007932 | 132367 | NA | NA | NA | 0,000413411 | 54,74379334 |
| 47 | 13 | rs9533100 | 42996548 | G | T | 0,4624 | -0,067726902 | 0,009159497 | 1,4E-13 | Thyroid preparations | GCST007932 | 132367 | NA | NA | NA | 0,000412876 | 54,67296065 |
| 48 | 2 | rs2111485 | 163110536 | A | G | 0,391151 | -0,068354703 | 0,009314726 | 2,2E-13 | Thyroid preparations | GCST007932 | 132367 | NA | NA | NA | 0,000406668 | 53,85053905 |
| 49 | 3 | rs145268310 | 12310773 | C | G | 0,121225 | 0,102305218 | 0,013963637 | 2,4E-13 | Thyroid preparations | GCST007932 | 132367 | NA | NA | NA | 0,000405362 | 53,67745203 |
| 50 | 8 | rs7831557 | 10280228 | G | A | 0,482275 | -0,066134966 | 0,009150161 | 4,9E-13 | Thyroid preparations | GCST007932 | 132367 | NA | NA | NA | 0,000394506 | 52,23940295 |
| 51 | 3 | rs4688013 | 119229486 | A | G | 0,191322 | 0,083276851 | 0,011549827 | 5,6E-13 | Thyroid preparations | GCST007932 | 132367 | NA | NA | NA | 0,000392597 | 51,98655866 |
| 52 | 4 | rs13136820 | 40307564 | C | T | 0,319992 | 0,070655075 | 0,009885372 | 8,8E-13 | Thyroid preparations | GCST007932 | 132367 | NA | NA | NA | 0,000385792 | 51,08508355 |
| 53 | 12 | rs1320344 | 103887477 | A | G | 0,314107 | 0,069094347 | 0,009798616 | 1,8E-12 | Thyroid preparations | GCST007932 | 132367 | NA | NA | NA | 0,000375502 | 49,72204674 |
| 54 | 5 | rs1991797 | 102622453 | T | G | 0,328168 | -0,067278454 | 0,009731536 | 4,7E-12 | Thyroid preparations | GCST007932 | 132367 | NA | NA | NA | 0,000360955 | 47,7950182 |
| 55 | 20 | rs6111715 | 17860022 | C | G | 0,179159 | -0,082008659 | 0,011882613 | 5,1E-12 | Thyroid preparations | GCST007932 | 132367 | NA | NA | NA | 0,000359716 | 47,63091473 |
| 56 | 5 | rs10036386 | 76543603 | T | C | 0,381268 | 0,064672821 | 0,009382382 | 5,5E-12 | Thyroid preparations | GCST007932 | 132367 | NA | NA | NA | 0,000358824 | 47,51282468 |
| 57 | 8 | rs7005834 | 134214204 | T | C | 0,307655 | -0,066507467 | 0,009841599 | 1,4E-11 | Thyroid preparations | GCST007932 | 132367 | NA | NA | NA | 0,000344889 | 45,66704588 |
| 58 | 1 | rs61776678 | 38377021 | A | G | 0,410504 | -0,061821667 | 0,009273056 | 2,6E-11 | Thyroid preparations | GCST007932 | 132367 | NA | NA | NA | 0,000335668 | 44,44563464 |
| 59 | 16 | rs8056260 | 67476572 | G | A | 0,0435819 | 0,148567829 | 0,022284839 | 2,6E-11 | Thyroid preparations | GCST007932 | 132367 | NA | NA | NA | 0,000335664 | 44,44510859 |
| 60 | 11 | rs174599 | 61621556 | C | G | 0,374506 | -0,062605112 | 0,009437661 | 3,3E-11 | Thyroid preparations | GCST007932 | 132367 | NA | NA | NA | 0,000332328 | 44,00320282 |
| 61 | 8 | rs7823699 | 141635061 | A | G | 0,255166 | 0,06977586 | 0,010554763 | 3,8E-11 | Thyroid preparations | GCST007932 | 132367 | NA | NA | NA | 0,000330058 | 43,70256139 |
| 62 | 3 | rs11714050 | 39357107 | G | A | 0,126095 | 0,090516447 | 0,01369856 | 3,9E-11 | Thyroid preparations | GCST007932 | 132367 | NA | NA | NA | 0,000329748 | 43,66149456 |
| 63 | 3 | rs111793536 | 121738203 | A | G | 0,0626354 | 0,124473258 | 0,018882099 | 4,3E-11 | Thyroid preparations | GCST007932 | 132367 | NA | NA | NA | 0,000328193 | 43,45552377 |
| 64 | 19 | rs12980063 | 50196992 | G | A | 0,391542 | -0,061521228 | 0,009363134 | 5E-11 | Thyroid preparations | GCST007932 | 132367 | NA | NA | NA | 0,000326051 | 43,17188057 |
| 65 | 11 | rs2282655 | 93911856 | T | C | 0,0725154 | -0,115793217 | 0,01761873 | 5E-11 | Thyroid preparations | GCST007932 | 132367 | NA | NA | NA | 0,000326209 | 43,19271339 |
| 66 | 21 | rs2823272 | 16798586 | A | T | 0,314683 | -0,063779944 | 0,009841599 | 9,1E-11 | Thyroid preparations | GCST007932 | 132367 | NA | NA | NA | 0,00031719 | 41,99817032 |
| 67 | 14 | rs3784099 | 68749927 | A | G | 0,28051 | -0,064911779 | 0,01013818 | 1,5E-10 | Thyroid preparations | GCST007932 | 132367 | NA | NA | NA | 0,000309608 | 40,99401546 |
| 68 | 6 | rs3008034 | 166043862 | C | T | 0,305208 | -0,063043782 | 0,00985865 | 1,6E-10 | Thyroid preparations | GCST007932 | 132367 | NA | NA | NA | 0,000308842 | 40,89244107 |
| 69 | 16 | rs8054578 | 79316815 | A | G | 0,223812 | 0,069831447 | 0,010947476 | 1,8E-10 | Thyroid preparations | GCST007932 | 132367 | NA | NA | NA | 0,000307299 | 40,68810929 |
| 70 | 8 | rs1561924 | 129569371 | A | G | 0,126271 | -0,087239883 | 0,01375763 | 2,3E-10 | Thyroid preparations | GCST007932 | 132367 | NA | NA | NA | 0,000303691 | 40,21021071 |
| 71 | 21 | rs12482947 | 43852037 | T | C | 0,398927 | -0,059588803 | 0,009418694 | 2,5E-10 | Thyroid preparations | GCST007932 | 132367 | NA | NA | NA | 0,000302299 | 40,02592351 |
| 72 | 5 | rs12697352 | 35837234 | A | G | 0,33964 | -0,060907224 | 0,009645008 | 2,7E-10 | Thyroid preparations | GCST007932 | 132367 | NA | NA | NA | 0,000301177 | 39,87731259 |
| 73 | 1 | rs12742404 | 200811765 | G | A | 0,121153 | 0,087284889 | 0,013958012 | 4E-10 | Thyroid preparations | GCST007932 | 132367 | NA | NA | NA | 0,00029534 | 39,10429423 |
| 74 | 10 | rs7088058 | 124149352 | T | C | 0,412751 | -0,057947976 | 0,009273498 | 4,1E-10 | Thyroid preparations | GCST007932 | 132367 | NA | NA | NA | 0,000294905 | 39,04656493 |
| 75 | 7 | rs10279209 | 37424240 | A | G | 0,134841 | 0,083503352 | 0,013371091 | 4,2E-10 | Thyroid preparations | GCST007932 | 132367 | NA | NA | NA | 0,000294555 | 39,00026476 |
| 76 | 3 | rs13076468 | 5022961 | C | A | 0,228471 | 0,067697167 | 0,010835083 | 4,2E-10 | Thyroid preparations | GCST007932 | 132367 | NA | NA | NA | 0,000294828 | 39,03641737 |
| 77 | 2 | rs7583027 | 62544391 | A | C | 0,357133 | -0,059402887 | 0,009520573 | 4,4E-10 | Thyroid preparations | GCST007932 | 132367 | NA | NA | NA | 0,000294023 | 38,9298189 |
| 78 | 11 | rs61907718 | 128172470 | A | G | 0,216956 | 0,06928254 | 0,011124578 | 4,7E-10 | Thyroid preparations | GCST007932 | 132367 | NA | NA | NA | 0,000292937 | 38,78590535 |
| 79 | 2 | rs5865 | 98373006 | C | T | 0,334138 | 0,06017709 | 0,009663693 | 4,8E-10 | Thyroid preparations | GCST007932 | 132367 | NA | NA | NA | 0,000292866 | 38,77658285 |
| 80 | 2 | rs13412022 | 8468210 | T | G | 0,330147 | -0,060663042 | 0,009756008 | 5E-10 | Thyroid preparations | GCST007932 | 132367 | NA | NA | NA | 0,00029201 | 38,66317437 |
| 81 | 16 | rs1872691 | 50350210 | A | G | 0,181059 | -0,073665624 | 0,011845738 | 5E-10 | Thyroid preparations | GCST007932 | 132367 | NA | NA | NA | 0,000292078 | 38,67220263 |
| 82 | 1 | rs61778692 | 38650752 | G | A | 0,245765 | -0,065728844 | 0,010585182 | 5,3E-10 | Thyroid preparations | GCST007932 | 132367 | NA | NA | NA | 0,000291212 | 38,55749469 |
| 83 | 2 | rs7596240 | 242444173 | G | A | 0,270163 | 0,063452716 | 0,01022874 | 5,5E-10 | Thyroid preparations | GCST007932 | 132367 | NA | NA | NA | 0,000290637 | 38,48128441 |
| 84 | 8 | rs1032129 | 119951900 | C | A | 0,353542 | -0,059290909 | 0,009567829 | 5,8E-10 | Thyroid preparations | GCST007932 | 132367 | NA | NA | NA | 0,000290031 | 38,40103035 |
| 85 | 3 | rs114558062 | 187641130 | C | T | 0,0122204 | 0,256125702 | 0,041433623 | 6,3E-10 | Thyroid preparations | GCST007932 | 132367 | NA | NA | NA | 0,000288599 | 38,21148638 |
| 86 | 1 | rs1723022 | 167405418 | T | G | 0,370454 | 0,059215498 | 0,009605119 | 7E-10 | Thyroid preparations | GCST007932 | 132367 | NA | NA | NA | 0,000287052 | 38,00657318 |
| 87 | 1 | rs10489626 | 67793171 | G | C | 0,163317 | 0,075578895 | 0,012304875 | 8,1E-10 | Thyroid preparations | GCST007932 | 132367 | NA | NA | NA | 0,000284933 | 37,72594153 |
| 88 | 4 | rs6833591 | 123546282 | G | A | 0,346625 | -0,058954707 | 0,009598676 | 8,2E-10 | Thyroid preparations | GCST007932 | 132367 | NA | NA | NA | 0,000284912 | 37,7231304 |
| 89 | 9 | rs7028486 | 5387279 | C | T | 0,301266 | 0,06116547 | 0,009969597 | 8,5E-10 | Thyroid preparations | GCST007932 | 132367 | NA | NA | NA | 0,000284285 | 37,64010971 |
| 90 | 1 | rs2234167 | 2494330 | A | G | 0,133222 | 0,081784837 | 0,013360309 | 9,3E-10 | Thyroid preparations | GCST007932 | 132367 | NA | NA | NA | 0,000283015 | 37,47192835 |
| 91 | 18 | rs56249713 | 67533332 | C | T | 0,418115 | -0,057151405 | 0,009335863 | 9,3E-10 | Thyroid preparations | GCST007932 | 132367 | NA | NA | NA | 0,000283036 | 37,47471621 |
| 92 | 1 | rs72922276 | 65429319 | A | G | 0,108775 | -0,089580171 | 0,014685601 | 1,1E-09 | Thyroid preparations | GCST007932 | 132367 | NA | NA | NA | 0,000281021 | 37,20777983 |
| 93 | 18 | rs7240256 | 77174220 | C | T | 0,317032 | 0,05994282 | 0,009862602 | 1,2E-09 | Thyroid preparations | GCST007932 | 132367 | NA | NA | NA | 0,000278991 | 36,9389715 |
| 94 | 6 | rs9469899 | 34793124 | A | G | 0,358222 | 0,057007816 | 0,009489203 | 1,9E-09 | Thyroid preparations | GCST007932 | 132367 | NA | NA | NA | 0,000272591 | 36,09132055 |
| 95 | 11 | rs7931398 | 116879676 | C | T | 0,259045 | -0,062308691 | 0,010408629 | 2,1E-09 | Thyroid preparations | GCST007932 | 132367 | NA | NA | NA | 0,000270653 | 35,83469194 |
| 96 | 11 | rs479777 | 64107477 | C | T | 0,340767 | -0,057627312 | 0,009627233 | 2,2E-09 | Thyroid preparations | GCST007932 | 132367 | NA | NA | NA | 0,000270618 | 35,83002943 |
| 97 | 2 | rs112165453 | 204664815 | T | C | 0,0163088 | 0,214980462 | 0,036061623 | 2,5E-09 | Thyroid preparations | GCST007932 | 132367 | NA | NA | NA | 0,000268418 | 35,53864588 |
| 98 | 10 | rs7893467 | 6079035 | G | T | 0,071527 | 0,104973002 | 0,01764793 | 2,7E-09 | Thyroid preparations | GCST007932 | 132367 | NA | NA | NA | 0,000267222 | 35,38026956 |
| 99 | 2 | rs10202630 | 191262925 | C | T | 0,480418 | 0,05442924 | 0,009156577 | 2,8E-09 | Thyroid preparations | GCST007932 | 132367 | NA | NA | NA | 0,000266872 | 35,33390617 |
| 100 | 14 | rs1257926 | 98692996 | A | G | 0,474691 | 0,054403924 | 0,009154541 | 2,8E-09 | Thyroid preparations | GCST007932 | 132367 | NA | NA | NA | 0,000266742 | 35,31674908 |
| 101 | 10 | rs586511 | 6448894 | G | A | 0,264667 | 0,060740463 | 0,010303375 | 3,7E-09 | Thyroid preparations | GCST007932 | 132367 | NA | NA | NA | 0,000262484 | 34,75286828 |
| 102 | 5 | rs13360007 | 156577720 | G | A | 0,135676 | 0,077983611 | 0,013293483 | 4,5E-09 | Thyroid preparations | GCST007932 | 132367 | NA | NA | NA | 0,000259918 | 34,41299869 |
| 103 | 9 | rs2274780 | 127075021 | C | T | 0,470695 | 0,053449968 | 0,009119769 | 4,6E-09 | Thyroid preparations | GCST007932 | 132367 | NA | NA | NA | 0,000259439 | 34,34951767 |
| 104 | 19 | rs72977594 | 1187675 | A | G | 0,149821 | -0,075491429 | 0,0128825 | 4,6E-09 | Thyroid preparations | GCST007932 | 132367 | NA | NA | NA | 0,00025936 | 34,33906287 |
| 105 | 8 | rs4733823 | 129014697 | T | C | 0,456248 | 0,053653162 | 0,009174084 | 0,000000005 | Thyroid preparations | GCST007932 | 132367 | NA | NA | NA | 0,000258329 | 34,20256494 |
| 106 | 3 | rs2969903 | 108279216 | T | C | 0,233721 | 0,062769596 | 0,010745875 | 5,2E-09 | Thyroid preparations | GCST007932 | 132367 | NA | NA | NA | 0,000257705 | 34,11995942 |
| 107 | 5 | rs114378220 | 110566360 | T | C | 0,0637665 | 0,1107751 | 0,01908583 | 6,5E-09 | Thyroid preparations | GCST007932 | 132367 | NA | NA | NA | 0,000254432 | 33,68648052 |
| 108 | 6 | rs71571466 | 167342767 | A | G | 0,0627323 | 0,108549603 | 0,018716142 | 6,6E-09 | Thyroid preparations | GCST007932 | 132367 | NA | NA | NA | 0,000254059 | 33,63700128 |
| 109 | 10 | rs10748781 | 101283330 | C | A | 0,434051 | 0,053942885 | 0,009322589 | 7,2E-09 | Thyroid preparations | GCST007932 | 132367 | NA | NA | NA | 0,000252875 | 33,48025325 |
| 110 | 11 | rs10751647 | 306884 | C | T | 0,40255 | -0,05415626 | 0,009375309 | 7,6E-09 | Thyroid preparations | GCST007932 | 132367 | NA | NA | NA | 0,000252021 | 33,36718266 |
| 111 | 22 | rs5758311 | 41759375 | T | G | 0,228031 | -0,062579796 | 0,010870163 | 8,6E-09 | Thyroid preparations | GCST007932 | 132367 | NA | NA | NA | 0,000250327 | 33,14283165 |
| 112 | 1 | rs2473808 | 19638883 | T | C | 0,33565 | 0,055621753 | 0,009684348 | 9,3E-09 | Thyroid preparations | GCST007932 | 132367 | NA | NA | NA | 0,00024915 | 32,98694125 |
| 113 | 7 | rs34591253 | 128721877 | T | C | 0,135132 | 0,076426098 | 0,013319536 | 9,6E-09 | Thyroid preparations | GCST007932 | 132367 | NA | NA | NA | 0,000248667 | 32,92294155 |
| 114 | 4 | rs113473633 | 103449131 | G | A | 0,0221276 | -0,178552767 | 0,031151332 | 9,9E-09 | Thyroid preparations | GCST007932 | 132367 | NA | NA | NA | 0,000248138 | 32,85287403 |
| 115 | 1 | rs484959 | 110366083 | T | C | 0,463768 | -0,052000816 | 0,009112308 | 0,000000012 | Thyroid preparations | GCST007932 | 132367 | NA | NA | NA | 0,000245967 | 32,5654392 |
| 116 | 4 | rs366327 | 87980030 | T | A | 0,184729 | 0,06757675 | 0,011895218 | 0,000000013 | Thyroid preparations | GCST007932 | 132367 | NA | NA | NA | 0,000243761 | 32,27329093 |
| 117 | 8 | rs7017073 | 129204262 | C | T | 0,213216 | -0,063424989 | 0,011146197 | 0,000000013 | Thyroid preparations | GCST007932 | 132367 | NA | NA | NA | 0,000244558 | 32,37880502 |
| 118 | 7 | rs1734911 | 100308811 | C | G | 0,113645 | -0,081313484 | 0,01433346 | 0,000000014 | Thyroid preparations | GCST007932 | 132367 | NA | NA | NA | 0,000243074 | 32,18225931 |
| 119 | 9 | rs2094920 | 110669755 | C | G | 0,449845 | 0,052081451 | 0,009170454 | 0,000000014 | Thyroid preparations | GCST007932 | 132367 | NA | NA | NA | 0,000243612 | 32,25357973 |
| 120 | 5 | rs13358767 | 72180703 | G | A | 0,285135 | -0,057781751 | 0,01020388 | 0,000000015 | Thyroid preparations | GCST007932 | 132367 | NA | NA | NA | 0,000242195 | 32,06595127 |
| 121 | 8 | rs10098103 | 23397081 | C | T | 0,242332 | -0,060081989 | 0,010637018 | 0,000000016 | Thyroid preparations | GCST007932 | 132367 | NA | NA | NA | 0,000240971 | 31,90378634 |
| 122 | 16 | rs142997491 | 50729820 | G | A | 0,0119418 | 0,236767964 | 0,041971547 | 0,000000017 | Thyroid preparations | GCST007932 | 132367 | NA | NA | NA | 0,000240354 | 31,8221389 |
| 123 | 11 | rs59183580 | 95425470 | G | A | 0,130354 | 0,075851339 | 0,01350742 | 0,00000002 | Thyroid preparations | GCST007932 | 132367 | NA | NA | NA | 0,000238176 | 31,53371277 |
| 124 | 7 | rs215634 | 32369148 | A | G | 0,38727 | 0,052579995 | 0,009410992 | 0,000000023 | Thyroid preparations | GCST007932 | 132367 | NA | NA | NA | 0,00023577 | 31,21502316 |
| 125 | 11 | rs3781627 | 47441472 | T | A | 0,299038 | 0,056455427 | 0,010139171 | 0,000000026 | Thyroid preparations | GCST007932 | 132367 | NA | NA | NA | 0,000234167 | 31,00272754 |
| 126 | 2 | rs10172181 | 55891140 | C | T | 0,0535539 | 0,111839025 | 0,020185921 | 0,00000003 | Thyroid preparations | GCST007932 | 132367 | NA | NA | NA | 0,000231851 | 30,69609031 |
| 127 | 3 | rs74378591 | 188007689 | C | G | 0,108871 | 0,081116183 | 0,014673144 | 0,000000032 | Thyroid preparations | GCST007932 | 132367 | NA | NA | NA | 0,000230828 | 30,5606145 |
| 128 | 1 | rs75972122 | 1151973 | C | G | 0,151208 | -0,070190554 | 0,012701915 | 0,000000033 | Thyroid preparations | GCST007932 | 132367 | NA | NA | NA | 0,000230642 | 30,53601511 |
| 129 | 10 | rs8177788 | 5994841 | T | C | 0,0101659 | -0,251848837 | 0,045567302 | 0,000000033 | Thyroid preparations | GCST007932 | 132367 | NA | NA | NA | 0,000230724 | 30,54686979 |
| 130 | 1 | rs810048 | 117310296 | T | C | 0,0796453 | -0,091661275 | 0,016748757 | 0,000000044 | Thyroid preparations | GCST007932 | 132367 | NA | NA | NA | 0,000226219 | 29,95022442 |
| 131 | 6 | rs1724088 | 36978745 | A | G | 0,284883 | 0,055100037 | 0,01007757 | 0,000000046 | Thyroid preparations | GCST007932 | 132367 | NA | NA | NA | 0,000225795 | 29,8941059 |
| 132 | 5 | rs28674017 | 142856120 | T | C | 0,288697 | -0,054981897 | 0,010067752 | 0,000000047 | Thyroid preparations | GCST007932 | 132367 | NA | NA | NA | 0,000225267 | 29,82413652 |

**Table S23.** SNPs selection for thyroid preparations

|  | chr.exposure | SNP | pos.exposure | effect_allele.exposure | other_allele.exposure | eaf.exposure | beta.exposure | se.exposure | pval.exposure | exposure | id.exposure | samplesize.exposure | mr_keep.exposure | pval_origin.exposure | data_source.exposure | r2 | F |
| --- | --- | --- | --- | --- | --- | --- | --- | --- | --- | --- | --- | --- | --- | --- | --- | --- | --- |
| 1 | 9 | rs1333047 | 22124504 | T | A | 0,492809 | 0,165319679 | 0,019089253 | 4,7E-18 | Vasodilators used in cardiac diseases | GCST007925 | 132367 | NA | NA | NA | 0,000566299 | 75,00059186 |
| 2 | 6 | rs55730499 | 161005610 | T | C | 0,0811461 | 0,298491837 | 0,034986724 | 1,4E-17 | Vasodilators used in cardiac diseases | GCST007925 | 132367 | NA | NA | NA | 0,000549591 | 72,78666059 |
| 3 | 6 | rs140570886 | 161013013 | C | T | 0,0155529 | 0,495162446 | 0,077368712 | 1,6E-10 | Vasodilators used in cardiac diseases | GCST007925 | 132367 | NA | NA | NA | 0,00030935 | 40,95982559 |

**Supplementary Table 24. Statistical Power of Significant Associations between Drug Exposure and Dementia/Cognitive Outcomes (with R², N, and K values)**

| Exposure | Outcome | IVW_OR | Exposure_R2 | N | K (case/case+control) | Power |
| --- | --- | --- | --- | --- | --- | --- |
| Drugs used in diabetes | Vascular dementia | 1.063398 | 0.027147 | 132367 | 0.006866 | 6.13% |
| Drugs used in diabetes | Dementia due to Parkinsons disease | 0.870815 | 0.027147 | 132367 | 0.003191 | 7.21% |
| Drugs used in diabetes | frontotemporal dementia | 1.001291 | 0.027147 | 132367 | 0.000329 | 5.00% |
| Drugs used in diabetes | Dementia in Alzheimer disease | 1.071735 | 0.024606 | 132367 | 0.015569 | 7.98% |
| Agents acting on the renin-angiotensin system | Vascular dementia | 1.092562 | 0.071346 | 132367 | 0.006866 | 11.51% |
| Agents acting on the renin-angiotensin system | Dementia due to Parkinsons disease | 1.107805 | 0.072006 | 132367 | 0.003191 | 9.12% |
| Agents acting on the renin-angiotensin system | frontotemporal dementia | 1.033998 | 0.072006 | 132367 | 0.000329 | 5.04% |
| Agents acting on the renin-angiotensin system | Dementia in Alzheimer disease | 0.957299 | 0.072006 | 132367 | 0.015569 | 8.11% |
| Thyroid preparations | Vascular dementia | 0.963292 | 0.077421 | 132367 | 0.006866 | 6.09% |
| Thyroid preparations | Dementia due to Parkinsons disease | 0.982053 | 0.077421 | 132367 | 0.003191 | 5.12% |
| Thyroid preparations | frontotemporal dementia | 1.033692 | 0.077421 | 132367 | 0.000329 | 5.04% |
| Thyroid preparations | Dementia in Alzheimer disease | 1.017319 | 0.077421 | 132367 | 0.015569 | 5.54% |
| Adrenergics inhalants | Vascular dementia | 0.921828 | 0.026426 | 132367 | 0.006866 | 6.69% |
| Adrenergics inhalants | Dementia due to Parkinsons disease | 1.122384 | 0.026426 | 132367 | 0.003191 | 6.93% |
| Adrenergics inhalants | frontotemporal dementia | 1.019298 | 0.026426 | 132367 | 0.000329 | 5.00% |
| Adrenergics inhalants | Dementia in Alzheimer disease | 0.95705 | 0.026426 | 132367 | 0.015569 | 6.14% |
| Diuretics | Vascular dementia | 1.128205 | 0.039784 | 132367 | 0.006866 | 11.98% |
| Diuretics | Dementia due to Parkinsons disease | 0.933569 | 0.039784 | 132367 | 0.003191 | 5.85% |
| Diuretics | frontotemporal dementia | 1.062175 | 0.039784 | 132367 | 0.000329 | 5.08% |
| Diuretics | Dementia in Alzheimer disease | 0.920564 | 0.039784 | 132367 | 0.015569 | 11.02% |
| HMG CoA reductase inhibitors | Vascular dementia | 1.140002 | 0.064765 | 132367 | 0.006866 | 18.78% |
| HMG CoA reductase inhibitors | Dementia due to Parkinsons disease | 0.85596 | 0.046997 | 132367 | 0.003191 | 9.82% |
| HMG CoA reductase inhibitors | frontotemporal dementia | 1.009556 | 0.064765 | 132367 | 0.000329 | 5.00% |
| HMG CoA reductase inhibitors | Dementia in Alzheimer disease | 1.036297 | 0.054403 | 132367 | 0.015569 | 6.68% |
| Beta blocking agents | Vascular dementia | 1.132375 | 0.019697 | 132367 | 0.006866 | 8.63% |
| Beta blocking agents | Dementia due to Parkinsons disease | 1.006248 | 0.019697 | 132367 | 0.003191 | 5.00% |
| Beta blocking agents | frontotemporal dementia | 1.059738 | 0.019697 | 132367 | 0.000329 | 5.04% |
| Beta blocking agents | Dementia in Alzheimer disease | 0.968473 | 0.019697 | 132367 | 0.015569 | 5.46% |
| Calcium channel blockers | Vascular dementia | 1.146342 | 0.038306 | 132367 | 0.006866 | 13.80% |
| Calcium channel blockers | Dementia due to Parkinsons disease | 1.135206 | 0.038601 | 132367 | 0.003191 | 8.46% |
| Calcium channel blockers | frontotemporal dementia | 1.037451 | 0.038601 | 132367 | 0.000329 | 5.03% |
| Calcium channel blockers | Dementia in Alzheimer disease | 0.994793 | 0.038601 | 132367 | 0.015569 | 5.02% |
| Antiglaucoma preparations and miotics | Vascular dementia | 1.039764 | 0.00559 | 132367 | 0.006866 | 5.09% |
| Antiglaucoma preparations and miotics | Dementia due to Parkinsons disease | 1.026173 | 0.00559 | 132367 | 0.003191 | 5.02% |
| Antiglaucoma preparations and miotics | frontotemporal dementia | 1.014163 | 0.00559 | 132367 | 0.000329 | 5.00% |
| Antiglaucoma preparations and miotics | Dementia in Alzheimer disease | 1.009173 | 0.00559 | 132367 | 0.015569 | 5.01% |
| Drugs affecting bone structure and mineralization | Vascular dementia | 0.884177 | 0.003063 | 132367 | 0.006866 | 5.43% |
| Drugs affecting bone structure and mineralization | Dementia due to Parkinsons disease | 0.877655 | 0.003063 | 132367 | 0.003191 | 5.22% |
| Drugs affecting bone structure and mineralization | frontotemporal dementia | 1.053002 | 0.002271 | 132367 | 0.000329 | 5.00% |
| Drugs affecting bone structure and mineralization | Dementia in Alzheimer disease | 0.925963 | 0.003063 | 132367 | 0.015569 | 5.39% |
| Opioids | Vascular dementia | 1.21927 | 0.000795 | 132367 | 0.006866 | 5.40% |
| Opioids | Dementia due to Parkinsons disease | 2.640631 | 0.000795 | 132367 | 0.003191 | 15.77% |
| Opioids | frontotemporal dementia | 0.939163 | 0.000795 | 132367 | 0.000329 | 5.00% |
| Opioids | Dementia in Alzheimer disease | 1.358227 | 0.000795 | 132367 | 0.015569 | 7.38% |
| Antimigraine preparations | Vascular dementia | 0.915075 | 0.003383 | 132367 | 0.006866 | 5.25% |
| Antimigraine preparations | Dementia due to Parkinsons disease | 0.967765 | 0.004422 | 132367 | 0.003191 | 5.02% |
| Antimigraine preparations | frontotemporal dementia | 0.962123 | 0.004422 | 132367 | 0.000329 | 5.00% |
| Antimigraine preparations | Dementia in Alzheimer disease | 1.056884 | 0.004422 | 132367 | 0.015569 | 5.33% |
| Vasodilators used in cardiac diseases | Vascular dementia | 0.896296 | 0.001425 | 132367 | 0.006866 | 5.16% |
| Vasodilators used in cardiac diseases | Dementia due to Parkinsons disease | 1.712025 | 0.001425 | 132367 | 0.003191 | 8.54% |
| Vasodilators used in cardiac diseases | frontotemporal dementia | 1.115278 | 0.001425 | 132367 | 0.000329 | 5.01% |
| Vasodilators used in cardiac diseases | Dementia in Alzheimer disease | 0.97616 | 0.001425 | 132367 | 0.015569 | 5.02% |
| Antithrombotic agents | Vascular dementia | 0.951576 | 0.005879 | 132367 | 0.006866 | 5.14% |
| Antithrombotic agents | Dementia due to Parkinsons disease | 1.049703 | 0.005879 | 132367 | 0.003191 | 5.07% |
| Antithrombotic agents | frontotemporal dementia | 1.066207 | 0.005879 | 132367 | 0.000329 | 5.01% |
| Antithrombotic agents | Dementia in Alzheimer disease | 1.22265 | 0.005879 | 132367 | 0.015569 | 11.96% |
| Glucocorticoids | Vascular dementia | 1.003016 | 0.009634 | 132367 | 0.006866 | 5.00% |
| Glucocorticoids | Dementia due to Parkinsons disease | 1.122072 | 0.008019 | 132367 | 0.003191 | 5.58% |
| Glucocorticoids | frontotemporal dementia | 1.025527 | 0.009634 | 132367 | 0.000329 | 5.00% |
| Glucocorticoids | Dementia in Alzheimer disease | 1.012271 | 0.009634 | 132367 | 0.015569 | 5.03% |
| Antihypertensives | Vascular dementia | 1.422696 | 0.001006 | 132367 | 0.006866 | 6.87% |
| Antihypertensives | Dementia due to Parkinsons disease | 1.305675 | 0.001006 | 132367 | 0.003191 | 5.45% |
| Antihypertensives | frontotemporal dementia | 1.127667 | 0.001006 | 132367 | 0.000329 | 5.01% |
| Antihypertensives | Dementia in Alzheimer disease | 0.825735 | 0.001006 | 132367 | 0.015569 | 5.72% |
| Salicylic acid and derivatives | Vascular dementia | 1.040236 | 0.003625 | 132367 | 0.006866 | 5.06% |
| Salicylic acid and derivatives | Dementia due to Parkinsons disease | 1.106551 | 0.003625 | 132367 | 0.003191 | 5.20% |
| Salicylic acid and derivatives | frontotemporal dementia | 1.130111 | 0.003625 | 132367 | 0.000329 | 5.03% |
| Salicylic acid and derivatives | Dementia in Alzheimer disease | 1.402304 | 0.003625 | 132367 | 0.015569 | 19.21% |
| Antihistamines for systemic use | Vascular dementia | 0.898245 | 0.002304 | 132367 | 0.006866 | 5.25% |
| Antihistamines for systemic use | Dementia due to Parkinsons disease | 0.775033 | 0.002304 | 132367 | 0.003191 | 5.57% |
| Antihistamines for systemic use | frontotemporal dementia | 1.01998 | 0.002304 | 132367 | 0.000329 | 5.00% |
| Antihistamines for systemic use | Dementia in Alzheimer disease | 1.024688 | 0.002304 | 132367 | 0.015569 | 5.03% |
| Antiinflammatroy and antirheumatic products non-steroids | Vascular dementia | 1.0296 | 0.001736 | 132367 | 0.006866 | 5.02% |
| Antiinflammatroy and antirheumatic products non-steroids | Dementia due to Parkinsons disease | 0.737693 | 0.001736 | 132367 | 0.003191 | 5.58% |
| Antiinflammatroy and antirheumatic products non-steroids | frontotemporal dementia | 1.191654 | 0.001736 | 132367 | 0.000329 | 5.03% |
| Antiinflammatroy and antirheumatic products non-steroids | Dementia in Alzheimer disease | 1.38419 | 0.001736 | 132367 | 0.015569 | 11.07% |
| Anilides | Vascular dementia | 0.833772 | 0.002411 | 132367 | 0.006866 | 5.69% |
| Anilides | Dementia due to Parkinsons disease | 0.436898 | 0.002411 | 132367 | 0.003191 | 8.78% |
| Anilides | frontotemporal dementia | 1.123781 | 0.002411 | 132367 | 0.000329 | 5.02% |
| Anilides | Dementia in Alzheimer disease | 0.761168 | 0.002411 | 132367 | 0.015569 | 8.28% |
| Drugs for peptic ulcer and gastro-oesophageal reflux disease (GORD) | Vascular dementia | 1.548859 | 0.001383 | 132367 | 0.006866 | 9.38% |
| Drugs for peptic ulcer and gastro-oesophageal reflux disease (GORD) | Dementia due to Parkinsons disease | 0.723355 | 0.001383 | 132367 | 0.003191 | 5.51% |
| Drugs for peptic ulcer and gastro-oesophageal reflux disease (GORD) | frontotemporal dementia | 0.949694 | 0.001383 | 132367 | 0.000329 | 5.00% |
| Drugs for peptic ulcer and gastro-oesophageal reflux disease (GORD) | Dementia in Alzheimer disease | 1.144716 | 0.001383 | 132367 | 0.015569 | 5.67% |
| Immunosuppressants | Vascular dementia | 0.892803 | 0.003462 | 132367 | 0.006866 | 5.41% |
| Immunosuppressants | Dementia due to Parkinsons disease | 0.871133 | 0.003462 | 132367 | 0.003191 | 5.28% |
| Immunosuppressants | frontotemporal dementia | 1.065695 | 0.003462 | 132367 | 0.000329 | 5.01% |
| Immunosuppressants | Dementia in Alzheimer disease | 0.960403 | 0.003462 | 132367 | 0.015569 | 5.13% |
